# Supplementary material for: Competitive interactions between corals and turf algae depend on coral colony form
Source: PeerJ. 2016 May 10;4:e1984. doi: 10.7717/peerj.1984 (PMC4867736; doi:10.7717/peerj.1984)
Supplement: Supplemental Information 1 [file peerj-04-1984-s001.pdf]

Sample #	Date	Sample picture code	Genus	Growth form	Growth form group	Size class	Largest diameter	Circumference
1	28/03/13	1.1.1	**Acropora**	Tabulate	Plating	40-80	75	220
2	28/03/13	1.1.2	**Acropora**	Branching	Branching			
3	28/03/13	1.1.3	**Fungia**	Solitary	Solitary	10-20	15	51
4	28/03/13	1.1.4	**Fungia**	Solitary	Solitary	10-20	13	40
5	28/03/13	1.1.5	**Acropora**	Branching	Branching			
6	28/03/13	1.1.6	**Fungia**	Solitary	Solitary	5-10	8	23
7	28/03/13	1.1.7	**Porites**	Digitate	Branching	10-20	14	45
8	28/03/13	1.1.8	**Fungia**	Solitary	Solitary	10-20	12	32
9	28/03/13	1.1.9	**Porites**	Massive	Massive	5-10	7	19
10	28/03/13	1.1.10	**Montastrea**	Submassive	Massive	10-20	14	48
11	29/03/13	2.1.1	**Porites**	Columnar	Upright	20-40	31	106
12	29/03/13	2.1.2	**Pocillopora**	Corymbose	Branching	0-5	4	14
13	29/03/13	2.1.4	**Porites**	Columnar	Upright	10-20	18	76
14	29/03/13	2.1.5	**Favites**	Thin Encrusting	Encrusting	10-20	10	31
15	29/03/13	2.1.6	**Goniastrea**	Massive	Massive	5-10	8	34
16	29/03/13	2.1.8	**Porites**	Columnar	Upright	5-10	9	41
17	29/03/13	2.2.1	**Porites**	Columnar	Upright	40-80	71	252
18	29/03/13	2.2.2	**Favites**	Thick Encrusting	Encrusting	5-10	8	19
19	29/03/13	2.2.3	**Favia**	Massive	Massive	10-20	14	46
20	29/03/13	2.2.5	**Goniastrea**	Massive	Massive	5-10	9	24
21	29/03/13	2.2.6	**Favites**	Massive	Massive	10-20	15	48
22	29/03/13	2.2.7	**Favia**	Massive	Massive	10-20	12	38
23	29/03/13	2.2.9	**Porites**	Massive	Massive	0-5	4	11
24	29/03/13	2.2.10	**Goniastrea**	Massive	Massive	0-5	4	10
25	29/03/13	2.3.1	**Favia**	Thick Encrusting	Encrusting	5-10	8	21
26	29/03/13	2.3.2	**Porites**	Massive	Massive	0-5	3	8
27	29/03/13	2.3.3	**Goniastrea**	Massive	Massive	5-10	7	21
28	29/03/13	2.3.4	**Goniastrea**	Massive	Massive	0-5	4	11
29	29/03/13	2.3.5	**Porites**	Massive	Massive	20-40	32	113
30	29/03/13	2.3.6	**Porites**	Columnar	Upright	40-80	54	154
31	29/03/13	2.3.7	**Favia**	Thick Encrusting	Encrusting	5-10	7	19
32	29/03/13	2.3.8	**Hydnophora**	Thick Encrusting	Encrusting	10-20	17	50
33	29/03/13	2.3.9	**Galaxea**	Thick Encrusting	Encrusting	10-20	11	37
34	29/03/13	2.3.10	**Porites**	Columnar	Upright	20-40	39	122
35	29/03/13	2.3.12	**Goniastrea**	Massive	Massive	0-5	3	10
36	29/03/13	2.3.13	**Porites**	Columnar	Upright	20-40	24	87
37	29/03/13	2.3.14	**Goniastrea**	Thick Encrusting	Encrusting	10-20	10	30
38	29/03/13	2.3.15	**Goniastrea**	Massive	Massive	5-10	5	15
39	29/03/13	2.3.16	**Porites**	Columnar	Upright	40-80	65	210
40	30/03/13	3.1.1	**Porites**	Submassive	Massive	10-20	11	36
41	30/03/13	3.1.2	**Porites**	Columnar	Upright	40-80	71	236
42	30/03/13	3.1.4	**Leptastrea**	Thin Encrusting	Encrusting	5-10	9	29
43	30/03/13	3.1.5	**Fungia**	Solitary	Solitary	0-5	4	14
44	30/03/13	3.1.6	**Porites**	Massive	Massive	20-40	34	99
45	30/03/13	3.1.8	**Porites**	Massive	Massive	10-20	11	34
46	30/03/13	3.1.9	**Goniastrea**	Thick Encrusting	Encrusting	5-10	7	18
47	30/03/13	3.1.10	**Leptastrea**	Thin Encrusting	Encrusting	10-20	18	50
48	30/03/13	3.1.12	**Porites**	Thin Encrusting	Encrusting	20-40	23	80
49	30/03/13	3.1.13	**Porites**	Columnar	Upright	10-20	11	40
50	30/03/13	3.1.14	**Favia**	Massive	Massive	5-10	9	32
51	30/03/13	3.1.15	**Pavona**	Foliose	Upright	5-10	7	19
52	30/03/13	3.1.16	**Astreopora**	Massive	Massive	5-10	7	19
53	30/03/13	3.1.17	**Porites**	Thin Encrusting	Encrusting	20-40	30	104
54	30/03/13	3.1.18	**Leptastrea**	Thin Encrusting	Encrusting	5-10	7	22
55	30/03/13	3.1.19	**Leptastrea**	Thin Encrusting	Encrusting	10-20	16	47
56	30/03/13	3.1.20	**Porites**	Massive	Massive	10-20	14	39
57	30/03/13	3.1.21	**Goniastrea**	Thick Encrusting	Encrusting	5-10	5	17
58	30/03/13	3.1.22	**Cyphastrea**	Submassive	Massive	10-20	14	55
59	30/03/13	3.1.23	**Favites**	Thick Encrusting	Encrusting	5-10	9	28
60	30/03/13	3.1.24	**Favites**	Submassive	Massive	5-10	7	31
61	30/03/13	3.1.25	**Favites**	Thick Encrusting	Encrusting	10-20	12	31
62	30/03/13	3.1.26	**Leptastrea**	Thin Encrusting	Encrusting	20-40	26	95

63	30/03/13	3.1.27	**Montastrea**	Submassive	Massive	0-5	4	16
64	30/03/13	3.1.28	**Pavona**	Foliose	Upright	5-10	6	20
65	30/03/13	3.1.29	**Porites**	Submassive	Massive	0-5	3	11
66	30/03/13	3.1.30	**Favites**	Thin Encrusting	Encrusting	5-10	5	16
67	30/03/13	3.2.1	**Porites**	Thin Encrusting	Encrusting	10-20	18	72
68	30/03/13	3.2.2	**Porites**	Massive	Massive	40-80	51	162
69	30/03/13	3.2.3	**Cyphastrea**	Columnar	Upright	10-20	11	43
70	30/03/13	3.2.4	**Psammocora**	Thick Encrusting	Encrusting	5-10	9	30
71	30/03/13	3.2.5	**Porites**	Massive	Massive	5-10	8	28
72	30/03/13	3.2.6	**Porites**	Thin Encrusting	Encrusting	10-20	14	56
73	30/03/13	3.2.7	**Galaxea**	Thick Encrusting	Encrusting	5-10	5	14
74	30/03/13	3.2.8	**Favia**	Thick Encrusting	Encrusting	5-10	6	25
75	30/03/13	3.2.9	**Fungia**	Solitary	Solitary	10-20	10	35
76	30/03/13	3.2.10	**Porites**	Massive	Massive	5-10	5	14
77	30/03/13	3.2.11	**Porites**	Massive	Massive	20-40	28	91
78	30/03/13	3.2.12	**Porites**	Massive	Massive	10-20	14	39
79	30/03/13	3.2.13	**Fungia**	Solitary	Solitary	10-20	10	31
80	30/03/13	3.2.14	**Fungia**	Solitary	Solitary	10-20	11	35
81	30/03/13	3.2.15	**Fungia**	Solitary	Solitary	5-10	9	29
82	30/03/13	3.2.16	**Astreopora**	Massive	Massive	10-20	10	31
83	30/03/13	3.2.17	**Fungia**	Solitary	Solitary	10-20	12	39
84	30/03/13	3.2.18	**Galaxea**	Thick Encrusting	Encrusting	5-10	6	18
85	30/03/13	3.2.19	**Fungia**	Solitary	Solitary	10-20	14	48
86	30/03/13	3.2.20	**Fungia**	Solitary	Solitary	5-10	8	24
87	30/03/13	3.2.21	**Fungia**	Solitary	Solitary	10-20	10	32
88	30/03/13	3.2.22	**Fungia**	Solitary	Solitary	10-20	12	38
89	30/03/13	3.2.23	**Porites**	Thin Encrusting	Encrusting	5-10	6	19
90	30/03/13	3.2.24	**Fungia**	Solitary	Solitary	10-20	10	31
91	30/03/13	3.2.25	**Fungia**	Solitary	Solitary	10-20	13	41
92	30/03/13	3.2.26	**Fungia**	Solitary	Solitary	10-20	13	39
93	30/03/13	3.2.27	**Fungia**	Solitary	Solitary	10-20	12	36
94	30/03/13	3.2.29	**Ctenactis**	Solitary	Solitary	20-40	23	62
95	30/03/13	3.2.30	**Fungia**	Solitary	Solitary	5-10	9	23
96	30/03/13	3.2.31	**Favia**	Thick Encrusting	Encrusting	5-10	7	18
97	30/03/13	3.2.32	**Galaxea**	Thick Encrusting	Encrusting	5-10	5	13
98	30/03/13	3.2.33	**Fungia**	Solitary	Solitary	5-10	6	20
99	30/03/13	3.2.34	**Fungia**	Solitary	Solitary	5-10	7	23
100	30/03/13	3.2.35	**Ctenactis**	Solitary	Solitary	5-10	6	16
101	30/03/13	3.2.36	**Pavona**	Submassive	Massive	5-10	8	18
102	30/03/13	3.2.37	**Fungia**	Solitary	Solitary	10-20	17	56
103	30/03/13	3.2.38	**Montastrea**	Submassive	Massive	5-10	7	29
104	30/03/13	3.2.39	**Fungia**	Solitary	Solitary	10-20	15	46
105	30/03/13	3.2.40	**Fungia**	Solitary	Solitary	5-10	6	20
106	30/03/13	3.2.41	**Fungia**	Solitary	Solitary	10-20	18	54
107	30/03/13	3.2.43	**Porites**	Massive	Massive	0-5	3	9
108	30/03/13	3.2.45	**Porites**	Massive	Massive	10-20	13	40
109	30/03/13	3.2.46	**Acropora**	Branching	Branching			
110	30/03/13	3.2.47	**Acropora**	Branching	Branching			
111	30/03/13	3.2.48	**Acropora**	Branching	Branching			
112	30/03/13	3.2.49	**Acropora**	Branching	Branching			
113	01/04/13	4.1.1	**Fungia**	Solitary	Solitary	10-20	12	38
114	01/04/13	4.1.2	**Fungia**	Solitary	Solitary	10-20	13	43
115	01/04/13	4.1.3	**Ctenactis**	Solitary	Solitary	10-20	13	29
116	01/04/13	4.1.4	**Pavona**	Foliose	Upright	5-10	5	18
117	01/04/13	4.1.5	**Fungia**	Solitary	Solitary	10-20	15	50
118	01/04/13	4.1.6	**Fungia**	Solitary	Solitary	10-20	14	47
119	01/04/13	4.1.7	**Fungia**	Solitary	Solitary	10-20	10	31
120	01/04/13	4.1.8	**Goniastrea**	Massive	Massive	5-10	6	18
121	01/04/13	4.1.9	**Galaxea**	Thick Encrusting	Encrusting	5-10	5	16
122	01/04/13	4.1.10	**Cyphastrea**	Columnar	Upright	10-20	16	51
123	01/04/13	4.1.11	**Leptoria**	Thin Encrusting	Encrusting	10-20	17	71
124	01/04/13	4.1.12	**Cyphastrea**	Submassive	Massive	5-10	8	25
125	01/04/13	4.1.13	**Fungia**	Solitary	Solitary	5-10	5	15
126	01/04/13	4.1.15	**Porites**	Digitate	Branching	5-10	5	15
127	01/04/13	4.1.16	**Fungia**	Solitary	Solitary	0-5	4	14
128	01/04/13	4.1.17	**Fungia**	Solitary	Solitary	5-10	5	17

129	01/04/13	4.1.19	**Fungia**	Solitary	Solitary	5-10	7	20
130	01/04/13	4.1.20	**Montipora**	Digitate	Branching	0-5	3	9
131	01/04/13	4.1.21	**Fungia**	Solitary	Solitary	10-20	10	35
132	01/04/13	4.1.22	**Fungia**	Solitary	Solitary	10-20	12	39
133	01/04/13	4.1.23	**Cyphastrea**	Massive	Massive	5-10	5	17
134	01/04/13	4.1.24	**Cyphastrea**	Submassive	Massive	10-20	15	58
135	01/04/13	4.1.25	**Fungia**	Solitary	Solitary	10-20	16	50
136	01/04/13	4.1.26	**Fungia**	Solitary	Solitary	10-20	11	34
137	01/04/13	4.1.28	**Fungia**	Solitary	Solitary	10-20	13	46
138	01/04/13	4.1.29	**Fungia**	Solitary	Solitary	10-20	13	40
139	01/04/13	4.1.30	**Fungia**	Solitary	Solitary	10-20	15	44
140	01/04/13	4.1.32	**Ctenactis**	Solitary	Solitary	20-40	21	49
141	01/04/13	4.1.33	**Fungia**	Solitary	Solitary	10-20	10	29
142	01/04/13	4.1.34	**Fungia**	Solitary	Solitary	10-20	15	45
143	01/04/13	4.1.35	**Leptoria**	Thick Encrusting	Encrusting	10-20	17	70
144	01/04/13	4.1.36	**Pocillopora**	Corymbose	Branching	5-10	5	14
145	01/04/13	4.1.37	**Favites**	Submassive	Massive	10-20	15	43
146	01/04/13	4.1.38	**Fungia**	Solitary	Solitary	10-20	12	39
147	01/04/13	4.1.39	**Ctenactis**	Solitary	Solitary	20-40	21	46
148	01/04/13	4.1.40	**Porites**	Submassive	Massive	5-10	6	14
149	01/04/13	4.1.41	**Ctenactis**	Solitary	Solitary	20-40	27	72
150	01/04/13	4.1.42	**Fungia**	Solitary	Solitary	10-20	13	36
151	01/04/13	4.1.43	**Astreopora**	Submassive	Massive	5-10	7	23
152	01/04/13	4.1.44	**Fungia**	Solitary	Solitary	10-20	16	49
153	01/04/13	4.1.45	**Fungia**	Solitary	Solitary	10-20	16	47
154	01/04/13	4.1.46	**Fungia**	Solitary	Solitary	10-20	15	48
155	01/04/13	4.1.47	**Fungia**	Solitary	Solitary	10-20	13	41
156	01/04/13	4.1.48	**Fungia**	Solitary	Solitary	10-20	15	49
157	01/04/13	4.1.49	**Fungia**	Solitary	Solitary	5-10	9	30
158	01/04/13	4.1.50	**Fungia**	Solitary	Solitary	10-20	15	45
159	01/04/13	4.1.51	**Ctenactis**	Solitary	Solitary	10-20	13	51
160	01/04/13	4.1.52	**Fungia**	Solitary	Solitary	10-20	12	41
161	01/04/13	4.1.53	**Fungia**	Solitary	Solitary	10-20	14	43
162	01/04/13	4.1.54	**Fungia**	Solitary	Solitary	10-20	14	43
163	01/04/13	4.1.55	**Fungia**	Solitary	Solitary	5-10	7	24
164	01/04/13	4.1.56	**Fungia**	Solitary	Solitary	10-20	16	50
165	01/04/13	4.1.57	**Fungia**	Solitary	Solitary	10-20	13	49
166	01/04/13	4.1.58	**Fungia**	Solitary	Solitary	10-20	12	37
167	01/04/13	4.1.59	**Fungia**	Solitary	Solitary	10-20	12	39
168	01/04/13	4.1.63	**Fungia**	Solitary	Solitary	10-20	10	39
169	01/04/13	4.2.1	**Acropora**	Branching	Branching			
170	01/04/13	4.2.2	**Acropora**	Branching	Branching			
171	01/04/13	4.2.3	**Acropora**	Branching	Branching			
172	01/04/13	4.2.4	**Favites**	Massive	Massive	0-5	4	12
173	01/04/13	4.2.5	**Porites**	Submassive	Massive	0-5	4	14
174	01/04/13	4.2.6	**Favites**	Massive	Massive	5-10	5	18
175	01/04/13	4.2.7	**Porites**	Thin Encrusting	Encrusting	20-40	29	76
176	01/04/13	4.2.9	**Porites**	Massive	Massive	10-20	13	39
177	01/04/13	4.2.10	**Favites**	Submassive	Massive	5-10	8	36
178	01/04/13	4.2.11	**Leptastrea**	Thin Encrusting	Encrusting	5-10	9	25
179	01/04/13	4.2.13	**Montastrea**	Submassive	Massive	10-20	11	31
180	01/04/13	4.2.14	**Pavona**	Thin Encrusting	Encrusting	0-5	4	11
181	01/04/13	4.2.15	**Leptastrea**	Thick Encrusting	Encrusting	0-5	4	11
182	01/04/13	4.2.16	**Porites**	Thin Encrusting	Encrusting	10-20	16	57
183	01/04/13	4.2.17	**Leptoria**	Thin Encrusting	Encrusting	20-40	26	78
184	01/04/13	4.2.18	**Porites**	Thick Encrusting	Encrusting	20-40	27	67
185	01/04/13	4.2.19	**Porites**	Massive	Massive	0-5	4	11
186	01/04/13	4.2.20	**Porites**	Massive	Massive	10-20	10	30
187	01/04/13	4.2.21	**Cyphastrea**	Massive	Massive	0-5	3	9
188	01/04/13	4.2.22	**Acropora**	Branching	Branching			
189	01/04/13	4.2.23	**Acropora**	Branching	Branching			
190	01/04/13	4.2.24	**Galaxea**	Thick Encrusting	Encrusting	10-20	12	40
191	01/04/13	4.2.25	**Porites**	Massive	Massive	10-20	19	61
192	01/04/13	4.3.1	**Montipora**	Digitate	Branching	10-20	15	55
193	01/04/13	4.3.3	**Porites**	Massive	Massive	10-20	14	39
194	01/04/13	4.3.4	**Porites**	Massive	Massive	10-20	16	47

195	01/04/13	4.3.5	**Montipora**	Digitate	Branching	5-10	7	17
196	01/04/13	4.3.7	**Porites**	Massive	Massive	0-5	3	14
197	01/04/13	4.3.8	**Porites**	Massive	Massive	10-20	13	39
198	01/04/13	4.3.9	**Favites**	Thick Encrusting	Encrusting	0-5	3	9
199	01/04/13	4.3.10	**Porites**	Massive	Massive	10-20	10	29
200	01/04/13	4.3.12	**Porites**	Massive	Massive	10-20	11	38
201	01/04/13	4.3.13	**Leptastrea**	Thick Encrusting	Encrusting	5-10	7	24
202	01/04/13	4.3.14	**Leptoria**	Thin Encrusting	Encrusting	10-20	13	38
203	01/04/13	4.3.15	**Porites**	Massive	Massive	5-10	9	28
204	01/04/13	4.3.16	**Favites**	Submassive	Massive	0-5	4	12
205	01/04/13	4.3.17	**Porites**	Massive	Massive	80+	84	253
206	01/04/13	4.3.19	**Porites**	Massive	Massive	10-20	13	4
207	01/04/13	4.3.20	**Turbinaria**	Thick Encrusting	Encrusting	5-10	7	22
208	03/04/13	5.1.1	**Acropora**	Branching	Branching			
209	03/04/13	5.1.2	**Pocillopora**	Corymbose	Branching	10-20	10	32
210	03/04/13	5.1.3	**Acropora**	Branching	Branching			
211	03/04/13	5.1.4	**Acropora**	Branching	Branching			
212	03/04/13	5.1.5	**Acropora**	Branching	Branching			
213	03/04/13	5.1.6	**Acropora**	Branching	Branching			
214	03/04/13	5.1.7	**Pocillopora**	Corymbose	Branching	0-5	3	9
215	03/04/13	5.1.10	**Favia**	Thick Encrusting	Encrusting	0-5	4	12
216	03/04/13	5.1.12	**Porites**	Thin Encrusting	Encrusting	20-40	21	75
217	03/04/13	5.1.13	**Porites**	Thin Encrusting	Encrusting	5-10	6	20
218	03/04/13	5.1.14	**Pavona**	Foliose	Upright	10-20	14	42
219	03/04/13	5.1.15	**Pavona**	Foliose	Upright	5-10	7	21
220	03/04/13	5.1.16	**Leptastrea**	Thick Encrusting	Encrusting	20-40	26	85
221	03/04/13	5.1.17	**Pocillopora**	Corymbose	Branching	20-40	22	61
222	03/04/13	5.1.18	**Acropora**	Plating	Plating	10-20	18	58
223	03/04/13	5.1.19	**Leptastrea**	Thick Encrusting	Encrusting	5-10	8	19
224	03/04/13	5.1.20	**Pocillopora**	Corymbose	Branching	5-10	6	17
225	03/04/13	5.1.21	**Acropora**	Tabulate	Plating	40-80	43	123
226	03/04/13	5.1.22	**Favia**	Thick Encrusting	Encrusting	10-20	13	43
227	03/04/13	5.1.23	**Acropora**	Plating	Plating	20-40	21	66
228	03/04/13	5.1.24	**Acropora**	Tabulate	Plating	40-80	46	143
229	03/04/13	5.2.1	**Porites**	Columnar	Upright	20-40	25	107
230	03/04/13	5.2.2	**Pocillopora**	Corymbose	Branching	10-20	13	42
231	03/04/13	5.2.5	**Acropora**	Tabulate	Plating	40-80	66	216
232	03/04/13	5.2.6	**Goniastrea**	Thick Encrusting	Encrusting	10-20	10	32
233	03/04/13	5.2.8	**Favia**	Massive	Massive	10-20	18	53
234	03/04/13	5.2.9	**Pocillopora**	Corymbose	Branching	10-20	18	51
235	03/04/13	5.2.10	**Porites**	Massive	Massive	0-5	4	12
236	03/04/13	5.2.13	**Porites**	Columnar	Upright	10-20	10	33
237	03/04/13	5.2.14	**Porites**	Massive	Massive	20-40	27	84
238	03/04/13	5.2.15	**Porites**	Massive	Massive	20-40	20	66
239	03/04/13	5.2.17	**Galaxea**	Thin Encrusting	Encrusting	10-20	12	34
240	03/04/13	5.2.18	**Porites**	Massive	Massive	20-40	23	70
241	03/04/13	5.3.2	**Porites**	Columnar	Upright	20-40	31	106
242	03/04/13	5.3.3	**Pocillopora**	Corymbose	Branching	5-10	5	13
243	03/04/13	5.3.4	**Porites**	Massive	Massive	5-10	6	15
244	03/04/13	5.3.6	**Porites**	Columnar	Upright	5-10	8	20
245	03/04/13	5.3.7	**Porites**	Massive	Massive	0-5	4	13
246	03/04/13	5.3.8	**Porites**	Columnar	Upright	10-20	15	47
247	03/04/13	5.3.9	**Favites**	Massive	Massive	20-40	25	77
248	03/04/13	5.3.11	**Favites**	Submassive	Massive	5-10	6	16
249	03/04/13	5.3.12	**Porites**	Massive	Massive	40-80	71	226
250	03/04/13	5.3.13	**Porites**	Massive	Massive	20-40	25	97
251	03/04/13	5.3.14	**Porites**	Massive	Massive	5-10	6	21
252	03/04/13	5.3.15	**Favites**	Massive	Massive	20-40	23	86
253	03/04/13	5.3.16	**Porites**	Columnar	Upright	20-40	22	86
254	03/04/13	5.3.17	**Porites**	Digitate	Branching	10-20	13	46
255	03/04/13	5.3.19	**Favites**	Massive	Massive	5-10	6	17
256	03/04/13	5.3.20	**Porites**	Columnar	Upright	20-40	36	102
257	03/04/13	5.3.21	**Porites**	Massive	Massive	5-10	8	27
258	03/04/13	5.3.22	**Porites**	Digitate	Branching	10-20	11	41
259	03/04/13	5.3.23	**Porites**	Columnar	Upright	10-20	13	38
260	03/04/13	5.3.24	**Porites**	Submassive	Massive	20-40	24	74

261	03/04/13	5.3.25	**Porites**	Digitate	Branching	5-10	7	21
262	03/04/13	5.3.26	**Porites**	Columnar	Upright	20-40	27	88
263	03/04/13	5.3.27	**Favites**	Thick Encrusting	Encrusting	5-10	6	19
264	03/04/13	5.3.28	**Porites**	Massive	Massive	5-10	5	13
265	03/04/13	5.3.29	**Porites**	Columnar	Upright	10-20	16	60
266	03/04/13	5.3.30	**Porites**	Columnar	Upright	20-40	25	83
267	03/04/13	5.3.31	**Favites**	Thin Encrusting	Encrusting	5-10	9	27
268	03/04/13	5.3.32	**Porites**	Submassive	Massive	5-10	9	28
269	03/04/13	5.4.1	**Porites**	Columnar	Upright	20-40	26	88
270	03/04/13	5.4.2	**Goniopora**	Massive	Massive	20-40	25	68
271	03/04/13	5.4.3	**Porites**	Massive	Massive	5-10	5	16
272	03/04/13	5.4.4	**Favites**	Thick Encrusting	Encrusting	0-5	4	13
273	03/04/13	5.4.5	**Porites**	Digitate	Branching	10-20	11	36
274	03/04/13	5.4.6	**Montastrea**	Massive	Massive	10-20	17	50
275	03/04/13	5.4.7	**Porites**	Columnar	Upright	20-40	25	91
276	03/04/13	5.4.8	**Porites**	Massive	Massive	5-10	5	14
277	03/04/13	5.4.9	**Porites**	Columnar	Upright	20-40	25	84
278	03/04/13	5.4.10	**Porites**	Columnar	Upright	20-40	21	77
279	03/04/13	5.4.11	**Porites**	Submassive	Massive	10-20	11	34
280	03/04/13	5.4.12	**Pavona**	Foliose	Upright	5-10	6	20
281	03/04/13	5.4.13	**Porites**	Columnar	Upright	20-40	29	105
282	03/04/13	5.4.14	**Porites**	Massive	Massive	20-40	26	83
283	03/04/13	5.4.15	**Montastrea**	Thick Encrusting	Encrusting	10-20	13	48
284	03/04/13	5.4.16	**Favia**	Thick Encrusting	Encrusting	10-20	12	44
285	03/04/13	5.4.17	**Hydnophora**	Digitate	Branching	10-20	16	45
286	03/04/13	5.4.18	**Favia**	Thick Encrusting	Encrusting	0-5	4	10
287	03/04/13	5.4.19	**Leptastrea**	Thin Encrusting	Encrusting	5-10	6	22
288	03/04/13	5.4.20	**Favia**	Thick Encrusting	Encrusting	10-20	11	31
289	03/04/13	5.4.21	**Porites**	Columnar	Upright	40-80	40	126
290	03/04/13	5.4.22	**Pocillopora**	Corymbose	Branching	0-5	3	11
291	03/04/13	5.4.23	**Pavona**	Foliose	Upright	0-5	4	13
292	03/04/13	5.4.24	**Favites**	Thick Encrusting	Encrusting	5-10	7	16
293	03/04/13	5.4.25	**Porites**	Columnar	Upright	40-80	55	200
294	03/04/13	5.4.26	**Porites**	Columnar	Upright	20-40	26	69
295	03/04/13	5.4.27	**Pocillopora**	Corymbose	Branching	0-5	3	11
296	03/04/13	5.4.28	**Favites**	Thick Encrusting	Encrusting	5-10	9	23
297	03/04/13	5.4.29	**Porites**	Digitate	Branching	80+	100	291
298	03/04/13	5.4.30	**Pocillopora**	Corymbose	Branching	5-10	5	14
299	03/04/13	5.4.31	**Favia**	Thick Encrusting	Encrusting	10-20	14	45
300	03/04/13	5.4.32	**Porites**	Massive	Massive	10-20	13	37
301	03/04/13	5.4.33	**Pocillopora**	Corymbose	Branching	10-20	14	37
302	03/04/13	5.5.1	**Favites**	Thin Encrusting	Encrusting	5-10	6	18
303	03/04/13	5.5.2	**Montipora**	Digitate	Branching	5-10	7	26
304	03/04/13	5.5.3	**Favia**	Massive	Massive	5-10	7	19
305	03/04/13	5.5.4	**Goniastrea**	Massive	Massive	0-5	3	11
306	03/04/13	5.5.5	**Favia**	Thick Encrusting	Encrusting	5-10	9	25
307	03/04/13	5.5.6	**Favia**	Thick Encrusting	Encrusting	10-20	14	33
308	03/04/13	5.5.7	**Favites**	Submassive	Massive	5-10	7	17
309	03/04/13	5.5.8	**Favites**	Thick Encrusting	Encrusting	5-10	8	25
310	03/04/13	5.5.9	**Platygyra**	Massive	Massive	10-20	16	56
311	03/04/13	5.5.11	**Galaxea**	Thick Encrusting	Encrusting	5-10	9	23
312	03/04/13	5.5.12	**Favia**	Massive	Massive	20-40	20	53
313	03/04/13	5.5.13	**Leptastrea**	Thin Encrusting	Encrusting	5-10	7	19
314	03/04/13	5.5.14	**Favites**	Thick Encrusting	Encrusting	5-10	7	25
315	03/04/13	5.5.15	**Favites**	Massive	Massive	10-20	10	32
316	03/04/13	5.5.16	**Favites**	Thick Encrusting	Encrusting	5-10	7	23
317	03/04/13	5.5.17	**Porites**	Submassive	Massive	5-10	9	29
318	03/04/13	5.5.18	**Favites**	Thick Encrusting	Encrusting	5-10	9	28
319	03/04/13	5.6.1	**Pavona**	Foliose	Upright	20-40	35	102
320	03/04/13	5.6.4	**Favia**	Thick Encrusting	Encrusting	10-20	11	27
321	03/04/13	5.6.5	**Favites**	Thick Encrusting	Encrusting	5-10	6	17
322	03/04/13	5.6.6	**Favites**	Thick Encrusting	Encrusting	10-20	11	31
323	03/04/13	5.6.7	**Montastrea**	Thin Encrusting	Encrusting	5-10	7	20
324	03/04/13	5.6.8	**Favites**	Thin Encrusting	Encrusting	0-5	4	12
325	03/04/13	5.6.9	**Goniastrea**	Thick Encrusting	Encrusting	5-10	5	17
326	03/04/13	5.6.10	**Acropora**	Tabulate	Plating	40-80	58	212

327	03/04/13	5.6.11	**Porites**	Massive	Massive	40-80	66	202
328	03/04/13	5.6.12	**Favites**	Thin Encrusting	Encrusting	10-20	11	36
329	03/04/13	5.6.14	**Leptastrea**	Thick Encrusting	Encrusting	10-20	16	43
330	03/04/13	5.6.15	**Favia**	Massive	Massive	5-10	9	24
331	03/04/13	5.6.16	**Favia**	Massive	Massive	10-20	11	38
332	03/04/13	5.6.17	**Favites**	Massive	Massive	5-10	7	24
333	03/04/13	5.6.18	**Montipora**	Thin Encrusting	Encrusting	20-40	34	104
334	03/04/13	5.6.19	**Favia**	Massive	Massive	10-20	11	28
335	03/04/13	5.6.21	**Favites**	Massive	Massive	5-10	9	25
336	03/04/13	5.6.22	**Montastrea**	Massive	Massive	10-20	17	58
337	03/04/13	5.7.2	**Montastrea**	Thick Encrusting	Encrusting	10-20	17	67
338	03/04/13	5.7.3	**Montastrea**	Massive	Massive	10-20	11	35
339	03/04/13	5.7.5	**Porites**	Thin Encrusting	Encrusting	20-40	25	121
340	03/04/13	5.7.6	**Galaxea**	Thin Encrusting	Encrusting	10-20	14	43
341	03/04/13	5.7.7	**Porites**	Digitate	Branching	40-80	67	234
342	03/04/13	5.7.8	**Montastrea**	Thin Encrusting	Encrusting	5-10	9	28
343	03/04/13	5.7.9	**Porites**	Digitate	Branching	10-20	15	40
344	03/04/13	5.7.10	**Montastrea**	Massive	Massive	5-10	5	15
345	03/04/13	5.7.12	**Pocillopora**	Corymbose	Branching	40-80	55	186
346	03/04/13	5.7.13	**Galaxea**	Thick Encrusting	Encrusting	5-10	9	25
347	03/04/13	5.7.14	**Favia**	Massive	Massive	5-10	7	24
348	03/04/13	5.7.15	**Leptastrea**	Thick Encrusting	Encrusting	5-10	7	21
349	03/04/13	5.7.16	**Favia**	Massive	Massive	5-10	7	24
350	03/04/13	5.7.17	**Favia**	Massive	Massive	10-20	12	34
351	03/04/13	5.7.19	**Favia**	Thick Encrusting	Encrusting	5-10	7	19
352	03/04/13	5.7.20	**Leptastrea**	Thin Encrusting	Encrusting	5-10	6	18
353	03/04/13	5.7.21	**Favites**	Massive	Massive	5-10	8	23
354	03/04/13	5.7.23	**Goniastrea**	Massive	Massive	5-10	9	30
355	03/04/13	5.7.24	**Leptastrea**	Thick Encrusting	Encrusting	10-20	16	48
356	03/04/13	5.7.25	**Acropora**	Corymbose	Branching	40-80	44	175
357	03/04/13	5.7.26	**Favia**	Massive	Massive	5-10	7	22
358	03/04/13	5.7.28	**Montipora**	Thin Encrusting	Encrusting	20-40	36	111
359	03/04/13	5.7.29	**Montastrea**	Thin Encrusting	Encrusting	10-20	18	54
360	03/04/13	5.7.30	**Pocillopora**	Corymbose	Branching	20-40	26	75
361	03/04/13	5.7.31	**Montipora**	Thick Encrusting	Encrusting	20-40	20	58
362	03/04/13	5.7.32	**Acropora**	Tabulate	Plating	40-80	51	152
363	03/04/13	5.7.33	**Montastrea**	Massive	Massive	5-10	7	22
364	03/04/13	5.7.36	**Montastrea**	Massive	Massive	10-20	11	39
365	03/04/13	5.7.37	**Favites**	Thick Encrusting	Encrusting	10-20	18	71
366	03/04/13	5.7.38	**Pocillopora**	Corymbose	Branching	20-40	22	65
367	03/04/13	5.8.1	**Porites**	Digitate	Branching	20-40	32	95
368	03/04/13	5.8.2	**Porites**	Massive	Massive	10-20	15	47
369	03/04/13	5.8.3	**Leptastrea**	Thin Encrusting	Encrusting	5-10	5	20
370	03/04/13	5.8.4	**Montipora**	Thin Encrusting	Encrusting	20-40	30	89
371	03/04/13	5.8.5	**Porites**	Columnar	Upright	20-40	25	84
372	03/04/13	5.8.6	**Favia**	Massive	Massive	10-20	12	31
373	03/04/13	5.8.11	**Leptastrea**	Thin Encrusting	Encrusting	5-10	9	36
374	03/04/13	5.8.12	**Favia**	Thick Encrusting	Encrusting	10-20	14	39
375	03/04/13	5.8.13	**Pocillopora**	Corymbose	Branching	20-40	24	74
376	03/04/13	5.8.14	**Favia**	Massive	Massive	10-20	10	32
377	03/04/13	5.8.15	**Favia**	Massive	Massive	10-20	10	34
378	03/04/13	6.1.1	**Montipora**	Thin Encrusting	Encrusting	20-40	21	67
379	03/04/13	6.1.2	**Porites**	Massive	Massive	20-40	22	62
380	03/04/13	6.1.4	**Porites**	Massive	Massive	5-10	5	16
381	03/04/13	6.1.5	**Porites**	Massive	Massive	5-10	6	16
382	03/04/13	6.1.7	**Porites**	Branching	Branching	5-10	8	23
383	03/04/13	6.1.8	**Galaxea**	Thick Encrusting	Encrusting	5-10	9	29
384	03/04/13	6.1.13	**Montipora**	Thin Encrusting	Encrusting	10-20	16	44
385	03/04/13	6.1.14	**Porites**	Massive	Massive	5-10	5	13
386	03/04/13	6.1.16	**Montipora**	Digitate	Branching	10-20	11	37
387	03/04/13	6.1.18	**Porites**	Thin Encrusting	Encrusting	5-10	9	33
388	03/04/13	6.1.19	**Montastrea**	Thick Encrusting	Encrusting	0-5	4	11
389	03/04/13	6.1.21	**Porites**	Thin Encrusting	Encrusting	10-20	10	41
390	03/04/13	6.1.25	**Galaxea**	Thick Encrusting	Encrusting	0-5	4	12
391	03/04/13	6.1.28	**Cyphastrea**	Thick Encrusting	Encrusting	5-10	5	13
392	03/04/13	6.1.29	**Porites**	Columnar	Upright	5-10	6	16

393	03/04/13	6.1.30	**Cyphastrea**	Massive	Massive	5-10	5	12
394	03/04/13	6.1.32	**Pavona**	Foliose	Upright	0-5	4	12
395	03/04/13	6.1.33	**Montipora**	Digitate	Branching	5-10	9	33
396	03/04/13	6.1.34	**Pocillopora**	Corymbose	Branching	10-20	13	51
397	03/04/13	6.1.35	**Galaxea**	Thick Encrusting	Encrusting	5-10	7	20
398	03/04/13	6.1.36	**Montipora**	Thin Encrusting	Encrusting	10-20	12	31
399	03/04/13	6.1.37	**Porites**	Massive	Massive	20-40	25	70
400	03/04/13	6.1.41	**Porites**	Massive	Massive	20-40	25	73
401	03/04/13	6.1.47	**Acropora**	Branching	Branching			
402	03/04/13	6.1.48	**Acropora**	Branching	Branching			
403	03/04/13	6.1.51	**Pocillopora**	Corymbose	Branching	5-10	5	14
404	03/04/13	6.1.55	**Acropora**	Branching	Branching			
405	03/04/13	6.1.56	**Acropora**	Branching	Branching			
406	03/04/13	6.1.57	**Montipora**	Thick Encrusting	Encrusting	5-10	7	20
407	03/04/13	6.1.59	**Acropora**	Branching	Branching			
408	03/04/13	6.1.60	**Pocillopora**	Corymbose	Branching	5-10	7	21
409	03/04/13	6.1.61	**Goniastrea**	Massive	Massive	5-10	6	16
410	03/04/13	6.2.1	**Pocillopora**	Corymbose	Branching	5-10	6	16
411	03/04/13	6.2.2	**Porites**	Thin Encrusting	Encrusting	5-10	5	13
412	03/04/13	6.2.3	**Galaxea**	Thick Encrusting	Encrusting	5-10	5	17
413	03/04/13	6.2.4	**Platygyra**	Massive	Massive	5-10	5	13
414	03/04/13	6.2.5	**Galaxea**	Thick Encrusting	Encrusting	5-10	7	22
415	03/04/13	6.2.6	**Acropora**	Branching	Branching			
416	03/04/13	6.2.8	**Pocillopora**	Corymbose	Branching	10-20	12	35
417	03/04/13	6.2.9	**Pocillopora**	Corymbose	Branching	10-20	13	35
418	03/04/13	6.2.10	**Montipora**	Thin Encrusting	Encrusting	5-10	9	31
419	03/04/13	6.2.13	**Porites**	Thin Encrusting	Encrusting	5-10	7	22
420	03/04/13	6.2.14	**Acropora**	Corymbose	Branching			
421	03/04/13	6.2.20	**Porites**	Columnar	Upright	5-10	6	20
422	03/04/13	6.2.22	**Galaxea**	Thick Encrusting	Encrusting	5-10	6	18
423	03/04/13	6.2.23	**Favia**	Thick Encrusting	Encrusting	5-10	5	15
424	03/04/13	6.2.24	**Montipora**	Thin Encrusting	Encrusting	10-20	16	40
425	03/04/13	6.2.25	**Pocillopora**	Corymbose	Branching	5-10	5	13
426	03/04/13	6.2.27	**Acropora**	Branching	Branching			
427	03/04/13	6.2.29	**Astreopora**	Thick Encrusting	Encrusting	0-5	3	8
428	03/04/13	6.2.30	**Porites**	Thin Encrusting	Encrusting	5-10	7	21
429	03/04/13	6.2.31	**Porites**	Branching	Branching	5-10	5	
430	03/04/13	6.2.32	**Montipora**	Digitate	Branching			
431	03/04/13	6.2.33	**Acropora**	Tabulate	Plating	40-80	47	222
432	03/04/13	6.2.34	**Cyphastrea**	Massive	Massive	10-20	12	37
433	03/04/13	6.2.35	**Porites**	Massive	Massive	10-20	11	31
434	03/04/13	6.2.36	**Porites**	Massive	Massive	5-10	5	16
435	03/04/13	6.2.37	**Pocillopora**	Corymbose	Branching	5-10	5	13
436	03/04/13	6.2.38	**Porites**	Branching	Branching			
437	03/04/13	6.2.39	**Porites**	Massive	Massive	5-10	6	18
438	03/04/13	6.2.41	**Porites**	Branching	Branching			
439	03/04/13	6.2.42	**Galaxea**	Thick Encrusting	Encrusting	5-10	7	19
440	03/04/13	6.2.43	**Porites**	Branching	Branching			
441	03/04/13	6.2.46	**Acropora**	Branching	Branching			
442	03/04/13	6.2.47	**Acropora**	Branching	Branching			
443	03/04/13	6.2.48	**Acropora**	Branching	Branching			
444	03/04/13	6.2.49	**Acropora**	Branching	Branching			
445	03/04/13	6.2.52	**Platygyra**	Thick Encrusting	Encrusting	10-20	11	37
446	03/04/13	6.2.53	**Leptastrea**	Thick Encrusting	Encrusting	0-5	4	13
447	03/04/13	6.2.55	**Porites**	Massive	Massive	10-20	10	33
448	03/04/13	6.2.56	**Leptastrea**	Thin Encrusting	Encrusting	5-10	9	30
449	03/04/13	6.2.58	**Hydnophora**	Digitate	Branching			
450	03/04/13	6.2.59	**Porites**	Massive	Massive	10-20	11	35
451	03/04/13	6.2.60	**Montipora**	Thin Encrusting	Encrusting	10-20	15	44
452	03/04/13	6.2.61	**Porites**	Massive	Massive	10-20	16	48
453	03/04/13	6.3.1	**Goniastrea**	Massive	Massive	5-10	7	20
454	03/04/13	6.3.5	**Favia**	Thick Encrusting	Encrusting	5-10	6	19
455	03/04/13	6.3.9	**Galaxea**	Thick Encrusting	Encrusting	5-10	7	18
456	03/04/13	6.3.10	**Pocillopora**	Corymbose	Branching	5-10	5	13
457	03/04/13	6.3.11	**Leptastrea**	Thick Encrusting	Encrusting	5-10	6	19
458	03/04/13	6.3.12	**Favites**	Thick Encrusting	Encrusting	10-20	10	28

459	03/04/13	6.3.13	**Leptastrea**	Thin Encrusting	Encrusting	5-10	7	17
460	03/04/13	6.3.15	**Porites**	Massive	Massive	5-10	7	21
461	03/04/13	6.3.16	**Porites**	Massive	Massive	5-10	7	23
462	03/04/13	6.3.17	**Porites**	Massive	Massive	10-20	11	32
463	03/04/13	6.3.20	**Favia**	Massive	Massive	5-10	5	15
464	03/04/13	6.3.21	**Goniastrea**	Thick Encrusting	Encrusting	10-20	12	49
465	03/04/13	6.3.22	**Porites**	Massive	Massive	0-5	4	12
466	03/04/13	6.3.24	**Montipora**	Digitate	Branching			
467	03/04/13	6.3.25	**Montastrea**	Massive	Massive	5-10	6	15
468	03/04/13	6.3.26	**Favites**	Thick Encrusting	Encrusting	5-10	5	24
469	03/04/13	6.3.29	**Galaxea**	Thick Encrusting	Encrusting	5-10	6	17
470	03/04/13	6.3.30	**Porites**	Massive	Massive	5-10	6	19
471	03/04/13	6.3.31	**Porites**	Massive	Massive	5-10	8	28
472	03/04/13	6.3.32	**Porites**	Massive	Massive	5-10	5	15
473	03/04/13	6.3.33	**Favia**	Massive	Massive	5-10	8	22
474	03/04/13	6.3.35	**Galaxea**	Thick Encrusting	Encrusting	10-20	10	43
475	03/04/13	6.3.36	**Galaxea**	Thin Encrusting	Encrusting	5-10	6	16
476	03/04/13	6.3.37	**Euphyllia**	Thin Encrusting	Encrusting	0-5	4	10
477	03/04/13	6.3.38	**Porites**	Massive	Massive	5-10	7	20
478	03/04/13	6.3.40	**Galaxea**	Thick Encrusting	Encrusting	5-10	5	16
479	03/04/13	6.3.41	**Porites**	Massive	Massive	10-20	11	35
480	03/04/13	6.3.43	**Montastrea**	Thick Encrusting	Encrusting	20-40	20	66
481	03/04/13	6.3.46	**Goniastrea**	Thick Encrusting	Encrusting	5-10	5	16
482	03/04/13	6.3.48	**Montipora**	Thin Encrusting	Encrusting	5-10	8	23
483	03/04/13	6.3.49	**Acropora**	Tabulate	Plating	40-80	63	199
484	03/04/13	6.3.50	**Favites**	Thick Encrusting	Encrusting	10-20	11	52
485	03/04/13	6.3.51	**Porites**	Thin Encrusting	Encrusting	20-40	28	80
486	03/04/13	6.3.53	**Porites**	Thick Encrusting	Encrusting	10-20	18	50
487	03/04/13	6.3.54	**Favites**	Thick Encrusting	Encrusting	5-10	7	18
488	03/04/13	6.3.55	**Leptastrea**	Thin Encrusting	Encrusting	10-20	10	36
489	03/04/13	6.3.57	**Favites**	Thick Encrusting	Encrusting	20-40	20	59
490	03/04/13	6.3.58	**Favites**	Thin Encrusting	Encrusting	10-20	19	53
491	03/04/13	6.3.59	**Porites**	Massive	Massive	10-20	11	30
492	03/04/13	6.3.60	**Porites**	Massive	Massive	10-20	13	38
493	03/04/13	6.3.61	**Montastrea**	Thick Encrusting	Encrusting	5-10	8	26
494	03/04/13	6.3.62	**Porites**	Massive	Massive	5-10	6	16
495	03/04/13	6.3.63	**Porites**	Thin Encrusting	Encrusting	10-20	13	35
496	03/04/13	6.3.64	**Acropora**	Tabulate	Plating	40-80	54	178
497	03/04/13	6.3.66	**Favia**	Thick Encrusting	Encrusting	10-20	18	53
498	03/04/13	6.3.67	**Goniopora**	Thick Encrusting	Encrusting	40-80	40	144
499	03/04/13	6.3.69	**Goniastrea**	Thick Encrusting	Encrusting	5-10	8	26
500	03/04/13	6.3.70	**Montipora**	Thin Encrusting	Encrusting	5-10	8	21
501	03/04/13	6.3.72	**Porites**	Digitate	Branching			
502	03/04/13	6.3.73	**Porites**	Massive	Massive	5-10	7	20
503	03/04/13	6.3.74	**Porites**	Digitate	Branching			
504	03/04/13	6.3.77	**Porites**	Massive	Massive	5-10	9	27
505	03/04/13	6.3.78	**Montastrea**	Thick Encrusting	Encrusting	5-10	6	19
506	03/04/13	6.3.79	**Porites**	Massive	Massive	20-40	25	78
507	03/04/13	6.3.81	**Porites**	Massive	Massive	5-10	9	25
508	03/04/13	6.3.82	**Montastrea**	Massive	Massive	10-20	17	
509	03/04/13	6.3.84	**Galaxea**	Thick Encrusting	Encrusting	5-10	7	22
510	03/04/13	6.3.85	**Porites**	Massive	Massive	10-20	16	50
511	03/04/13	6.3.86	**Goniastrea**	Thick Encrusting	Encrusting	5-10	5	15
512	03/04/13	6.3.87	**Turbinaria**	Thin Encrusting	Encrusting	10-20	19	56
513	03/04/13	6.3.89	**Cyphastrea**	Columnar	Upright	10-20	15	44
514	03/04/13	6.4.1	**Montipora**	Thin Encrusting	Encrusting	20-40	33	94
515	03/04/13	6.4.5	**Montipora**	Thin Encrusting	Encrusting	40-80	44	133
516	03/04/13	6.4.6	**Leptastrea**	Thick Encrusting	Encrusting	5-10	8	22
517	03/04/13	6.4.7	**Galaxea**	Thick Encrusting	Encrusting	5-10	5	13
518	03/04/13	6.4.8	**Porites**	Massive	Massive	10-20	13	38
519	03/04/13	6.4.9	**Leptastrea**	Columnar	Upright	10-20	17	47
520	03/04/13	6.4.12	**Pocillopora**	Corymbose	Branching	0-5	3	11
521	03/04/13	6.4.14	**Acropora**	Branching	Branching			
522	03/04/13	6.4.15	**Acropora**	Branching	Branching			
523	03/04/13	6.4.16	**Acropora**	Branching	Branching			
524	03/04/13	6.5.1	**Acropora**	Branching	Branching			

525	03/04/13	6.5.2	**Pocillopora**	Corymbose	Branching	5-10	8	21
526	03/04/13	6.5.3	**Acropora**	Corymbose	Branching			
527	03/04/13	6.5.4	**Acropora**	Corymbose	Branching	20-40	34	106
528	03/04/13	6.5.5	**Porites**	Columnar	Upright	5-10	6	19
529	03/04/13	6.5.7	**Pavona**	Foliose	Upright	10-20	11	34
530	03/04/13	6.5.8	**Cyphastrea**	Thick Encrusting	Encrusting	5-10	5	16
531	03/04/13	6.5.9	**Caulastrea**	Corymbose	Branching	20-40	20	64
532	03/04/13	6.5.10	**Porites**	Thin Encrusting	Encrusting	5-10	8	26
533	03/04/13	6.5.13	**Montipora**	Plating	Plating	10-20	14	37
534	03/04/13	6.5.16	**Favia**	Massive	Massive	5-10	6	18
535	03/04/13	6.5.17	**Pocillopora**	Corymbose	Branching	5-10	5	15
536	03/04/13	6.5.19	**Galaxea**	Thick Encrusting	Encrusting	5-10	7	17
537	03/04/13	6.5.20	**Porites**	Columnar	Upright	5-10	7	26
538	03/04/13	6.5.21	**Porites**	Massive	Massive	5-10	6	17
539	03/04/13	6.5.22	**Porites**	Massive	Massive	5-10	6	16
540	03/04/13	6.5.23	**Porites**	Massive	Massive	5-10	5	15
541	03/04/13	6.5.26	**Galaxea**	Thick Encrusting	Encrusting	5-10	6	16
542	03/04/13	6.5.28	**Pocillopora**	Corymbose	Branching	5-10	7	22
543	03/04/13	6.5.30	**Porites**	Branching	Branching			
544	03/04/13	6.5.31	**Porites**	Branching	Branching			
545	03/04/13	6.5.32	**Porites**	Branching	Branching			
546	03/04/13	6.5.33	**Galaxea**	Thin Encrusting	Encrusting	5-10	9	29
547	03/04/13	6.5.37	**Pocillopora**	Corymbose	Branching	5-10	5	13
548	03/04/13	6.5.38	**Cyphastrea**	Thin Encrusting	Encrusting	5-10	7	18
549	03/04/13	6.5.40	**Leptastrea**	Thin Encrusting	Encrusting	5-10	7	21
550	03/04/13	6.5.6.2	**Montipora**	Plating	Plating	20-40	39	125
551	03/04/13	6.6.1	**Montastrea**	Thick Encrusting	Encrusting	5-10	5	16
552	03/04/13	6.6.2	**Montipora**	Thin Encrusting	Encrusting	10-20	12	36
553	03/04/13	6.6.4	**Pocillopora**	Corymbose	Branching	10-20	13	38
554	03/04/13	6.6.5	**Galaxea**	Thick Encrusting	Encrusting	5-10	7	18
555	03/04/13	6.6.7	**Galaxea**	Thick Encrusting	Encrusting	10-20	11	30
556	03/04/13	6.6.8	**Cyphastrea**	Thick Encrusting	Encrusting	5-10	5	13
557	03/04/13	6.6.11	**Pocillopora**	Corymbose	Branching	5-10	6	17
558	03/04/13	6.6.13	**Galaxea**	Thin Encrusting	Encrusting	5-10	7	23
559	03/04/13	6.6.14	**Porites**	Digitate	Branching			
560	03/04/13	6.6.15	**Porites**	Digitate	Branching			
561	03/04/13	6.6.22	**Favia**	Massive	Massive	5-10	6	17
562	03/04/13	6.6.23	**Galaxea**	Thick Encrusting	Encrusting	5-10	5	14
563	03/04/13	6.6.25	**Pavona**	Columnar	Upright	5-10	6	19
564	03/04/13	6.6.26	**Favites**	Massive	Massive	5-10	5	15
565	03/04/13	6.6.27	**Porites**	Thin Encrusting	Encrusting	5-10	7	20
566	03/04/13	6.6.30	**Porites**	Thin Encrusting	Encrusting	10-20	17	53
567	03/04/13	6.6.30.2	**Porites**	Thin Encrusting	Encrusting	5-10	8	22
568	03/04/13	6.6.31	**Acropora**	Branching	Branching			
569	04/04/13	7.1.1	**Acropora**	Branching	Branching			
570	04/04/13	7.1.2	**Acropora**	Branching	Branching			
571	04/04/13	7.1.3	**Acropora**	Branching	Branching			
572	04/04/13	7.1.4	**Acropora**	Branching	Branching			
573	04/04/13	7.1.5	**Acropora**	Plating	Plating	20-40	25	78
574	04/04/13	7.1.6	**Acropora**	Branching	Branching			
575	04/04/13	7.1.7	**Acropora**	Branching	Branching			
576	04/04/13	7.1.8	**Acropora**	Branching	Branching			
577	04/04/13	7.1.9	**Acropora**	Branching	Branching			
578	04/04/13	7.1.10	**Acropora**	Branching	Branching			
579	04/04/13	7.1.11	**Montipora**	Thin Encrusting	Encrusting	40-80	44	139
580	04/04/13	7.1.12	**Acropora**	Branching	Branching			
581	04/04/13	7.1.13	**Acropora**	Branching	Branching			
582	04/04/13	7.1.14	**Acropora**	Branching	Branching			
583	04/04/13	7.1.15	**Acropora**	Branching	Branching			
584	04/04/13	7.1.16	**Montipora**	Thin Encrusting	Encrusting	40-80	45	136
585	04/04/13	7.1.17	**Montipora**	Thin Encrusting	Encrusting	20-40	38	122
586	04/04/13	7.1.18	**Acropora**	Branching	Branching			
587	04/04/13	7.1.19	**Acropora**	Branching	Branching			
588	04/04/13	7.1.20	**Acropora**	Branching	Branching			
589	04/04/13	7.1.21	**Acropora**	Branching	Branching			
590	04/04/13	7.1.22	**Acropora**	Plating	Plating	20-40	31	83

591	04/04/13	7.1.23	**Acropora**	Branching	Branching			
592	04/04/13	7.1.24	**Acropora**	Branching	Branching			
593	04/04/13	7.1.25	**Acropora**	Branching	Branching			
594	04/04/13	7.1.26	**Acropora**	Branching	Branching			
595	04/04/13	7.1.27	**Acropora**	Branching	Branching			
596	04/04/13	7.1.28	**Porites**	Massive	Massive	20-40	28	86
597	04/04/13	7.1.29	**Acropora**	Branching	Branching			
598	04/04/13	7.1.30.1	**Goniopora**	Massive	Massive	20-40	30	86
599	04/04/13	7.1.30	**Acropora**	Plating	Plating	20-40	28	80
600	04/04/13	7.1.31	**Acropora**	Branching	Branching			
601	04/04/13	7.2.1	**Acropora**	Branching	Branching			
602	04/04/13	7.2.2	**Acropora**	Branching	Branching			
603	04/04/13	7.2.3	**Acropora**	Branching	Branching			
604	04/04/13	7.2.4	**Acropora**	Branching	Branching			
605	04/04/13	7.2.5	**Acropora**	Branching	Branching			
606	04/04/13	7.2.6	**Acropora**	Branching	Branching			
607	04/04/13	7.2.7	**Acropora**	Branching	Branching			
608	04/04/13	7.2.8	**Acropora**	Branching	Branching			
609	04/04/13	7.2.9	**Acropora**	Branching	Branching			
610	04/04/13	7.2.10	**Acropora**	Branching	Branching			
611	04/04/13	7.2.11	**Acropora**	Branching	Branching			
612	04/04/13	7.2.11.2	**Acropora**	Branching	Branching			
613	04/04/13	7.2.12	**Acropora**	Branching	Branching			
614	04/04/13	7.2.13	**Acropora**	Branching	Branching			
615	04/04/13	7.2.14	**Acropora**	Branching	Branching			
616	04/04/13	7.2.15	**Acropora**	Branching	Branching			
617	04/04/13	7.2.16	**Acropora**	Branching	Branching			
618	04/04/13	7.2.17	**Acropora**	Branching	Branching			
619	04/04/13	7.2.18	**Acropora**	Branching	Branching			
620	04/04/13	7.2.19	**Acropora**	Branching	Branching			
621	04/04/13	7.2.20	**Acropora**	Branching	Branching			
622	04/04/13	7.2.21	**Acropora**	Branching	Branching			
623	04/04/13	7.2.22	**Acropora**	Branching	Branching			
624	04/04/13	7.2.23	**Acropora**	Branching	Branching			
625	04/04/13	7.2.24	**Acropora**	Branching	Branching			
626	04/04/13	7.2.25	**Acropora**	Branching	Branching			
627	04/04/13	7.2.26	**Acropora**	Branching	Branching			
628	04/04/13	7.2.27	**Acropora**	Branching	Branching			
629	04/04/13	7.2.28	**Acropora**	Branching	Branching			
630	04/04/13	7.2.29	**Acropora**	Branching	Branching			
631	04/04/13	7.2.30	**Acropora**	Branching	Branching			
632	04/04/13	7.2.31	**Acropora**	Branching	Branching			
633	04/04/13	7.2.32	**Acropora**	Branching	Branching			
634	04/04/13	7.2.33	**Acropora**	Branching	Branching			
635	04/04/13	7.2.34	**Acropora**	Branching	Branching			
636	04/04/13	7.2.35	**Acropora**	Branching	Branching			
637	04/04/13	7.2.36	**Acropora**	Branching	Branching			
638	04/04/13	7.2.37	**Acropora**	Branching	Branching			
639	04/04/13	7.3.1	**Acropora**	Branching	Branching			
640	04/04/13	7.3.2	**Acropora**	Branching	Branching			
641	04/04/13	7.3.3	**Acropora**	Branching	Branching			
642	04/04/13	7.3.4	**Acropora**	Branching	Branching			
643	04/04/13	7.3.5	**Acropora**	Branching	Branching			
644	04/04/13	7.3.5.2	**Acropora**	Branching	Branching			
645	04/04/13	7.3.6	**Acropora**	Branching	Branching			
646	04/04/13	7.3.7	**Acropora**	Branching	Branching			
647	05/04/13	7.3.8	**Acropora**	Branching	Branching			
648	04/04/13	7.3.9	**Acropora**	Branching	Branching			
649	04/04/13	7.3.10	**Acropora**	Branching	Branching			
650	04/04/13	7.3.11	**Acropora**	Branching	Branching			
651	04/04/13	7.3.12	**Acropora**	Branching	Branching			
652	04/04/13	7.3.13	**Acropora**	Branching	Branching			
653	04/04/13	7.3.14	**Acropora**	Branching	Branching			
654	04/04/13	7.3.15	**Acropora**	Branching	Branching			
655	04/04/13	7.3.16	**Acropora**	Branching	Branching			
656	04/04/13	7.3.17	**Acropora**	Branching	Branching			

657	04/04/13	7.3.18	**Acropora**	Branching	Branching			
658	04/04/13	7.3.20	**Acropora**	Branching	Branching			
659	04/04/13	7.3.21	**Acropora**	Branching	Branching			
660	04/04/13	7.3.22	**Acropora**	Branching	Branching			
661	04/04/13	7.3.23	**Acropora**	Branching	Branching			
662	04/04/13	7.3.24	**Acropora**	Branching	Branching			
663	04/04/13	7.3.25	**Acropora**	Branching	Branching			
664	04/04/13	7.3.26	**Acropora**	Branching	Branching			
665	04/04/13	7.3.27	**Acropora**	Branching	Branching			
666	04/04/13	7.3.28	**Acropora**	Branching	Branching			
667	04/04/13	7.3.29	**Acropora**	Branching	Branching			
668	04/04/13	7.3.30	**Acropora**	Branching	Branching			
669	04/04/13	7.3.31	**Acropora**	Branching	Branching			
670	04/04/13	7.3.32	**Acropora**	Branching	Branching			
671	04/04/13	7.3.33	**Acropora**	Branching	Branching			
672	04/04/13	7.3.34	**Acropora**	Branching	Branching			
673	04/04/13	7.3.35	**Acropora**	Branching	Branching			
674	04/04/13	7.3.36	**Acropora**	Branching	Branching			
675	04/04/13	7.3.37	**Acropora**	Branching	Branching			
676	04/04/13	7.3.38	**Acropora**	Branching	Branching			
677	04/04/13	7.4.1	**Acropora**	Branching	Branching			
678	04/04/13	7.4.2	**Acropora**	Branching	Branching			
679	04/04/13	7.4.3	**Acropora**	Branching	Branching			
680	04/04/13	7.4.4	**Acropora**	Branching	Branching			
681	04/04/13	7.4.5	**Acropora**	Branching	Branching			
682	04/04/13	7.4.6	**Acropora**	Branching	Branching			
683	04/04/13	7.4.7	**Acropora**	Branching	Branching			
684	04/04/13	7.4.8	**Acropora**	Branching	Branching			
685	04/04/13	7.4.9	**Acropora**	Branching	Branching			
686	04/04/13	7.4.10	**Acropora**	Branching	Branching			
687	04/04/13	7.4.11	**Acropora**	Branching	Branching			
688	04/04/13	7.4.12	**Acropora**	Branching	Branching			
689	04/04/13	7.4.13	**Acropora**	Branching	Branching			
690	04/04/13	7.4.14	**Acropora**	Branching	Branching			
691	04/04/13	7.4.15	**Acropora**	Branching	Branching			
692	04/04/13	7.4.17	**Acropora**	Branching	Branching			
693	04/04/13	7.4.18	**Acropora**	Branching	Branching			
694	04/04/13	7.4.19	**Acropora**	Branching	Branching			
695	04/04/13	7.4.20	**Acropora**	Branching	Branching			
696	04/04/13	7.4.21	**Acropora**	Branching	Branching			
697	04/04/13	7.4.22	**Acropora**	Branching	Branching			
698	04/04/13	7.4.23	**Acropora**	Branching	Branching			
699	04/04/13	7.4.24	**Acropora**	Branching	Branching			
700	04/04/13	7.4.25	**Acropora**	Branching	Branching			
701	04/04/13	7.4.26	**Acropora**	Branching	Branching			
702	04/04/13	7.4.27	**Acropora**	Branching	Branching			
703	04/04/13	7.4.28	**Acropora**	Branching	Branching			
704	04/04/13	7.4.29	**Acropora**	Branching	Branching			
705	04/04/13	7.4.30	**Acropora**	Branching	Branching			
706	04/04/13	7.4.31	**Acropora**	Branching	Branching			
707	04/04/13	7.4.32	**Acropora**	Branching	Branching			
708	04/04/13	7.4.33	**Acropora**	Branching	Branching			
709	04/04/13	7.4.34	**Acropora**	Branching	Branching			
710	04/04/13	7.4.35	**Acropora**	Branching	Branching			
711	04/04/13	7.4.36	**Acropora**	Branching	Branching			
712	04/04/13	7.4.37	**Acropora**	Branching	Branching			
713	04/04/13	7.4.38	**Acropora**	Branching	Branching			
714	04/04/13	7.5.1	**Acropora**	Branching	Branching			
715	04/04/13	7.5.2	**Acropora**	Branching	Branching			
716	04/04/13	7.5.3	**Acropora**	Branching	Branching			
717	04/04/13	7.5.4	**Acropora**	Branching	Branching			
718	04/04/13	7.5.5	**Acropora**	Branching	Branching			
719	04/04/13	7.5.6	**Acropora**	Branching	Branching			
720	04/04/13	7.5.7	**Acropora**	Branching	Branching			
721	04/04/13	7.5.8	**Acropora**	Branching	Branching			
722	04/04/13	7.5.8.2	**Mycedium**	Thin Encrusting	Encrusting	5-10	9	30

723	04/04/13	7.5.9	**Montipora**	Thin Encrusting	Encrusting	10-20	16	46
724	05/04/13	8.1.1	**Montastrea**	Massive	Massive	10-20	18	50
725	05/04/13	8.1.2	**Acropora**	Corymbose	Branching			
726	05/04/13	8.1.3	**Favia**	Massive	Massive	10-20	15	41
727	05/04/13	8.1.4	**Acropora**	Plating	Plating	40-80	46	139
728	05/04/13	8.1.5	**Pocillopora**	Corymbose	Branching	10-20	16	50
729	05/04/13	8.1.6	**Favia**	Massive	Massive	10-20	13	36
730	05/04/13	8.1.8	**Goniastrea**	Massive	Massive	5-10	8	27
731	05/04/13	8.1.9	**Porites**	Digitate	Branching	10-20	11	31
732	05/04/13	8.1.10	**Pocillopora**	Corymbose	Branching	10-20	18	54
733	05/04/13	8.1.11	**Porites**	Thin Encrusting	Encrusting	20-40	37	104
734	05/04/13	8.1.12	**Acropora**	Plating	Plating	20-40	25	69
735	05/04/13	8.1.14	**Pocillopora**	Corymbose	Branching	20-40	37	97
736	05/04/13	8.1.16	**Fungia**	Solitary	Solitary	20-40	21	61
737	05/04/13	8.1.17	**Cyphastrea**	Massive	Massive	10-20	16	57
738	05/04/13	8.1.18	**Montastrea**	Massive	Massive	10-20	10	28
739	05/04/13	8.1.19	**Platygyra**	Massive	Massive	5-10	7	20
740	05/04/13	8.1.20	**Montipora**	Thick Encrusting	Encrusting	10-20	12	39
741	05/04/13	8.1.21	**Favia**	Massive	Massive	20-40	20	55
742	05/04/13	8.1.22	**Goniastrea**	Massive	Massive	10-20	11	32
743	05/04/13	8.1.23	**Acropora**	Tabulate	Plating	40-80	69	225
744	05/04/13	8.1.24	**Pocillopora**	Corymbose	Branching	20-40	26	75
745	05/04/13	8.1.25	**Pocillopora**	Corymbose	Branching	20-40	39	114
746	05/04/13	8.1.26	**Pavona**	Foliose	Upright	10-20	16	54
747	05/04/13	8.1.29	**Goniastrea**	Massive	Massive	10-20	13	40
748	05/04/13	8.1.30	**Goniastrea**	Massive	Massive	10-20	11	30
749	05/04/13	8.1.34	**Favites**	Massive	Massive	5-10	8	24
750	05/04/13	8.1.36	**Favia**	Massive	Massive	10-20	15	51
751	05/04/13	8.1.38	**Pocillopora**	Corymbose	Branching	5-10	5	13
752	05/04/13	8.1.39	**Pocillopora**	Corymbose	Branching	10-20	13	44
753	05/04/13	8.1.42	**Montipora**	Digitate	Branching	20-40	25	80
754	05/04/13	8.1.43	**Pocillopora**	Corymbose	Branching	20-40	27	72
755	05/04/13	8.1.45	**Montipora**	Digitate	Branching	5-10	7	24
756	05/04/13	8.1.46	**Porites**	Thin Encrusting	Encrusting	5-10	6	16
757	05/04/13	8.1.47	**Montipora**	Thin Encrusting	Encrusting	10-20	13	44
758	05/04/13	8.1.48	**Pocillopora**	Corymbose	Branching	10-20	19	54
759	05/04/13	8.1.49	**Acropora**	Tabulate	Plating	40-80	44	163
760	05/04/13	8.1.50	**Montipora**	Thin Encrusting	Encrusting	20-40	23	65
761	05/04/13	8.1.51	**Pocillopora**	Corymbose	Branching	10-20	15	40
762	05/04/13	8.1.53	**Porites**	Digitate	Branching	20-40	29	89
763	05/04/13	8.1.54	**Pavona**	Foliose	Upright	20-40	38	104
764	05/04/13	8.1.56	**Favia**	Massive	Massive	10-20	12	34
765	05/04/13	8.1.57	**Cyphastrea**	Columnar	Upright	10-20	10	32
766	05/04/13	8.1.59	**Pavona**	Foliose	Upright	20-40	25	71
767	05/04/13	8.1.64	**Montastrea**	Massive	Massive	10-20	13	35
768	05/04/13	8.1.66	**Acropora**	Tabulate	Plating	40-80	65	200
769	05/04/13	8.2.1	**Porites**	Thin Encrusting	Encrusting	5-10	8	24
770	05/04/13	8.2.3	**Galaxea**	Thin Encrusting	Encrusting	5-10	5	14
771	05/04/13	8.2.6	**Goniastrea**	Submassive	Massive	10-20	12	36
772	05/04/13	8.2.7	**Porites**	Thin Encrusting	Encrusting	10-20	10	28
773	05/04/13	8.2.8	**Acropora**	Tabulate	Plating	80+	84	287
774	05/04/13	8.2.9	**Montipora**	Plating	Plating	10-20	18	48
775	05/04/13	8.2.13	**Acropora**	Branching	Branching			
776	05/04/13	8.2.15	**Montipora**	Plating	Plating	10-20	16	47
777	05/04/13	8.2.18	**Platygyra**	Thick Encrusting	Encrusting	10-20	12	39
778	05/04/13	8.2.24	**Porites**	Digitate	Branching	5-10	7	18
779	05/04/13	8.2.25	**Cyphastrea**	Columnar	Upright	10-20	11	30
780	05/04/13	8.2.26	**Pavona**	Foliose	Upright	5-10	6	21
781	05/04/13	8.2.28	**Porites**	Thin Encrusting	Encrusting	20-40	23	87
782	05/04/13	8.2.29	**Mycedium**	Thin Encrusting	Encrusting	5-10	7	19
783	05/04/13	8.2.30	**Porites**	Branching	Branching	10-20	18	49
784	05/04/13	8.2.31	**Galaxea**	Submassive	Massive	20-40	23	77
785	05/04/13	8.2.32	**Goniastrea**	Submassive	Massive	5-10	7	21
786	05/04/13	8.2.35	**Galaxea**	Thin Encrusting	Encrusting	10-20	14	31
787	05/04/13	8.2.37	**Galaxea**	Thin Encrusting	Encrusting	20-40	22	83
788	05/04/13	8.2.38	**Montipora**	Thin Encrusting	Encrusting	10-20	12	32

789	05/04/13	8.2.39	**Montipora**	Thin Encrusting	Encrusting	10-20	16	50
790	05/04/13	8.2.40	**Montipora**	Thin Encrusting	Encrusting	5-10	9	29
791	05/04/13	8.2.41	**Galaxea**	Thin Encrusting	Encrusting	10-20	12	30
792	05/04/13	8.2.43	**Goniastrea**	Thick Encrusting	Encrusting	10-20	10	27
793	05/04/13	8.2.44	**Porites**	Massive	Massive	20-40	37	121
794	05/04/13	8.2.45	**Cyphastrea**	Columnar	Upright	10-20	11	27
795	05/04/13	8.2.48	**Porites**	Submassive	Massive	10-20	16	52
796	05/04/13	8.2.49	**Platygyra**	Thin Encrusting	Encrusting	10-20	10	25
797	05/04/13	8.2.50	**Montipora**	Thin Encrusting	Encrusting	5-10	8	23
798	05/04/13	8.2.51	**Tubastrea**	Branching	Branching	0-5	4	10
799	05/04/13	8.2.52	**Leptastrea**	Thin Encrusting	Encrusting	5-10	6	18
800	05/04/13	8.2.53	**Favia**	Massive	Massive	5-10	5	15
801	05/04/13	8.2.55	**Porites**	Massive	Massive	20-40	26	76
802	05/04/13	8.2.56	**Porites**	Massive	Massive	10-20	17	46
803	05/04/13	8.2.58	**Cyphastrea**	Thick Encrusting	Encrusting	20-40	32	81
804	05/04/13	8.2.59	**Hydnophora**	Thick Encrusting	Encrusting	5-10	7	21
805	05/04/13	8.2.60	**Platygyra**	Massive	Massive	10-20	19	56
806	05/04/13	8.3.1	**Pavona**	Foliose	Upright	20-40	26	73
807	05/04/13	8.3.2	**Favites**	Thick Encrusting	Encrusting	10-20	19	65
808	05/04/13	8.3.3	**Montastrea**	Submassive	Massive	5-10	8	24
809	05/04/13	8.3.4	**Pavona**	Foliose	Upright	10-20	18	51
810	05/04/13	8.3.5	**Pocillopora**	Corymbose	Branching	10-20	11	34
811	05/04/13	8.3.8	**Montipora**	Thin Encrusting	Encrusting	20-40	34	98
812	05/04/13	8.3.9	**Pocillopora**	Corymbose	Branching	10-20	18	49
813	05/04/13	8.3.10	**Favia**	Massive	Massive	10-20	16	64
814	05/04/13	8.3.11	**Acropora**	Plating	Plating	20-40	22	66
815	05/04/13	8.3.12	**Montipora**	Thin Encrusting	Encrusting	10-20	17	56
816	05/04/13	8.3.14	**Montastrea**	Thick Encrusting	Encrusting	5-10	8	27
817	05/04/13	8.3.15	**Acropora**	Tabulate	Plating	40-80	46	128
818	05/04/13	8.3.16	**Favites**	Thick Encrusting	Encrusting	10-20	12	39
819	05/04/13	8.3.18	**Montipora**	Thin Encrusting	Encrusting	10-20	16	48
820	05/04/13	8.3.19	**Acropora**	Tabulate	Plating	20-40	21	58
821	05/04/13	8.3.20	**Montastrea**	Thick Encrusting	Encrusting	10-20	12	38
822	05/04/13	8.3.22	**Galaxea**	Thin Encrusting	Encrusting	10-20	12	32
823	05/04/13	8.3.23	**Acropora**	Corymbose	Branching	20-40	22	56
824	05/04/13	8.3.24	**Montipora**	Digitate	Branching	20-40	20	59
825	05/04/13	8.3.25	**Montipora**	Digitate	Branching	10-20	12	37
826	05/04/13	8.3.27	**Pocillopora**	Corymbose	Branching	10-20	17	
827	05/04/13	8.3.28	**Montastrea**	Massive	Massive	10-20	16	47
828	05/04/13	8.3.29	**Acropora**	Tabulate	Plating	40-80	55	157
829	05/04/13	8.3.30	**Pocillopora**	Corymbose	Branching	10-20	11	32
830	05/04/13	8.3.33	**Montastrea**	Columnar	Upright	10-20	13	40
831	05/04/13	8.3.34	**Favites**	Thick Encrusting	Encrusting	5-10	5	15
832	05/04/13	8.3.36	**Montipora**	Thin Encrusting	Encrusting	20-40	25	86
833	05/04/13	8.3.39	**Montastrea**	Submassive	Massive	10-20	14	37
834	05/04/13	8.3.40	**Leptastrea**	Thick Encrusting	Encrusting	10-20	18	51
835	05/04/13	8.3.41	**Montipora**	Thin Encrusting	Encrusting	10-20	15	49
836	05/04/13	8.3.44	**Favia**	Massive	Massive	10-20	15	41
837	05/04/13	8.3.45	**Montastrea**	Columnar	Upright	20-40	20	56
838	05/04/13	8.3.47	**Montastrea**	Columnar	Upright	10-20	15	49
839	05/04/13	8.3.48	**Symphyllia**	Thick Encrusting	Encrusting	10-20	18	55
840	05/04/13	8.3.49	**Cyphastrea**	Thick Encrusting	Encrusting	10-20	17	52
841	05/04/13	8.3.50	**Montastrea**	Massive	Massive	10-20	11	30
842	05/04/13	8.3.51	**Montipora**	Thin Encrusting	Encrusting	10-20	13	37
843	05/04/13	8.3.52	**Montastrea**	Thick Encrusting	Encrusting	10-20	15	46
844	05/04/13	8.3.55	**Caulastrea**	Corymbose	Branching	10-20	16	51
845	05/04/13	8.3.56	**Favia**	Massive	Massive	10-20	10	27
846	05/04/13	8.3.57	**Favia**	Massive	Massive	10-20	13	41
847	05/04/13	8.3.59	**Acropora**	Tabulate	Plating	40-80	65	197
848	05/04/13	8.4.1	**Galaxea**	Thick Encrusting	Encrusting	10-20	13	37
849	05/04/13	8.4.3	**Pocillopora**	Corymbose	Branching	5-10	6	16
850	05/04/13	8.4.4	**Favia**	Thick Encrusting	Encrusting	5-10	5	18
851	05/04/13	8.4.7	**Galaxea**	Thick Encrusting	Encrusting	5-10	6	17
852	05/04/13	8.4.10	**Goniastrea**	Submassive	Massive	10-20	11	28
853	05/04/13	8.4.11	**Porites**	Thin Encrusting	Encrusting	5-10	8	28
854	05/04/13	8.4.12	**Montastrea**	Thin Encrusting	Encrusting	10-20	11	32

855	05/04/13	8.4.12.2	**Leptastrea**	Thin Encrusting	Encrusting	5-10	7	19
856	05/04/13	8.4.13	**Galaxea**	Submassive	Massive	5-10	7	21
857	05/04/13	8.4.14	**Porites**	Thin Encrusting	Encrusting	10-20	11	31
858	05/04/13	8.4.15	**Galaxea**	Submassive	Massive	20-40	26	87
859	05/04/13	8.4.16	**Leptastrea**	Thin Encrusting	Encrusting	10-20	12	36
860	05/04/13	8.4.17	**Favites**	Thin Encrusting	Encrusting	5-10	8	25
861	05/04/13	8.4.17.2	**Favia**	Massive	Massive	10-20	11	32
862	05/04/13	8.4.18	**Favia**	Thick Encrusting	Encrusting	5-10	7	22
863	05/04/13	8.4.20	**Montipora**	Digitate	Branching	5-10	5	14
864	05/04/13	8.4.22	**Galaxea**	Thick Encrusting	Encrusting	5-10	6	15
865	05/04/13	8.4.23	**Goniastrea**	Thick Encrusting	Encrusting	10-20	11	40
866	05/04/13	8.4.24	**Porites**	Massive	Massive	80+	140	335
867	05/04/13	8.4.25	**Porites**	Massive	Massive	40-80	55	168
868	05/04/13	8.4.29	**Montastrea**	Submassive	Massive	20-40	27	90
869	05/04/13	8.4.30	**Favites**	Submassive	Massive	10-20	16	45
870	05/04/13	8.4.31	**Favia**	Thick Encrusting	Encrusting	10-20	16	66
871	05/04/13	8.4.32	**Galaxea**	Thin Encrusting	Encrusting	5-10	9	27
872	05/04/13	8.4.33	**Acropora**	Corymbose	Branching	20-40	20	60
873	05/04/13	8.4.34	**Caulastrea**	Corymbose	Branching	80+	113	326
874	05/04/13	8.4.36	**Leptastrea**	Thin Encrusting	Encrusting	10-20	12	38
875	05/04/13	8.4.38	**Galaxea**	Submassive	Massive	10-20	10	27
876	05/04/13	8.4.40	**Montipora**	Thin Encrusting	Encrusting	10-20	13	47
877	05/04/13	8.4.42	**Galaxea**	Thick Encrusting	Encrusting	5-10	8	25
878	05/04/13	8.4.43	**Porites**	Thick Encrusting	Encrusting	20-40	23	68
879	05/04/13	8.4.44	**Mycedium**	Thin Encrusting	Encrusting	10-20	17	54
880	05/04/13	8.4.45	**Galaxea**	Thick Encrusting	Encrusting	20-40	23	87
881	05/04/13	8.4.46	**Favites**	Thick Encrusting	Encrusting	10-20	12	33
882	05/04/13	8.4.46.2	**Galaxea**	Massive	Massive	5-10	7	19
883	05/04/13	8.4.48	**Porites**	Thin Encrusting	Encrusting	5-10	7	29
884	05/04/13	8.4.54	**Montastrea**	Thick Encrusting	Encrusting	10-20	12	38
885	05/04/13	8.4.55	**Galaxea**	Thick Encrusting	Encrusting	10-20	18	56
886	05/04/13	8.4.56	**Goniastrea**	Thick Encrusting	Encrusting	5-10	9	28
887	05/04/13	8.4.57	**Porites**	Massive	Massive	80+	88	258
888	05/04/13	8.4.58	**Caulastrea**	Corymbose	Branching	20-40	38	140
889	05/04/13	8.4.59	**Galaxea**	Thin Encrusting	Encrusting	5-10	6	19
890	05/04/13	8.4.60	**Porites**	Digitate	Branching	5-10	9	29
891	05/04/13	8.4.61	**Porites**	Digitate	Branching	10-20	11	30
892	05/04/13	8.4.63	**Favites**	Massive	Massive	10-20	11	34
893	05/04/13	8.4.64	**Goniastrea**	Massive	Massive	10-20	10	30
894	05/04/13	8.4.66	**Montastrea**	Columnar	Upright	10-20	16	50
895	05/04/13	8.5.1	**Acropora**	Tabulate	Plating	80+	140	394
896	05/04/13	8.5.2	**Hydnophora**	Digitate	Branching	5-10	7	19
897	05/04/13	8.5.3	**Montastrea**	Thick Encrusting	Encrusting	10-20	12	35
898	05/04/13	8.5.4	**Favites**	Thin Encrusting	Encrusting	10-20	16	51
899	05/04/13	8.5.5	**Goniastrea**	Thin Encrusting	Encrusting	5-10	8	32
900	05/04/13	8.5.6	**Pavona**	Foliose	Upright	10-20	10	30
901	05/04/13	8.5.7	**Favites**	Submassive	Massive	5-10	9	26
902	05/04/13	8.5.8	**Favites**	Thick Encrusting	Encrusting	20-40	29	127
903	05/04/13	8.5.9	**Galaxea**	Thin Encrusting	Encrusting	5-10	5	12
904	05/04/13	8.5.11	**Montastrea**	Submassive	Massive	5-10	6	18
905	05/04/13	8.5.12	**Porites**	Thin Encrusting	Encrusting	5-10	7	21
906	05/04/13	8.5.13	**Montipora**	Thin Encrusting	Encrusting	10-20	13	37
907	05/04/13	8.5.14	**Galaxea**	Thick Encrusting	Encrusting	5-10	8	22
908	05/04/13	8.5.15	**Galaxea**	Thick Encrusting	Encrusting	5-10	7	19
909	05/04/13	8.5.17	**Pocillopora**	Corymbose	Branching	20-40	28	82
910	05/04/13	8.5.18	**Porites**	Thin Encrusting	Encrusting	20-40	29	88
911	05/04/13	8.5.19	**Favites**	Massive	Massive	10-20	16	56
912	05/04/13	8.6.1	**Pavona**	Foliose	Upright	10-20	10	38
913	05/04/13	8.6.2	**Goniastrea**	Submassive	Massive	5-10	9	37
914	05/04/13	8.6.6	**Leptastrea**	Thin Encrusting	Encrusting	5-10	7	32
915	05/04/13	8.6.7	**Leptastrea**	Thin Encrusting	Encrusting	5-10	8	24
916	05/04/13	8.6.8	**Caulastrea**	Corymbose	Branching	10-20	13	35
917	05/04/13	8.6.10	**Porites**	Massive	Massive	10-20	17	49
918	05/04/13	8.6.13	**Platygyra**	Massive	Massive	5-10	6	15
919	05/04/13	8.6.15	**Porites**	Thin Encrusting	Encrusting	5-10	6	21
920	05/04/13	8.6.16	**Galaxea**	Thin Encrusting	Encrusting	5-10	9	22

921	05/04/13	8.6.17	**Caulastrea**	Corymbose	Branching	5-10	6	20
922	05/04/13	8.6.21	**Porites**	Thin Encrusting	Encrusting	10-20	15	36
923	05/04/13	8.6.24	**Montipora**	Digitate	Branching	5-10	6	22
924	05/04/13	8.6.26	**Montipora**	Thin Encrusting	Encrusting	10-20	13	39
925	05/04/13	8.6.27	**Acropora**	Branching	Branching			
926	05/04/13	8.6.28	**Mycedium**	Thin Encrusting	Encrusting	20-40	27	100
927	05/04/13	8.6.30	**Hydnophora**	Thin Encrusting	Encrusting	10-20	16	45
928	05/04/13	8.6.33	**Montipora**	Thin Encrusting	Encrusting	10-20	18	60
929	05/04/13	8.6.34	**Montipora**	Thin Encrusting	Encrusting	5-10	7	27
930	05/04/13	8.6.35	**Lobophyllia**	Thick Encrusting	Encrusting	0-5	4	11
931	05/04/13	8.6.36	**Montastrea**	Thick Encrusting	Encrusting	5-10	7	20
932	05/04/13	8.6.37	**Leptastrea**	Thick Encrusting	Encrusting	10-20	10	29
933	05/04/13	8.6.38	**Montipora**	Thin Encrusting	Encrusting	10-20	13	38
934	05/04/13	8.6.40	**Leptastrea**	Thin Encrusting	Encrusting	5-10	7	22
935	05/04/13	8.6.41	**Galaxea**	Thick Encrusting	Encrusting	5-10	9	24
936	05/04/13	8.6.42	**Pavona**	Submassive	Massive	5-10	6	17
937	05/04/13	8.6.43	**Cyphastrea**	Thin Encrusting	Encrusting	5-10	6	16
938	05/04/13	8.6.46	**Favites**	Thick Encrusting	Encrusting	5-10	8	24
939	05/04/13	8.6.47	**Favites**	Thick Encrusting	Encrusting	5-10	8	26
940	05/04/13	8.6.49	**Favites**	Thick Encrusting	Encrusting	5-10	7	26
941	05/04/13	8.6.50	**Porites**	Thin Encrusting	Encrusting	10-20	12	39
942	05/04/13	8.6.51	**Astreopora**	Thin Encrusting	Encrusting	5-10	7	17
943	05/04/13	8.6.52	**Porites**	Branching	Branching			
944	05/04/13	8.6.53	**Galaxea**	Thin Encrusting	Encrusting	5-10	7	22
945	05/04/13	8.6.54	**Leptoria**	Thick Encrusting	Encrusting	5-10	9	27
946	05/04/13	8.6.57	**Porites**	Thin Encrusting	Encrusting	5-10	8	21
947	05/04/13	8.6.60	**Ctenactis**	Solitary	Solitary	5-10	8	19
948	05/04/13	8.6.61	**Leptastrea**	Thin Encrusting	Encrusting	5-10	7	23
949	05/04/13	8.6.62	**Cyphastrea**	Columnar	Upright	5-10	5	16
950	05/04/13	8.6.63	**Porites**	Branching	Branching			
951	05/04/13	8.6.65	**Porites**	Branching	Branching	10-20	16	43
952	05/04/13	8.6.66	**Mycedium**	Thin Encrusting	Encrusting	10-20	16	50
953	05/04/13	8.6.68	**Pavona**	Foliose	Upright	10-20	15	38
954	05/04/13	8.6.69	**Leptastrea**	Thin Encrusting	Encrusting	5-10	6	19
955	05/04/13	8.6.70	**Leptastrea**	Thick Encrusting	Encrusting	5-10	9	37
956	05/04/13	8.6.72	**Porites**	Thin Encrusting	Encrusting	5-10	9	23
957	05/04/13	8.6.73	**Porites**	Thick Encrusting	Encrusting	5-10	6	17
958	05/04/13	8.6.74	**Leptastrea**	Thick Encrusting	Encrusting	5-10	6	19
959	05/04/13	8.6.75	**Montastrea**	Thick Encrusting	Encrusting	5-10	6	19
960	05/04/13	8.6.76	**Favites**	Thick Encrusting	Encrusting	10-20	10	29
961	05/04/13	8.7.1	**Astreopora**	Thick Encrusting	Encrusting	5-10	5	15
962	05/04/13	8.7.4	**Mycedium**	Thin Encrusting	Encrusting	20-40	26	92
963	05/04/13	8.7.8	**Acropora**	Tabulate	Plating	40-80	68	208
964	05/04/13	8.8.2	**Montastrea**	Submassive	Massive	5-10	6	17
965	05/04/13	8.8.3	**Porites**	Digitate	Branching			
966	05/04/13	8.8.5	**Montipora**	Digitate	Branching			
967	05/04/13	8.8.10	**Favites**	Submassive	Massive	5-10	5	15
968	05/04/13	8.8.12	**Favites**	Massive	Massive	10-20	16	49
969	05/04/13	8.8.13	**Porites**	Digitate	Branching			
970	05/04/13	8.8.14	**Porites**	Digitate	Branching			
971	05/04/13	8.8.16	**Porites**	Digitate	Branching			
972	05/04/13	8.8.17	**Porites**	Digitate	Branching			
973	05/04/13	8.8.18	**Favites**	Massive	Massive	10-20	19	55
974	05/04/13	8.8.20	**Favites**	Massive	Massive	5-10	9	25
975	05/04/13	8.8.21	**Favia**	Massive	Massive	10-20	18	53
976	05/04/13	8.8.22	**Galaxea**	Thick Encrusting	Encrusting	5-10	8	26
977	05/04/13	8.8.23	**Favia**	Massive	Massive	20-40	23	83
978	05/04/13	8.8.25	**Pocillopora**	Corymbose	Branching	5-10	7	21
979	05/04/13	8.8.27	**Favites**	Submassive	Massive	20-40	30	101
980	05/04/13	8.8.29	**Goniastrea**	Massive	Massive	10-20	14	38
981	05/04/13	8.8.30	**Pocillopora**	Corymbose	Branching	20-40	23	81
982	05/04/13	8.8.31	**Porites**	Thin Encrusting	Encrusting	10-20	12	47
983	05/04/13	8.8.32	**Favites**	Thick Encrusting	Encrusting	5-10	8	27
984	05/04/13	8.8.35	**Pocillopora**	Corymbose	Branching	20-40	39	132
985	05/04/13	8.8.36	**Favia**	Submassive	Massive	5-10	5	18
986	05/04/13	8.8.37	**Favia**	Submassive	Massive	10-20	13	38

987	05/04/13	8.8.38	**Leptastrea**	Thick Encrusting	Encrusting	5-10	9	23
988	05/04/13	8.8.39	**Pocillopora**	Corymbose	Branching	10-20	15	45
989	05/04/13	8.8.40	**Montastrea**	Massive	Massive	10-20	17	48
990	05/04/13	8.9.2	**Porites**	Digitate	Branching	5-10	8	
991	05/04/13	8.9.3	**Porites**	Columnar	Upright	20-40	22	88
992	05/04/13	8.9.5	**Porites**	Columnar	Upright	10-20	19	62
993	05/04/13	8.9.7	**Porites**	Digitate	Branching			
994	05/04/13	8.9.8	**Porites**	Massive	Massive	10-20	18	62
995	05/04/13	8.9.9	**Porites**	Digitate	Branching			
996	05/04/13	8.9.9.2	**Porites**	Digitate	Branching			
997	05/04/13	8.9.10	**Porites**	Digitate	Branching			
998	05/04/13	8.9.11	**Porites**	Digitate	Branching			
999	05/04/13	8.9.12	**Favites**	Massive	Massive	20-40	20	61
1000	05/04/13	8.9.14	**Porites**	Digitate	Branching			
1001	05/04/13	8.9.16	**Porites**	Digitate	Branching			
1002	05/04/13	8.9.17	**Leptastrea**	Thin Encrusting	Encrusting	5-10	6	19
1003	05/04/13	8.9.19	**Montipora**	Digitate	Branching			
1004	05/04/13	8.9.20	**Porites**	Digitate	Branching			
1005	05/04/13	8.9.21	**Porites**	Digitate	Branching			
1006	05/04/13	8.9.22	**Favia**	Thin Encrusting	Encrusting	5-10	5	15
1007	05/04/13	8.9.24	**Porites**	Digitate	Branching			
1008	05/04/13	8.9.25	**Porites**	Digitate	Branching			
1009	05/04/13	8.9.26	**Favia**	Submassive	Massive	5-10	5	16
1010	05/04/13	8.9.27	**Favia**	Submassive	Massive	10-20	11	31
1011	05/04/13	8.9.28	**Porites**	Digitate	Branching			
1012	05/04/13	8.9.29	**Porites**	Digitate	Branching			
1013	05/04/13	8.9.30	**Symphyllia**	Thick Encrusting	Encrusting	10-20	14	43
1014	05/04/13	8.9.31	**Porites**	Digitate	Branching			
1015	05/04/13	8.9.32	**Porites**	Digitate	Branching			
1016	05/04/13	8.9.33	**Favites**	Thick Encrusting	Encrusting	10-20	10	28
1017	05/04/13	8.9.34	**Porites**	Digitate	Branching			
1018	05/04/13	8.9.35	**Porites**	Digitate	Branching			
1019	05/04/13	8.9.36	**Porites**	Digitate	Branching			
1020	05/04/13	8.9.37	**Porites**	Digitate	Branching			
1021	05/04/13	8.9.38	**Porites**	Digitate	Branching			
1022	05/04/13	8.9.39	**Favia**	Submassive	Massive	5-10	9	21
1023	05/04/13	8.9.42	**Porites**	Digitate	Branching			
1024	05/04/13	8.9.43	**Favia**	Massive	Massive	10-20	14	35
1025	05/04/13	8.9.44	**Favia**	Submassive	Massive	5-10	5	13
1026	05/04/13	8.9.45	**Leptastrea**	Thin Encrusting	Encrusting	10-20	14	38
1027	05/04/13	8.9.47	**Montipora**	Digitate	Branching			
1028	05/04/13	8.9.50	**Favia**	Thick Encrusting	Encrusting	5-10	7	25
1029	05/04/13	8.9.51	**Montastrea**	Thick Encrusting	Encrusting	10-20	15	55
1030	05/04/13	8.9.52	**Favia**	Submassive	Massive	5-10	7	19
1031	05/04/13	8.9.53	**Favia**	Thick Encrusting	Encrusting	10-20	12	41
1032	05/04/13	8.9.55	**Porites**	Digitate	Branching			
1033	05/04/13	8.9.56	**Platygyra**	Thick Encrusting	Encrusting	5-10	6	19
1034	05/04/13	8.9.58	**Favites**	Thick Encrusting	Encrusting	5-10	8	25
1035	05/04/13	8.9.60	**Porites**	Massive	Massive	80+	150	330
1036	05/04/13	8.9.63	**Favites**	Thick Encrusting	Encrusting	5-10	6	17
1037	05/04/13	8.9.64	**Favites**	Thin Encrusting	Encrusting	5-10	8	25
1038	05/04/13	8.9.65	**Favites**	Submassive	Massive	10-20	10	42
1039	05/04/13	8.9.66	**Favia**	Massive	Massive	10-20	13	38
1040	05/04/13	8.9.67	**Montastrea**	Submassive	Massive	10-20	18	74
1041	05/04/13	8.9.68	**Platygyra**	Submassive	Massive	5-10	5	14
1042	05/04/13	8.9.69	**Porites**	Thin Encrusting	Encrusting	10-20	12	35
1043	05/04/13	8.9.73	**Porites**	Digitate	Branching	40-80	45	
1044	05/04/13	8.9.75	**Goniopora**	Submassive	Massive	20-40	24	68
1045	05/04/13	8.9.78	**Porites**	Digitate	Branching			
1046	05/04/13	8.9.79	**Porites**	Digitate	Branching			
1047	05/04/13	8.9.80	**Pocillopora**	Corymbose	Branching	5-10	6	16
1048	05/04/13	8.9.81	**Favites**	Massive	Massive	10-20	16	51
1049	05/04/13	8.10.1	**Acropora**	Branching	Branching			
1050	05/04/13	8.10.2	**Acropora**	Branching	Branching			
1051	05/04/13	8.10.3	**Acropora**	Branching	Branching			
1052	05/04/13	8.10.4	**Acropora**	Branching	Branching			

1053	05/04/13	8.10.5	**Acropora**	Branching	Branching					
1054	05/04/13	8.10.6	**Acropora**	Branching	Branching					
1055	05/04/13	8.10.7	**Acropora**	Branching	Branching					
1056	05/04/13	8.10.8	**Acropora**	Branching	Branching					
1057	05/04/13	8.10.9	**Acropora**	Branching	Branching					
1058	05/04/13	8.10.10	**Acropora**	Branching	Branching					
1059	05/04/13	8.10.11	**Acropora**	Branching	Branching					
1060	05/04/13	8.10.12	**Acropora**	Branching	Branching					
1061	05/04/13	8.10.13	**Acropora**	Branching	Branching					
1062	05/04/13	8.10.14	**Acropora**	Branching	Branching					
1063	05/04/13	8.10.15	**Acropora**	Branching	Branching					
1064	05/04/13	8.10.16	**Acropora**	Branching	Branching					
1065	05/04/13	8.10.17	**Acropora**	Branching	Branching					
1066	05/04/13	8.10.18	**Acropora**	Branching	Branching					
1067	05/04/13	8.10.19	**Acropora**	Branching	Branching					
1068	05/04/13	8.10.20	**Acropora**	Branching	Branching					
1069	05/04/13	8.10.21	**Acropora**	Branching	Branching					
1070	05/04/13	8.10.22	**Acropora**	Branching	Branching					
1071	05/04/13	8.10.23	**Acropora**	Branching	Branching					
1072	05/04/13	8.10.24	**Acropora**	Branching	Branching					
1073	05/04/13	8.10.25	**Acropora**	Branching	Branching					
1074	05/04/13	8.10.26	**Acropora**	Branching	Branching					
1075	05/04/13	8.10.27	**Acropora**	Branching	Branching					
1076	05/04/13	8.10.28	**Acropora**	Branching	Branching					
1077	05/04/13	8.10.29	**Acropora**	Branching	Branching					
1078	05/04/13	8.10.30	**Acropora**	Branching	Branching					
1079	05/04/13	8.10.31	**Acropora**	Branching	Branching					
1080	05/04/13	8.10.32	**Acropora**	Branching	Branching					
1081	05/04/13	8.10.33	**Acropora**	Branching	Branching					
1082	05/04/13	8.10.34	**Acropora**	Branching	Branching					
1083	05/04/13	8.10.35	**Acropora**	Branching	Branching					
1084	05/04/13	8.10.36	**Acropora**	Branching	Branching					
1085	05/04/13	8.10.37	**Acropora**	Branching	Branching					
1086	05/04/13	8.10.38	**Acropora**	Branching	Branching					
1087	05/04/13	8.10.39	**Acropora**	Branching	Branching					
1088	05/05/13	9.1.1	**Podabacia**	Thin Encrusting	Encrusting	5-10	8		28	
1089	05/05/13	9.1.2	**Pavona**	Foliose	Upright	10-20	11		31	
1090	05/05/13	9.1.3	**Acropora**	Tabulate	Plating	40-80	52		162	
1091	05/05/13	9.1.4	**Montipora**	Thin Encrusting	Encrusting	20-40	25		74	
1092	05/05/13	9.1.6	**Montastrea**	Thin Encrusting	Encrusting	5-10	9		34	
1093	05/05/13	9.1.7	**Pavona**	Foliose	Upright	10-20	11		35	
1094	05/05/13	9.1.8	**Montipora**	Thin Encrusting	Encrusting	10-20	17		50	
1095	05/05/13	9.1.9	**Acropora**	Tabulate	Plating	80+	82		227	
1096	05/05/13	9.1.11	**Acropora**	Tabulate	Plating	20-40	39		117	
1097	05/05/13	9.1.12	**Acropora**	Tabulate	Plating	20-40	32		95	
1098	05/05/13	9.1.13	**Acropora**	Tabulate	Plating	40-80	52		160	
1099	05/05/13	9.1.14	**Acropora**	Tabulate	Plating	40-80	75		200	
1100	05/05/13	9.1.15	**Pocillopora**	Corymbose	Branching	5-10	8		32	
1101	05/05/13	9.1.16	**Acropora**	Tabulate	Plating	40-80	40		105	
1102	05/05/13	9.1.17	**Pocillopora**	Corymbose	Branching	20-40	25		73	
1103	05/05/13	9.1.18	**Porites**	Thin Encrusting	Encrusting	20-40	24		72	
1104	05/05/13	9.1.19	**Platygyra**	Submassive	Massive	20-40	22		63	
1105	05/05/13	9.1.20	**Porites**	Thin Encrusting	Encrusting	20-40	31		112	
1106	05/05/13	9.1.23	**Galaxea**	Thin Encrusting	Encrusting	5-10	8		24	
1107	05/05/13	9.1.24	**Favia**	Massive	Massive	5-10	8		24	
1108	05/05/13	9.1.25	**Porites**	Digitate	Branching					
1109	05/05/13	9.1.26	**Montastrea**	Thin Encrusting	Encrusting	10-20	13		42	
1110	05/05/13	9.1.27	**Pavona**	Foliose	Upright	20-40	24		96	
1111	05/05/13	9.1.28	**Acropora**	Tabulate	Plating	80+	84		242	
1112	05/05/13	9.1.30	**Porites**	Branching	Branching					
1113	05/05/13	9.1.31	**Pavona**	Foliose	Upright	5-10	9		26	
1114	05/05/13	9.1.32	**Porites**	Digitate	Branching					
1115	05/05/13	9.1.33	**Porites**	Digitate	Branching					
1116	05/05/13	9.1.34	**Goniastrea**	Thick Encrusting	Encrusting	5-10	9		27	
1117	05/05/13	9.1.36	**Porites**	Thin Encrusting	Encrusting	40-80	43		16	
1118	05/05/13	9.1.37	**Goniastrea**	Massive	Massive	10-20	17		46	

1119	05/05/13	9.1.38	**Favia**	Submassive	Massive	10-20	10	27
1120	05/05/13	9.1.39	**Goniastrea**	Massive	Massive	5-10	6	17
1121	05/05/13	9.1.40	**Pocillopora**	Corymbose	Branching	10-20	17	49
1122	05/05/13	9.1.41	**Montastrea**	Thick Encrusting	Encrusting	5-10	7	19
1123	05/05/13	9.1.43	**Porites**	Thin Encrusting	Encrusting	10-20	18	57
1124	05/05/13	9.1.44	**Porites**	Digitate	Branching			
1125	05/05/13	9.1.48	**Montipora**	Thin Encrusting	Encrusting	20-40	30	86
1126	05/05/13	9.1.49	**Pocillopora**	Corymbose	Branching	10-20	15	40
1127	05/05/13	9.1.50	**Porites**	Digitate	Branching			
1128	05/05/13	9.1.51	**Acropora**	Tabulate	Plating	20-40	20	60
1129	05/05/13	9.1.52	**Pocillopora**	Corymbose	Branching	10-20	11	28
1130	05/05/13	9.1.54	**Porites**	Digitate	Branching			
1131	05/05/13	9.1.55	**Acropora**	Tabulate	Plating	40-80	50	129
1132	05/05/13	9.1.56	**Porites**	Digitate	Branching			
1133	05/05/13	9.1.57	**Porites**	Digitate	Branching			
1134	05/05/13	9.1.58	**Montastrea**	Columnar	Upright	20-40	30	81
1135	05/05/13	9.2.1	**Favia**	Massive	Massive	5-10	9	30
1136	05/05/13	9.2.2	**Acropora**	Tabulate	Plating	80+	85	282
1137	05/05/13	9.2.2.2	**Acropora**	Tabulate	Plating	40-80	51	195
1138	05/05/13	9.2.3	**Montastrea**	Columnar	Upright	10-20	11	32
1139	05/05/13	9.2.4	**Acropora**	Tabulate	Plating	40-80	54	145
1140	05/05/13	9.2.5	**Ctenactis**	Solitary	Solitary	10-20	15	38
1141	05/05/13	9.2.6	**Acropora**	Tabulate	Plating	80+	91	391
1142	05/05/13	9.2.7	**Montastrea**	Thick Encrusting	Encrusting	5-10	9	33
1143	05/05/13	9.2.8	**Montipora**	Digitate	Branching			
1144	05/05/13	9.2.9	**Montipora**	Digitate	Branching			
1145	05/05/13	9.2.10	**Montipora**	Digitate	Branching			
1146	05/05/13	9.2.12	**Platygyra**	Submassive	Massive	10-20	17	56
1147	05/05/13	9.2.14	**Platygyra**	Submassive	Massive	10-20	13	41
1148	05/05/13	9.2.15	**Goniastrea**	Massive	Massive	5-10	6	16
1149	05/05/13	9.2.16	**Favia**	Massive	Massive	5-10	7	18
1150	05/05/13	9.2.17	**Porites**	Digitate	Branching			
1151	05/05/13	9.2.18	**Galaxea**	Thin Encrusting	Encrusting	5-10	5	19
1152	05/05/13	9.2.19	**Galaxea**	Thin Encrusting	Encrusting	5-10	6	16
1153	05/05/13	9.2.20	**Cyphastrea**	Submassive	Massive	5-10	8	22
1154	05/05/13	9.2.21	**Pavona**	Foliose	Upright	10-20	19	51
1155	05/05/13	9.2.22	**Caulastrea**	Corymbose	Branching	40-80	40	155
1156	05/05/13	9.2.23	**Acropora**	Tabulate	Plating	40-80	58	218
1157	05/05/13	9.2.24	**Acropora**	Tabulate	Plating	40-80	57	161
1158	05/05/13	9.2.25	**Acropora**	Tabulate	Plating	40-80	62	199
1159	05/05/13	9.2.27	**Pocillopora**	Corymbose	Branching	20-40	25	70
1160	05/05/13	9.2.28	**Pavona**	Foliose	Upright	20-40	29	83
1161	05/05/13	9.2.33	**Pavona**	Foliose	Upright	10-20	15	45
1162	05/05/13	9.2.34	**Pocillopora**	Corymbose	Branching	10-20	13	40
1163	05/05/13	9.2.36	**Pavona**	Foliose	Upright	10-20	12	36
1164	05/05/13	9.2.37	**Pavona**	Foliose	Upright	10-20	14	41
1165	05/05/13	9.2.40	**Caulastrea**	Corymbose	Branching	5-10	9	25
1166	05/05/13	9.2.41	**Porites**	Thin Encrusting	Encrusting	20-40	20	62
1167	05/05/13	9.2.45	**Caulastrea**	Corymbose	Branching	5-10	7	19
1168	05/05/13	9.2.46	**Leptastrea**	Thin Encrusting	Encrusting	10-20	18	66
1169	05/05/13	9.2.49	**Pocillopora**	Corymbose	Branching	20-40	34	109
1170	05/05/13	9.2.50	**Montastrea**	Massive	Massive	20-40	20	51
1171	05/05/13	9.2.52	**Favia**	Massive	Massive	10-20	17	50
1172	05/05/13	9.2.53	**Acropora**	Tabulate	Plating	40-80	66	188
1173	05/05/13	9.2.54	**Caulastrea**	Corymbose	Branching	10-20	13	39
1174	05/05/13	9.2.55	**Montipora**	Digitate	Branching			
1175	05/05/13	9.2.56	**Favia**	Submassive	Massive	5-10	8	26
1176	05/05/13	9.2.57	**Acropora**	Branching	Branching			
1177	05/05/13	9.2.58	**Porites**	Digitate	Branching			
1178	05/05/13	9.3.1	**Acropora**	Branching	Branching			
1179	05/05/13	9.3.2	**Porites**	Digitate	Branching			
1180	05/05/13	9.3.6	**Montipora**	Thin Encrusting	Encrusting	20-40	25	72
1181	05/05/13	9.3.8	**Acropora**	Tabulate	Plating	40-80	62	198
1182	05/05/13	9.3.9	**Fungia**	Solitary	Solitary	5-10	9	27
1183	05/05/13	9.3.10	**Acropora**	Branching	Branching	5-10	6	
1184	05/05/13	9.3.11	**Porites**	Thin Encrusting	Encrusting	5-10	5	14

1185	05/05/13	9.3.12	**Porites**	Thin Encrusting	Encrusting	5-10	7	22
1186	05/05/13	9.3.13	**Acropora**	Branching	Branching			
1187	05/05/13	9.3.14	**Porites**	Branching	Branching			
1188	05/05/13	9.3.15	**Porites**	Thin Encrusting	Encrusting	20-40	33	95
1189	05/05/13	9.3.16	**Galaxea**	Thick Encrusting	Encrusting	10-20	13	39
1190	05/05/13	9.3.18	**Acropora**	Branching	Branching			
1191	05/05/13	9.3.19	**Acropora**	Corymbose	Branching			
1192	05/05/13	9.3.20	**Montastrea**	Columnar	Upright	10-20	16	48
1193	05/05/13	9.3.21	**Acropora**	Tabulate	Plating	40-80	62	203
1194	05/05/13	9.3.22	**Montipora**	Digitate	Branching			
1195	05/05/13	9.3.24	**Fungia**	Solitary	Solitary	10-20	13	39
1196	05/05/13	9.3.25	**Fungia**	Solitary	Solitary	10-20	13	42
1197	05/05/13	9.3.26	**Acropora**	Tabulate	Plating	40-80	59	236
1198	05/05/13	9.3.27	**Pocillopora**	Corymbose	Branching	5-10	9	28
1199	05/05/13	9.3.29	**Acropora**	Tabulate	Plating	40-80	72	249
1200	05/05/13	9.3.30	**Acropora**	Tabulate	Plating	40-80	49	139
1201	05/05/13	9.3.31	**Favia**	Massive	Massive	10-20	13	39
1202	05/05/13	9.3.32	**Platygyra**	Submassive	Massive	20-40	21	59
1203	05/05/13	9.3.33	**Fungia**	Solitary	Solitary	10-20	15	47
1204	05/05/13	9.3.35	**Montipora**	Thin Encrusting	Encrusting	20-40	25	73
1205	05/05/13	9.3.36	**Acropora**	Tabulate	Plating	20-40	39	113
1206	05/05/13	9.3.37	**Acropora**	Tabulate	Plating	40-80	69	258
1207	05/05/13	9.3.38	**Acropora**	Tabulate	Plating	80+	84	255
1208	05/05/13	9.3.39	**Acropora**	Tabulate	Plating	40-80	43	114
1209	05/05/13	9.3.40	**Montipora**	Digitate	Branching			
1210	05/05/13	9.3.41	**Pocillopora**	Corymbose	Branching	10-20	10	30
1211	05/05/13	9.3.42	**Acropora**	Tabulate	Plating	40-80	55	171
1212	05/05/13	9.3.44	**Acropora**	Tabulate	Plating	80+	89	250
1213	05/05/13	9.3.45	**Pavona**	Foliose	Upright	5-10	7	19
1214	05/05/13	9.3.46	**Montipora**	Digitate	Branching			
1215	05/05/13	9.3.47	**Pavona**	Foliose	Upright	5-10	9	28
1216	05/05/13	9.3.48	**Acropora**	Tabulate	Plating	20-40	21	60
1217	05/05/13	9.3.49	**Acropora**	Tabulate	Plating	20-40	37	111
1218	05/05/13	9.3.50	**Acropora**	Corymbose	Branching			
1219	05/05/13	9.3.51	**Acropora**	Tabulate	Plating	20-40	23	72
1220	05/05/13	9.3.52	**Acropora**	Corymbose	Branching			
1221	05/05/13	9.3.53	**Acropora**	Tabulate	Plating	40-80	49	141
1222	05/05/13	9.3.54	**Acropora**	Tabulate	Plating	20-40	20	60
1223	05/05/13	9.3.55	**Acropora**	Tabulate	Plating	20-40	24	70
1224	05/05/13	9.3.56	**Acropora**	Tabulate	Plating	40-80	44	118
1225	05/05/13	9.3.57	**Acropora**	Tabulate	Plating	20-40	24	73
1226	05/05/13	9.3.58	**Montipora**	Plating	Plating	5-10	9	28
1227	05/05/13	9.3.59	**Acropora**	Tabulate	Plating	80+	90	257
1228	05/05/13	9.3.60	**Montipora**	Digitate	Branching			
1229	05/05/13	9.3.61	**Pavona**	Foliose	Upright	5-10	6	19
1230	05/05/13	9.3.62	**Porites**	Thin Encrusting	Encrusting	10-20	11	35
1231	05/05/13	9.3.63	**Porites**	Digitate	Branching			
1232	05/05/13	9.3.64	**Acropora**	Tabulate	Plating	40-80	45	162
1233	05/05/13	9.3.65	**Acropora**	Tabulate	Plating	40-80	40	124
1234	05/05/13	9.3.66	**Montipora**	Plating	Plating	10-20	16	48
1235	05/05/13	9.3.67	**Montipora**	Thin Encrusting	Encrusting	40-80	45	150
1236	05/05/13	9.3.68	**Galaxea**	Thick Encrusting	Encrusting	5-10	8	27
1237	05/05/13	9.3.70	**Acropora**	Corymbose	Branching			
1238	05/05/13	9.3.71	**Ctenactis**	Solitary	Solitary	5-10	7	17
1239	05/05/13	9.3.72	**Leptastrea**	Submassive	Massive	10-20	11	29
1240	05/05/13	9.4.1	**Fungia**	Solitary	Solitary	10-20	14	43
1241	05/05/13	9.4.2	**Ctenactis**	Solitary	Solitary	5-10	7	18
1242	05/05/13	9.4.3	**Astreopora**	Thin Encrusting	Encrusting	10-20	14	41
1243	05/05/13	9.4.4	**Acropora**	Tabulate	Plating	20-40	33	94
1244	05/05/13	9.4.5	**Galaxea**	Thin Encrusting	Encrusting	5-10	8	23
1245	05/05/13	9.4.6	**Pocillopora**	Corymbose	Branching	10-20	11	31
1246	05/05/13	9.4.7	**Pocillopora**	Corymbose	Branching	10-20	11	33
1247	05/05/13	9.4.8	**Fungia**	Solitary	Solitary	10-20	11	36
1248	05/05/13	9.4.9	**Fungia**	Solitary	Solitary	5-10	7	22
1249	05/05/13	9.4.10	**Porites**	Thin Encrusting	Encrusting	20-40	34	93
1250	05/05/13	9.4.11	**Montipora**	Thin Encrusting	Encrusting	20-40	25	73

1251	05/05/13	9.4.12	**Platygyra**	Thick Encrusting	Encrusting	20-40	24	86
1252	05/05/13	9.4.13	**Pavona**	Foliose	Upright	10-20	15	49
1253	05/05/13	9.4.14	**Pavona**	Foliose	Upright	10-20	17	50
1254	05/05/13	9.4.15	**Caulastrea**	Corymbose	Branching	20-40	20	55
1255	05/05/13	9.4.16	**Favites**	Submassive	Massive	20-40	21	66
1256	05/05/13	9.4.17	**Caulastrea**	Corymbose	Branching	10-20	15	46
1257	05/05/13	9.4.18	**Porites**	Thin Encrusting	Encrusting	20-40	36	110
1258	05/05/13	9.4.19	**Montipora**	Thin Encrusting	Encrusting	10-20	15	46
1259	05/05/13	9.4.20	**Favia**	Massive	Massive	10-20	15	47
1260	05/05/13	9.4.21	**Acropora**	Tabulate	Plating	80+	109	301
1261	05/05/13	9.4.23	**Pavona**	Foliose	Upright	10-20	11	39
1262	05/05/13	9.4.24	**Favia**	Thick Encrusting	Encrusting	5-10	5	15
1263	05/05/13	9.4.25	**Leptastrea**	Thick Encrusting	Encrusting	10-20	11	28
1264	05/05/13	9.4.26	**Porites**	Massive	Massive	10-20	10	29
1265	05/05/13	9.4.28	**Montipora**	Thin Encrusting	Encrusting	10-20	18	58
1266	05/05/13	9.4.30	**Acropora**	Tabulate	Plating	40-80	46	143
1267	05/05/13	9.4.31	**Porites**	Massive	Massive	10-20	11	35
1268	05/05/13	9.4.32	**Favia**	Massive	Massive	10-20	14	43
1269	05/05/13	9.4.34	**Porites**	Columnar	Upright	10-20	12	32
1270	05/05/13	9.4.35	**Porites**	Columnar	Upright	10-20	16	52
1271	05/05/13	9.4.36	**Porites**	Columnar	Upright	10-20	13	45
1272	05/05/13	9.4.37	**Favia**	Submassive	Massive	10-20	16	47
1273	05/05/13	9.4.41	**Pavona**	Foliose	Upright	10-20	17	48
1274	05/05/13	9.4.42	**Favia**	Massive	Massive	10-20	17	49
1275	05/05/13	9.4.44	**Favia**	Massive	Massive	10-20	10	33
1276	05/05/13	9.4.45	**Galaxea**	Submassive	Massive	5-10	5	14

# of branches (Acropora Branching)	% f border interacting with algae	Length of border interacting with algae	Turf % of interaction	Total length in interaction with Turf	*% Algae win*	*in cm*	*% Neutral*
	26%	57,2	100%	57,20	*0%*	*0*	*0%*
100+	45%	0	82%	-	*70%*	*0*	*30%*
	20%	10	100%	10,00	*100%*	*10*	*0%*
	40%	16	100%	16,00	*85%*	*13,6*	*15%*
65	10%	0	0%	-	*0%*		*0%*
	0%	0	0%	-	*0%*		*0%*
	8%	3,6	100%	3,60	*25%*	*0,9*	*75%*
	70%	22,4	20%	4,48	*100%*	*4,48*	*0%*
	30%	5,7	100%	5,70	*30%*	*1,71*	*70%*
	90%	43,2	95%	41,04	*30%*	*12,312*	*40%*
	15%	16	100%	16,00	*20%*	*3,2*	*40%*
	40%	5,6	100%	5,60	*60%*	*3,36*	*40%*
	39%	30	100%	30,00	*50%*	*15*	*30%*
	100%	31	100%	31,00	*71%*	*22*	*29%*
	100%	34	100%	34,00	*100%*	*34*	*0%*
	54%	22	100%	22,00	*100%*	*22*	*0%*
	100%	252	90%	226,80	*90%*	*204,12*	*0%*
	100%	19	100%	19,00	*70%*	*13,3*	*0%*
	45%	20,7	60%	12,42	*25%*	*3,105*	*35%*
	80%	19,2	80%	15,36	*60%*	*9,216*	*10%*
	30%	14,4	100%	14,40	*0%*	*0*	*30%*
	19%	7,189189189	100%	7,19	*100%*	*7,189189189*	*0%*
	65%	7,15	100%	7,15	*45%*	*3,2175*	*0%*
	70%	7	100%	7,00	*60%*	*4,2*	*40%*
	65%	13,65	40%	5,46	*0%*	*0*	*40%*
	100%	8	70%	5,60	*30%*	*1,68*	*70%*
	100%	21	100%	21,00	*20%*	*4,2*	*40%*
	100%	11	90%	9,90	*0%*	*0*	*40%*
	40%	45,2	80%	36,16	*60%*	*21,696*	*25%*
	50%	77	80%	61,60	*80%*	*49,28*	*20%*
	100%	19	100%	19,00	*30%*	*5,7*	*50%*
	90%	45	80%	36,00	*15%*	*5,4*	*20%*
	55%	20,35	100%	20,35	*30%*	*6,105*	*40%*
	45%	54,9	65%	35,69	*80%*	*28,548*	*20%*
	80%	8	85%	6,80	*60%*	*4,08*	*40%*
	10%	8,7	100%	8,70	*40%*	*3,48*	*60%*
	100%	30	50%	15,00	*35%*	*5,25*	*65%*
	80%	12	100%	12,00	30%	*3,6*	*20%*
	50%	105	85%	89,25	*30%*	*26,775*	*50%*
	90%	32,4	90%	29,16	*70%*	*20,412*	*30%*
	55%	129,8	60%	77,88	*50%*	*38,94*	*0%*
	100%	29	100%	29,00	*100%*	*29*	*0%*
	60%	8,4	100%	8,40	*80%*	*6,72*	*20%*
	40%	39,6	90%	35,64	*25%*	*8,91*	*50%*
	60%	20,4	60%	12,24	*0%*	*0*	*0%*
	85%	15,3	80%	12,24	*50%*	*6,12*	*25%*
	100%	50	100%	50,00	*100%*	*50*	*0%*
	100%	80	100%	80,00	*5%*	*4*	*25%*
	90%	36	100%	36,00	*100%*	*36*	*0%*
	63%	20	100%	20,00	*20%*	*4*	*80%*
	100%	19	100%	19,00	*15%*	*2,85*	*85%*
	90%	17,1	65%	11,12	*10%*	*1,1115*	*40%*
	100%	104	70%	72,80	*60%*	*43,68*	*20%*
	80%	17,6	100%	17,60	*20%*	*3,52*	*25%*
	90%	42,3	90%	38,07	*20%*	*7,614*	*10%*
	40%	15,6	30%	4,68	*10%*	*0,468*	*90%*
	100%	17	100%	17,00	*50%*	*8,5*	*40%*
	85%	46,75	95%	44,41	*40%*	*17,765*	*10%*
	71%	20	100%	20,00	*60%*	*12*	*40%*
	95%	29,45	95%	27,98	*60%*	*16,7865*	*25%*
	100%	31	100%	31,00	*40%*	*12,4*	*60%*
	85%	80,75	80%	64,60	*35%*	*22,61*	*45%*

	100%	16	100%	16,00	*35%*	*5,6*	*20%*
	85%	17	100%	17,00	*10%*	*1,7*	*25%*
	100%	11	100%	11,00	*40%*	*4,4*	*30%*
	100%	16	100%	16,00	*70%*	*11,2*	*30%*
	100%	72	100%	72,00	*53%*	*38*	*20%*
	10%	16,2	100%	16,20	*0%*	*0*	*70%*
	85%	36,55	45%	16,45	*30%*	*4,93425*	*60%*
	95%	28,5	60%	17,10	*10%*	*1,71*	*50%*
	40%	11,2	75%	8,40	*0%*	*0*	*100%*
	80%	44,8	100%	44,80	*25%*	*11,2*	*15%*
	100%	14	85%	11,90	*10%*	*1,19*	*80%*
	100%	25	100%	25,00	*30%*	*7,5*	*40%*
	12%	4,2	84%	3,53	*0%*	*0*	*0%*
	86%	12	100%	12,00	*40%*	*4,8*	*60%*
	80%	72,8	100%	72,80	*0%*	*0*	*60%*
	100%	39	63%	24,57	*20%*	*4,914*	*50%*
	30%	9,3	0%	-	*0%*		*0%*
	35%	12,25	70%	8,58	*10%*	*0,8575*	*90%*
	83%	24	60%	14,40	*0%*	*0*	*100%*
	77%	24	85%	20,40	*15%*	*3,06*	*45%*
	49%	19	50%	9,50	*80%*	*7,6*	*20%*
	100%	18	100%	18,00	*0%*	*0*	*70%*
	15%	7,2	60%	4,32	*100%*	*4,32*	*0%*
	8%	1,92	10%	0,19	*0%*	*0*	*0%*
	12%	3,84	0%	-	*0%*		*0%*
	18%	6,84	60%	4,10	*0%*	*0*	*100%*
	70%	13,3	100%	13,30	*0%*	*0*	*10%*
	16%	4,96	0%	-	*0%*		*0%*
	20%	8,2	15%	1,23	*3%*	*0,0369*	*97%*
	10%	3,9	100%	3,90	*0%*	*0*	*0%*
	4%	1,44	0%	-	*0%*		*0%*
	0%	0	0%	-	*0%*		*0%*
	0%	0	0%	-	*0%*		*0%*
	90%	16,2	70%	11,34	*20%*	*2,268*	*65%*
	100%	13	100%	13,00	*20%*	*2,6*	*50%*
	25%	5	20%	1,00	*0%*	*0*	*100%*
	100%	23	100%	23,00	*0%*	*0*	*0%*
	20%	3,2	60%	1,92	*0%*	*0*	*100%*
	95%	17,1	90%	15,39	*30%*	*4,617*	*70%*
	40%	22,4	0%	-	*0%*		*0%*
	100%	29	55%	15,95	*80%*	*12,76*	*0%*
	27%	12,42	0%	-	*0%*		*0%*
	18%	3,6	35%	1,26	*0%*	*0*	*0%*
	20%	10,8	50%	5,40	*80%*	*4,32*	*20%*
	85%	7,65	100%	7,65	*40%*	*3,06*	*0%*
	80%	32	95%	30,40	*75%*	*22,8*	*25%*
20	20%	0	100%	-	*100%*	*0*	*0%*
4	70%	0	100%	-	*80%*	*0*	*0%*
15	65%	0	50%	-	*70%*	*0*	*0%*
100+	60%	0	55%	-	*80%*	*0*	*0%*
	16%	6,08	100%	6,08	*50%*	*3,04*	*50%*
	10%	4,3	100%	4,30	*10%*	*0,43*	*90%*
	26%	7,54	100%	7,54	*30%*	*2,262*	*70%*
	72%	12,96	100%	12,96	*60%*	*7,776*	*40%*
	16%	8	100%	8,00	*63%*	*5*	*37%*
	22%	10,34	100%	10,34	*0%*	*0*	*100%*
	55%	17,05	100%	17,05	*15%*	*2,5575*	*85%*
	100%	18	100%	18,00	*80%*	*14,4*	*20%*
	100%	16	100%	16,00	*50%*	*8*	*30%*
	100%	51	100%	51,00	*60%*	*30,6*	*30%*
	95%	67,45	100%	67,45	*15%*	*10,1175*	*25%*
	100%	25	100%	25,00	*10%*	*2,5*	*40%*
	0%	0	0%	-	*0%*		*0%*
	20%	3	60%	1,80	*40%*	*0,72*	*60%*
	30%	4,2	0%	-	*0%*		*0%*
	0%	0	0%	-	*0%*		*0%*

	45%	9	100%	9,00	*0%*	*0*	*100%*
	100%	9	70%	6,30	*0%*	*0*	*100%*
	15%	5,25	0%	-	*0%*		*0%*
	15%	5,85	0%	-	*0%*		*0%*
	83%	14,11	100%	14,11	*17%*	*2,351666667*	*43%*
	81%	47	100%	47,00	*45%*	*21*	*21%*
	28%	14	100%	14,00	*0%*	*0*	*100%*
	0%	0	0%	-	*0%*		*0%*
	0%		0%	-	*0%*		*0%*
	0%	0	0%	-	*0%*		*0%*
	2%	0,88	100%	0,88	*100%*	*0,88*	*0%*
	0%	0	0%	-	*0%*		*0%*
	27%	7,83	50%	3,92	*0%*	*0*	*100%*
	64%	29	100%	29,00	*0%*	*0*	*100%*
	67%	47	62%	29,00	*40%*	*11,6*	*20%*
	79%	11	50%	5,50	*0%*	*0*	*20%*
	72%	31	60%	18,60	*30%*	*5,58*	*50%*
	0%	0	0%	-	*0%*		*0%*
	0%	0	0%	-	*0%*		*0%*
	100%	14	100%	14,00	*60%*	*8,4*	*25%*
	0%	0	0%	-	*0%*		*0%*
	53%	19	63%	12,00	*0%*	*0*	*100%*
	83%	19	60%	11,40	*20%*	*2,28*	*15%*
	10%	4,9	100%	4,90	*0%*	*0*	*100%*
	0%	0	0%	-	*0%*		*0%*
	0%	0	0%	-	*0%*		*0%*
	0%	0	0%	-	*0%*		*0%*
	13%	6,37	25%	1,59	*0%*	*0*	*100%*
	15%	4,5	75%	3,38	*20%*	*0,675*	*80%*
	15%	6,75	90%	6,08	*25%*	*1,51875*	*75%*
	20%	10,2	100%	10,20	*15%*	*1,53*	*85%*
	11%	4,51	100%	4,51	*20%*	*0,902*	*80%*
	8%	3,44	100%	3,44	*10%*	*0,344*	*90%*
	5%	2,15	100%	2,15	*7%*	*0,1575*	*93%*
	0%	0	0%	-	*0%*		*0%*
	0%	0	0%	-	*0%*		*0%*
	12%	5,88	0%	-	*0%*		*0%*
	3%	1,11	100%	1,11	*0%*	*0*	*100%*
	16%	6,24	100%	6,24	*100%*	*6,24*	*0%*
	17%	6,63	100%	6,63	*38%*	*2,535*	*62%*
100	20%	0	85%	-	*80%*	*0*	*20%*
100	80%	0	50%	-	*100%*	*0*	*0%*
15	10%	0	0%	-	*0%*		*0%*
	100%	12	40%	4,80	*75%*	*3,6*	*25%*
	75%	10,5	80%	8,40	*80%*	*6,72*	*20%*
	95%	17,1	20%	3,42	*40%*	*1,368*	*60%*
	100%	76	95%	72,20	*60%*	*43,32*	*0%*
	100%	39	75%	29,25	*40%*	*11,7*	*60%*
	90%	32,4	75%	24,30	*50%*	*12,15*	*30%*
	80%	20	100%	20,00	*80%*	*16*	*20%*
	100%	31	90%	27,90	*30%*	*8,37*	*60%*
	70%	7,7	100%	7,70	*30%*	*2,31*	*60%*
	70%	7,7	100%	7,70	*60%*	*4,62*	*30%*
	100%	57	85%	48,45	*20%*	*9,69*	*0%*
	100%	78	71%	55,38	*0%*	*0*	*10%*
	90%	60,3	100%	60,30	*10%*	*6,03*	*0%*
	70%	7,7	100%	7,70	*40%*	*3,08*	*20%*
	60%	18	100%	18,00	*0%*	*0*	*30%*
	65%	5,85	100%	5,85	*10%*	*0,585*	*40%*
4	10%	0	100%	-	*0%*	*0*	*100%*
9	40%	0	100%	-	*40%*	*0*	*60%*
	100%	40	40%	16,00	*20%*	*3,2*	*80%*
	50%	30,5	50%	15,25	*25%*	*3,8125*	*35%*
	10%	5,5	0%	-	*0%*		*0%*
	55%	21,45	100%	21,45	*100%*	*21,45*	*0%*
	80%	37,6	100%	37,60	*10%*	*3,76*	*40%*

	0%	0	0%	-	0%		0%
	60%	8,4	100%	8,40	50%	4,2	50%
	60%	23,4	100%	23,40	35%	8,19	30%
	100%	9	100%	9,00	20%	1,8	40%
	30%	8,7	100%	8,70	55%	4,785	35%
	75%	28,5	100%	28,50	60%	17,1	25%
	100%	24	100%	24,00	65%	15,6	30%
	100%	38	84%	32,00	20%	6,4	0%
	5%	1,4	100%	1,40	0%	0	100%
	100%	12	100%	12,00	40%	4,8	40%
	40%	101,2	20%	20,24	0%	0	100%
	82%	3,28	100%	3,28	40%	1,312	50%
	100%	22	100%	22,00	10%	2,2	0%
30	0%	0	0%	-	0%		0%
	22%	7,04	0%	-	0%		0%
20	10%	0	60%	-	0%	0	100%
8	15%	0	100%	-	0%	0	100%
25	15%	0	70%	-	0%	0	100%
18	0%	0	0%	-	0%		0%
	0%	0	0%	-	0%		0%
	100%	12	100%	12,00	20%	2,4	60%
	100%	75	100%	75,00	50%	37,5	0%
	75%	15	100%	15,00	30%	4,5	0%
	60%	25,2	90%	22,68	30%	6,804	70%
	80%	16,8	100%	16,80	75%	12,6	25%
	60%	51	100%	51,00	100%	51	0%
	5%	3,05	100%	3,05	100%	3,05	0%
	5%	2,9	100%	2,90	0%	0	0%
	100%	19	90%	17,10	80%	13,68	20%
	35%	5,95	15%	0,89	0%	0	100%
	20%	25	100%	25,00	100%	25	0%
	90%	38,7	100%	38,70	65%	25,155	20%
	0%	0	0%	-	0%		0%
	15%	21,45	100%	21,45	100%	21,45	0%
	56%	60	70%	42,00	70%	29,4	15%
	67%	28	55%	15,40	0%	0	100%
	33%	71	80%	56,80	40%	22,72	60%
	81%	26	100%	26,00	35%	9,1	65%
	90%	47,7	80%	38,16	30%	11,448	70%
	22%	11	55%	6,00	0%	0	100%
	20%	2,4	25%	0,60	75%	0,45	25%
	55%	18,15	0%	-	0%		0%
	33%	27,72	100%	27,72	50%	13,86	40%
	20%	13,2	100%	13,20	30%	3,96	70%
	100%	34	100%	34,00	30%	10,2	55%
	40%	28	100%	28,00	20%	5,6	40%
	100%	106	80%	84,80	70%	59,36	30%
	100%	13	80%	10,40	90%	9,36	10%
	77%	11,55	45%	5,20	10%	0,51975	70%
	68%	13,6	98%	13,33	65%	8,6632	35%
	100%	13	95%	12,35	80%	9,88	20%
	43%	20,21	100%	20,21	85%	17,1785	15%
	71%	55	50%	27,50	80%	22	20%
	80%	12,8	100%	12,80	90%	11,52	10%
	18%	40,68	100%	40,68	15%	6,102	75%
	100%	97	100%	97,00	100%	97	0%
	57%	12	100%	12,00	40%	4,8	60%
	55%	47,3	100%	47,30	80%	37,84	20%
	30%	25,8	100%	25,80	0%	0	100%
	10%	4,6	100%	4,60	0%	0	100%
	40%	6,8	100%	6,80	60%	4,08	40%
	15%	15,3	100%	15,30	0%	0	100%
	60%	16,2	50%	8,10	50%	4,05	50%
	20%	8,2	100%	8,20	50%	4,1	50%
	40%	15,2	55%	8,36	50%	4,18	50%
	37%	27,38	100%	27,38	5%	1,369	60%

20%	4,2	100%	4,20	40%	1,68	60%
8%	7,04	100%	7,04	23%	1,6192	64%
63%	12	100%	12,00	18%	2,16	44%
55%	7,15	100%	7,15	10%	0,715	60%
55%	33	100%	33,00	30%	9,9	70%
80%	66,4	65%	43,16	0%	0	100%
60%	16,2	75%	12,15	30%	3,645	45%
40%	11,2	100%	11,20	20%	2,24	80%
35%	30,8	100%	30,80	20%	6,16	80%
29%	20	78%	15,60	0%	0	80%
22%	3,52	100%	3,52	65%	2,288	35%
100%	13	100%	13,00	60%	7,8	32%
10%	3,6	100%	3,60	80%	2,88	20%
25%	12,5	100%	12,50	20%	2,5	80%
40%	36,4	0%	-	0%		0%
82%	11,48	100%	11,48	100%	11,48	0%
40%	33,6	100%	33,60	0%	0	100%
25%	19,25	100%	19,25	20%	3,85	80%
60%	20,4	80%	16,32	65%	10,608	35%
100%	20	100%	20,00	100%	20	0%
70%	73,5	80%	58,80	100%	58,8	0%
20%	16,6	30%	4,98	0%	0	100%
65%	31,2	100%	31,20	32%	9,984	64%
82%	36,08	100%	36,08	45%	16,236	45%
50%	22,5	100%	22,50	60%	13,5	40%
100%	10	100%	10,00	40%	4	50%
73%	16	100%	16,00	51%	8,16	11%
65%	20,15	40%	8,06	0%	0	80%
32%	40,32	100%	40,32	40%	16,128	60%
100%	11	100%	11,00	60%	6,6	40%
80%	10,4	100%	10,40	30%	3,12	70%
100%	16	100%	16,00	50%	8	50%
0%	0	0%	-	0%		0%
20%	13,8	80%	11,04	70%	7,728	30%
10%	1,1	100%	1,10	50%	0,55	50%
57%	13	100%	13,00	15%	1,95	75%
5%	14,55	100%	14,55	0%	0	100%
30%	4,2	100%	4,20	30%	1,26	70%
85%	38,25	100%	38,25	50%	19,125	35%
7%	2,59	100%	2,59	0%	0	100%
60%	22,2	100%	22,20	30%	6,66	70%
100%	18	100%	18,00	60%	10,8	40%
65%	16,9	100%	16,90	60%	10,14	40%
30%	5,7	100%	5,70	50%	2,85	50%
100%	11	100%	11,00	40%	4,4	60%
60%	15	100%	15,00	50%	7,5	50%
70%	23,1	100%	23,10	10%	2,31	50%
47%	8	100%	8,00	25%	2	65%
56%	14	100%	14,00	45%	6,3	40%
70%	39,2	40%	15,68	80%	12,544	20%
38%	8,74	100%	8,74	20%	1,748	70%
40%	21	20%	4,20	80%	3,36	20%
84%	15,96	100%	15,96	12%	1,9152	59%
56%	14	100%	14,00	50%	7	30%
22%	7,04	100%	7,04	70%	4,928	30%
77%	17,71	70%	12,40	70%	8,6779	30%
64%	18,56	95%	17,63	62%	10,93184	20%
88%	24,64	100%	24,64	60%	14,784	20%
20%	20,4	100%	20,40	0%	0	100%
90%	24,3	100%	24,30	70%	17,01	30%
65%	11	81%	8,91	10%	0,891	25%
80%	24,8	100%	24,80	0%	0	45%
100%	20	78%	15,60	15%	2,34	50%
100%	12	80%	9,60	35%	3,36	65%
70%	11,9	30%	3,57	80%	2,856	20%
0%	0	0%	-	0%		0%

	40%	80,8	0%	-	0%		0%
	100%	36	100%	36,00	30%	10,8	25%
	90%	38,7	100%	38,70	100%	38,7	0%
	55%	13,2	35%	4,62	30%	1,386	40%
	60%	22,8	100%	22,80	70%	15,96	30%
	100%	24	100%	24,00	10%	2,4	85%
	95%	98,8	100%	98,80	5%	4,94	10%
	70%	19,6	100%	19,60	35%	6,86	65%
	100%	25	100%	25,00	60%	15	30%
	38%	22,04	70%	15,43	80%	12,3424	20%
	100%	67	100%	67,00	25%	16,75	50%
	80%	28	75%	21,00	30%	6,3	50%
	73%	88	100%	88,00	30%	26,4	10%
	100%	43	90%	38,70	30%	11,61	70%
400	30%	70,2	40%	28,08	20%	5,616	80%
	100%	28	100%	28,00	35%	9,8	40%
25	20%	8	100%	8,00	0%	0	100%
	62%	9,3	100%	9,30	50%	4,65	50%
	8%	14,88	80%	11,90	0%	0	100%
	100%	25	100%	25,00	40%	10	60%
	85%	20,4	65%	13,26	25%	3,315	75%
	80%	16,8	100%	16,80	80%	13,44	20%
	60%	14,4	100%	14,40	25%	3,6	65%
	100%	34	50%	17,00	35%	5,95	65%
	50%	9,5	100%	9,50	40%	3,8	25%
	70%	12,6	100%	12,60	20%	2,52	50%
	100%	23	100%	23,00	30%	6,9	60%
	100%	30	100%	30,00	37%	11	63%
	100%	48	100%	48,00	60%	28,8	30%
	35%	62	100%	62,00	100%	62	0%
	80%	17,6	100%	17,60	15%	2,64	50%
	64%	71	100%	71,00	0%	0	0%
	48%	25,92	65%	16,85	10%	1,6848	70%
	35%	26,25	25%	6,56	80%	5,25	20%
	46%	26,68	100%	26,68	0%	0	0%
	10%	15,2	50%	7,60	0%	0	100%
	70%	15,4	100%	15,40	55%	8,47	35%
	90%	35,1	100%	35,10	20%	7,02	30%
	85%	60,35	75%	45,26	40%	18,105	45%
	7%	4,55	100%	4,55	30%	1,365	70%
180	30%	28,5	100%	28,50	100%	28,5	0%
	15%	7,05	100%	7,05	0%	0	100%
	85%	17	100%	17,00	65%	11,05	25%
	90%	80,1	100%	80,10	0%	0	20%
	25%	21	47%	9,87	30%	2,961	70%
	40%	12,4	100%	12,40	40%	4,96	60%
	80%	28,8	100%	28,80	60%	17,28	25%
	90%	35,1	65%	22,82	25%	5,70375	55%
	20%	14,8	100%	14,80	15%	2,22	85%
	75%	24	100%	24,00	15%	3,6	85%
	80%	27,2	55%	14,96	60%	8,976	30%
	100%	67	90%	60,30	0%	0	0%
	39%	24	90%	21,60	15%	3,24	85%
	70%	11,2	100%	11,20	30%	3,36	70%
	100%	16	100%	16,00	40%	6,4	60%
	20%	4,6	100%	4,60	100%	4,6	0%
	100%	29	100%	29,00	0%	0	60%
	40%	17,6	100%	17,60	0%	0	0%
	90%	11,7	100%	11,70	0%	0	100%
	55%	20,35	100%	20,35	100%	20,35	0%
	100%	33	90%	29,70	20%	5,94	30%
	100%	11	60%	6,60	25%	1,65	75%
	100%	41	80%	32,80	20%	6,56	30%
	100%	12	100%	12,00	20%	2,4	80%
	100%	13	100%	13,00	10%	1,3	60%
	100%	16	70%	11,20	30%	3,36	70%

	30%	3,6	100%	3,60	*80%*	*2,88*	*20%*
	15%	1,8	100%	1,80	*100%*	*1,8*	*0%*
	60%	19,8	70%	13,86	*100%*	*13,86*	*0%*
	10%	5,1	100%	5,10	*50%*	*2,55*	*50%*
	10%	2	100%	2,00	*0%*	*0*	*100%*
	90%	27,9	100%	27,90	*20%*	*5,58*	*40%*
	20%	14	100%	14,00	*0%*	*0*	*100%*
	62%	45	100%	45,00	*0%*	*0*	*100%*
4	50%	0	100%	-	*100%*	*0*	*0%*
3	0%	0	0%	-	*0%*		*0%*
	0%	0	0%	-	*0%*		*0%*
9	5%	0	100%	-	*100%*	*0*	*0%*
5	20%	0	100%	-	*100%*	*0*	*0%*
	80%	16	65%	10,40	*30%*	*3,12*	*20%*
8	0%	0	0%	-	*0%*		*0%*
	15%	3,15	100%	3,15	*50%*	*1,575*	*50%*
	50%	8	60%	4,80	*0%*	*0*	*100%*
	15%	2,4	100%	2,40	*100%*	*2,4*	*0%*
	100%	13	100%	13,00	*0%*	*0*	*30%*
	30%	5,1	100%	5,10	*80%*	*4,08*	*20%*
	40%	5,2	100%	5,20	*40%*	*2,08*	*20%*
	70%	15,4	66%	10,16	*70%*	*7,1148*	*30%*
7	14%	0	100%	-	*100%*	*0*	*0%*
	10%	3,5	100%	3,50	*50%*	*1,75*	*50%*
	25%	8,75	100%	8,75	*100%*	*8,75*	*0%*
	70%	21,7	100%	21,70	*0%*	*0*	*15%*
	80%	17,6	80%	14,08	*0%*	*0*	*35%*
40	30%	0	100%	-	*100%*	*0*	*0%*
	100%	20	100%	20,00	*10%*	*2*	*60%*
	100%	18	100%	18,00	*60%*	*10,8*	*40%*
	100%	15	100%	15,00	*40%*	*6*	*50%*
	90%	36	100%	36,00	*30%*	*10,8*	*0%*
	15%	1,95	100%	1,95	*100%*	*1,95*	*0%*
14	21%	0	100%	-	*70%*	*0*	*30%*
	100%	8	100%	8,00	*15%*	*1,2*	*60%*
	100%	21	100%	21,00	*40%*	*8,4*	*30%*
10	0%	0	0%	-	*0%*		*0%*
25	0%	0	0%	-	*0%*		*0%*
	39%	86	84%	72,00	*100%*	*72*	*0%*
	68%	25	100%	25,00	*24%*	*6*	*76%*
	58%	17,98	50%	8,99	*0%*	*0*	*100%*
	100%	16	95%	15,20	*15%*	*2,28*	*85%*
	0%	0	0%	-	*0%*		*0%*
20	0%	0	0%	-	*0%*		*0%*
	60%	10,8	100%	10,80	*20%*	*2,16*	*80%*
15	0%	0	0%	-	*0%*		*0%*
	68%	13	100%	13,00	*17%*	*2,21*	*64%*
18	11%	0	100%	-	*100%*	*0*	*0%*
4	0%	0	0%	-	*0%*		*0%*
3	0%	0	0%	-	*0%*		*0%*
5	0%	0	0%	-	*0%*		*0%*
8	0%	0	0%	-	*0%*		*0%*
	100%	37	80%	29,60	*65%*	*19,24*	*25%*
	100%	13	100%	13,00	*60%*	*7,8*	*40%*
	52%	17	100%	17,00	*77%*	*13,09*	*23%*
	100%	30	20%	6,00	*100%*	*6*	*0%*
800	35%	0	100%	-	*50%*	*0*	*50%*
	67%	23,45	95%	22,28	*30%*	*6,68325*	*70%*
	90%	39,6	100%	39,60	*0%*	*0*	*20%*
	45%	21,6	100%	21,60	*15%*	*3,24*	*85%*
	64%	12,8	100%	12,80	*0%*	*0*	*100%*
	90%	17,1	80%	13,68	*40%*	*5,472*	*60%*
	56%	10	100%	10,00	*20%*	*2*	*50%*
	40%	5,2	100%	5,20	*100%*	*5,2*	*0%*
	100%	19	100%	19,00	*35%*	*6,65*	*35%*
	70%	19,6	100%	19,60	*65%*	*12,74*	*30%*

	100%	17	100%	17,00	*100%*	*17*	*0%*
	43%	9	100%	9,00	*35%*	*3,15*	*65%*
	80%	18,4	100%	18,40	*100%*	*18,4*	*0%*
	0%	0	0%	-	*0%*		*0%*
	100%	15	100%	15,00	*0%*	*0*	*100%*
	100%	49	82%	40,18	*0%*	*0*	*17%*
	83%	9,96	41%	4,08	*0%*	*0*	*0%*
15	60%	0	100%	-	*100%*	*0*	*0%*
	73%	11	100%	11,00	*100%*	*11*	*0%*
	79%	18,85714286	70%	13,20	*0%*	*0*	*100%*
	100%	17	100%	17,00	*15%*	*2,55*	*55%*
	100%	19	100%	19,00	*25%*	*4,75*	*75%*
	43%	12	100%	12,00	*100%*	*12*	*0%*
	0%	0	0%	-	*0%*		*0%*
	64%	14	100%	14,00	*40%*	*5,6*	*50%*
	100%	43	100%	43,00	*65%*	*27,95*	*35%*
	100%	16	100%	16,00	*20%*	*3,2*	*80%*
	85%	8,5	100%	8,50	*35%*	*3*	*30%*
	85%	17	100%	17,00	*0%*	*0*	*65%*
	80%	12,8	100%	12,80	*29%*	*3,657142857*	*50%*
	34%	12	100%	12,00	*0%*	*0*	*100%*
	73%	48	100%	48,00	*40%*	*19,2*	*60%*
	81%	13	100%	13,00	*23%*	*3*	*36%*
	100%	23	100%	23,00	*30%*	*6,9*	*0%*
	0%	0	0%	-	*0%*		*0%*
	100%	52	85%	44,20	*55%*	*24,31*	*35%*
	100%	80	80%	64,00	*20%*	*12,8*	*50%*
	75%	37,5	75%	28,13	*20%*	*5,625*	*40%*
	100%	18	100%	18,00	*25%*	*4,5*	*25%*
	100%	36	100%	36,00	*50%*	*18*	*30%*
	100%	59	81%	48,00	*19%*	*9*	*41%*
	100%	53	85%	45,05	*15%*	*6,7575*	*45%*
	100%	30	100%	30,00	*0%*	*0*	*100%*
	100%	38	100%	38,00	*29%*	*11*	*62%*
	100%	26	90%	23,40	*20%*	*4,68*	*60%*
	100%	16	100%	16,00	*0%*	*0*	*100%*
	83%	29	70%	20,30	*5%*	*1,015*	*15%*
	5%	8,9	100%	8,90	*0%*	*0*	*0%*
	80%	42,4	100%	42,40	*15%*	*6,36*	*50%*
	78%	112,32	67%	75,25	*10%*	*7,52544*	*40%*
	73%	19	100%	19,00	*15%*	*2,85*	*35%*
	88%	18,48	100%	18,48	*32%*	*5,9136*	*16%*
18	0%	0	0%	-	*0%*		*0%*
	25%	5	100%	5,00	*30%*	*1,5*	*70%*
16	0%	0	0%	-	*0%*		*0%*
	59%	16	100%	16,00	*0%*	*0*	*40%*
	100%	19	100%	19,00	*30%*	*5,7*	*20%*
	15%	11,7	0%	-	*0%*		*0%*
	55%	13,75	80%	11,00	*0%*	*0*	*100%*
48	2%	0	0%	-	*0%*		*0%*
	36%	8	75%	6,00	*17%*	*1*	*33%*
	0%	0	0%	-	*0%*		*0%*
	60%	9	100%	9,00	*0%*	*0*	*60%*
	100%	56	100%	56,00	*8%*	*4,48*	*0%*
	85%	37,4	100%	37,40	*30%*	*11,22*	*58%*
	37%	35	100%	35,00	*0%*	*0*	*0%*
	25%	33	100%	33,00	*0%*	*0*	*0%*
	20%	4,4	0%	-	*0%*		*0%*
	90%	11,7	100%	11,70	*15%*	*1,755*	*80%*
	68%	25,84	95%	24,55	*26%*	*6,312342857*	*74%*
	100%	47	85%	39,95	*32%*	*12,784*	*52%*
	0%	0	0%	-	*0%*		*0%*
5	10%	0	100%	-	*100%*	*0*	*0%*
6	17%	0	100%	-	*100%*	*0*	*0%*
3	100%	0	100%	-	*60%*	*0*	*40%*
7	0%	0	0%	-	*0%*		*0%*

	10%	2,1	0%	-	*0%*		*0%*
	0%	0	0%	-	*0%*		*0%*
	0%	0	0%	-	*0%*		*0%*
	100%	19	74%	14,00	*20%*	*2,8*	*80%*
	30%	10,2	100%	10,20	*50%*	*5,1*	*50%*
	75%	12	100%	12,00	*25%*	*3*	*65%*
	32%	20,48	100%	20,48	*72%*	*14,72*	*28%*
	85%	22	80%	17,60	*45%*	*7,92*	*25%*
	80%	29,6	100%	29,60	*8%*	*2,368*	*0%*
	100%	18	100%	18,00	*10%*	*1,8*	*90%*
	0%	0	0%	-	*0%*		*0%*
	100%	17	100%	17,00	*24%*	*4*	*76%*
	31%	8	100%	8,00	*0%*	*0*	*100%*
	65%	11	100%	11,00	*20%*	*2,2*	*80%*
	31%	5	100%	5,00	*40%*	*2*	*60%*
	33%	4,95	84%	4,16	*100%*	*4,158*	*0%*
	100%	16	100%	16,00	*0%*	*0*	*100%*
	0%	0	0%	-	*0%*		*0%*
32	16%	0	100%	-	*100%*	*0*	*0%*
60	22%	0	100%	-	*100%*	*0*	*0%*
25	16%	0	100%	-	*100%*	*0*	*0%*
	100%	29	65%	18,85	*75%*	*14,1375*	*25%*
	0%	0	0%	-	*0%*		*0%*
	100%	18	100%	18,00	*39%*	*7*	*28%*
	100%	21	86%	18,00	*40%*	*7,2*	*45%*
	10%	12,5	100%	12,50	*0%*	*0*	*100%*
	69%	11	100%	11,00	*70%*	*7,7*	*30%*
	44%	16	100%	16,00	*44%*	*7*	*0%*
	11%	4	0%	-	*0%*		*0%*
	100%	18	100%	18,00	*42%*	*7,56*	*50%*
	100%	30	100%	30,00	*7%*	*2,1*	*93%*
	75%	9,75	100%	9,75	*20%*	*1,95*	*40%*
22	20%	3,4	0%	-	*0%*		*0%*
	70%	16,1	100%	16,10	*40%*	*6,44*	*30%*
20	25%	0	40%	-	*100%*	*0*	*0%*
9	0%	0	0%	-	*0%*		*0%*
	41%	7	100%	7,00	*60%*	*4,2*	*40%*
	100%	14	100%	14,00	*30%*	*4,2*	*70%*
	58%	11	100%	11,00	*100%*	*11*	*0%*
	87%	13	100%	13,00	*70%*	*9,1*	*30%*
	100%	20	100%	20,00	*10%*	*2*	*25%*
	85%	45,05	69%	31,08	*20%*	*6,2169*	*20%*
	100%	22	100%	22,00	*15%*	*3,3*	*20%*
1	0%	0	0%	-	*0%*		*0%*
4	50%	0	100%	-	*100%*	*0*	*0%*
34	0%	0	0%	-	*0%*		*0%*
25	0%	0	0%	-	*0%*		*0%*
30	35%	0	30%	-	*50%*	*0*	*50%*
	10%	7,8	100%	7,80	*50%*	*3,9*	*50%*
6	100%	0	100%	-	*100%*	*0*	*0%*
8	69%	0	100%	-	*100%*	*0*	*0%*
28	24%	0	0%	-	*0%*		*0%*
13	100%	0	100%	-	*100%*	*0*	*0%*
7	21%	0	100%	-	*100%*	*0*	*0%*
	98%	136,22	55%	74,92	*0%*	*0*	*0%*
30	47%	0	100%	-	*100%*	*0*	*0%*
56	80%	0	100%	-	*100%*	*0*	*0%*
48	24%	0	100%	-	*100%*	*0*	*0%*
100+	90%	0	100%	-	*100%*	*0*	*0%*
	95%	129,2	70%	90,44	*10%*	*9,044*	*20%*
	57%	70	100%	70,00	*0%*	*0*	*30%*
18	75%	0	100%	-	*100%*	*0*	*0%*
11	100%	0	100%	-	*100%*	*0*	*0%*
5	100%	0	100%	-	*100%*	*0*	*0%*
14	40%	0	75%	-	*100%*	*0*	*0%*
	23%	19	100%	19,00	*100%*	*19*	*0%*

42	20%	0	35%	-	*100%*	*0*	*0%*
30	50%	0	100%	-	*100%*	*0*	*0%*
26	0%	0	0%	-	*0%*		*0%*
34	22%	0	100%	-	*100%*	*0*	*0%*
18	15%	0	100%	-	*0%*	*0*	*100%*
	0%	0	0%	-	*0%*		*0%*
12	4%	0	100%	-	*0%*	*0*	*100%*
	38%	32,68	0%	-	*0%*		*0%*
	15%	12	100%	12,00	*40%*	*4,8*	*60%*
14	100%	0	100%	-	*100%*	*0*	*0%*
30	12%	0	100%	-	*100%*	*0*	*0%*
20	30%	0	100%	-	*100%*	*0*	*0%*
28	40%	0	100%	-	*100%*	*0*	*0%*
100+	34%	0	100%	-	*100%*	*0*	*0%*
100+	14%	0	100%	-	*60%*	*0*	*40%*
30	55%	0	100%	-	*100%*	*0*	*0%*
23	10%	0	0%	-	*0%*		*0%*
45	40%	0	100%	-	*100%*	*0*	*0%*
22	32%	0	100%	-	*100%*	*0*	*0%*
14	100%	0	45%	-	*100%*	*0*	*0%*
24	0%	0	0%	-	*0%*		*0%*
35	10%	0	60%	-	*30%*	*0*	*70%*
9	60%	0	100%	-	*100%*	*0*	*0%*
28	12%	0	100%	-	*50%*	*0*	*50%*
5	20%	0	100%	-	*100%*	*0*	*0%*
60	0%	0	0%	-	*0%*		*0%*
26	8%	0	0%	-	*0%*		*0%*
30	55%	0	100%	-	*100%*	*0*	*0%*
33	9%	0	100%	-	*100%*	*0*	*0%*
18	28%	0	100%	-	*100%*	*0*	*0%*
13	38%	0	40%	-	*0%*	*0*	*0%*
50	15%	0	100%	-	*100%*	*0*	*0%*
26	25%	0	100%	-	*100%*	*0*	*0%*
12	42%	0	100%	-	*100%*	*0*	*0%*
14	0%	0	0%	-	*0%*		*0%*
17	0%	0	0%	-	*0%*		*0%*
3	100%	0	100%	-	*100%*	*0*	*0%*
60	100%	0	100%	-	*100%*	*0*	*0%*
45	100%	0	100%	-	*100%*	*0*	*0%*
55	30%	0	70%	-	*80%*	*0*	*20%*
32	60%	0	100%	-	*100%*	*0*	*0%*
10	60%	0	100%	-	*100%*	*0*	*0%*
8	75%	0	100%	-	*100%*	*0*	*0%*
12	42%	0	100%	-	*100%*	*0*	*0%*
8	0%	0	0%	-	*0%*		*0%*
30	100%	0	100%	-	*100%*	*0*	*0%*
10	10%	0	0%	-	*0%*		*0%*
10	20%	0	100%	-	*100%*	*0*	*0%*
35	15%	0	100%	-	*60%*	*0*	*40%*
30	60%	0	80%	-	*50%*	*0*	*50%*
15	13%	0	100%	-	*50%*	*0*	*50%*
8	0%	0	0%	-	*0%*		*0%*
50	0%	0	0%	-	*0%*		*0%*
100	20%	0	100%	-	*100%*	*0*	*0%*
28	0%	0	0%	-	*0%*		*0%*
25	20%	0	100%	-	*100%*	*0*	*0%*
35	40%	0	100%	-	*100%*	*0*	*0%*
25	0%	0	0%	-	*0%*		*0%*
9	11%	0	100%	-	*100%*	*0*	*0%*
15	13%	0	100%	-	*100%*	*0*	*0%*
34	50%	0	20%	-	*50%*	*0*	*50%*
50	20%	0	100%	-	*100%*	*0*	*0%*
32	85%	0	100%	-	*100%*	*0*	*0%*
28	32%	0	100%	-	*100%*	*0*	*0%*
55	70%	0	100%	-	*100%*	*0*	*0%*
8	0%	0	0%	-	*0%*		*0%*

13	0%	0	0%	-	*0%*		*0%*
24	16%	0	100%	-	*100%*	*0*	*0%*
35	34%	0	100%	-	*100%*	*0*	*0%*
17	18%	0	0%	-	*0%*		*0%*
44	7%	0	100%	-	*100%*	*0*	*0%*
14	7%	0	100%	-	*100%*	*0*	*0%*
19	5%	0	0%	-	*0%*		*0%*
10	50%	0	100%	-	*100%*	*0*	*0%*
40	88%	0	100%	-	*100%*	*0*	*0%*
21	43%	0	100%	-	*100%*	*0*	*0%*
5	100%	0	0%	-	*0%*		*0%*
12	17%	0	100%	-	*100%*	*0*	*0%*
23	70%	0	0%	-	*0%*		*0%*
5	100%	0	100%	-	*100%*	*0*	*0%*
90	70%	0	100%	-	*40%*	*0*	*60%*
2	30%	0	100%	-	*100%*	*0*	*0%*
5	30%	0	100%	-	*100%*	*0*	*0%*
7	50%	0	100%	-	*100%*	*0*	*0%*
29	75%	0	60%	-	*100%*	*0*	*0%*
10	100%	0	100%	-	*20%*	*0*	*50%*
3	100%	0	67%	-	*100%*	*0*	*0%*
18	100%	0	100%	-	*100%*	*0*	*0%*
12	60%	0	100%	-	*100%*	*0*	*0%*
9	0%	0	0%	-	*0%*		*0%*
25	15%	0	100%	-	*100%*	*0*	*0%*
22	0%	0	0%	-	*0%*		*0%*
15	15%	0	0%	-	*0%*		*0%*
90	70%	0	0%	-	*0%*		*0%*
100+	75%	0	100%	-	*100%*	*0*	*0%*
60	100%	0	100%	-	*100%*	*0*	*0%*
25	25%	0	0%	-	*0%*		*0%*
35	6%	0	0%	-	*0%*		*0%*
18	39%	0	100%	-	*100%*	*0*	*0%*
10	20%	0	100%	-	*40%*	*0*	*60%*
25	80%	0	100%	-	*100%*	*0*	*0%*
18	11%	0	100%	-	*0%*	*0*	*80%*
15	30%	0	50%	-	*0%*	*0*	*0%*
20	15%	0	100%	-	*100%*	*0*	*0%*
100+	45%	0	55%	-	*100%*	*0*	*0%*
20	0%	0	0%	-	*0%*		*0%*
100+	0%	0	0%	-	*0%*		*0%*
100+	4%	0	100%	-	*100%*	*0*	*0%*
30	13%	0	0%	-	*0%*		*0%*
7	29%	0	100%	-	*100%*	*0*	*0%*
3	67%	0	100%	-	*0%*	*0*	*60%*
25	10%	0	0%	-	*0%*		*0%*
100+	20%	0	80%	-	*0%*	*0*	*0%*
17	29%	0	100%	-	*100%*	*0*	*0%*
12	25%	0	100%	-	*100%*	*0*	*0%*
8	38%	0	100%	-	*100%*	*0*	*0%*
4	0%	0	0%	-	*0%*		*0%*
12	42%	0	100%	-	*100%*	*0*	*0%*
36	17%	0	100%	-	*100%*	*0*	*0%*
50	60%	0	100%	-	*100%*	*0*	*0%*
45	25%	0	100%	-	*100%*	*0*	*0%*
12	42%	0	100%	-	*100%*	*0*	*0%*
16	38%	0	100%	-	*100%*	*0*	*0%*
35	71%	0	100%	-	*100%*	*0*	*0%*
6	33%	0	100%	-	*100%*	*0*	*0%*
100+	100%	0	100%	-	*100%*	*0*	*0%*
100+	80%	0	75%	-	*100%*	*0*	*0%*
100+	90%	0	100%	-	*100%*	*0*	*0%*
100+	15%	0	100%	-	*100%*	*0*	*0%*
100+	50%	0	33%	-	*100%*	*0*	*0%*
100+	72%	0	33%	-	*100%*	*0*	*0%*
	100%	30	53%	16,00	*0%*	*0*	*0%*

	100%	46	89%	41,00	*4%*	*1,64*	*0%*
	70%	35	69%	24,00	*42%*	*10*	*58%*
100	84%	0	0%	-	*0%*		*0%*
	100%	41	75%	30,75	*30%*	*9,225*	*70%*
	8%	11	100%	11,00	*100%*	*11*	*0%*
	0%	0	0%	-	*0%*		*0%*
	35%	12,6	100%	12,60	*50%*	*6,3*	*50%*
	70%	18,9	59%	11,20	*40%*	*4,48*	*60%*
35	35%	10,85	0%	-	*0%*		*0%*
	30%	16	100%	16,00	*100%*	*16*	*0%*
	30%	31,2	100%	31,20	*0%*	*0*	*20%*
	25%	17,25	100%	17,25	*100%*	*17,25*	*0%*
	25%	24,25	100%	24,25	*0%*	*0*	*100%*
	8%	5	100%	5,00	*100%*	*5*	*0%*
	60%	34	38%	13,00	*100%*	*13*	*0%*
	21%	6	100%	6,00	*100%*	*6*	*0%*
	45%	9	80%	7,20	*50%*	*3,6*	*50%*
	100%	39	85%	33,00	*30%*	*9,9*	*70%*
	38%	21	76%	16,00	*20%*	*3,2*	*70%*
	100%	32	91%	29,00	*12%*	*3,48*	*88%*
	60%	136	31%	42,00	*100%*	*42*	*0%*
	48%	36	0%	-	*0%*		*0%*
	67%	76	50%	38,00	*50%*	*19*	*50%*
	15%	8,1	95%	7,70	*67%*	*5,13*	*33%*
	100%	40	83%	33,00	*38%*	*12,54*	*44%*
	83%	25	95%	23,75	*38%*	*9,134615385*	*62%*
	71%	17	47%	7,99	*100%*	*7,99*	*0%*
	100%	51	73%	37,22	*74%*	*27,54*	*26%*
	10%	1,3	100%	1,30	*100%*	*1,3*	*0%*
	61%	27	89%	24,00	*46%*	*11*	*54%*
	13%	10	100%	10,00	*100%*	*10*	*0%*
	40%	28,8	100%	28,80	*100%*	*28,8*	*0%*
	20%	4,8	100%	4,80	*50%*	*2,4*	*50%*
	100%	16	100%	16,00	*25%*	*4*	*35%*
	100%	44	100%	44,00	*0%*	*0*	*0%*
	54%	29,36842105	100%	29,37	*42%*	*12,31578947*	*58%*
	36%	58	100%	58,00	*100%*	*58*	*0%*
	86%	56	57%	32,00	*15%*	*4,8*	*0%*
	40%	16	100%	16,00	*100%*	*16*	*0%*
	29%	26	100%	26,00	*100%*	*26*	*0%*
	14%	15	87%	13,00	*0%*	*0*	*100%*
	68%	23	100%	23,00	*57%*	*13,11*	*25%*
	81%	26	85%	22,10	*20%*	*4,42*	*60%*
	80%	56,8	100%	56,80	*40%*	*22,72*	*60%*
	100%	35	82%	28,82	*46%*	*13,38235294*	*18%*
	0%	0	0%	-	*0%*		*0%*
	75%	18	100%	18,00	*10%*	*1,8*	*20%*
	100%	14	100%	14,00	*80%*	*11,2*	*20%*
	100%	36	100%	36,00	*30%*	*10,8*	*55%*
	85%	23,8	90%	21,42	*20%*	*4,284*	*10%*
	0%	0	0%	-	*0%*		*0%*
	52%	25	100%	25,00	*0%*	*0*	*0%*
1	100%	0	100%	-	*0%*	*0*	*0%*
	80%	37,6	100%	37,60	*0%*	*0*	*10%*
	100%	39	100%	39,00	*100%*	*39*	*0%*
	0%	0	100%	-	*20%*	*0*	*15%*
	100%	30	100%	30,00	*29%*	*8,709677419*	*71%*
	100%	21	100%	21,00	*70%*	*14,7*	*30%*
	20%	17,4	100%	17,40	*10%*	*1,74*	*0%*
	89%	17	100%	17,00	*32%*	*5,44*	*0%*
	15%	7,35	100%	7,35	*20%*	*1,47*	*80%*
	20%	15,4	100%	15,40	*0%*	*0*	*100%*
	100%	21	85%	17,85	*40%*	*7,14*	*50%*
	100%	31	100%	31,00	*60%*	*18,6*	*40%*
	80%	66,4	30%	19,92	*45%*	*8,964*	*40%*
	96%	30,72	100%	30,72	*30%*	*9,216*	*20%*

	85%	42,5	95%	40,38	*65%*	*26,24375*	*0%*
	98%	28,42	100%	28,42	*45%*	*12,789*	*10%*
	85%	25,5	80%	20,40	*30%*	*6,12*	*10%*
	50%	13,5	100%	13,50	*10%*	*1,35*	*30%*
	0%	0	0%	-	*0%*		*0%*
	88%	23,76	100%	23,76	*60%*	*14,256*	*10%*
	100%	52	100%	52,00	*21%*	*11*	*60%*
	100%	25	80%	20,00	*30%*	*6*	*0%*
	100%	23	80%	18,40	*0%*	*0*	*20%*
	40%	4	0%	-	*0%*		*0%*
	100%	18	100%	18,00	*60%*	*10,8*	*40%*
	90%	13,5	100%	13,50	*15%*	*2,025*	*75%*
	0%	0	0%	-	*0%*		*0%*
	0%	0	0%	-	*0%*		*0%*
	100%	81	100%	81,00	*40%*	*32,4*	*20%*
	100%	21	100%	21,00	*0%*	*0*	*20%*
	100%	56	100%	56,00	*0%*	*0*	*100%*
	100%	73	100%	73,00	*5%*	*3,65*	*85%*
	100%	65	100%	65,00	*60%*	*39*	*40%*
	85%	20,4	100%	20,40	*30%*	*6,12*	*70%*
	24%	12	0%	-	*0%*		*0%*
	20%	6,8	100%	6,80	*15%*	*1,02*	*85%*
	67%	66	100%	66,00	*0%*	*0*	*0%*
	60%	29,4	100%	29,40	*70%*	*20,58*	*30%*
	90%	57,6	85%	48,96	*44%*	*21,5424*	*40%*
	0%	0	0%	-	*0%*		*0%*
	63%	35	100%	35,00	*0%*	*0*	*15%*
	80%	21,6	100%	21,60	*40%*	*8,64*	*20%*
	38%	49	82%	40,00	*75%*	*30*	*25%*
	100%	39	100%	39,00	*30%*	*11,7*	*30%*
	100%	48	100%	48,00	*30%*	*14,4*	*20%*
	28%	16	100%	16,00	*85%*	*13,6*	*15%*
	100%	38	100%	38,00	*68%*	*25,84*	*15%*
	100%	32	100%	32,00	*40%*	*12,8*	*50%*
	34%	19	100%	19,00	*100%*	*19*	*0%*
	60%	35,4	55%	19,47	*100%*	*19,47*	*0%*
	60%	22,2	90%	19,98	*100%*	*19,98*	*0%*
50	88%	0	65%	-	*70%*	*0*	*30%*
	40%	19	100%	19,00	*100%*	*19*	*0%*
	0%	0	0%	-	*0%*		*0%*
	30%	9,6	100%	9,60	*100%*	*9,6*	*0%*
	20%	8	100%	8,00	*0%*	*0*	*100%*
	87%	13,05	100%	13,05	*60%*	*7,83*	*35%*
	60%	51,6	100%	51,60	*0%*	*0*	*0%*
	81%	30	100%	30,00	*15%*	*4,5*	*25%*
	92%	46,92	84%	39,41	*70%*	*27,58896*	*30%*
	90%	44,1	70%	30,87	*65%*	*20,0655*	*10%*
	70%	28,7	80%	22,96	*40%*	*9,184*	*60%*
	20%	11,2	100%	11,20	*0%*	*0*	*100%*
	10%	4,9	0%	-	*0%*		*0%*
	87%	48	100%	48,00	*32%*	*15,36*	*25%*
	100%	52	100%	52,00	*60%*	*31,2*	*25%*
	77%	23	100%	23,00	*87%*	*20*	*13%*
	70%	25,9	100%	25,90	*30%*	*7,77*	*40%*
	97%	44,62	71%	31,73	*40%*	*12,69191111*	*40%*
	67%	34	90%	30,60	*0%*	*0*	*100%*
	100%	27	100%	27,00	*15%*	*4,05*	*85%*
	82%	33,62	100%	33,62	*22%*	*7,471111111*	*78%*
	16%	31	100%	31,00	*80%*	*24,8*	*20%*
	60%	22,2	75%	16,65	*10%*	*1,665*	*50%*
	100%	16	100%	16,00	*70%*	*11,2*	*30%*
	83%	15	100%	15,00	*53%*	*8*	*47%*
	85%	14,45	100%	14,45	*15%*	*2,1675*	*65%*
	20%	5,6	100%	5,60	*100%*	*5,6*	*0%*
	61%	17	80%	13,60	*50%*	*6,8*	*0%*
	100%	32	100%	32,00	*38%*	*12*	*35%*

	100%	19	100%	19,00	*85%*	*16,15*	*15%*
	90%	18,9	100%	18,90	*15%*	*2,835*	*45%*
	100%	31	100%	31,00	*20%*	*6,2*	*0%*
	100%	87	100%	87,00	*30%*	*26,1*	*40%*
	100%	36	100%	36,00	*40%*	*14,4*	*60%*
	96%	24	100%	24,00	*29%*	*7*	*65%*
	100%	32	100%	32,00	*15%*	*4,8*	*65%*
	100%	22	100%	22,00	*15%*	*3,3*	*75%*
14	7%	1	100%	1,00	*50%*	*0,5*	*50%*
	100%	15	100%	15,00	*20%*	*3*	*80%*
	100%	40	100%	40,00	*75%*	*30*	*25%*
	0%	0	0%	-	*0%*		*0%*
	33%	55	100%	55,00	*100%*	*55*	*0%*
	35%	31,5	100%	31,50	*40%*	*12,6*	*60%*
	70%	31,5	100%	31,50	*25%*	*7,875*	*50%*
	55%	36,3	100%	36,30	*65%*	*23,595*	*35%*
	44%	12	100%	12,00	*100%*	*12*	*0%*
	0%	0	0%	-	*0%*		*0%*
	40%	130,4	100%	130,40	*50%*	*65,2*	*50%*
	100%	38	100%	38,00	*60%*	*22,8*	*40%*
	45%	12,15	100%	12,15	*0%*	*0*	*40%*
	82%	38,54	80%	30,83	*20%*	*6,1664*	*50%*
	40%	10	100%	10,00	*0%*	*0*	*50%*
	45%	30,6	100%	30,60	*0%*	*0*	*30%*
	65%	35	100%	35,00	*0%*	*0*	*0%*
	68%	59	100%	59,00	*0%*	*0*	*20%*
	60%	19,8	40%	7,92	*100%*	*7,92*	*0%*
	30%	5,7	100%	5,70	*40%*	*2,28*	*60%*
	100%	29	100%	29,00	*34%*	*10*	*0%*
	100%	38	100%	38,00	*32%*	*12*	*3%*
	82%	45,92	100%	45,92	*45%*	*20,664*	*10%*
	100%	28	100%	28,00	*15%*	*4,2*	*55%*
	0%	0	0%	-	*0%*		*0%*
	80%	112	0%	-	*0%*		*0%*
	100%	19	92%	17,48	*70%*	*12,236*	*30%*
28	40%	11,6	100%	11,60	*100%*	*11,6*	*0%*
	0%	0	0%	-	*0%*		*0%*
	14%	4,722222222	100%	4,72	*100%*	*4,722222222*	*0%*
	100%	30	100%	30,00	*0%*	*0*	*68%*
	20%	10	100%	10,00	*0%*	*0*	*100%*
	0%	0	0%	-	*0%*		*0%*
10	20%	3,8	100%	3,80	*100%*	*3,8*	*0%*
	100%	35	100%	35,00	*37%*	*13*	*43%*
	100%	51	100%	51,00	*24%*	*12,24*	*66%*
	100%	32	66%	21,00	*15%*	*3,15*	*70%*
	65%	19,5	100%	19,50	*78%*	*15,21*	*22%*
	70%	18,2	100%	18,20	*20%*	*3,64*	*60%*
	100%	127	100%	127,00	*70%*	*88,9*	*25%*
	100%	12	85%	10,20	*80%*	*8,16*	*15%*
	100%	18	100%	18,00	*100%*	*18*	*0%*
	86%	18	100%	18,00	*28%*	*5*	*12%*
	100%	37	78%	29,00	*0%*	*0*	*0%*
	100%	22	100%	22,00	*20%*	*4,4*	*50%*
	68%	13	54%	7,00	*86%*	*6*	*14%*
	17%	14	0%	-	*0%*		*0%*
	100%	88	85%	75,00	*12%*	*9*	*16%*
	100%	56	54%	30,24	*0%*	*0*	*24%*
	100%	38	80%	30,40	*30%*	*9,12*	*70%*
	100%	37	100%	37,00	*90%*	*33,3*	*10%*
	84%	27	100%	27,00	*40%*	*10,8*	*20%*
	100%	24	100%	24,00	*30%*	*7,2*	*60%*
	70%	24,5	100%	24,50	*100%*	*24,5*	*0%*
	60%	29,4	100%	29,40	*14%*	*4,116*	*58%*
	100%	15	75%	11,25	*20%*	*2,25*	*80%*
	90%	19	100%	19,00	*26%*	*5*	*0%*
	73%	16	36%	5,76	*100%*	*5,76*	*0%*

	100%	20	100%	20,00	*100%*	*20*	*0%*
	70%	25,2	100%	25,20	*0%*	*0*	*0%*
15	10%	2,2	100%	2,20	*100%*	*2,2*	*0%*
	100%	39	100%	39,00	*21%*	*8,19*	*0%*
4	75%	0	100%	-	*20%*	*0*	*0%*
	100%	100	100%	100,00	*7%*	*7*	*0%*
	100%	45	70%	31,50	*50%*	*15,75*	*50%*
	100%	60	100%	60,00	*0%*	*0*	*0%*
	100%	27	100%	27,00	*11%*	*3*	*0%*
	100%	11	100%	11,00	*0%*	*0*	*0%*
	100%	20	100%	20,00	*55%*	*11*	*30%*
	100%	29	100%	29,00	*60%*	*17,4*	*30%*
	92%	35	100%	35,00	*40%*	*14*	*30%*
	100%	22	100%	22,00	30%	*6,6*	*60%*
	50%	12	100%	12,00	*20%*	*2,4*	*80%*
	82%	14	100%	14,00	*18%*	*2,52*	*18%*
	100%	16	100%	16,00	*28%*	*4,48*	*72%*
	92%	22	100%	22,00	*30%*	*6,6*	*30%*
	100%	26	100%	26,00	*40%*	*10,4*	*10%*
	100%	26	100%	26,00	*40%*	*10,4*	*30%*
	90%	35,1	100%	35,10	*0%*	*0*	*0%*
	100%	17	100%	17,00	*20%*	*3,4*	*40%*
18	56%	0	80%	-	*100%*	*0*	*0%*
	68%	15	100%	15,00	*43%*	*6,428571429*	*0%*
	85%	23	48%	11,00	*20%*	*2,2*	*0%*
	100%	21	100%	21,00	*43%*	*9*	*32%*
	0%	0	0%	-	*0%*		*0%*
	74%	17,02	100%	17,02	*80%*	*13,616*	*20%*
	81%	13	100%	13,00	*38%*	*5*	*50%*
7	29%	0	100%	-	*0%*	*0*	*100%*
22	41%	17,59090909	44%	7,82	*100%*	*7,818181818*	*0%*
	100%	50	77%	38,50	*0%*	*0*	*0%*
	25%	9,5	100%	9,50	*65%*	*6,175*	*35%*
	100%	19	100%	19,00	*44%*	*8,3125*	*6%*
	100%	37	100%	37,00	*70%*	*25,9*	*20%*
	65%	15	100%	15,00	*40%*	*6*	*30%*
	71%	12	100%	12,00	*0%*	*0*	*0%*
	82%	15,58	100%	15,58	*60%*	*9,348*	*40%*
	100%	19	100%	19,00	*60%*	*11,4*	*30%*
	100%	29	100%	29,00	*0%*	*0*	*100%*
	100%	15	100%	15,00	*15%*	*2,25*	*85%*
	100%	92	100%	92,00	*0%*	*0*	*0%*
	0%	0	0%	-	*0%*		*0%*
	82%	14	100%	14,00	*31%*	*4,307692308*	*69%*
7	14%	0	100%	-	*100%*	*0*	*0%*
85	30%	0	100%	-	*100%*	*0*	*0%*
	93%	14	100%	14,00	*14%*	*2*	*86%*
	90%	44,1	100%	44,10	*0%*	*0*	*100%*
100+	0%	0	0%	-	*0%*		*0%*
15	20%	0	35%	-	*50%*	*0*	*50%*
42	19%	0	100%	-	*100%*	*0*	*0%*
55	0%	0	0%	-	*0%*		*0%*
	0%	0	0%	-	*0%*		*0%*
	100%	25	84%	21,00	*19%*	*4*	*81%*
	40%	21,2	100%	21,20	*100%*	*21,2*	*0%*
	100%	26	100%	26,00	30%	*7,8*	*30%*
	60%	50	88%	44,00	*40%*	*17,6*	*60%*
	7%	1,5	100%	1,50	*100%*	*1,5*	*0%*
	0%	0	0%	-	*0%*		*0%*
	47%	17,88235294	100%	17,88	*80%*	*14,30588235*	*20%*
	20%	16,2	100%	16,20	*100%*	*16,2*	*0%*
	100%	47	68%	32,00	*35%*	*11,2*	*30%*
	85%	22,95	100%	22,95	*65%*	*14,9175*	*35%*
	12%	15,84	100%	15,84	*100%*	*15,84*	*0%*
	100%	18	100%	18,00	*60%*	*10,8*	*40%*
	92%	35	46%	16,00	*0%*	*0*	*90%*

	95%	21,85	100%	21,85	*40%*	*8,74*	*50%*
	0%	0	0%	-	*0%*		*0%*
	60%	28,8	100%	28,80	*20%*	*5,76*	*30%*
	25%	0	100%	-	*100%*	*0*	*0%*
	30%	26,4	100%	26,40	*50%*	*13,2*	*50%*
	97%	60	100%	60,00	*80%*	*48*	*20%*
10	60%	0	100%	-	*100%*	*0*	*0%*
	29%	18	100%	18,00	*100%*	*18*	*0%*
70	90%	0	100%	-	*100%*	*0*	*0%*
25	40%	0	100%	-	*100%*	*0*	*0%*
20	65%	0	100%	-	*100%*	*0*	*0%*
45	65%	0	100%	-	*100%*	*0*	*0%*
	20%	12,2	100%	12,20	*75%*	*9,15*	*25%*
12	20%	0	100%	-	*100%*	*0*	*0%*
12	83%	0	100%	-	*100%*	*0*	*0%*
	100%	19	100%	19,00	*85%*	*16,15*	*15%*
160	72%	0	100%	-	*100%*	*0*	*0%*
200	50%	0	100%	-	*100%*	*0*	*0%*
12	65%	0	100%	-	*100%*	*0*	*0%*
	100%	15	100%	15,00	*50%*	*7,5*	*0%*
5	10%	0	100%	-	*50%*	*0*	*50%*
8	25%	0	100%	-	*50%*	*0*	*50%*
	55%	8,8	100%	8,80	*80%*	*7,04*	*20%*
	45%	14	100%	14,00	*85%*	*11,9*	*15%*
18	25%	0	100%	-	*60%*	*0*	*40%*
15	20%	0	100%	-	*100%*	*0*	*0%*
	60%	25,8	100%	25,80	*10%*	*2,58*	*90%*
60	12%	0	100%	-	*100%*	*0*	*0%*
50	15%	0	100%	-	*100%*	*0*	*0%*
	100%	28	100%	28,00	*59%*	*16,59259259*	*41%*
30	40%	0	100%	-	*100%*	*0*	*0%*
90	7%	0	0%	-	*0%*		*0%*
30	0%	0	0%	-	*0%*		*0%*
9	17%	0	100%	-	*100%*	*0*	*0%*
6	67%	0	100%	-	*100%*	*0*	*0%*
	81%	17	88%	14,96	*80%*	*11,968*	*20%*
18	17%	0	100%	-	*60%*	*0*	*40%*
	71%	25	100%	25,00	*68%*	*17*	*32%*
	85%	11	100%	11,00	*36%*	*4*	*64%*
	72%	27,36	70%	19,15	*100%*	*19,152*	*0%*
9	78%	0	100%	-	*85%*	*0*	*15%*
	100%	25	90%	22,50	*60%*	*13,5*	*25%*
	100%	55	93%	51,00	*25%*	*13*	*30%*
	100%	19	100%	19,00	*28%*	*5,32*	*30%*
	51%	21	100%	21,00	*81%*	*17*	*0%*
25	30%	0	100%	-	*70%*	*0*	*30%*
	100%	19	100%	19,00	*50%*	*9,5*	*50%*
	92%	23	100%	23,00	*15%*	*3,45*	*60%*
	100%	330	80%	264,00	*30%*	*79,2*	*70%*
	100%	17	100%	17,00	*59%*	*10*	*41%*
	100%	25	100%	25,00	*90%*	*22,5*	*10%*
	100%	42	100%	42,00	*77%*	*32,34*	*23%*
	100%	38	100%	38,00	*47%*	*18*	*42%*
	100%	74	100%	74,00	*43%*	*32*	*14%*
	100%	14	100%	14,00	*100%*	*14*	*0%*
	49%	17	100%	17,00	*0%*	*0*	*0%*
	10%	0	100%	-	*40%*	*0*	*60%*
	90%	61,2	100%	61,20	*21%*	*12,852*	*56%*
29	17%	0	100%	-	*100%*	*0*	*0%*
70	14%	0	100%	-	*0%*	*0*	*100%*
	18%	2,88	40%	1,15	*50%*	*0,576*	*50%*
	100%	51	100%	51,00	*0%*	*0*	*100%*
100+	20%	0	70%	-	*100%*	*0*	*0%*
100+	24%	0	75%	-	*100%*	*0*	*0%*
100+	50%	0	50%	-	*100%*	*0*	*0%*
100+	35%	0	90%	-	*100%*	*0*	*0%*

100+	80%	0	100%	-	*100%*	*0*	*0%*
100+	100%	0	85%	-	*100%*	*0*	*0%*
100+	75%	0	100%	-	*100%*	*0*	*0%*
5	40%	0	100%	-	*25%*	*0*	*75%*
100+	65%	0	40%	-	*100%*	*0*	*0%*
100+	85%	0	100%	-	*100%*	*0*	*0%*
100+	85%	0	100%	-	*100%*	*0*	*0%*
100+	70%	0	85%	-	*100%*	*0*	*0%*
100+	50%	0	100%	-	*100%*	*0*	*0%*
100+	100%	0	100%	-	*100%*	*0*	*0%*
100+	55%	0	85%	-	*100%*	*0*	*0%*
100+	30%	0	82%	-	*100%*	*0*	*0%*
100+	65%	0	70%	-	*100%*	*0*	*0%*
100+	30%	0	100%	-	*100%*	*0*	*0%*
100+	15%	0	70%	-	*100%*	*0*	*0%*
100+	20%	0	100%	-	*100%*	*0*	*0%*
100+	12%	0	100%	-	*100%*	*0*	*0%*
100+	18%	0	100%	-	*100%*	*0*	*0%*
100+	15%	0	75%	-	*100%*	*0*	*0%*
100+	30%	0	100%	-	*100%*	*0*	*0%*
100+	18%	0	0%	-	*0%*		*0%*
100+	15%	0	100%	-	*100%*	*0*	*0%*
100+	8%	0	100%	-	*100%*	*0*	*0%*
100+	25%	0	100%	-	*100%*	*0*	*0%*
60	35%	0	20%	-	*100%*	*0*	*0%*
100+	85%	0	100%	-	*100%*	*0*	*0%*
100+	60%	0	100%	-	*100%*	*0*	*0%*
100+	70%	0	70%	-	*0%*	*0*	*0%*
100+	20%	0	100%	-	*100%*	*0*	*0%*
100+	10%	0	100%	-	*100%*	*0*	*0%*
60	70%	0	40%	-	*100%*	*0*	*0%*
16	100%	0	100%	-	*100%*	*0*	*0%*
100+	90%	0	100%	-	*100%*	*0*	*0%*
100+	50%	0	100%	-	*100%*	*0*	*0%*
8	63%	0	100%	-	*100%*	*0*	*0%*
	50%	14	71%	10,00	*0%*	*0*	*0%*
	100%	31	100%	31,00	*0%*	*0*	*100%*
	0%	0	0%	-	*0%*		*0%*
	100%	74	100%	74,00	*15%*	*11,1*	*20%*
	100%	34	90%	30,60	*13%*	*4,08*	*37%*
	100%	35	50%	17,50	*85%*	*14,875*	*15%*
	38%	19	53%	10,00	*50%*	*5*	*35%*
	0%	0	0%	-	*0%*		*0%*
	8%	9	0%	-	*0%*		*0%*
	0%	0	0%	-	*0%*		*0%*
	0%	0	0%	-	*0%*		*0%*
	0%	0	0%	-	*0%*		*0%*
	45%	14,4	100%	14,40	*100%*	*14,4*	*0%*
	35%	37	100%	37,00	*70%*	*25,9*	*20%*
	10%	7,3	100%	7,30	*20%*	*1,46*	*80%*
	92%	66	96%	63,36	*16%*	*10,1376*	*0%*
	100%	63	100%	63,00	*50%*	*31,5*	*35%*
	100%	112	100%	112,00	*50%*	*56*	*20%*
	100%	24	100%	24,00	*30%*	*7,2*	*40%*
	100%	24	100%	24,00	*10%*	*2,4*	*90%*
100	25%	0	100%	-	*100%*	*0*	*0%*
	97%	40,74	100%	40,74	*30%*	*12,222*	*50%*
	100%	96	80%	76,80	*100%*	*76,8*	*0%*
	0%	0	0%	-	*0%*		*0%*
14	0%	0	0%	-	*0%*		*0%*
	85%	22	100%	22,00	*80%*	*17,6*	*20%*
25	28%	0	100%	-	*60%*	*0*	*40%*
36	40%	0	100%	-	*85%*	*0*	*15%*
	100%	27	100%	27,00	*65%*	*17,55*	*35%*
	100%	16	100%	16,00	*10%*	*1,6*	*0%*
	59%	27	100%	27,00	*30%*	*8,1*	*50%*

	100%	27	85%	22,95	0%	0	25%
	100%	17	100%	17,00	29%	5	71%
	7%	3,43	0%	-	0%		0%
	100%	19	100%	19,00	0%	0	58%
	100%	57	88%	50,00	55%	27,5	15%
36	28%	0	60%	-	100%	0	0%
	96%	82,56	85%	70,18	100%	70,176	0%
	30%	12	100%	12,00	100%	12	0%
100+	70%	0	100%	-	100%	0	0%
	12%	7	100%	7,00	50%	3,5	50%
	20%	5,6	100%	5,60	30%	1,68	70%
34	10%	0	100%	-	100%	0	0%
	19%	24	100%	24,00	100%	24	0%
70	40%	0	100%	-	80%	0	20%
100	4%	0	100%	-	100%	0	0%
	65%	52,65	100%	52,65	18%	9,477	74%
	100%	30	100%	30,00	55%	16,5	35%
	0%	0	0%	-	0%		0%
	0%	0	0%	-	0%		0%
	100%	32	100%	32,00	20%	6,4	80%
	0%	0	0%	-	0%		0%
	0%	0	0%	-	0%		0%
	0%	0	0%	-	0%		0%
	84%	27,72	100%	27,72	16%	4,4352	84%
100	5%	0	0%	-	0%		0%
20	0%	0	0%	-	0%		0%
100+	65%	0	100%	-	100%	0	0%
	100%	56	100%	56,00	5%	2,8	65%
	100%	41	96%	39,36	15%	5,904	50%
	100%	16	100%	16,00	13%	2	60%
	100%	18	100%	18,00	20%	3,6	40%
22	18%	0	100%	-	50%	0	50%
	90%	17,1	100%	17,10	100%	17,1	0%
	75%	12	100%	12,00	75%	9	25%
	64%	14	100%	14,00	0%	0	100%
	65%	33	100%	33,00	15%	4,95	85%
	70%	108,5	100%	108,50	100%	108,5	0%
	0%	0	0%	-	0%		0%
	21%	34	100%	34,00	80%	27,2	0%
	20%	40	100%	40,00	100%	40	0%
	12%	8,4	100%	8,40	100%	8,4	0%
	18%	14,94	100%	14,94	100%	14,94	0%
	18%	8,1	100%	8,10	100%	8,1	0%
	35%	14	75%	10,50	100%	10,5	0%
	18%	6,48	100%	6,48	100%	6,48	0%
	45%	18,45	100%	18,45	100%	18,45	0%
	12%	3	100%	3,00	100%	3	0%
	88%	54,56	100%	54,56	0%	0	10%
	30%	5,7	100%	5,70	100%	5,7	0%
	100%	66	100%	66,00	70%	46,2	30%
	5%	5,45	100%	5,45	100%	5,45	0%
	84%	42,84	100%	42,84	15%	6,426	60%
	100%	50	100%	50,00	40%	20	60%
	32%	60	100%	60,00	70%	42	30%
	0%	0	0%	-	0%		0%
28	15%	0	100%	-	100%	0	0%
	100%	26	100%	26,00	38%	9,88	18%
25	24%	0	100%	-	100%	0	0%
70	40%	0	100%	-	100%	0	0%
14	0%	0	0%	-	0%		0%
20	60%	0	100%	-	100%	0	0%
	94%	67,68	100%	67,68	25%	16,92	15%
	0%	0	0%	-	0%		0%
	15%	4	100%	4,00	100%	4	0%
	83%	0	100%	-	100%	0	0%
	100%	14	100%	14,00	0%	0	70%

	100%	22	100%		22,00	*10%*	*2,2*	*40%*
50	80%	0	100%		-	*20%*	*0*	*80%*
60	0%	0	0%		-	*0%*		*0%*
	92%	87,4	100%		87,40	*5%*	*4,37*	*15%*
	85%	33,15	100%		33,15	*80%*	*26,52*	*20%*
25	0%	0	0%		-	*0%*		*0%*
35	24%	0	100%		-	*0%*	*0*	*25%*
	100%	48	100%		48,00	*0%*	*0*	*100%*
	11%	22,33	100%		22,33	*100%*	*22,33*	*0%*
70	65%	0	70%		-	*100%*	*0*	*0%*
	0%	0	0%		-	*0%*		*0%*
	14%	6	100%		6,00	*100%*	*6*	*0%*
	39%	91	100%		91,00	*70%*	*63,7*	*20%*
	23%	6,44	100%		6,44	*100%*	*6,44*	*0%*
	14%	34,86	100%		34,86	*100%*	*34,86*	*0%*
	17%	23	100%		23,00	*50%*	*11,5*	*50%*
	100%	39	80%		31,20	*20%*	*6,24*	*72%*
	100%	59	100%		59,00	*30%*	*17,7*	*40%*
	60%	28	75%		21,00	*10%*	*2,1*	*90%*
	100%	73	100%		73,00	*0%*	*0*	*22%*
	14%	16	100%		16,00	*100%*	*16*	*0%*
	38%	97	58%		56,00	*100%*	*56*	*0%*
	0%	0	0%		-	*0%*		*0%*
	32%	36	100%		36,00	*40%*	*14,4*	*30%*
90	60%	0	100%	60%	-	*100%*	*0*	*0%*
	18%	5,4	100%	18%	5,40	*45%*	*2,43*	*55%*
	0%	0	0%		-	*0%*		*0%*
	7%	17,5	100%	7%	17,50	*100%*	*17,5*	*0%*
	30%	5,7	85%	26%	4,85	*40%*	*1,938*	*60%*
50	12%	0	5%	1%	-	*100%*	*0*	*0%*
	85%	23,8	84%	71%	19,99	*100%*	*19,992*	*0%*
	18%	11	100%	18%	11,00	*100%*	*11*	*0%*
	0%	0	0%		-	*0%*		*0%*
95	0%	0	0%		-	*0%*		*0%*
	0%	0	0%		-	*0%*		*0%*
65	12%	0	100%	12%	-	*20%*	*0*	*80%*
	0%	0	0%		-	*0%*		*0%*
	0%	0	0%		-	*0%*		*0%*
	0%	0	0%		-	*0%*		*0%*
	0%	0	0%		-	*0%*		*0%*
	10%	7,3	0%		-	*0%*		*0%*
	0%	0	0%		-	*0%*		*0%*
	0%	0	0%		-	*0%*		*0%*
100+	0%	0	0%		-	*0%*		*0%*
	100%	19	100%	100%	19,00	*100%*	*19*	*0%*
	34%	12	100%	34%	12,00	*40%*	*4,8*	*0%*
75	0%	0	0%		-	*0%*		*0%*
	10%	17	100%	10%	17,00	*0%*	*0*	*0%*
	0%	0	0%		-	*0%*		*0%*
	31%	15	45%	14%	6,75	*0%*	*0*	*0%*
	38%	57	84%	32%	47,88	*0%*	*0*	*30%*
	44%	12	100%	44%	12,00	*0%*	*0*	*30%*
35	35%	0	100%	35%	-	*100%*	*0*	*0%*
	35%	6	100%	35%	6,00	*30%*	*1,8*	*70%*
	38%	11	28%	11%	3,08	*100%*	*3,08*	*0%*
	12%	5	100%	12%	5,00	*30%*	*1,5*	*70%*
	39%	7	100%	39%	7,00	*75%*	*5,25*	*25%*
	80%	32,8	100%	80%	32,80	*60%*	*19,68*	*30%*
	36%	34	100%	36%	34,00	*100%*	*34*	*0%*
	65%	15	100%	65%	15,00	*60%*	*9*	*40%*
	25%	7,75	100%	25%	7,75	*100%*	*7,75*	*0%*
	0%	0	0%		-	*0%*		*0%*
	100%	36	100%	100%	36,00	*8%*	*3*	*92%*
	54%	11,88	100%	54%	11,88	*25%*	*2,97*	*75%*
	68%	63	65%	44%	40,95	*25%*	*10,2375*	*55%*
	100%	73	100%	100%	73,00	*0%*	*0*	*20%*

100%	86	100%	100%	86,00	*30%*	*25,8*	*40%*
40%	19,6	100%	40%	19,60	*100%*	*19,6*	*0%*
35%	17,5	100%	35%	17,50	*100%*	*17,5*	*0%*
85%	46,75	100%	85%	46,75	*0%*	*0*	*0%*
100%	66	100%	100%	66,00	*33%*	*21,78*	*67%*
15%	6,9	100%	15%	6,90	*100%*	*6,9*	*0%*
45%	49	100%	45%	49,00	*30%*	*14,7*	*20%*
100%	46	100%	100%	46,00	*0%*	*0*	*0%*
100%	47	100%	100%	47,00	*7%*	*3,29*	*80%*
0%	0	0%		-	*0%*		*0%*
40%	15,6	18%	7%	2,81	*0%*	*0*	*100%*
100%	15	100%	100%	15,00	*53%*	*8*	*0%*
100%	28	100%	100%	28,00	*46%*	*13*	*24%*
62%	18	100%	62%	18,00	*0%*	*0*	*100%*
94%	54,52	100%	94%	54,52	*10%*	*5,452*	*6%*
0%	0	0%		-	*0%*		*0%*
100%	35	100%	100%	35,00	*37%*	*13*	*63%*
100%	43	100%	100%	43,00	*0%*	*0*	*100%*
100%	32	81%	81%	26,00	*0%*	*0*	*100%*
62%	32	100%	62%	32,00	*0%*	*0*	*100%*
11%	5	0%		-	*0%*		*0%*
100%	47	100%	100%	47,00	*0%*	*0*	*50%*
100%	48	100%	100%	48,00	*100%*	*48*	*0%*
0%	0	0%		-	*0%*		*0%*
50%	16,5	100%	50%	16,50	*0%*	*0*	*100%*
100%	14	100%	100%	14,00	*0%*	*0*	*100%*

in cm	% Coral wins	in cm		Amphiroa % of interaction	Total length in interaction with Amphiroa	% Algae win	% Neutral	% Coral wins
0	100%		57,20	0%	-	0%	0%	0%
0	0%	0		8%	-	0%	100%	0%
0	0%	0		0%	-	0%	0%	0%
2,4	0%	0		0%	-	0%	0%	0%
	0%			0%	-	0%	0%	0%
	0%			0%	-	0%	0%	0%
2,7	0%	0		0%	-	0%	0%	0%
0	0%	0		0%	-	0%	0%	0%
3,99	0%	0		0%	-	0%	0%	0%
16,416	30%	12,312		0%	-	0%	0%	0%
6,4	40%	6,4		0%	-	0%	0%	0%
2,24	0%	0		0%	-	0%	0%	0%
9	20%	6		0%	-	0%	0%	0%
8,99	0%	0		0%	-	0%	0%	0%
0	0%	0		0%	-	0%	0%	0%
0	0%	0		0%	-	0%	0%	0%
0	10%	22,68		0%	-	0%	0%	0%
0	30%	5,7		0%	-	0%	0%	0%
4,347	40%	4,968		0%	-	0%	0%	0%
1,536	30%	4,608		0%	-	0%	0%	0%
4,32	70%	10,08		0%	-	0%	0%	0%
0	0%	0		0%	-	0%	0%	0%
0	55%	3,9325		0%	-	0%	0%	0%
2,8	0%	0		0%	-	0%	0%	0%
2,184	60%	3,276		0%	-	0%	0%	0%
3,92	0%	0		0%	-	0%	0%	0%
8,4	40%	8,4		0%	-	0%	0%	0%
3,96	60%	5,94		0%	-	0%	0%	0%
9,04	15%	5,424		0%	-	0%	0%	0%
12,32	0%	0		10%	7,70	70%	30%	0%
9,5	20%	3,8		0%	-	0%	0%	0%
7,2	65%	23,4		0%	-	0%	0%	0%
8,14	30%	6,105		0%	-	0%	0%	0%
7,137	0%	0		0%	-	0%	0%	0%
2,72	0%	0		0%	-	0%	0%	0%
5,22	0%	0		0%	-	0%	0%	0%
9,75	0%	0		0%	-	0%	0%	0%
2,4	50%	6		0%	-	0%	0%	0%
44,625	20%	17,85		0%	-	0%	0%	0%
8,748	0%	0		0%	-	0%	0%	0%
0	50%	38,94		0%	-	0%	0%	0%
0	0%	0		0%	-	0%	0%	0%
1,68	0%	0		0%	-	0%	0%	0%
17,82	25%	8,91		0%	-	0%	0%	0%
0	100%	12,24		0%	-	0%	0%	0%
3,06	25%	3,06		0%	-	0%	0%	0%
0	0%	0		0%	-	0%	0%	0%
20	70%	56		0%	-	0%	0%	0%
0	0%	0		0%	-	0%	0%	0%
16	0%	0		0%	-	0%	0%	0%
16,15	0%	0		0%	-	0%	0%	0%
4,446	50%	5,5575		0%	-	0%	0%	0%
14,56	20%	14,56		0%	-	0%	0%	0%
4,4	55%	9,68		0%	-	0%	0%	0%
3,807	70%	26,649		0%	-	0%	0%	0%
4,212	0%	0		0%	-	0%	0%	0%
6,8	10%	1,7		0%	-	0%	0%	0%
4,44125	50%	22,20625		0%	-	0%	0%	0%
8	0%	0		0%	-	0%	0%	0%
6,994375	15%	4,196625		5%	1,47	100%	0%	0%
18,6	0%	0		0%	-	0%	0%	0%
29,07	20%	12,92		0%	-	0%	0%	0%

3,2	45%	7,2	0%	-	0%	0%	0%
4,25	65%	11,05	0%	-	0%	0%	0%
3,3	30%	3,3	0%	-	0%	0%	0%
4,8	0%	0	0%	-	0%	0%	0%
14,4	27%	19,44	0%	-	0%	0%	0%
11,34	30%	4,86	0%	-	0%	0%	0%
9,8685	10%	1,64475	10%	3,66	40%	60%	0%
8,55	40%	6,84	20%	5,70	15%	70%	15%
8,4	0%	0	15%	1,68	0%	100%	0%
6,72	60%	26,88	0%	-	0%	0%	0%
9,52	10%	1,19	0%	-	0%	0%	0%
10	30%	7,5	0%	-	0%	0%	0%
0	0%	0	0%	-	0%	0%	0%
7,2	0%	0	0%	-	0%	0%	0%
43,68	40%	29,12	0%	-	0%	0%	0%
12,285	30%	7,371	0%	-	0%	0%	0%
	0%		55%	5,12	0%	100%	0%
7,7175	0%	0	30%	3,68	0%	100%	0%
14,4	0%	0	40%	9,60	30%	70%	0%
9,18	40%	8,16	0%	-	0%	0%	0%
1,9	0%	0	40%	7,60	100%	0%	0%
12,6	30%	5,4	0%	-	0%	0%	0%
0	0%	0	40%	2,88	0%	100%	0%
0	0%	0	90%	1,73	0%	100%	0%
	0%		32%	1,23	0%	100%	0%
4,104	0%	0	0%	-	0%	0%	0%
1,33	90%	11,97	0%	-	0%	0%	0%
	0%		0%	-	0%	0%	0%
1,1931	0%	0	55%	4,51	0%	100%	0%
0	0%	0	0%	-	0%	0%	0%
	0%		0%	-	0%	0%	0%
	0%		0%	-	0%	0%	0%
	0%		0%	-	0%	0%	0%
7,371	15%	1,701	15%	2,43	30%	70%	0%
6,5	30%	3,9	0%	-	0%	0%	0%
1	0%	0	0%	-	0%	0%	0%
0	0%	0	0%	-	0%	0%	0%
1,92	0%	0	40%	1,28	20%	80%	0%
10,773	0%	0	0%	-	0%	0%	0%
	0%		100%	22,40	20%	80%	0%
0	20%	3,19	45%	13,05	100%	0%	0%
	0%		0%	-	0%	0%	0%
0	0%	0	65%	2,34	100%	0%	0%
1,08	0%	0	20%	2,16	0%	0%	100%
0	60%	4,59	0%	-	0%	0%	0%
7,6	0%	0	0%	-	0%	0%	0%
0	0%	0	0%	-	0%	0%	0%
0	20%	0	0%	-	0%	0%	0%
0	30%	0	30%	-	70%	0%	30%
0	20%	0	0%	-	0%	0%	0%
3,04	0%	0	0%	-	0%	0%	0%
3,87	0%	0	0%	-	0%	0%	0%
5,278	0%	0	0%	-	0%	0%	0%
5,184	0%	0	0%	-	0%	0%	0%
2,96	0%	0	0%	-	0%	0%	0%
10,34	0%	0	0%	-	0%	0%	0%
14,4925	0%	0	0%	-	0%	0%	0%
3,6	0%	0	0%	-	0%	0%	0%
4,8	20%	3,2	0%	-	0%	0%	0%
15,3	10%	5,1	0%	-	0%	0%	0%
16,8625	60%	40,47	0%	-	0%	0%	0%
10	50%	12,5	0%	-	0%	0%	0%
	0%		0%	-	0%	0%	0%
1,08	0%	0	0%	-	0%	0%	0%
	0%		0%	-	0%	0%	0%
	0%		0%	-	0%	0%	0%

9	0%	0	0%	-	0%	0%	0%
6,3	0%	0	0%	-	0%	0%	0%
	0%		0%	-	0%	0%	0%
	0%		0%	-	0%	0%	0%
6,0673	40%	5,644	0%	-	0%	0%	0%
9,87	34%	16	0%	-	0%	0%	0%
14	0%	0	0%	-	0%	0%	0%
	0%		0%	-	0%	0%	0%
	0%		0%	-	0%	0%	0%
	0%		0%	-	0%	0%	0%
0	0%	0	0%	-	0%	0%	0%
	0%		0%	-	0%	0%	0%
3,915	0%	0	0%	-	0%	0%	0%
29	0%	0	0%	-	0%	0%	0%
5,8	40%	11,6	0%	-	0%	0%	0%
1,1	80%	4,4	0%	-	0%	0%	0%
9,3	20%	3,72	0%	-	0%	0%	0%
	0%		0%	-	0%	0%	0%
	0%		0%	-	0%	0%	0%
3,5	15%	2,1	0%	-	0%	0%	0%
	0%		0%	-	0%	0%	0%
12	0%	0	0%	-	0%	0%	0%
1,71	65%	7,41	0%	-	0%	0%	0%
4,9	0%	0	0%	-	0%	0%	0%
	0%		0%	-	0%	0%	0%
	0%		0%	-	0%	0%	0%
	0%		0%	-	0%	0%	0%
1,5925	0%	0	0%	-	0%	0%	0%
2,7	0%	0	0%	-	0%	0%	0%
4,55625	0%	0	10%	0,68	0%	100%	0%
8,67	0%	0	0%	-	0%	0%	0%
3,608	0%	0	0%	-	0%	0%	0%
3,096	0%	0	0%	-	0%	0%	0%
1,9995	0%	0	0%	-	0%	0%	0%
	0%		0%	-	0%	0%	0%
	0%		0%	-	0%	0%	0%
	0%		100%	5,88	80%	20%	0%
1,11	0%	0	0%	-	0%	0%	0%
0	0%	0	0%	-	0%	0%	0%
4,1106	0%	0	0%	-	0%	0%	0%
0	0%	0	0%	-	0%	0%	0%
0	0%	0	10%	-	80%	20%	0%
	0%		50%	-	0%	100%	0%
1,2	0%	0	60%	7,20	75%	25%	0%
1,68	0%	0	20%	2,10	35%	65%	0%
2,052	0%	0	80%	13,68	90%	10%	0%
0	40%	28,88	0%	-	0%	0%	0%
17,55	0%	0	10%	3,90	100%	0%	0%
7,29	20%	4,86	0%	-	0%	0%	0%
4	0%	0	0%	-	0%	0%	0%
16,74	10%	2,79	0%	-	0%	0%	0%
4,62	10%	0,77	0%	-	0%	0%	0%
2,31	10%	0,77	0%	-	0%	0%	0%
0	80%	38,76	10%	5,70	0%	0%	100%
5,538	90%	49,842	7%	5,46	60%	0%	40%
0	90%	54,27	0%	-	0%	0%	0%
1,54	40%	3,08	0%	-	0%	0%	0%
5,4	70%	12,6	0%	-	0%	0%	0%
2,34	50%	2,925	0%	-	0%	0%	0%
0	0%	0	0%	-	0%	0%	0%
0	0%	0	0%	-	0%	0%	0%
12,8	0%	0	60%	24,00	20%	30%	50%
5,3375	40%	6,1	0%	-	0%	0%	0%
	0%		0%	-	0%	0%	0%
0	0%	0	0%	-	0%	0%	0%
15,04	50%	18,8	0%	-	0%	0%	0%

	0%		0%	-	0%	0%	0%
4,2	0%	0	0%	-	0%	0%	0%
7,02	35%	8,19	0%	-	0%	0%	0%
3,6	40%	3,6	0%	-	0%	0%	0%
3,045	10%	0,87	0%	-	0%	0%	0%
7,125	15%	4,275	0%	-	0%	0%	0%
7,2	5%	1,2	0%	-	0%	0%	0%
0	80%	25,6	16%	6,08	50%	0%	50%
1,4	0%	0	0%	-	0%	0%	0%
4,8	20%	2,4	0%	-	0%	0%	0%
20,24	0%	0	10%	10,12	15%	70%	15%
1,64	10%	0,328	0%	-	0%	0%	0%
0	90%	19,8	0%	-	0%	0%	0%
	0%		0%	-	0%	0%	0%
	0%		0%	-	0%	0%	0%
0	0%	0	0%	-	0%	0%	0%
0	0%	0	0%	-	0%	0%	0%
0	0%	0	0%	-	0%	0%	0%
	0%		0%	-	0%	0%	0%
	0%		0%	-	0%	0%	0%
7,2	20%	2,4	0%	-	0%	0%	0%
0	50%	37,5	0%	-	0%	0%	0%
0	70%	10,5	0%	-	0%	0%	0%
15,876	0%	0	0%	-	0%	0%	0%
4,2	0%	0	0%	-	0%	0%	0%
0	0%	0	0%	-	0%	0%	0%
0	0%	0	0%	-	0%	0%	0%
0	0%	0	0%	-	0%	0%	0%
3,42	0%	0	0%	-	0%	0%	0%
0,8925	0%	0	0%	-	0%	0%	0%
0	0%	0	0%	-	0%	0%	0%
7,74	15%	5,805	0%	-	0%	0%	0%
	0%		0%	-	0%	0%	0%
0	0%	0	0%	-	0%	0%	0%
6,3	15%	6,3	20%	12,00	60%	40%	0%
15,4	0%	0	0%	-	0%	0%	0%
34,08	0%	0	0%	-	0%	0%	0%
16,9	0%	0	0%	-	0%	0%	0%
26,712	0%	0	10%	4,77	0%	100%	0%
6	0%	0	45%	4,95	0%	100%	0%
0,15	0%	0	0%	-	0%	0%	0%
	0%		50%	9,08	0%	65%	35%
11,088	10%	2,772	0%	-	0%	0%	0%
9,24	0%	0	0%	-	0%	0%	0%
18,7	15%	5,1	0%	-	0%	0%	0%
11,2	40%	11,2	0%	-	0%	0%	0%
25,44	0%	0	0%	-	0%	0%	0%
1,04	0%	0	0%	-	0%	0%	0%
3,63825	20%	1,0395	0%	-	0%	0%	0%
4,6648	0%	0	0%	-	0%	0%	0%
2,47	0%	0	0%	-	0%	0%	0%
3,0315	0%	0	0%	-	0%	0%	0%
5,5	0%	0	0%	-	0%	0%	0%
1,28	0%	0	0%	-	0%	0%	0%
30,51	10%	4,068	0%	-	0%	0%	0%
0	0%	0	0%	-	0%	0%	0%
7,2	0%	0	0%	-	0%	0%	0%
9,46	0%	0	0%	-	0%	0%	0%
25,8	0%	0	0%	-	0%	0%	0%
4,6	0%	0	0%	-	0%	0%	0%
2,72	0%	0	0%	-	0%	0%	0%
15,3	0%	0	0%	-	0%	0%	0%
4,05	0%	0	0%	-	0%	0%	0%
4,1	0%	0	0%	-	0%	0%	0%
4,18	0%	0	0%	-	0%	0%	0%
16,428	35%	9,583	0%	-	0%	0%	0%

2,52	0%	0	0%	-	0%	0%	0%
4,5056	13%	0,9152	0%	-	0%	0%	0%
5,28	38%	4,56	0%	-	0%	0%	0%
4,29	30%	2,145	0%	-	0%	0%	0%
23,1	0%	0	0%	-	0%	0%	0%
43,16	0%	0	0%	-	0%	0%	0%
5,4675	25%	3,0375	0%	-	0%	0%	0%
8,96	0%	0	0%	-	0%	0%	0%
24,64	0%	0	0%	-	0%	0%	0%
12,48	20%	3,12	0%	-	0%	0%	0%
1,232	0%	0	0%	-	0%	0%	0%
4,16	8%	1,04	0%	-	0%	0%	0%
0,72	0%	0	0%	-	0%	0%	0%
10	0%	0	0%	-	0%	0%	0%
	0%		0%	-	0%	0%	0%
0	0%	0	0%	-	0%	0%	0%
33,6	0%	0	0%	-	0%	0%	0%
15,4	0%	0	0%	-	0%	0%	0%
5,712	0%	0	0%	-	0%	0%	0%
0	0%	0	0%	-	0%	0%	0%
0	0%	0	0%	-	0%	0%	0%
4,98	0%	0	0%	-	0%	0%	0%
19,968	4%	1,248	0%	-	0%	0%	0%
16,236	10%	3,608	0%	-	0%	0%	0%
9	0%	0	0%	-	0%	0%	0%
5	10%	1	0%	-	0%	0%	0%
1,76	38%	6	0%	-	0%	0%	0%
6,448	20%	1,612	0%	-	0%	0%	0%
24,192	0%	0	0%	-	0%	0%	0%
4,4	0%	0	0%	-	0%	0%	0%
7,28	0%	0	0%	-	0%	0%	0%
8	0%	0	0%	-	0%	0%	0%
	0%		0%	-	0%	0%	0%
3,312	0%	0	0%	-	0%	0%	0%
0,55	0%	0	0%	-	0%	0%	0%
9,75	10%	1,3	0%	-	0%	0%	0%
14,55	0%	0	0%	-	0%	0%	0%
2,94	0%	0	0%	-	0%	0%	0%
13,3875	15%	5,7375	0%	-	0%	0%	0%
2,59	0%	0	0%	-	0%	0%	0%
15,54	0%	0	0%	-	0%	0%	0%
7,2	0%	0	0%	-	0%	0%	0%
6,76	0%	0	0%	-	0%	0%	0%
2,85	0%	0	0%	-	0%	0%	0%
6,6	0%	0	0%	-	0%	0%	0%
7,5	0%	0	0%	-	0%	0%	0%
11,55	40%	9,24	0%	-	0%	0%	0%
5,2	10%	0,8	0%	-	0%	0%	0%
5,6	15%	2,1	0%	-	0%	0%	0%
3,136	0%	0	0%	-	0%	0%	0%
6,118	10%	0,874	0%	-	0%	0%	0%
0,84	0%	0	0%	-	0%	0%	0%
9,4164	29%	4,6284	0%	-	0%	0%	0%
4,2	20%	2,8	0%	-	0%	0%	0%
2,112	0%	0	0%	-	0%	0%	0%
3,7191	0%	0	0%	-	0%	0%	0%
3,5264	18%	3,17376	0%	-	0%	0%	0%
4,928	20%	4,928	0%	-	0%	0%	0%
20,4	0%	0	0%	-	0%	0%	0%
7,29	0%	0	0%	-	0%	0%	0%
2,2275	65%	5,7915	0%	-	0%	0%	0%
11,16	55%	13,64	0%	-	0%	0%	0%
7,8	35%	5,46	0%	-	0%	0%	0%
6,24	0%	0	0%	-	0%	0%	0%
0,714	0%	0	0%	-	0%	0%	0%
	0%		0%	-	0%	0%	0%

	0%		0%	-	0%	0%	0%
9	45%	16,2	0%	-	0%	0%	0%
0	0%	0	0%	-	0%	0%	0%
1,848	30%	1,386	0%	-	0%	0%	0%
6,84	0%	0	0%	-	0%	0%	0%
20,4	5%	1,2	0%	-	0%	0%	0%
9,88	85%	83,98	0%	-	0%	0%	0%
12,74	0%	0	0%	-	0%	0%	0%
7,5	10%	2,5	0%	-	0%	0%	0%
3,0856	0%	0	0%	-	0%	0%	0%
33,5	25%	16,75	0%	-	0%	0%	0%
10,5	20%	4,2	0%	-	0%	0%	0%
8,8	60%	52,8	0%	-	0%	0%	0%
27,09	0%	0	0%	-	0%	0%	0%
22,464	0%	0	0%	-	0%	0%	0%
11,2	25%	7	0%	-	0%	0%	0%
8	0%	0	0%	-	0%	0%	0%
4,65	0%	0	0%	-	0%	0%	0%
11,904	0%	0	0%	-	0%	0%	0%
15	0%	0	0%	-	0%	0%	0%
9,945	0%	0	0%	-	0%	0%	0%
3,36	0%	0	0%	-	0%	0%	0%
9,36	10%	1,44	0%	-	0%	0%	0%
11,05	0%	0	0%	-	0%	0%	0%
2,375	35%	3,325	0%	-	0%	0%	0%
6,3	30%	3,78	0%	-	0%	0%	0%
13,8	10%	2,3	0%	-	0%	0%	0%
18,9	0%	0	0%	-	0%	0%	0%
14,4	10%	4,8	0%	-	0%	0%	0%
0	0%	0	0%	-	0%	0%	0%
8,8	35%	6,16	0%	-	0%	0%	0%
0	100%	71	0%	-	0%	0%	0%
11,7936	20%	3,3696	0%	-	0%	0%	0%
1,3125	0%	0	0%	-	0%	0%	0%
0	100%	26,68	0%	-	0%	0%	0%
7,6	0%	0	0%	-	0%	0%	0%
5,39	10%	1,54	0%	-	0%	0%	0%
10,53	50%	17,55	0%	-	0%	0%	0%
20,368125	15%	6,789375	0%	-	0%	0%	0%
3,185	0%	0	0%	-	0%	0%	0%
0	0%	0	0%	-	0%	0%	0%
7,05	0%	0	0%	-	0%	0%	0%
4,25	10%	1,7	0%	-	0%	0%	0%
16,02	80%	64,08	0%	-	0%	0%	0%
6,909	0%	0	0%	-	0%	0%	0%
7,44	0%	0	0%	-	0%	0%	0%
7,2	15%	4,32	0%	-	0%	0%	0%
12,54825	20%	4,563	35%	12,29	100%	0%	0%
12,58	0%	0	0%	-	0%	0%	0%
20,4	0%	0	0%	-	0%	0%	0%
4,488	10%	1,496	15%	4,08	50%	50%	0%
0	100%	60,3	0%	-	0%	0%	0%
18,36	0%	0	0%	-	0%	0%	0%
7,84	0%	0	0%	-	0%	0%	0%
9,6	0%	0	0%	-	0%	0%	0%
0	0%	0	0%	-	0%	0%	0%
17,4	40%	11,6	0%	-	0%	0%	0%
0	100%	17,6	0%	-	0%	0%	0%
11,7	0%	0	0%	-	0%	0%	0%
0	0%	0	0%	-	0%	0%	0%
8,91	50%	14,85	0%	-	0%	0%	0%
4,95	0%	0	0%	-	0%	0%	0%
9,84	50%	16,4	0%	-	0%	0%	0%
9,6	0%	0	0%	-	0%	0%	0%
7,8	30%	3,9	0%	-	0%	0%	0%
7,84	0%	0	0%	-	0%	0%	0%

0,72	0%	0	0%	-	0%	0%	0%
0	0%	0	0%	-	0%	0%	0%
0	0%	0	0%	-	0%	0%	0%
2,55	0%	0	0%	-	0%	0%	0%
2	0%	0	0%	-	0%	0%	0%
11,16	40%	11,16	0%	-	0%	0%	0%
14	0%	0	0%	-	0%	0%	0%
45	0%	0	0%	-	0%	0%	0%
0	0%	0	0%	-	0%	0%	0%
	0%		0%	-	0%	0%	0%
	0%		0%	-	0%	0%	0%
0	0%	0	0%	-	0%	0%	0%
0	0%	0	0%	-	0%	0%	0%
2,08	50%	5,2	0%	-	0%	0%	0%
	0%		0%	-	0%	0%	0%
1,575	0%	0	0%	-	0%	0%	0%
4,8	0%	0	0%	-	0%	0%	0%
0	0%	0	0%	-	0%	0%	0%
3,9	70%	9,1	0%	-	0%	0%	0%
1,02	0%	0	0%	-	0%	0%	0%
1,04	40%	2,08	0%	-	0%	0%	0%
3,0492	0%	0	0%	-	0%	0%	0%
0	0%	0	0%	-	0%	0%	0%
1,75	0%	0	0%	-	0%	0%	0%
0	0%	0	0%	-	0%	0%	0%
3,255	85%	18,445	0%	-	0%	0%	0%
4,928	65%	9,152	0%	-	0%	0%	0%
0	0%	0	0%	-	0%	0%	0%
12	30%	6	0%	-	0%	0%	0%
7,2	0%	0	0%	-	0%	0%	0%
7,5	10%	1,5	0%	-	0%	0%	0%
0	70%	25,2	0%	-	0%	0%	0%
0	0%	0	0%	-	0%	0%	0%
0	0%	0	0%	-	0%	0%	0%
4,8	25%	2	0%	-	0%	0%	0%
6,3	30%	6,3	0%	-	0%	0%	0%
	0%		0%	-	0%	0%	0%
	0%		0%	-	0%	0%	0%
0	0%	0	0%	-	0%	0%	0%
19	0%	0	0%	-	0%	0%	0%
8,99	0%	0	50%	8,99	50%	50%	0%
12,92	0%	0	0%	-	0%	0%	0%
	0%		0%	-	0%	0%	0%
	0%		0%	-	0%	0%	0%
8,64	0%	0	0%	-	0%	0%	0%
	0%		0%	-	0%	0%	0%
8,32	19%	2,47	0%	-	0%	0%	0%
0	0%	0	0%	-	0%	0%	0%
	0%		0%	-	0%	0%	0%
	0%		0%	-	0%	0%	0%
	0%		0%	-	0%	0%	0%
	0%		0%	-	0%	0%	0%
7,4	10%	2,96	0%	-	0%	0%	0%
5,2	0%	0	0%	-	0%	0%	0%
3,91	0%	0	0%	-	0%	0%	0%
0	0%	0	15%	4,50	100%	0%	0%
0	0%	0	0%	-	0%	0%	0%
15,59425	0%	0	0%	-	0%	0%	0%
7,92	80%	31,68	0%	-	0%	0%	0%
18,36	0%	0	0%	-	0%	0%	0%
12,8	0%	0	0%	-	0%	0%	0%
8,208	0%	0	20%	3,42	100%	0%	0%
5	30%	3	0%	-	0%	0%	0%
0	0%	0	0%	-	0%	0%	0%
6,65	30%	5,7	0%	-	0%	0%	0%
5,88	5%	0,98	0%	-	0%	0%	0%

0	0%	0	0%	-	0%	0%	0%
5,85	0%	0	0%	-	0%	0%	0%
0	0%	0	0%	-	0%	0%	0%
	0%		0%	-	0%	0%	0%
15	0%	0	0%	-	0%	0%	0%
6,8306	83%	33,3494	18%	9,00	100%	0%	0%
0	100%	4,0836	0%	-	0%	0%	0%
0	0%	0	0%	-	0%	0%	0%
0	0%	0	0%	-	0%	0%	0%
13,2	0%	0	0%	-	0%	0%	0%
9,35	30%	5,1	0%	-	0%	0%	0%
14,25	0%	0	0%	-	0%	0%	0%
0	0%	0	0%	-	0%	0%	0%
	0%		0%	-	0%	0%	0%
7	10%	1,4	0%	-	0%	0%	0%
15,05	0%	0	0%	-	0%	0%	0%
12,8	0%	0	0%	-	0%	0%	0%
2,55	35%	2,975	0%	-	0%	0%	0%
11,05	35%	5,95	0%	-	0%	0%	0%
6,4	21%	2,688	0%	-	0%	0%	0%
12	0%	0	0%	-	0%	0%	0%
28,8	0%	0	0%	-	0%	0%	0%
4,68	41%	5,33	0%	-	0%	0%	0%
0	70%	16	0%	-	0%	0%	0%
	0%		0%	-	0%	0%	0%
15,47	10%	4,42	15%	7,80	100%	0%	0%
32	30%	19,2	0%	-	0%	0%	0%
11,25	40%	11,25	0%	-	0%	0%	0%
4,5	50%	9	0%	-	0%	0%	0%
10,8	20%	7,2	0%	-	0%	0%	0%
19,68	40%	19,2	0%	-	0%	0%	0%
20,2725	40%	18,02	0%	-	0%	0%	0%
30	0%	0	0%	-	0%	0%	0%
23,56	9%	3,42	0%	-	0%	0%	0%
14,04	20%	4,68	0%	-	0%	0%	0%
16	0%	0	0%	-	0%	0%	0%
3,045	80%	16,24	0%	-	0%	0%	0%
0	100%	8,9	0%	-	0%	0%	0%
21,2	35%	14,84	0%	-	0%	0%	0%
30,10176	50%	37,6272	33%	37,07	75%	25%	0%
6,65	50%	9,5	0%	-	0%	0%	0%
2,9568	52%	9,6096	0%	-	0%	0%	0%
	0%		0%	-	0%	0%	0%
3,5	0%	0	0%	-	0%	0%	0%
	0%		0%	-	0%	0%	0%
6,4	60%	9,6	0%	-	0%	0%	0%
3,8	50%	9,5	0%	-	0%	0%	0%
	0%		0%	-	0%	0%	0%
11	0%	0	0%	-	0%	0%	0%
	0%		0%	-	0%	0%	0%
2	50%	3	0%	-	0%	0%	0%
	0%		0%	-	0%	0%	0%
5,4	40%	3,6	0%	-	0%	0%	0%
0	92%	51,52	0%	-	0%	0%	0%
21,692	12%	4,488	0%	-	0%	0%	0%
0	100%	35	0%	-	0%	0%	0%
0	100%	33	0%	-	0%	0%	0%
	0%		0%	-	0%	0%	0%
9,36	5%	0,585	0%	-	0%	0%	0%
18,16552	0%	0	0%	-	0%	0%	0%
20,774	16%	6,392	15%	7,00	20%	80%	0%
	0%		0%	-	0%	0%	0%
0	0%	0	0%	-	0%	0%	0%
0	0%	0	0%	-	0%	0%	0%
0	0%	0	0%	-	0%	0%	0%
	0%		0%	-	0%	0%	0%

	0%		100%	2,10	100%	0%	0%
	0%		0%	-	0%	0%	0%
	0%		0%	-	0%	0%	0%
11,2	0%	0	26%	4,94	100%	0%	0%
5,1	0%	0	0%	-	0%	0%	0%
7,8	10%	1,2	0%	-	0%	0%	0%
5,7344	0%	0	0%	-	0%	0%	0%
4,4	30%	5,28	20%	4,40	0%	100%	0%
0	92%	27,232	0%	-	0%	0%	0%
16,2	0%	0	0%	-	0%	0%	0%
	0%		0%	-	0%	0%	0%
12,92	0%	0	0%	-	0%	0%	0%
8	0%	0	0%	-	0%	0%	0%
8,8	0%	0	0%	-	0%	0%	0%
3	0%	0	0%	-	0%	0%	0%
0	0%	0	0%	-	0%	0%	0%
16	0%	0	0%	-	0%	0%	0%
	0%		0%	-	0%	0%	0%
0	0%	0	0%	-	0%	0%	0%
0	0%	0	0%	-	0%	0%	0%
0	0%	0	0%	-	0%	0%	0%
4,7125	0%	0	28%	8,00	100%	0%	0%
	0%		0%	-	0%	0%	0%
5,04	33%	6	0%	-	0%	0%	0%
8,1	15%	2,7	0%	-	0%	0%	0%
12,5	0%	0	0%	-	0%	0%	0%
3,3	0%	0	0%	-	0%	0%	0%
0	56%	8,96	0%	-	0%	0%	0%
	0%		0%	-	0%	0%	0%
9	8%	1,44	0%	-	0%	0%	0%
27,9	0%	0	0%	-	0%	0%	0%
3,9	40%	3,9	0%	-	0%	0%	0%
	0%		0%	-	0%	0%	0%
4,83	30%	4,83	0%	-	0%	0%	0%
0	0%	0	0%	-	0%	0%	0%
	0%		0%	-	0%	0%	0%
2,8	0%	0	0%	-	0%	0%	0%
9,8	0%	0	0%	-	0%	0%	0%
0	0%	0	0%	-	0%	0%	0%
3,9	0%	0	0%	-	0%	0%	0%
5	65%	13	0%	-	0%	0%	0%
6,2169	60%	18,6507	31%	14,02	75%	25%	0%
4,4	65%	14,3	0%	-	0%	0%	0%
	0%		0%	-	0%	0%	0%
0	0%	0	0%	-	0%	0%	0%
	0%		0%	-	0%	0%	0%
	0%		0%	-	0%	0%	0%
0	0%	0	40%	-	100%	0%	0%
3,9	0%	0	0%	-	0%	0%	0%
0	0%	0	0%	-	0%	0%	0%
0	0%	0	0%	-	0%	0%	0%
	0%		100%	-	40%	60%	0%
0	0%	0	0%	-	0%	0%	0%
0	0%	0	0%	-	0%	0%	0%
0	100%	74,921	45%	61,30	10%	0%	90%
0	0%	0	0%	-	0%	0%	0%
0	0%	0	0%	-	0%	0%	0%
0	0%	0	0%	-	0%	0%	0%
0	0%	0	0%	-	0%	0%	0%
18,088	70%	63,308	0%	-	0%	0%	0%
21	70%	49	0%	-	0%	0%	0%
0	0%	0	0%	-	0%	0%	0%
0	0%	0	0%	-	0%	0%	0%
0	0%	0	0%	-	0%	0%	0%
0	0%	0	25%	-	50%	50%	0%
0	0%	0	0%	-	0%	0%	0%

0	0%	0	65%	-	50%	50%	0%
0	0%	0	0%	-	0%	0%	0%
	0%		0%	-	0%	0%	0%
0	0%	0	0%	-	0%	0%	0%
0	0%	0	0%	-	0%	0%	0%
	0%		0%	-	0%	0%	0%
0	0%	0	0%	-	0%	0%	0%
	0%		0%	-	0%	0%	0%
7,2	0%	0	0%	-	0%	0%	0%
0	0%	0	0%	-	0%	0%	0%
0	0%	0	0%	-	0%	0%	0%
0	0%	0	0%	-	0%	0%	0%
0	0%	0	0%	-	0%	0%	0%
0	0%	0	0%	-	0%	0%	0%
0	0%	0	0%	-	0%	0%	0%
0	0%	0	0%	-	0%	0%	0%
	0%		100%	-	30%	70%	0%
0	0%	0	0%	-	0%	0%	0%
0	0%	0	0%	-	0%	0%	0%
0	0%	0	15%	-	50%	50%	0%
	0%		0%	-	0%	0%	0%
0	0%	0	0%	-	0%	0%	0%
0	0%	0	0%	-	0%	0%	0%
0	0%	0	0%	-	0%	0%	0%
0	0%	0	0%	-	0%	0%	0%
	0%		0%	-	0%	0%	0%
	0%		0%	-	0%	0%	0%
0	0%	0	0%	-	0%	0%	0%
0	0%	0	0%	-	0%	0%	0%
0	0%	0	0%	-	0%	0%	0%
0	0%	0	0%	-	0%	0%	0%
0	0%	0	0%	-	0%	0%	0%
0	0%	0	0%	-	0%	0%	0%
0	0%	0	0%	-	0%	0%	0%
	0%		0%	-	0%	0%	0%
	0%		0%	-	0%	0%	0%
0	0%	0	0%	-	0%	0%	0%
0	0%	0	0%	-	0%	0%	0%
0	0%	0	0%	-	0%	0%	0%
0	0%	0	0%	-	0%	0%	0%
0	0%	0	0%	-	0%	0%	0%
0	0%	0	0%	-	0%	0%	0%
0	0%	0	0%	-	0%	0%	0%
0	0%	0	0%	-	0%	0%	0%
	0%		0%	-	0%	0%	0%
0	0%	0	0%	-	0%	0%	0%
	0%		0%	-	0%	0%	0%
0	0%	0	0%	-	0%	0%	0%
0	0%	0	0%	-	0%	0%	0%
0	0%	0	0%	-	0%	0%	0%
0	0%	0	0%	-	0%	0%	0%
	0%		0%	-	0%	0%	0%
	0%		0%	-	0%	0%	0%
0	0%	0	0%	-	0%	0%	0%
	0%		0%	-	0%	0%	0%
0	0%	0	0%	-	0%	0%	0%
0	0%	0	0%	-	0%	0%	0%
	0%		0%	-	0%	0%	0%
0	0%	0	0%	-	0%	0%	0%
0	0%	0	0%	-	0%	0%	0%
0	0%	0	60%	-	50%	50%	0%
0	0%	0	0%	-	0%	0%	0%
0	0%	0	0%	-	0%	0%	0%
0	0%	0	0%	-	0%	0%	0%
0	0%	0	0%	-	0%	0%	0%
	0%		0%	-	0%	0%	0%

	0%		0%	-	*0%*	*0%*	*0%*
0	*0%*	*0*	0%	-	*0%*	*0%*	*0%*
0	*0%*	*0*	0%	-	*0%*	*0%*	*0%*
	0%		100%	-	*100%*	*0%*	*0%*
0	*0%*	*0*	0%	-	*0%*	*0%*	*0%*
0	*0%*	*0*	0%	-	*0%*	*0%*	*0%*
	0%		100%	-	*100%*	*0%*	*0%*
0	*0%*	*0*	0%	-	*0%*	*0%*	*0%*
0	*0%*	*0*	0%	-	*0%*	*0%*	*0%*
0	*0%*	*0*	0%	-	*0%*	*0%*	*0%*
	0%		100%	-	*100%*	*0%*	*0%*
0	*0%*	*0*	0%	-	*0%*	*0%*	*0%*
	0%		0%	-	*0%*	*0%*	*0%*
0	*0%*	*0*	0%	-	*0%*	*0%*	*0%*
0	*0%*	*0*	0%	-	*0%*	*0%*	*0%*
0	*0%*	*0*	0%	-	*0%*	*0%*	*0%*
0	*0%*	*0*	0%	-	*0%*	*0%*	*0%*
0	*0%*	*0*	0%	-	*0%*	*0%*	*0%*
0	*0%*	*0*	40%	-	*100%*	*0%*	*0%*
0	*30%*	*0*	0%	-	*0%*	*0%*	*0%*
0	*0%*	*0*	33%	-	*100%*	*0%*	*0%*
0	*0%*	*0*	0%	-	*0%*	*0%*	*0%*
0	*0%*	*0*	0%	-	*0%*	*0%*	*0%*
	0%		0%	-	*0%*	*0%*	*0%*
0	*0%*	*0*	0%	-	*0%*	*0%*	*0%*
	0%		0%	-	*0%*	*0%*	*0%*
	0%		0%	-	*0%*	*0%*	*0%*
	0%		0%	-	*0%*	*0%*	*0%*
0	*0%*	*0*	0%	-	*0%*	*0%*	*0%*
0	*0%*	*0*	0%	-	*0%*	*0%*	*0%*
	0%		100%	-	*70%*	*30%*	*0%*
	0%		0%	-	*0%*	*0%*	*0%*
0	*0%*	*0*	0%	-	*0%*	*0%*	*0%*
0	*0%*	*0*	0%	-	*0%*	*0%*	*0%*
0	*0%*	*0*	0%	-	*0%*	*0%*	*0%*
0	*20%*	*0*	0%	-	*0%*	*0%*	*0%*
0	*0%*	*0*	50%	-	*20%*	*80%*	*0%*
0	*0%*	*0*	0%	-	*0%*	*0%*	*0%*
0	*0%*	*0*	35%	-	*100%*	*0%*	*0%*
	0%		0%	-	*0%*	*0%*	*0%*
	0%		0%	-	*0%*	*0%*	*0%*
0	*0%*	*0*	0%	-	*0%*	*0%*	*0%*
	0%		0%	-	*0%*	*0%*	*0%*
0	*0%*	*0*	0%	-	*0%*	*0%*	*0%*
0	*40%*	*0*	0%	-	*0%*	*0%*	*0%*
	0%		0%	-	*0%*	*0%*	*0%*
0	*0%*	*0*	0%	-	*0%*	*0%*	*0%*
0	*0%*	*0*	0%	-	*0%*	*0%*	*0%*
0	*0%*	*0*	0%	-	*0%*	*0%*	*0%*
0	*0%*	*0*	0%	-	*0%*	*0%*	*0%*
	0%		0%	-	*0%*	*0%*	*0%*
0	*0%*	*0*	0%	-	*0%*	*0%*	*0%*
0	*0%*	*0*	0%	-	*0%*	*0%*	*0%*
0	*0%*	*0*	0%	-	*0%*	*0%*	*0%*
0	*0%*	*0*	0%	-	*0%*	*0%*	*0%*
0	*0%*	*0*	0%	-	*0%*	*0%*	*0%*
0	*0%*	*0*	0%	-	*0%*	*0%*	*0%*
0	*0%*	*0*	0%	-	*0%*	*0%*	*0%*
0	*0%*	*0*	0%	-	*0%*	*0%*	*0%*
0	*0%*	*0*	0%	-	*0%*	*0%*	*0%*
0	*0%*	*0*	0%	-	*0%*	*0%*	*0%*
0	*0%*	*0*	0%	-	*0%*	*0%*	*0%*
0	*0%*	*0*	0%	-	*0%*	*0%*	*0%*
0	*0%*	*0*	0%	-	*0%*	*0%*	*0%*
0	*0%*	*0*	0%	-	*0%*	*0%*	*0%*
0	*100%*	*16*	0%	-	*0%*	*0%*	*0%*

0	96%	39,36	0%	-	0%	0%	0%
13,92	0%	0	0%	-	0%	0%	0%
	0%		0%	-	0%	0%	0%
21,525	0%	0	0%	-	0%	0%	0%
0	0%	0	0%	-	0%	0%	0%
	0%		0%	-	0%	0%	0%
6,3	0%	0	0%	-	0%	0%	0%
6,72	0%	0	0%	-	0%	0%	0%
	0%		0%	-	0%	0%	0%
0	0%	0	0%	-	0%	0%	0%
6,24	80%	24,96	0%	-	0%	0%	0%
0	0%	0	0%	-	0%	0%	0%
24,25	0%	0	0%	-	0%	0%	0%
0	0%	0	0%	-	0%	0%	0%
0	0%	0	0%	-	0%	0%	0%
0	0%	0	0%	-	0%	0%	0%
3,6	0%	0	0%	-	0%	0%	0%
23,1	0%	0	0%	-	0%	0%	0%
11,2	10%	1,6	0%	-	0%	0%	0%
25,52	0%	0	0%	-	0%	0%	0%
0	0%	0	0%	-	0%	0%	0%
	0%		0%	-	0%	0%	0%
19	0%	0	0%	-	0%	0%	0%
2,565	0%	0	0%	-	0%	0%	0%
14,52	18%	5,94	0%	-	0%	0%	0%
14,725	0%	0	0%	-	0%	0%	0%
0	0%	0	0%	-	0%	0%	0%
9,64864865	0%	0	0%	-	0%	0%	0%
0	0%	0	0%	-	0%	0%	0%
12,96	0%	0	0%	-	0%	0%	0%
0	0%	0	0%	-	0%	0%	0%
0	0%	0	0%	-	0%	0%	0%
2,4	0%	0	0%	-	0%	0%	0%
5,6	40%	6,4	0%	-	0%	0%	0%
0	100%	44	0%	-	0%	0%	0%
17,0336842	0%	0	0%	-	0%	0%	0%
0	0%	0	0%	-	0%	0%	0%
0	85%	27,2	0%	-	0%	0%	0%
0	0%	0	0%	-	0%	0%	0%
0	0%	0	0%	-	0%	0%	0%
13	0%	0	0%	-	0%	0%	0%
5,75	18%	4,14	0%	-	0%	0%	0%
13,26	20%	4,42	0%	-	0%	0%	0%
34,08	0%	0	0%	-	0%	0%	0%
5,18823529	36%	10,29411765	0%	-	0%	0%	0%
	0%		0%	-	0%	0%	0%
3,6	70%	12,6	0%	-	0%	0%	0%
2,8	0%	0	0%	-	0%	0%	0%
19,8	15%	5,4	0%	-	0%	0%	0%
2,142	70%	14,994	10%	2,38	100%	0%	0%
	0%		0%	-	0%	0%	0%
0	100%	25	0%	-	0%	0%	0%
0	100%	0	0%	-	0%	0%	0%
3,76	90%	33,84	0%	-	0%	0%	0%
0	0%	0	0%	-	0%	0%	0%
0	65%	0	0%	-	0%	0%	0%
21,3	0%	0	0%	-	0%	0%	0%
6,3	0%	0	0%	-	0%	0%	0%
0	90%	15,66	0%	-	0%	0%	0%
0	68%	11,56	0%	-	0%	0%	0%
5,88	0%	0	0%	-	0%	0%	0%
15,4	0%	0	0%	-	0%	0%	0%
8,925	10%	1,785	0%	-	0%	0%	0%
12,4	0%	0	0%	-	0%	0%	0%
7,968	15%	2,988	0%	-	0%	0%	0%
6,144	50%	15,36	0%	-	0%	0%	0%

0	35%	14,13125	0%	-	0%	0%	0%
2,842	45%	12,789	0%	-	0%	0%	0%
2,04	60%	12,24	0%	-	0%	0%	0%
4,05	60%	8,1	0%	-	0%	0%	0%
	0%		0%	-	0%	0%	0%
2,376	30%	7,128	0%	-	0%	0%	0%
31,2	19%	9,88	0%	-	0%	0%	0%
0	70%	14	0%	-	0%	0%	0%
3,68	80%	14,72	0%	-	0%	0%	0%
	0%		0%	-	0%	0%	0%
7,2	0%	0	0%	-	0%	0%	0%
10,125	10%	1,35	0%	-	0%	0%	0%
	0%		0%	-	0%	0%	0%
	0%		0%	-	0%	0%	0%
16,2	40%	32,4	0%	-	0%	0%	0%
4,2	80%	16,8	0%	-	0%	0%	0%
56	0%	0	0%	-	0%	0%	0%
62,05	10%	7,3	0%	-	0%	0%	0%
26	0%	0	0%	-	0%	0%	0%
14,28	0%	0	0%	-	0%	0%	0%
	0%		0%	-	0%	0%	0%
5,78	0%	0	0%	-	0%	0%	0%
0	100%	66	0%	-	0%	0%	0%
8,82	0%	0	0%	-	0%	0%	0%
19,584	16%	7,8336	0%	-	0%	0%	0%
	0%		0%	-	0%	0%	0%
5,25	85%	29,75	0%	-	0%	0%	0%
4,32	40%	8,64	0%	-	0%	0%	0%
10	0%	0	0%	-	0%	0%	0%
11,7	40%	15,6	0%	-	0%	0%	0%
9,6	50%	24	0%	-	0%	0%	0%
2,4	0%	0	0%	-	0%	0%	0%
5,7	17%	6,46	0%	-	0%	0%	0%
16	10%	3,2	0%	-	0%	0%	0%
0	0%	0	0%	-	0%	0%	0%
0	0%	0	0%	-	0%	0%	0%
0	0%	0	0%	-	0%	0%	0%
0	0%	0	0%	-	0%	0%	0%
0	0%	0	0%	-	0%	0%	0%
	0%		0%	-	0%	0%	0%
0	0%	0	0%	-	0%	0%	0%
8	0%	0	0%	-	0%	0%	0%
4,5675	5%	0,6525	0%	-	0%	0%	0%
0	100%	51,6	0%	-	0%	0%	0%
7,5	60%	18	0%	-	0%	0%	0%
11,82384	0%	0	0%	-	0%	0%	0%
3,087	25%	7,7175	0%	-	0%	0%	0%
13,776	0%	0	0%	-	0%	0%	0%
11,2	0%	0	0%	-	0%	0%	0%
	0%		0%	-	0%	0%	0%
12	43%	20,64	0%	-	0%	0%	0%
13	15%	7,8	0%	-	0%	0%	0%
2,99	0%	0	0%	-	0%	0%	0%
10,36	30%	7,77	0%	-	0%	0%	0%
12,6919111	20%	6,345955556	29%	12,94	100%	0%	0%
30,6	0%	0	10%	3,40	0%	100%	0%
22,95	0%	0	0%	-	0%	0%	0%
26,2236	0%	0	0%	-	0%	0%	0%
6,2	0%	0	0%	-	0%	0%	0%
8,325	40%	6,66	0%	-	0%	0%	0%
4,8	0%	0	0%	-	0%	0%	0%
7,05	0%	0	0%	-	0%	0%	0%
9,3925	20%	2,89	0%	-	0%	0%	0%
0	0%	0	0%	-	0%	0%	0%
0	50%	6,8	20%	3,40	0%	75%	25%
11,2	27%	8,64	0%	-	0%	0%	0%

2,85	0%	0	0%	-	0%	0%	0%
8,505	40%	7,56	0%	-	0%	0%	0%
0	80%	24,8	0%	-	0%	0%	0%
34,8	30%	26,1	0%	-	0%	0%	0%
21,6	0%	0	0%	-	0%	0%	0%
15,6	6%	1,44	0%	-	0%	0%	0%
20,8	20%	6,4	0%	-	0%	0%	0%
16,5	10%	2,2	0%	-	0%	0%	0%
0,5	0%	0	0%	-	0%	0%	0%
12	0%	0	0%	-	0%	0%	0%
10	0%	0	0%	-	0%	0%	0%
	0%		0%	-	0%	0%	0%
0	0%	0	0%	-	0%	0%	0%
18,9	0%	0	0%	-	0%	0%	0%
15,75	25%	7,875	0%	-	0%	0%	0%
12,705	0%	0	0%	-	0%	0%	0%
0	0%	0	0%	-	0%	0%	0%
	0%		0%	-	0%	0%	0%
65,2	0%	0	0%	-	0%	0%	0%
15,2	0%	0	0%	-	0%	0%	0%
4,86	60%	7,29	0%	-	0%	0%	0%
15,416	30%	9,2496	0%	-	0%	0%	0%
5	50%	5	0%	-	0%	0%	0%
9,18	70%	21,42	0%	-	0%	0%	0%
0	100%	35	0%	-	0%	0%	0%
11,8	80%	47,2	0%	-	0%	0%	0%
0	0%	0	0%	-	0%	0%	0%
3,42	0%	0	0%	-	0%	0%	0%
0	66%	19,14	0%	-	0%	0%	0%
1,14	65%	24,7	0%	-	0%	0%	0%
4,592	45%	20,664	0%	-	0%	0%	0%
15,4	30%	8,4	0%	-	0%	0%	0%
	0%		0%	-	0%	0%	0%
	0%		100%	112,00	100%	0%	0%
5,244	0%	0	0%	-	0%	0%	0%
0	0%	0	0%	-	0%	0%	0%
	0%		0%	-	0%	0%	0%
0	0%	0	0%	-	0%	0%	0%
20,4	32%	9,6	0%	-	0%	0%	0%
10	0%	0	0%	-	0%	0%	0%
	0%		0%	-	0%	0%	0%
0	0%	0	0%	-	0%	0%	0%
15,05	20%	7	0%	-	0%	0%	0%
33,66	10%	5,1	0%	-	0%	0%	0%
14,7	15%	3,15	0%	-	0%	0%	0%
4,29	0%	0	0%	-	0%	0%	0%
10,92	20%	3,64	0%	-	0%	0%	0%
31,75	5%	6,35	0%	-	0%	0%	0%
1,53	5%	0,51	0%	-	0%	0%	0%
0	0%	0	0%	-	0%	0%	0%
2,16	60%	10,8	0%	-	0%	0%	0%
0	100%	29	22%	8,14	60%	40%	0%
11	30%	6,6	0%	-	0%	0%	0%
0,98	0%	0	46%	5,98	100%	0%	0%
	0%		0%	-	0%	0%	0%
12	72%	54	0%	-	0%	0%	0%
7,2576	76%	22,9824	0%	-	0%	0%	0%
21,28	0%	0	0%	-	0%	0%	0%
3,7	0%	0	0%	-	0%	0%	0%
5,4	40%	10,8	0%	-	0%	0%	0%
14,4	10%	2,4	0%	-	0%	0%	0%
0	0%	0	0%	-	0%	0%	0%
17,052	28%	8,232	0%	-	0%	0%	0%
9	0%	0	0%	-	0%	0%	0%
0	74%	14,06	0%	-	0%	0%	0%
0	0%	0	0%	-	0%	0%	0%

0	0%	0	0%	-	0%	0%	0%
0	100%	25,2	0%	-	0%	0%	0%
0	0%	0	0%	-	0%	0%	0%
0	79%	30,81	0%	-	0%	0%	0%
0	80%	0	0%	-	0%	0%	0%
0	93%	93	0%	-	0%	0%	0%
15,75	0%	0	0%	-	0%	0%	0%
0	100%	60	0%	-	0%	0%	0%
0	89%	24,03	0%	-	0%	0%	0%
0	100%	11	0%	-	0%	0%	0%
6	15%	3	0%	-	0%	0%	0%
8,7	10%	2,9	0%	-	0%	0%	0%
10,5	30%	10,5	0%	-	0%	0%	0%
13,2	10%	2,2	0%	-	0%	0%	0%
9,6	0%	0	0%	-	0%	0%	0%
2,52	64%	8,96	0%	-	0%	0%	0%
11,52	0%	0	0%	-	0%	0%	0%
6,6	40%	8,8	0%	-	0%	0%	0%
2,6	50%	13	0%	-	0%	0%	0%
7,8	30%	7,8	0%	-	0%	0%	0%
0	0%	0	0%	-	0%	0%	0%
6,8	40%	6,8	0%	-	0%	0%	0%
0	0%	0	0%	-	0%	0%	0%
0	57%	8,55	0%	-	0%	0%	0%
0	80%	8,8	0%	-	0%	0%	0%
6,72	25%	5,25	0%	-	0%	0%	0%
	0%		0%	-	0%	0%	0%
3,404	0%	0	0%	-	0%	0%	0%
6,5	12%	1,56	0%	-	0%	0%	0%
0	0%	0	0%	-	0%	0%	0%
0	0%	0	56%	9,85	100%	0%	0%
0	100%	38,5	0%	-	0%	0%	0%
3,325	0%	0	0%	-	0%	0%	0%
1,14	50%	9,5	0%	-	0%	0%	0%
7,4	10%	3,7	0%	-	0%	0%	0%
4,5	30%	4,5	0%	-	0%	0%	0%
0	100%	12	0%	-	0%	0%	0%
6,232	0%	0	0%	-	0%	0%	0%
5,7	10%	1,9	0%	-	0%	0%	0%
29	0%	0	0%	-	0%	0%	0%
12,75	0%	0	0%	-	0%	0%	0%
0	100%	92	0%	-	0%	0%	0%
	0%		0%	-	0%	0%	0%
9,66	0%	0	0%	-	0%	0%	0%
0	0%	0	0%	-	0%	0%	0%
0	0%	0	0%	-	0%	0%	0%
12,04	0%	0	0%	-	0%	0%	0%
44,1	0%	0	0%	-	0%	0%	0%
	0%		0%	-	0%	0%	0%
0	0%	0	0%	-	0%	0%	0%
0	0%	0	0%	-	0%	0%	0%
	0%		0%	-	0%	0%	0%
	0%		0%	-	0%	0%	0%
17,01	0%	0	0%	-	0%	0%	0%
0	0%	0	0%	-	0%	0%	0%
7,8	40%	10,4	0%	-	0%	0%	0%
26,4	0%	0	0%	-	0%	0%	0%
0	0%	0	0%	-	0%	0%	0%
	0%		0%	-	0%	0%	0%
3,57647059	0%	0	0%	-	0%	0%	0%
0	0%	0	0%	-	0%	0%	0%
9,6	35%	11,2	0%	-	0%	0%	0%
8,0325	0%	0	0%	-	0%	0%	0%
0	0%	0	0%	-	0%	0%	0%
7,2	0%	0	0%	-	0%	0%	0%
14,4	10%	1,6	0%	-	0%	0%	0%

10,925	10%	2,185	0%	-	0%	0%	0%
	0%		0%	-	0%	0%	0%
8,64	50%	14,4	0%	-	0%	0%	0%
0	0%	0	0%	-	0%	0%	0%
13,2	0%	0	0%	-	0%	0%	0%
12	0%	0	0%	-	0%	0%	0%
0	0%	0	0%	-	0%	0%	0%
0	0%	0	0%	-	0%	0%	0%
0	0%	0	0%	-	0%	0%	0%
0	0%	0	0%	-	0%	0%	0%
0	0%	0	0%	-	0%	0%	0%
0	0%	0	0%	-	0%	0%	0%
3,05	0%	0	0%	-	0%	0%	0%
0	0%	0	0%	-	0%	0%	0%
0	0%	0	0%	-	0%	0%	0%
2,85	0%	0	0%	-	0%	0%	0%
0	0%	0	0%	-	0%	0%	0%
0	0%	0	0%	-	0%	0%	0%
0	0%	0	0%	-	0%	0%	0%
0	50%	7,5	0%	-	0%	0%	0%
0	0%	0	0%	-	0%	0%	0%
0	0%	0	0%	-	0%	0%	0%
1,76	0%	0	0%	-	0%	0%	0%
2,1	0%	0	0%	-	0%	0%	0%
0	0%	0	0%	-	0%	0%	0%
0	0%	0	0%	-	0%	0%	0%
23,22	0%	0	0%	-	0%	0%	0%
0	0%	0	0%	-	0%	0%	0%
0	0%	0	0%	-	0%	0%	0%
11,48	0%	0	0%	-	0%	0%	0%
0	0%	0	0%	-	0%	0%	0%
	0%		0%	-	0%	0%	0%
	0%		0%	-	0%	0%	0%
0	0%	0	0%	-	0%	0%	0%
0	0%	0	0%	-	0%	0%	0%
2,992	0%	0	0%	-	0%	0%	0%
0	0%	0	0%	-	0%	0%	0%
8	0%	0	0%	-	0%	0%	0%
7,04	0%	0	0%	-	0%	0%	0%
0	0%	0	0%	-	0%	0%	0%
0	0%	0	0%	-	0%	0%	0%
5,625	15%	3,375	0%	-	0%	0%	0%
15,3	45%	22,95	0%	-	0%	0%	0%
5,7	42%	8	0%	-	0%	0%	0%
0	19%	3,99	0%	-	0%	0%	0%
0	0%	0	0%	-	0%	0%	0%
9,5	0%	0	0%	-	0%	0%	0%
13,8	25%	5,75	0%	-	0%	0%	0%
184,8	0%	0	0%	-	0%	0%	0%
6,97	0%	0	0%	-	0%	0%	0%
2,5	0%	0	0%	-	0%	0%	0%
9,66	0%	0	0%	-	0%	0%	0%
15,96	11%	4	0%	-	0%	0%	0%
10,36	43%	32	0%	-	0%	0%	0%
0	0%	0	0%	-	0%	0%	0%
0	100%	17	0%	-	0%	0%	0%
0	0%	0	0%	-	0%	0%	0%
34,272	23%	14,076	0%	-	0%	0%	0%
0	0%	0	0%	-	0%	0%	0%
0	0%	0	0%	-	0%	0%	0%
0,576	0%	0	0%	-	0%	0%	0%
51	0%	0	0%	-	0%	0%	0%
0	0%	0	0%	-	0%	0%	0%
0	0%	0	0%	-	0%	0%	0%
0	0%	0	0%	-	0%	0%	0%
0	0%	0	0%	-	0%	0%	0%

0	0%	0	0%	-	0%	0%	0%
0	0%	0	0%	-	0%	0%	0%
0	0%	0	0%	-	0%	0%	0%
0	0%	0	0%	-	0%	0%	0%
0	0%	0	0%	-	0%	0%	0%
0	0%	0	0%	-	0%	0%	0%
0	0%	0	0%	-	0%	0%	0%
0	0%	0	0%	-	0%	0%	0%
0	0%	0	0%	-	0%	0%	0%
0	0%	0	0%	-	0%	0%	0%
0	0%	0	0%	-	0%	0%	0%
0	0%	0	0%	-	0%	0%	0%
0	0%	0	0%	-	0%	0%	0%
0	0%	0	0%	-	0%	0%	0%
0	0%	0	0%	-	0%	0%	0%
0	0%	0	0%	-	0%	0%	0%
0	0%	0	0%	-	0%	0%	0%
0	0%	0	0%	-	0%	0%	0%
0	0%	0	0%	-	0%	0%	0%
0	0%	0	0%	-	0%	0%	0%
	0%		0%	-	0%	0%	0%
0	0%	0	0%	-	0%	0%	0%
0	0%	0	0%	-	0%	0%	0%
0	0%	0	0%	-	0%	0%	0%
0	0%	0	0%	-	0%	0%	0%
0	0%	0	0%	-	0%	0%	0%
0	0%	0	0%	-	0%	0%	0%
0	0%	0	0%	-	0%	0%	0%
0	0%	0	0%	-	0%	0%	0%
0	0%	0	0%	-	0%	0%	0%
0	0%	0	60%	-	100%	0%	0%
0	0%	0	0%	-	0%	0%	0%
0	0%	0	0%	-	0%	0%	0%
0	0%	0	0%	-	0%	0%	0%
0	0%	0	0%	-	0%	0%	0%
0	100%	10	0%	-	0%	0%	0%
31	0%	0	0%	-	0%	0%	0%
	0%		0%	-	0%	0%	0%
14,8	65%	48,1	0%	-	0%	0%	0%
11,322	50%	15,3	5%	1,70	0%	100%	0%
2,625	0%	0	15%	5,25	0%	100%	0%
3,5	15%	1,5	11%	2,00	0%	100%	0%
	0%		0%	-	0%	0%	0%
	0%		0%	-	0%	0%	0%
	0%		0%	-	0%	0%	0%
	0%		0%	-	0%	0%	0%
	0%		0%	-	0%	0%	0%
0	0%	0	0%	-	0%	0%	0%
7,4	10%	3,7	0%	-	0%	0%	0%
5,84	0%	0	0%	-	0%	0%	0%
0	84%	53,2224	4%	2,64	100%	0%	0%
22,05	15%	9,45	0%	-	0%	0%	0%
22,4	30%	33,6	0%	-	0%	0%	0%
9,6	30%	7,2	0%	-	0%	0%	0%
21,6	0%	0	0%	-	0%	0%	0%
0	0%	0	0%	-	0%	0%	0%
20,37	20%	8,148	0%	-	0%	0%	0%
0	0%	0	0%	-	0%	0%	0%
	0%		0%	-	0%	0%	0%
	0%		0%	-	0%	0%	0%
4,4	0%	0	0%	-	0%	0%	0%
0	0%	0	0%	-	0%	0%	0%
0	0%	0	0%	-	0%	0%	0%
9,45	0%	0	0%	-	0%	0%	0%
0	90%	14,4	0%	-	0%	0%	0%
13,5	20%	5,4	0%	-	0%	0%	0%

5,7375	75%	17,2125	0%	-	0%	0%	0%
12,07	0%	0	0%	-	0%	0%	0%
	0%		0%	-	0%	0%	0%
11,02	42%	8	0%	-	0%	0%	0%
7,5	30%	15	0%	-	0%	0%	0%
0	0%	0	0%	-	0%	0%	0%
0	0%	0	0%	-	0%	0%	0%
0	0%	0	0%	-	0%	0%	0%
0	0%	0	0%	-	0%	0%	0%
3,5	0%	0	0%	-	0%	0%	0%
3,92	0%	0	0%	-	0%	0%	0%
0	0%	0	0%	-	0%	0%	0%
0	0%	0	0%	-	0%	0%	0%
0	0%	0	0%	-	0%	0%	0%
0	0%	0	0%	-	0%	0%	0%
38,961	8%	4,212	0%	-	0%	0%	0%
10,5	10%	3	0%	-	0%	0%	0%
	0%		0%	-	0%	0%	0%
	0%		0%	-	0%	0%	0%
25,6	0%	0	0%	-	0%	0%	0%
	0%		0%	-	0%	0%	0%
	0%		0%	-	0%	0%	0%
	0%		0%	-	0%	0%	0%
23,2848	0%	0	0%	-	0%	0%	0%
	0%		0%	-	0%	0%	0%
	0%		0%	-	0%	0%	0%
0	0%	0	0%	-	0%	0%	0%
36,4	30%	16,8	0%	-	0%	0%	0%
19,68	35%	13,776	0%	-	0%	0%	0%
9,6	27%	4,32	0%	-	0%	0%	0%
7,2	40%	7,2	0%	-	0%	0%	0%
0	0%	0	0%	-	0%	0%	0%
0	0%	0	0%	-	0%	0%	0%
3	0%	0	0%	-	0%	0%	0%
14	0%	0	0%	-	0%	0%	0%
28,05	0%	0	0%	-	0%	0%	0%
0	0%	0	0%	-	0%	0%	0%
	0%		0%	-	0%	0%	0%
0	20%	6,8	0%	-	0%	0%	0%
0	0%	0	0%	-	0%	0%	0%
0	0%	0	0%	-	0%	0%	0%
0	0%	0	0%	-	0%	0%	0%
0	0%	0	0%	-	0%	0%	0%
0	0%	0	0%	-	0%	0%	0%
0	0%	0	0%	-	0%	0%	0%
0	0%	0	0%	-	0%	0%	0%
0	0%	0	0%	-	0%	0%	0%
5,456	90%	49,104	0%	-	0%	0%	0%
0	0%	0	0%	-	0%	0%	0%
19,8	0%	0	0%	-	0%	0%	0%
0	0%	0	0%	-	0%	0%	0%
25,704	25%	10,71	0%	-	0%	0%	0%
30	0%	0	0%	-	0%	0%	0%
18	0%	0	0%	-	0%	0%	0%
	0%		0%	-	0%	0%	0%
0	0%	0	0%	-	0%	0%	0%
4,68	44%	11,44	0%	-	0%	0%	0%
0	0%	0	0%	-	0%	0%	0%
0	0%	0	0%	-	0%	0%	0%
	0%		0%	-	0%	0%	0%
0	0%	0	0%	-	0%	0%	0%
10,152	60%	40,608	0%	-	0%	0%	0%
	0%		0%	-	0%	0%	0%
0	0%	0	0%	-	0%	0%	0%
0	0%	0	0%	-	0%	0%	0%
9,8	30%	4,2	0%	-	0%	0%	0%

8,8	*50%*	*11*	0%	-	*0%*	*0%*	*0%*
0	*0%*	*0*	0%	-	*0%*	*0%*	*0%*
	0%		0%	-	*0%*	*0%*	*0%*
13,11	*80%*	*69,92*	0%	-	*0%*	*0%*	*0%*
6,63	*0%*	*0*	0%	-	*0%*	*0%*	*0%*
	0%		0%	-	*0%*	*0%*	*0%*
0	*75%*	*0*	0%	-	*0%*	*0%*	*0%*
48	*0%*	*0*	0%	-	*0%*	*0%*	*0%*
0	*0%*	*0*	0%	-	*0%*	*0%*	*0%*
0	*0%*	*0*	0%	-	*0%*	*0%*	*0%*
	0%		0%	-	*0%*	*0%*	*0%*
0	*0%*	*0*	0%	-	*0%*	*0%*	*0%*
18,2	*10%*	*9,1*	0%	-	*0%*	*0%*	*0%*
0	*0%*	*0*	0%	-	*0%*	*0%*	*0%*
0	*0%*	*0*	0%	-	*0%*	*0%*	*0%*
11,5	*0%*	*0*	0%	-	*0%*	*0%*	*0%*
22,464	*8%*	*2,496*	20%	7,80	*100%*	*0%*	*0%*
23,6	*30%*	*17,7*	0%	-	*0%*	*0%*	*0%*
18,9	*0%*	*0*	0%	-	*0%*	*0%*	*0%*
16,06	*78%*	*56,94*	0%	-	*0%*	*0%*	*0%*
0	*0%*	*0*	0%	-	*0%*	*0%*	*0%*
0	*0%*	*0*	0%	-	*0%*	*0%*	*0%*
	0%		0%	-	*0%*	*0%*	*0%*
10,8	*30%*	*10,8*	0%	-	*0%*	*0%*	*0%*
0	*0%*	*0*	0%	-	*0%*	*0%*	*0%*
2,97	*0%*	*0*	0%	-	*0%*	*0%*	*0%*
	0%		0%	-	*0%*	*0%*	*0%*
0	*0%*	*0*	0%	-	*0%*	*0%*	*0%*
2,907	*0%*	*0*	0%	-	*0%*	*0%*	*0%*
0	*0%*	*0*	0%	-	*0%*	*0%*	*0%*
0	*0%*	*0*	0%	-	*0%*	*0%*	*0%*
0	*0%*	*0*	0%	-	*0%*	*0%*	*0%*
	0%		0%	-	*0%*	*0%*	*0%*
	0%		0%	-	*0%*	*0%*	*0%*
	0%		0%	-	*0%*	*0%*	*0%*
0	*0%*	*0*	0%	-	*0%*	*0%*	*0%*
	0%		0%	-	*0%*	*0%*	*0%*
	0%		0%	-	*0%*	*0%*	*0%*
	0%		0%	-	*0%*	*0%*	*0%*
	0%		0%	-	*0%*	*0%*	*0%*
	0%		0%	-	*0%*	*0%*	*0%*
	0%		0%	-	*0%*	*0%*	*0%*
	0%		0%	-	*0%*	*0%*	*0%*
	0%		0%	-	*0%*	*0%*	*0%*
0	*0%*	*0*	0%	-	*0%*	*0%*	*0%*
0	*60%*	*7,2*	0%	-	*0%*	*0%*	*0%*
	0%		0%	-	*0%*	*0%*	*0%*
0	*0%*	*0*	0%	-	*0%*	*0%*	*0%*
	0%		0%	-	*0%*	*0%*	*0%*
0	*100%*	*6,75*	0%	-	*0%*	*0%*	*0%*
14,364	*70%*	*33,516*	0%	-	*0%*	*0%*	*0%*
3,6	*70%*	*8,4*	0%	-	*0%*	*0%*	*0%*
0	*0%*	*0*	0%	-	*0%*	*0%*	*0%*
4,2	*0%*	*0*	0%	-	*0%*	*0%*	*0%*
0	*0%*	*0*	0%	-	*0%*	*0%*	*0%*
3,5	*0%*	*0*	0%	-	*0%*	*0%*	*0%*
1,75	*0%*	*0*	0%	-	*0%*	*0%*	*0%*
9,84	*10%*	*3,28*	0%	-	*0%*	*0%*	*0%*
0	*0%*	*0*	0%	-	*0%*	*0%*	*0%*
6	*0%*	*0*	0%	-	*0%*	*0%*	*0%*
0	*0%*	*0*	0%	-	*0%*	*0%*	*0%*
	0%		0%	-	*0%*	*0%*	*0%*
33,12	*0%*	*0*	0%	-	*0%*	*0%*	*0%*
8,91	*0%*	*0*	0%	-	*0%*	*0%*	*0%*
22,5225	*20%*	*8,19*	0%	-	*0%*	*0%*	*0%*
14,6	*80%*	*58,4*	0%	-	*0%*	*0%*	*0%*

34,4	*30%*	*25,8*	0%	-	*0%*	*0%*	*0%*
0	*0%*	*0*	0%	-	*0%*	*0%*	*0%*
0	*0%*	*0*	0%	-	*0%*	*0%*	*0%*
0	*0%*	*0*	0%	-	*0%*	*0%*	*0%*
44,22	*0%*	*0*	0%	-	*0%*	*0%*	*0%*
0	*0%*	*0*	0%	-	*0%*	*0%*	*0%*
9,8	*50%*	*24,5*	0%	-	*0%*	*0%*	*0%*
0	*100%*	*46*	0%	-	*0%*	*0%*	*0%*
37,6	*13%*	*6,11*	0%	-	*0%*	*0%*	*0%*
	0%		0%	-	*0%*	*0%*	*0%*
2,808	*0%*	*0*	0%	-	*0%*	*0%*	*0%*
0	*47%*	*7,05*	0%	-	*0%*	*0%*	*0%*
6,72	*30%*	*8,4*	0%	-	*0%*	*0%*	*0%*
18	*0%*	*0*	0%	-	*0%*	*0%*	*0%*
3,2712	*84%*	*45,7968*	0%	-	*0%*	*0%*	*0%*
	0%		0%	-	*0%*	*0%*	*0%*
22,05	*0%*	*0*	0%	-	*0%*	*0%*	*0%*
43	*0%*	*0*	0%	-	*0%*	*0%*	*0%*
26	*0%*	*0*	0%	-	*0%*	*0%*	*0%*
32	*0%*	*0*	0%	-	*0%*	*0%*	*0%*
	0%		0%	-	*0%*	*0%*	*0%*
23,5	*50%*	*23,5*	0%	-	*0%*	*0%*	*0%*
0	*0%*	*0*	0%	-	*0%*	*0%*	*0%*
	0%		0%	-	*0%*	*0%*	*0%*
16,5	*0%*	*0*	0%	-	*0%*	*0%*	*0%*
14	*0%*	*0*	0%	-	*0%*	*0%*	*0%*

Sargassum % of interaction	Total length in interaction with Sargassum	*% Algae win*	*% Neutral*	*% Coral wins*	Turbinaria % of interaction	Total length in interaction with Turbinaria	*% Algae win*
0%	-	*0%*	*0%*	*0%*	0%	-	*0%*
0%	-	*0%*	*0%*	*0%*	0%	-	*0%*
0%	-	*0%*	*0%*	*0%*	0%	-	*0%*
0%	-	*0%*	*0%*	*0%*	0%	-	*0%*
0%	-	*0%*	*0%*	*0%*	0%	-	*0%*
0%	-	*0%*	*0%*	*0%*	0%	-	*0%*
0%	-	*0%*	*0%*	*0%*	0%	-	*0%*
0%	-	*0%*	*0%*	*0%*	80%	17,92	*100%*
0%	-	*0%*	*0%*	*0%*	0%	-	*0%*
0%	-	*0%*	*0%*	*0%*	5%	2,16	*100%*
0%	-	*0%*	*0%*	*0%*	0%	-	*0%*
0%	-	*0%*	*0%*	*0%*	0%	-	*0%*
0%	-	*0%*	*0%*	*0%*	0%	-	*0%*
0%	-	*0%*	*0%*	*0%*	0%	-	*0%*
0%	-	*0%*	*0%*	*0%*	0%	-	*0%*
0%	-	*0%*	*0%*	*0%*	0%	-	*0%*
0%	-	*0%*	*0%*	*0%*	10%	25,20	*100%*
0%	-	*0%*	*0%*	*0%*	0%	-	*0%*
0%	-	*0%*	*0%*	*0%*	0%	-	*0%*
0%	-	*0%*	*0%*	*0%*	0%	-	*0%*
0%	-	*0%*	*0%*	*0%*	0%	-	*0%*
0%	-	*0%*	*0%*	*0%*	0%	-	*0%*
0%	-	*0%*	*0%*	*0%*	0%	-	*0%*
0%	-	*0%*	*0%*	*0%*	0%	-	*0%*
0%	-	*0%*	*0%*	*0%*	0%	-	*0%*
0%	-	*0%*	*0%*	*0%*	0%	-	*0%*
0%	-	*0%*	*0%*	*0%*	0%	-	*0%*
0%	-	*0%*	*0%*	*0%*	0%	-	*0%*
0%	-	*0%*	*0%*	*0%*	20%	9,04	*100%*
0%	-	*0%*	*0%*	*0%*	0%	-	*0%*
0%	-	*0%*	*0%*	*0%*	0%	-	*0%*
0%	-	*0%*	*0%*	*0%*	0%	-	*0%*
0%	-	*0%*	*0%*	*0%*	0%	-	*0%*
35%	19,22	*30%*	*20%*	*50%*	0%	-	*0%*
0%	-	*0%*	*0%*	*0%*	0%	-	*0%*
0%	-	*0%*	*0%*	*0%*	0%	-	*0%*
0%	-	*0%*	*0%*	*0%*	0%	-	*0%*
0%	-	*0%*	*0%*	*0%*	0%	-	*0%*
0%	-	*0%*	*0%*	*0%*	0%	-	*0%*
0%	-	*0%*	*0%*	*0%*	0%	-	*0%*
0%	-	*0%*	*0%*	*0%*	20%	25,96	*60%*
0%	-	*0%*	*0%*	*0%*	0%	-	*0%*
0%	-	*0%*	*0%*	*0%*	0%	-	*0%*
0%	-	*0%*	*0%*	*0%*	0%	-	*0%*
0%	-	*0%*	*0%*	*0%*	0%	-	*0%*
0%	-	*0%*	*0%*	*0%*	0%	-	*0%*
0%	-	*0%*	*0%*	*0%*	0%	-	*0%*
0%	-	*0%*	*0%*	*0%*	0%	-	*0%*
0%	-	*0%*	*0%*	*0%*	0%	-	*0%*
0%	-	*0%*	*0%*	*0%*	0%	-	*0%*
0%	-	*0%*	*0%*	*0%*	0%	-	*0%*
0%	-	*0%*	*0%*	*0%*	0%	-	*0%*
0%	-	*0%*	*0%*	*0%*	0%	-	*0%*
0%	-	*0%*	*0%*	*0%*	0%	-	*0%*
0%	-	*0%*	*0%*	*0%*	0%	-	*0%*
0%	-	*0%*	*0%*	*0%*	70%	10,92	*20%*
0%	-	*0%*	*0%*	*0%*	0%	-	*0%*
0%	-	*0%*	*0%*	*0%*	0%	-	*0%*
0%	-	*0%*	*0%*	*0%*	0%	-	*0%*
0%	-	*0%*	*0%*	*0%*	0%	-	*0%*
0%	-	*0%*	*0%*	*0%*	0%	-	*0%*
0%	-	*0%*	*0%*	*0%*	15%	12,11	*100%*

0%	-	*0%*	*0%*	*0%*	0%	-	*0%*
0%	-	*0%*	*0%*	*0%*	0%	-	*0%*
0%	-	*0%*	*0%*	*0%*	0%	-	*0%*
0%	-	*0%*	*0%*	*0%*	0%	-	*0%*
0%	-	*0%*	*0%*	*0%*	0%	-	*0%*
0%	-	*0%*	*0%*	*0%*	0%	-	*0%*
0%	-	*0%*	*0%*	*0%*	30%	10,97	*20%*
0%	-	*0%*	*0%*	*0%*	0%	-	*0%*
0%	-	*0%*	*0%*	*0%*	0%	-	*0%*
0%	-	*0%*	*0%*	*0%*	0%	-	*0%*
0%	-	*0%*	*0%*	*0%*	0%	-	*0%*
0%	-	*0%*	*0%*	*0%*	0%	-	*0%*
0%	-	*0%*	*0%*	*0%*	0%	-	*0%*
0%	-	*0%*	*0%*	*0%*	0%	-	*0%*
0%	-	*0%*	*0%*	*0%*	0%	-	*0%*
0%	-	*0%*	*0%*	*0%*	0%	-	*0%*
0%	-	*0%*	*0%*	*0%*	45%	4,19	*0%*
0%	-	*0%*	*0%*	*0%*	0%	-	*0%*
0%	-	*0%*	*0%*	*0%*	0%	-	*0%*
0%	-	*0%*	*0%*	*0%*	0%	-	*0%*
0%	-	*0%*	*0%*	*0%*	0%	-	*0%*
0%	-	*0%*	*0%*	*0%*	0%	-	*0%*
0%	-	*0%*	*0%*	*0%*	0%	-	*0%*
0%	-	*0%*	*0%*	*0%*	0%	-	*0%*
0%	-	*0%*	*0%*	*0%*	28%	1,08	*0%*
0%	-	*0%*	*0%*	*0%*	0%	-	*0%*
0%	-	*0%*	*0%*	*0%*	0%	-	*0%*
0%	-	*0%*	*0%*	*0%*	0%	-	*0%*
0%	-	*0%*	*0%*	*0%*	0%	-	*0%*
0%	-	*0%*	*0%*	*0%*	0%	-	*0%*
0%	-	*0%*	*0%*	*0%*	0%	-	*0%*
0%	-	*0%*	*0%*	*0%*	0%	-	*0%*
0%	-	*0%*	*0%*	*0%*	0%	-	*0%*
0%	-	*0%*	*0%*	*0%*	0%	-	*0%*
0%	-	*0%*	*0%*	*0%*	0%	-	*0%*
80%	4,00	*70%*	*30%*	*0%*	0%	-	*0%*
0%	-	*0%*	*0%*	*0%*	0%	-	*0%*
0%	-	*0%*	*0%*	*0%*	0%	-	*0%*
0%	-	*0%*	*0%*	*0%*	0%	-	*0%*
0%	-	*0%*	*0%*	*0%*	0%	-	*0%*
0%	-	*0%*	*0%*	*0%*	0%	-	*0%*
0%	-	*0%*	*0%*	*0%*	0%	-	*0%*
0%	-	*0%*	*0%*	*0%*	0%	-	*0%*
0%	-	*0%*	*0%*	*0%*	30%	3,24	*100%*
0%	-	*0%*	*0%*	*0%*	0%	-	*0%*
0%	-	*0%*	*0%*	*0%*	0%	-	*0%*
0%	-	*0%*	*0%*	*0%*	0%	-	*0%*
0%	-	*0%*	*0%*	*0%*	0%	-	*0%*
20%	-	*70%*	*0%*	*30%*	0%	-	*0%*
25%	-	*50%*	*0%*	*50%*	0%	-	*0%*
0%	-	*0%*	*0%*	*0%*	0%	-	*0%*
0%	-	*0%*	*0%*	*0%*	0%	-	*0%*
0%	-	*0%*	*0%*	*0%*	0%	-	*0%*
0%	-	*0%*	*0%*	*0%*	0%	-	*0%*
0%	-	*0%*	*0%*	*0%*	0%	-	*0%*
0%	-	*0%*	*0%*	*0%*	0%	-	*0%*
0%	-	*0%*	*0%*	*0%*	0%	-	*0%*
0%	-	*0%*	*0%*	*0%*	0%	-	*0%*
0%	-	*0%*	*0%*	*0%*	0%	-	*0%*
0%	-	*0%*	*0%*	*0%*	0%	-	*0%*
0%	-	*0%*	*0%*	*0%*	0%	-	*0%*
0%	-	*0%*	*0%*	*0%*	0%	-	*0%*
0%	-	*0%*	*0%*	*0%*	0%	-	*0%*
0%	-	*0%*	*0%*	*0%*	0%	-	*0%*
0%	-	*0%*	*0%*	*0%*	0%	-	*0%*
0%	-	*0%*	*0%*	*0%*	0%	-	*0%*

0%	-	0%	0%	0%	0%	-	0%
0%	-	0%	0%	0%	0%	-	0%
0%	-	0%	0%	0%	0%	-	0%
0%	-	0%	0%	0%	0%	-	0%
0%	-	0%	0%	0%	0%	-	0%
0%	-	0%	0%	0%	0%	-	0%
0%	-	0%	0%	0%	0%	-	0%
0%	-	0%	0%	0%	0%	-	0%
0%	-	0%	0%	0%	0%	-	0%
0%	-	0%	0%	0%	0%	-	0%
0%	-	0%	0%	0%	0%	-	0%
0%	-	0%	0%	0%	0%	-	0%
0%	-	0%	0%	0%	0%	-	0%
0%	-	0%	0%	0%	0%	-	0%
0%	-	0%	0%	0%	38%	17,86	55%
0%	-	0%	0%	0%	50%	5,50	0%
0%	-	0%	0%	0%	30%	9,30	75%
0%	-	0%	0%	0%	0%	-	0%
0%	-	0%	0%	0%	0%	-	0%
0%	-	0%	0%	0%	0%	-	0%
0%	-	0%	0%	0%	0%	-	0%
0%	-	0%	0%	0%	37%	7,03	100%
0%	-	0%	0%	0%	15%	2,85	80%
0%	-	0%	0%	0%	0%	-	0%
0%	-	0%	0%	0%	0%	-	0%
0%	-	0%	0%	0%	0%	-	0%
0%	-	0%	0%	0%	0%	-	0%
0%	-	0%	0%	0%	75%	4,78	100%
0%	-	0%	0%	0%	25%	1,13	0%
0%	-	0%	0%	0%	0%	-	0%
0%	-	0%	0%	0%	0%	-	0%
0%	-	0%	0%	0%	0%	-	0%
0%	-	0%	0%	0%	0%	-	0%
0%	-	0%	0%	0%	0%	-	0%
0%	-	0%	0%	0%	0%	-	0%
0%	-	0%	0%	0%	0%	-	0%
0%	-	0%	0%	0%	0%	-	0%
0%	-	0%	0%	0%	0%	-	0%
0%	-	0%	0%	0%	0%	-	0%
0%	-	0%	0%	0%	0%	-	0%
0%	-	0%	0%	0%	5%	-	100%
0%	-	0%	0%	0%	0%	-	0%
0%	-	0%	0%	0%	20%	-	0%
0%	-	0%	0%	0%	0%	-	0%
0%	-	0%	0%	0%	0%	-	0%
0%	-	0%	0%	0%	0%	-	0%
0%	-	0%	0%	0%	5%	3,80	0%
0%	-	0%	0%	0%	0%	-	0%
0%	-	0%	0%	0%	25%	8,10	100%
0%	-	0%	0%	0%	0%	-	0%
0%	-	0%	0%	0%	0%	-	0%
0%	-	0%	0%	0%	0%	-	0%
0%	-	0%	0%	0%	0%	-	0%
0%	-	0%	0%	0%	5%	2,85	100%
0%	-	0%	0%	0%	22%	17,00	100%
0%	-	0%	0%	0%	0%	-	0%
0%	-	0%	0%	0%	0%	-	0%
0%	-	0%	0%	0%	0%	-	0%
0%	-	0%	0%	0%	0%	-	0%
0%	-	0%	0%	0%	0%	-	0%
0%	-	0%	0%	0%	0%	-	0%
0%	-	0%	0%	0%	0%	-	0%
0%	-	0%	0%	0%	50%	15,25	60%
0%	-	0%	0%	0%	100%	5,50	0%
0%	-	0%	0%	0%	0%	-	0%
0%	-	0%	0%	0%	0%	-	0%

0%	-	*0%*	*0%*	*0%*	0%	-	*0%*
0%	-	*0%*	*0%*	*0%*	0%	-	*0%*
0%	-	*0%*	*0%*	*0%*	0%	-	*0%*
0%	-	*0%*	*0%*	*0%*	0%	-	*0%*
0%	-	*0%*	*0%*	*0%*	0%	-	*0%*
0%	-	*0%*	*0%*	*0%*	0%	-	*0%*
0%	-	*0%*	*0%*	*0%*	0%	-	*0%*
0%	-	*0%*	*0%*	*0%*	0%	-	*0%*
0%	-	*0%*	*0%*	*0%*	0%	-	*0%*
0%	-	*0%*	*0%*	*0%*	0%	-	*0%*
0%	-	*0%*	*0%*	*0%*	20%	20,24	*15%*
0%	-	*0%*	*0%*	*0%*	0%	-	*0%*
0%	-	*0%*	*0%*	*0%*	0%	-	*0%*
0%	-	*0%*	*0%*	*0%*	0%	-	*0%*
0%	-	*0%*	*0%*	*0%*	0%	-	*0%*
0%	-	*0%*	*0%*	*0%*	0%	-	*0%*
0%	-	*0%*	*0%*	*0%*	0%	-	*0%*
0%	-	*0%*	*0%*	*0%*	0%	-	*0%*
0%	-	*0%*	*0%*	*0%*	0%	-	*0%*
0%	-	*0%*	*0%*	*0%*	0%	-	*0%*
0%	-	*0%*	*0%*	*0%*	0%	-	*0%*
0%	-	*0%*	*0%*	*0%*	0%	-	*0%*
0%	-	*0%*	*0%*	*0%*	0%	-	*0%*
0%	-	*0%*	*0%*	*0%*	0%	-	*0%*
0%	-	*0%*	*0%*	*0%*	0%	-	*0%*
0%	-	*0%*	*0%*	*0%*	0%	-	*0%*
0%	-	*0%*	*0%*	*0%*	0%	-	*0%*
0%	-	*0%*	*0%*	*0%*	0%	-	*0%*
0%	-	*0%*	*0%*	*0%*	0%	-	*0%*
0%	-	*0%*	*0%*	*0%*	85%	5,06	*75%*
0%	-	*0%*	*0%*	*0%*	0%	-	*0%*
0%	-	*0%*	*0%*	*0%*	0%	-	*0%*
0%	-	*0%*	*0%*	*0%*	0%	-	*0%*
0%	-	*0%*	*0%*	*0%*	0%	-	*0%*
0%	-	*0%*	*0%*	*0%*	0%	-	*0%*
0%	-	*0%*	*0%*	*0%*	45%	12,60	*0%*
0%	-	*0%*	*0%*	*0%*	20%	14,20	*60%*
0%	-	*0%*	*0%*	*0%*	0%	-	*0%*
0%	-	*0%*	*0%*	*0%*	0%	-	*0%*
0%	-	*0%*	*0%*	*0%*	0%	-	*0%*
0%	-	*0%*	*0%*	*0%*	0%	-	*0%*
0%	-	*0%*	*0%*	*0%*	50%	9,08	*0%*
0%	-	*0%*	*0%*	*0%*	0%	-	*0%*
0%	-	*0%*	*0%*	*0%*	0%	-	*0%*
0%	-	*0%*	*0%*	*0%*	0%	-	*0%*
0%	-	*0%*	*0%*	*0%*	0%	-	*0%*
5%	5,30	*0%*	*100%*	*0%*	0%	-	*0%*
0%	-	*0%*	*0%*	*0%*	0%	-	*0%*
0%	-	*0%*	*0%*	*0%*	40%	4,62	*0%*
0%	-	*0%*	*0%*	*0%*	0%	-	*0%*
0%	-	*0%*	*0%*	*0%*	0%	-	*0%*
0%	-	*0%*	*0%*	*0%*	0%	-	*0%*
0%	-	*0%*	*0%*	*0%*	50%	27,50	*50%*
0%	-	*0%*	*0%*	*0%*	0%	-	*0%*
0%	-	*0%*	*0%*	*0%*	0%	-	*0%*
0%	-	*0%*	*0%*	*0%*	0%	-	*0%*
0%	-	*0%*	*0%*	*0%*	0%	-	*0%*
0%	-	*0%*	*0%*	*0%*	0%	-	*0%*
0%	-	*0%*	*0%*	*0%*	0%	-	*0%*
0%	-	*0%*	*0%*	*0%*	0%	-	*0%*
0%	-	*0%*	*0%*	*0%*	0%	-	*0%*
0%	-	*0%*	*0%*	*0%*	0%	-	*0%*
0%	-	*0%*	*0%*	*0%*	0%	-	*0%*
0%	-	*0%*	*0%*	*0%*	0%	-	*0%*
0%	-	*0%*	*0%*	*0%*	0%	-	*0%*
0%	-	*0%*	*0%*	*0%*	0%	-	*0%*

0%	-	*0%*	*0%*	*0%*	0%	-	*0%*
0%	-	*0%*	*0%*	*0%*	0%	-	*0%*
0%	-	*0%*	*0%*	*0%*	0%	-	*0%*
0%	-	*0%*	*0%*	*0%*	0%	-	*0%*
0%	-	*0%*	*0%*	*0%*	0%	-	*0%*
0%	-	*0%*	*0%*	*0%*	35%	23,24	*100%*
0%	-	*0%*	*0%*	*0%*	0%	-	*0%*
0%	-	*0%*	*0%*	*0%*	0%	-	*0%*
0%	-	*0%*	*0%*	*0%*	0%	-	*0%*
0%	-	*0%*	*0%*	*0%*	22%	4,40	*40%*
0%	-	*0%*	*0%*	*0%*	0%	-	*0%*
0%	-	*0%*	*0%*	*0%*	0%	-	*0%*
0%	-	*0%*	*0%*	*0%*	0%	-	*0%*
0%	-	*0%*	*0%*	*0%*	0%	-	*0%*
0%	-	*0%*	*0%*	*0%*	100%	36,40	*80%*
0%	-	*0%*	*0%*	*0%*	0%	-	*0%*
0%	-	*0%*	*0%*	*0%*	0%	-	*0%*
0%	-	*0%*	*0%*	*0%*	0%	-	*0%*
0%	-	*0%*	*0%*	*0%*	0%	-	*0%*
0%	-	*0%*	*0%*	*0%*	0%	-	*0%*
0%	-	*0%*	*0%*	*0%*	0%	-	*0%*
0%	-	*0%*	*0%*	*0%*	0%	-	*0%*
0%	-	*0%*	*0%*	*0%*	0%	-	*0%*
0%	-	*0%*	*0%*	*0%*	0%	-	*0%*
0%	-	*0%*	*0%*	*0%*	0%	-	*0%*
0%	-	*0%*	*0%*	*0%*	0%	-	*0%*
0%	-	*0%*	*0%*	*0%*	0%	-	*0%*
0%	-	*0%*	*0%*	*0%*	0%	-	*0%*
0%	-	*0%*	*0%*	*0%*	0%	-	*0%*
0%	-	*0%*	*0%*	*0%*	0%	-	*0%*
0%	-	*0%*	*0%*	*0%*	0%	-	*0%*
0%	-	*0%*	*0%*	*0%*	0%	-	*0%*
0%	-	*0%*	*0%*	*0%*	0%	-	*0%*
0%	-	*0%*	*0%*	*0%*	0%	-	*0%*
0%	-	*0%*	*0%*	*0%*	0%	-	*0%*
0%	-	*0%*	*0%*	*0%*	0%	-	*0%*
0%	-	*0%*	*0%*	*0%*	0%	-	*0%*
0%	-	*0%*	*0%*	*0%*	0%	-	*0%*
0%	-	*0%*	*0%*	*0%*	0%	-	*0%*
0%	-	*0%*	*0%*	*0%*	0%	-	*0%*
0%	-	*0%*	*0%*	*0%*	0%	-	*0%*
0%	-	*0%*	*0%*	*0%*	0%	-	*0%*
0%	-	*0%*	*0%*	*0%*	0%	-	*0%*
0%	-	*0%*	*0%*	*0%*	0%	-	*0%*
0%	-	*0%*	*0%*	*0%*	0%	-	*0%*
0%	-	*0%*	*0%*	*0%*	0%	-	*0%*
0%	-	*0%*	*0%*	*0%*	0%	-	*0%*
0%	-	*0%*	*0%*	*0%*	0%	-	*0%*
0%	-	*0%*	*0%*	*0%*	0%	-	*0%*
0%	-	*0%*	*0%*	*0%*	0%	-	*0%*
0%	-	*0%*	*0%*	*0%*	0%	-	*0%*
0%	-	*0%*	*0%*	*0%*	0%	-	*0%*
0%	-	*0%*	*0%*	*0%*	0%	-	*0%*
0%	-	*0%*	*0%*	*0%*	0%	-	*0%*
0%	-	*0%*	*0%*	*0%*	0%	-	*0%*
30%	5,31	*100%*	*0%*	*0%*	0%	-	*0%*
0%	-	*0%*	*0%*	*0%*	0%	-	*0%*
0%	-	*0%*	*0%*	*0%*	0%	-	*0%*
0%	-	*0%*	*0%*	*0%*	0%	-	*0%*
0%	-	*0%*	*0%*	*0%*	0%	-	*0%*
0%	-	*0%*	*0%*	*0%*	0%	-	*0%*
0%	-	*0%*	*0%*	*0%*	0%	-	*0%*
0%	-	*0%*	*0%*	*0%*	0%	-	*0%*
0%	-	*0%*	*0%*	*0%*	0%	-	*0%*
70%	8,33	*20%*	*40%*	*40%*	0%	-	*0%*
0%	-	*0%*	*0%*	*0%*	0%	-	*0%*

60%	48,48	*100%*	*0%*	*0%*	0%	-	*0%*
0%	-	*0%*	*0%*	*0%*	0%	-	*0%*
0%	-	*0%*	*0%*	*0%*	0%	-	*0%*
0%	-	*0%*	*0%*	*0%*	0%	-	*0%*
0%	-	*0%*	*0%*	*0%*	0%	-	*0%*
0%	-	*0%*	*0%*	*0%*	0%	-	*0%*
0%	-	*0%*	*0%*	*0%*	0%	-	*0%*
0%	-	*0%*	*0%*	*0%*	0%	-	*0%*
0%	-	*0%*	*0%*	*0%*	0%	-	*0%*
0%	-	*0%*	*0%*	*0%*	0%	-	*0%*
0%	-	*0%*	*0%*	*0%*	0%	-	*0%*
0%	-	*0%*	*0%*	*0%*	0%	-	*0%*
0%	-	*0%*	*0%*	*0%*	0%	-	*0%*
0%	-	*0%*	*0%*	*0%*	0%	-	*0%*
0%	-	*0%*	*0%*	*0%*	0%	-	*0%*
0%	-	*0%*	*0%*	*0%*	0%	-	*0%*
0%	-	*0%*	*0%*	*0%*	0%	-	*0%*
0%	-	*0%*	*0%*	*0%*	0%	-	*0%*
0%	-	*0%*	*0%*	*0%*	0%	-	*0%*
0%	-	*0%*	*0%*	*0%*	0%	-	*0%*
0%	-	*0%*	*0%*	*0%*	0%	-	*0%*
0%	-	*0%*	*0%*	*0%*	0%	-	*0%*
0%	-	*0%*	*0%*	*0%*	0%	-	*0%*
0%	-	*0%*	*0%*	*0%*	25%	8,50	*20%*
0%	-	*0%*	*0%*	*0%*	0%	-	*0%*
0%	-	*0%*	*0%*	*0%*	0%	-	*0%*
0%	-	*0%*	*0%*	*0%*	0%	-	*0%*
0%	-	*0%*	*0%*	*0%*	0%	-	*0%*
0%	-	*0%*	*0%*	*0%*	0%	-	*0%*
0%	-	*0%*	*0%*	*0%*	0%	-	*0%*
0%	-	*0%*	*0%*	*0%*	0%	-	*0%*
0%	-	*0%*	*0%*	*0%*	0%	-	*0%*
35%	9,07	*0%*	*100%*	*0%*	0%	-	*0%*
0%	-	*0%*	*0%*	*0%*	0%	-	*0%*
0%	-	*0%*	*0%*	*0%*	0%	-	*0%*
0%	-	*0%*	*0%*	*0%*	0%	-	*0%*
0%	-	*0%*	*0%*	*0%*	0%	-	*0%*
0%	-	*0%*	*0%*	*0%*	0%	-	*0%*
0%	-	*0%*	*0%*	*0%*	0%	-	*0%*
0%	-	*0%*	*0%*	*0%*	0%	-	*0%*
0%	-	*0%*	*0%*	*0%*	0%	-	*0%*
0%	-	*0%*	*0%*	*0%*	0%	-	*0%*
0%	-	*0%*	*0%*	*0%*	0%	-	*0%*
0%	-	*0%*	*0%*	*0%*	0%	-	*0%*
0%	-	*0%*	*0%*	*0%*	53%	11,13	*10%*
0%	-	*0%*	*0%*	*0%*	0%	-	*0%*
0%	-	*0%*	*0%*	*0%*	0%	-	*0%*
0%	-	*0%*	*0%*	*0%*	0%	-	*0%*
0%	-	*0%*	*0%*	*0%*	0%	-	*0%*
0%	-	*0%*	*0%*	*0%*	0%	-	*0%*
0%	-	*0%*	*0%*	*0%*	0%	-	*0%*
0%	-	*0%*	*0%*	*0%*	0%	-	*0%*
0%	-	*0%*	*0%*	*0%*	0%	-	*0%*
0%	-	*0%*	*0%*	*0%*	0%	-	*0%*
0%	-	*0%*	*0%*	*0%*	0%	-	*0%*
0%	-	*0%*	*0%*	*0%*	0%	-	*0%*
0%	-	*0%*	*0%*	*0%*	0%	-	*0%*
0%	-	*0%*	*0%*	*0%*	0%	-	*0%*
0%	-	*0%*	*0%*	*0%*	0%	-	*0%*
0%	-	*0%*	*0%*	*0%*	0%	-	*0%*
0%	-	*0%*	*0%*	*0%*	0%	-	*0%*
0%	-	*0%*	*0%*	*0%*	0%	-	*0%*
0%	-	*0%*	*0%*	*0%*	0%	-	*0%*
0%	-	*0%*	*0%*	*0%*	0%	-	*0%*
0%	-	*0%*	*0%*	*0%*	0%	-	*0%*
0%	-	*0%*	*0%*	*0%*	0%	-	*0%*

0%	-	0%	0%	0%	0%	-	0%
0%	-	0%	0%	0%	0%	-	0%
0%	-	0%	0%	0%	0%	-	0%
0%	-	0%	0%	0%	0%	-	0%
0%	-	0%	0%	0%	0%	-	0%
0%	-	0%	0%	0%	0%	-	0%
0%	-	0%	0%	0%	0%	-	0%
0%	-	0%	0%	0%	0%	-	0%
0%	-	0%	0%	0%	0%	-	0%
0%	-	0%	0%	0%	0%	-	0%
0%	-	0%	0%	0%	0%	-	0%
0%	-	0%	0%	0%	0%	-	0%
0%	-	0%	0%	0%	0%	-	0%
0%	-	0%	0%	0%	0%	-	0%
0%	-	0%	0%	0%	0%	-	0%
0%	-	0%	0%	0%	0%	-	0%
0%	-	0%	0%	0%	0%	-	0%
0%	-	0%	0%	0%	0%	-	0%
0%	-	0%	0%	0%	0%	-	0%
0%	-	0%	0%	0%	0%	-	0%
0%	-	0%	0%	0%	0%	-	0%
0%	-	0%	0%	0%	0%	-	0%
0%	-	0%	0%	0%	0%	-	0%
0%	-	0%	0%	0%	0%	-	0%
0%	-	0%	0%	0%	0%	-	0%
0%	-	0%	0%	0%	0%	-	0%
0%	-	0%	0%	0%	0%	-	0%
0%	-	0%	0%	0%	0%	-	0%
0%	-	0%	0%	0%	0%	-	0%
0%	-	0%	0%	0%	0%	-	0%
0%	-	0%	0%	0%	0%	-	0%
0%	-	0%	0%	0%	0%	-	0%
0%	-	0%	0%	0%	0%	-	0%
0%	-	0%	0%	0%	0%	-	0%
0%	-	0%	0%	0%	0%	-	0%
0%	-	0%	0%	0%	0%	-	0%
0%	-	0%	0%	0%	0%	-	0%
0%	-	0%	0%	0%	0%	-	0%
0%	-	0%	0%	0%	0%	-	0%
0%	-	0%	0%	0%	0%	-	0%
0%	-	0%	0%	0%	0%	-	0%
0%	-	0%	0%	0%	0%	-	0%
0%	-	0%	0%	0%	0%	-	0%
0%	-	0%	0%	0%	0%	-	0%
0%	-	0%	0%	0%	0%	-	0%
0%	-	0%	0%	0%	0%	-	0%
0%	-	0%	0%	0%	0%	-	0%
0%	-	0%	0%	0%	0%	-	0%
0%	-	0%	0%	0%	0%	-	0%
0%	-	0%	0%	0%	0%	-	0%
0%	-	0%	0%	0%	0%	-	0%
0%	-	0%	0%	0%	0%	-	0%
0%	-	0%	0%	0%	0%	-	0%
0%	-	0%	0%	0%	0%	-	0%
0%	-	0%	0%	0%	0%	-	0%
0%	-	0%	0%	0%	0%	-	0%
0%	-	0%	0%	0%	0%	-	0%
0%	-	0%	0%	0%	0%	-	0%
0%	-	0%	0%	0%	0%	-	0%
0%	-	0%	0%	0%	0%	-	0%
0%	-	0%	0%	0%	0%	-	0%
0%	-	0%	0%	0%	0%	-	0%
0%	-	0%	0%	0%	0%	-	0%
0%	-	0%	0%	0%	0%	-	0%
0%	-	0%	0%	0%	0%	-	0%
0%	-	0%	0%	0%	0%	-	0%

0%	-	0%	0%	0%	0%	-	0%
0%	-	0%	0%	0%	0%	-	0%
0%	-	0%	0%	0%	0%	-	0%
0%	-	0%	0%	0%	0%	-	0%
0%	-	0%	0%	0%	0%	-	0%
0%	-	0%	0%	0%	0%	-	0%
0%	-	0%	0%	0%	0%	-	0%
0%	-	0%	0%	0%	0%	-	0%
0%	-	0%	0%	0%	0%	-	0%
0%	-	0%	0%	0%	0%	-	0%
0%	-	0%	0%	0%	0%	-	0%
0%	-	0%	0%	0%	0%	-	0%
0%	-	0%	0%	0%	0%	-	0%
0%	-	0%	0%	0%	0%	-	0%
0%	-	0%	0%	0%	0%	-	0%
0%	-	0%	0%	0%	0%	-	0%
0%	-	0%	0%	0%	0%	-	0%
0%	-	0%	0%	0%	0%	-	0%
0%	-	0%	0%	0%	0%	-	0%
0%	-	0%	0%	0%	0%	-	0%
0%	-	0%	0%	0%	0%	-	0%
0%	-	0%	0%	0%	0%	-	0%
0%	-	0%	0%	0%	0%	-	0%
0%	-	0%	0%	0%	0%	-	0%
0%	-	0%	0%	0%	0%	-	0%
0%	-	0%	0%	0%	0%	-	0%
0%	-	0%	0%	0%	0%	-	0%
0%	-	0%	0%	0%	0%	-	0%
0%	-	0%	0%	0%	0%	-	0%
0%	-	0%	0%	0%	0%	-	0%
0%	-	0%	0%	0%	0%	-	0%
0%	-	0%	0%	0%	0%	-	0%
0%	-	0%	0%	0%	0%	-	0%
0%	-	0%	0%	0%	0%	-	0%
0%	-	0%	0%	0%	0%	-	0%
0%	-	0%	0%	0%	0%	-	0%
0%	-	0%	0%	0%	0%	-	0%
0%	-	0%	0%	0%	0%	-	0%
0%	-	0%	0%	0%	0%	-	0%
0%	-	0%	0%	0%	0%	-	0%
0%	-	0%	0%	0%	0%	-	0%
0%	-	0%	0%	0%	0%	-	0%
0%	-	0%	0%	0%	0%	-	0%
0%	-	0%	0%	0%	0%	-	0%
0%	-	0%	0%	0%	0%	-	0%
0%	-	0%	0%	0%	0%	-	0%
0%	-	0%	0%	0%	0%	-	0%
0%	-	0%	0%	0%	100%	11,70	100%
0%	-	0%	0%	0%	20%	2,75	100%
0%	-	0%	0%	0%	0%	-	0%
0%	-	0%	0%	0%	0%	-	0%
0%	-	0%	0%	0%	0%	-	0%
0%	-	0%	0%	0%	0%	-	0%
0%	-	0%	0%	0%	0%	-	0%
0%	-	0%	0%	0%	0%	-	0%
0%	-	0%	0%	0%	0%	-	0%
0%	-	0%	0%	0%	0%	-	0%
0%	-	0%	0%	0%	0%	-	0%
0%	-	0%	0%	0%	0%	-	0%
0%	-	0%	0%	0%	0%	-	0%
0%	-	0%	0%	0%	0%	-	0%
0%	-	0%	0%	0%	0%	-	0%
0%	-	0%	0%	0%	0%	-	0%
0%	-	0%	0%	0%	0%	-	0%
0%	-	0%	0%	0%	0%	-	0%
0%	-	0%	0%	0%	0%	-	0%

0%	-	*0%*	*0%*	*0%*	0%	-	*0%*
0%	-	*0%*	*0%*	*0%*	0%	-	*0%*
0%	-	*0%*	*0%*	*0%*	0%	-	*0%*
0%	-	*0%*	*0%*	*0%*	0%	-	*0%*
0%	-	*0%*	*0%*	*0%*	0%	-	*0%*
0%	-	*0%*	*0%*	*0%*	0%	-	*0%*
0%	-	*0%*	*0%*	*0%*	0%	-	*0%*
0%	-	*0%*	*0%*	*0%*	0%	-	*0%*
0%	-	*0%*	*0%*	*0%*	0%	-	*0%*
0%	-	*0%*	*0%*	*0%*	0%	-	*0%*
0%	-	*0%*	*0%*	*0%*	0%	-	*0%*
0%	-	*0%*	*0%*	*0%*	0%	-	*0%*
0%	-	*0%*	*0%*	*0%*	0%	-	*0%*
0%	-	*0%*	*0%*	*0%*	0%	-	*0%*
0%	-	*0%*	*0%*	*0%*	0%	-	*0%*
0%	-	*0%*	*0%*	*0%*	0%	-	*0%*
0%	-	*0%*	*0%*	*0%*	0%	-	*0%*
0%	-	*0%*	*0%*	*0%*	0%	-	*0%*
0%	-	*0%*	*0%*	*0%*	0%	-	*0%*
0%	-	*0%*	*0%*	*0%*	0%	-	*0%*
0%	-	*0%*	*0%*	*0%*	0%	-	*0%*
0%	-	*0%*	*0%*	*0%*	0%	-	*0%*
0%	-	*0%*	*0%*	*0%*	0%	-	*0%*
0%	-	*0%*	*0%*	*0%*	0%	-	*0%*
0%	-	*0%*	*0%*	*0%*	0%	-	*0%*
0%	-	*0%*	*0%*	*0%*	0%	-	*0%*
0%	-	*0%*	*0%*	*0%*	0%	-	*0%*
0%	-	*0%*	*0%*	*0%*	0%	-	*0%*
100%	4,00	*100%*	*0%*	*0%*	0%	-	*0%*
0%	-	*0%*	*0%*	*0%*	0%	-	*0%*
0%	-	*0%*	*0%*	*0%*	0%	-	*0%*
0%	-	*0%*	*0%*	*0%*	0%	-	*0%*
0%	-	*0%*	*0%*	*0%*	0%	-	*0%*
0%	-	*0%*	*0%*	*0%*	0%	-	*0%*
0%	-	*0%*	*0%*	*0%*	60%	-	*100%*
0%	-	*0%*	*0%*	*0%*	0%	-	*0%*
0%	-	*0%*	*0%*	*0%*	0%	-	*0%*
0%	-	*0%*	*0%*	*0%*	0%	-	*0%*
0%	-	*0%*	*0%*	*0%*	0%	-	*0%*
0%	-	*0%*	*0%*	*0%*	0%	-	*0%*
0%	-	*0%*	*0%*	*0%*	0%	-	*0%*
0%	-	*0%*	*0%*	*0%*	0%	-	*0%*
0%	-	*0%*	*0%*	*0%*	0%	-	*0%*
0%	-	*0%*	*0%*	*0%*	0%	-	*0%*
0%	-	*0%*	*0%*	*0%*	0%	-	*0%*
0%	-	*0%*	*0%*	*0%*	0%	-	*0%*
0%	-	*0%*	*0%*	*0%*	0%	-	*0%*
0%	-	*0%*	*0%*	*0%*	0%	-	*0%*
0%	-	*0%*	*0%*	*0%*	0%	-	*0%*
0%	-	*0%*	*0%*	*0%*	0%	-	*0%*
0%	-	*0%*	*0%*	*0%*	0%	-	*0%*
0%	-	*0%*	*0%*	*0%*	0%	-	*0%*
0%	-	*0%*	*0%*	*0%*	0%	-	*0%*
0%	-	*0%*	*0%*	*0%*	0%	-	*0%*
0%	-	*0%*	*0%*	*0%*	0%	-	*0%*
0%	-	*0%*	*0%*	*0%*	0%	-	*0%*
0%	-	*0%*	*0%*	*0%*	0%	-	*0%*
0%	-	*0%*	*0%*	*0%*	0%	-	*0%*
0%	-	*0%*	*0%*	*0%*	0%	-	*0%*
0%	-	*0%*	*0%*	*0%*	0%	-	*0%*
0%	-	*0%*	*0%*	*0%*	0%	-	*0%*
0%	-	*0%*	*0%*	*0%*	0%	-	*0%*
0%	-	*0%*	*0%*	*0%*	0%	-	*0%*
0%	-	*0%*	*0%*	*0%*	0%	-	*0%*
0%	-	*0%*	*0%*	*0%*	0%	-	*0%*
0%	-	*0%*	*0%*	*0%*	0%	-	*0%*

0%	-	0%	0%	0%	0%	-	0%
0%	-	0%	0%	0%	0%	-	0%
0%	-	0%	0%	0%	0%	-	0%
0%	-	0%	0%	0%	0%	-	0%
0%	-	0%	0%	0%	0%	-	0%
0%	-	0%	0%	0%	0%	-	0%
0%	-	0%	0%	0%	0%	-	0%
100%	32,68	100%	0%	0%	0%	-	0%
0%	-	0%	0%	0%	0%	-	0%
0%	-	0%	0%	0%	0%	-	0%
0%	-	0%	0%	0%	0%	-	0%
0%	-	0%	0%	0%	0%	-	0%
0%	-	0%	0%	0%	0%	-	0%
0%	-	0%	0%	0%	0%	-	0%
0%	-	0%	0%	0%	0%	-	0%
0%	-	0%	0%	0%	0%	-	0%
0%	-	0%	0%	0%	0%	-	0%
0%	-	0%	0%	0%	0%	-	0%
0%	-	0%	0%	0%	0%	-	0%
0%	-	0%	0%	0%	0%	-	0%
0%	-	0%	0%	0%	0%	-	0%
0%	-	0%	0%	0%	0%	-	0%
0%	-	0%	0%	0%	0%	-	0%
0%	-	0%	0%	0%	0%	-	0%
0%	-	0%	0%	0%	0%	-	0%
0%	-	0%	0%	0%	0%	-	0%
0%	-	0%	0%	0%	0%	-	0%
0%	-	0%	0%	0%	0%	-	0%
0%	-	0%	0%	0%	0%	-	0%
0%	-	0%	0%	0%	0%	-	0%
0%	-	0%	0%	0%	0%	-	0%
0%	-	0%	0%	0%	0%	-	0%
0%	-	0%	0%	0%	0%	-	0%
0%	-	0%	0%	0%	0%	-	0%
0%	-	0%	0%	0%	0%	-	0%
0%	-	0%	0%	0%	0%	-	0%
0%	-	0%	0%	0%	0%	-	0%
0%	-	0%	0%	0%	0%	-	0%
0%	-	0%	0%	0%	0%	-	0%
0%	-	0%	0%	0%	0%	-	0%
0%	-	0%	0%	0%	0%	-	0%
0%	-	0%	0%	0%	0%	-	0%
0%	-	0%	0%	0%	0%	-	0%
0%	-	0%	0%	0%	0%	-	0%
0%	-	0%	0%	0%	0%	-	0%
0%	-	0%	0%	0%	0%	-	0%
0%	-	0%	0%	0%	0%	-	0%
0%	-	0%	0%	0%	0%	-	0%
0%	-	0%	0%	0%	0%	-	0%
20%	-	0%	100%	0%	0%	-	0%
0%	-	0%	0%	0%	0%	-	0%
0%	-	0%	0%	0%	0%	-	0%
0%	-	0%	0%	0%	0%	-	0%
0%	-	0%	0%	0%	0%	-	0%
0%	-	0%	0%	0%	0%	-	0%
0%	-	0%	0%	0%	0%	-	0%
0%	-	0%	0%	0%	0%	-	0%
0%	-	0%	0%	0%	0%	-	0%
0%	-	0%	0%	0%	0%	-	0%
0%	-	0%	0%	0%	0%	-	0%
0%	-	0%	0%	0%	0%	-	0%
0%	-	0%	0%	0%	0%	-	0%
0%	-	0%	0%	0%	0%	-	0%
0%	-	0%	0%	0%	0%	-	0%
0%	-	0%	0%	0%	0%	-	0%
0%	-	0%	0%	0%	0%	-	0%

0%	-	*0%*	*0%*	*0%*	0%	-	*0%*
0%	-	*0%*	*0%*	*0%*	0%	-	*0%*
0%	-	*0%*	*0%*	*0%*	0%	-	*0%*
0%	-	*0%*	*0%*	*0%*	0%	-	*0%*
0%	-	*0%*	*0%*	*0%*	0%	-	*0%*
0%	-	*0%*	*0%*	*0%*	0%	-	*0%*
0%	-	*0%*	*0%*	*0%*	0%	-	*0%*
0%	-	*0%*	*0%*	*0%*	0%	-	*0%*
0%	-	*0%*	*0%*	*0%*	0%	-	*0%*
0%	-	*0%*	*0%*	*0%*	0%	-	*0%*
0%	-	*0%*	*0%*	*0%*	0%	-	*0%*
0%	-	*0%*	*0%*	*0%*	0%	-	*0%*
0%	-	*0%*	*0%*	*0%*	0%	-	*0%*
0%	-	*0%*	*0%*	*0%*	0%	-	*0%*
0%	-	*0%*	*0%*	*0%*	0%	-	*0%*
0%	-	*0%*	*0%*	*0%*	0%	-	*0%*
0%	-	*0%*	*0%*	*0%*	0%	-	*0%*
0%	-	*0%*	*0%*	*0%*	0%	-	*0%*
0%	-	*0%*	*0%*	*0%*	0%	-	*0%*
0%	-	*0%*	*0%*	*0%*	0%	-	*0%*
0%	-	*0%*	*0%*	*0%*	0%	-	*0%*
0%	-	*0%*	*0%*	*0%*	0%	-	*0%*
0%	-	*0%*	*0%*	*0%*	0%	-	*0%*
0%	-	*0%*	*0%*	*0%*	0%	-	*0%*
0%	-	*0%*	*0%*	*0%*	0%	-	*0%*
0%	-	*0%*	*0%*	*0%*	0%	-	*0%*
0%	-	*0%*	*0%*	*0%*	0%	-	*0%*
0%	-	*0%*	*0%*	*0%*	0%	-	*0%*
0%	-	*0%*	*0%*	*0%*	0%	-	*0%*
0%	-	*0%*	*0%*	*0%*	0%	-	*0%*
0%	-	*0%*	*0%*	*0%*	0%	-	*0%*
0%	-	*0%*	*0%*	*0%*	100%	-	*100%*
0%	-	*0%*	*0%*	*0%*	0%	-	*0%*
0%	-	*0%*	*0%*	*0%*	0%	-	*0%*
0%	-	*0%*	*0%*	*0%*	0%	-	*0%*
0%	-	*0%*	*0%*	*0%*	0%	-	*0%*
0%	-	*0%*	*0%*	*0%*	0%	-	*0%*
0%	-	*0%*	*0%*	*0%*	0%	-	*0%*
0%	-	*0%*	*0%*	*0%*	0%	-	*0%*
0%	-	*0%*	*0%*	*0%*	0%	-	*0%*
0%	-	*0%*	*0%*	*0%*	0%	-	*0%*
0%	-	*0%*	*0%*	*0%*	0%	-	*0%*
0%	-	*0%*	*0%*	*0%*	0%	-	*0%*
0%	-	*0%*	*0%*	*0%*	0%	-	*0%*
0%	-	*0%*	*0%*	*0%*	0%	-	*0%*
0%	-	*0%*	*0%*	*0%*	0%	-	*0%*
0%	-	*0%*	*0%*	*0%*	0%	-	*0%*
0%	-	*0%*	*0%*	*0%*	0%	-	*0%*
0%	-	*0%*	*0%*	*0%*	0%	-	*0%*
0%	-	*0%*	*0%*	*0%*	0%	-	*0%*
0%	-	*0%*	*0%*	*0%*	0%	-	*0%*
0%	-	*0%*	*0%*	*0%*	0%	-	*0%*
0%	-	*0%*	*0%*	*0%*	0%	-	*0%*
0%	-	*0%*	*0%*	*0%*	0%	-	*0%*
0%	-	*0%*	*0%*	*0%*	0%	-	*0%*
0%	-	*0%*	*0%*	*0%*	0%	-	*0%*
0%	-	*0%*	*0%*	*0%*	0%	-	*0%*
0%	-	*0%*	*0%*	*0%*	0%	-	*0%*
0%	-	*0%*	*0%*	*0%*	0%	-	*0%*
0%	-	*0%*	*0%*	*0%*	0%	-	*0%*
0%	-	*0%*	*0%*	*0%*	0%	-	*0%*
0%	-	*0%*	*0%*	*0%*	0%	-	*0%*
0%	-	*0%*	*0%*	*0%*	0%	-	*0%*
0%	-	*0%*	*0%*	*0%*	0%	-	*0%*
0%	-	*0%*	*0%*	*0%*	0%	-	*0%*
0%	-	*0%*	*0%*	*0%*	0%	-	*0%*

0%	-	*0%*	*0%*	*0%*	0%	-	*0%*
0%	-	*0%*	*0%*	*0%*	0%	-	*0%*
0%	-	*0%*	*0%*	*0%*	0%	-	*0%*
0%	-	*0%*	*0%*	*0%*	25%	10,25	*50%*
0%	-	*0%*	*0%*	*0%*	0%	-	*0%*
0%	-	*0%*	*0%*	*0%*	0%	-	*0%*
0%	-	*0%*	*0%*	*0%*	0%	-	*0%*
0%	-	*0%*	*0%*	*0%*	41%	7,75	*100%*
0%	-	*0%*	*0%*	*0%*	30%	3,26	*100%*
0%	-	*0%*	*0%*	*0%*	0%	-	*0%*
0%	-	*0%*	*0%*	*0%*	0%	-	*0%*
0%	-	*0%*	*0%*	*0%*	0%	-	*0%*
0%	-	*0%*	*0%*	*0%*	0%	-	*0%*
0%	-	*0%*	*0%*	*0%*	0%	-	*0%*
0%	-	*0%*	*0%*	*0%*	62%	21,08	*100%*
0%	-	*0%*	*0%*	*0%*	0%	-	*0%*
20%	1,80	*100%*	*0%*	*0%*	0%	-	*0%*
0%	-	*0%*	*0%*	*0%*	0%	-	*0%*
0%	-	*0%*	*0%*	*0%*	0%	-	*0%*
0%	-	*0%*	*0%*	*0%*	0%	-	*0%*
0%	-	*0%*	*0%*	*0%*	0%	-	*0%*
28%	10,08	*100%*	*0%*	*0%*	0%	-	*0%*
30%	22,80	*100%*	*0%*	*0%*	20%	15,20	*100%*
5%	0,41	*100%*	*0%*	*0%*	0%	-	*0%*
0%	-	*0%*	*0%*	*0%*	17%	6,80	*0%*
0%	-	*0%*	*0%*	*0%*	0%	-	*0%*
0%	-	*0%*	*0%*	*0%*	0%	-	*0%*
0%	-	*0%*	*0%*	*0%*	0%	-	*0%*
0%	-	*0%*	*0%*	*0%*	0%	-	*0%*
0%	-	*0%*	*0%*	*0%*	0%	-	*0%*
0%	-	*0%*	*0%*	*0%*	0%	-	*0%*
0%	-	*0%*	*0%*	*0%*	0%	-	*0%*
0%	-	*0%*	*0%*	*0%*	0%	-	*0%*
0%	-	*0%*	*0%*	*0%*	0%	-	*0%*
0%	-	*0%*	*0%*	*0%*	0%	-	*0%*
0%	-	*0%*	*0%*	*0%*	0%	-	*0%*
0%	-	*0%*	*0%*	*0%*	0%	-	*0%*
43%	24,08	*75%*	*25%*	*0%*	0%	-	*0%*
0%	-	*0%*	*0%*	*0%*	0%	-	*0%*
0%	-	*0%*	*0%*	*0%*	0%	-	*0%*
0%	-	*0%*	*0%*	*0%*	13%	1,95	*0%*
0%	-	*0%*	*0%*	*0%*	0%	-	*0%*
0%	-	*0%*	*0%*	*0%*	15%	3,90	*0%*
0%	-	*0%*	*0%*	*0%*	0%	-	*0%*
0%	-	*0%*	*0%*	*0%*	18%	6,30	*100%*
0%	-	*0%*	*0%*	*0%*	0%	-	*0%*
0%	-	*0%*	*0%*	*0%*	0%	-	*0%*
0%	-	*0%*	*0%*	*0%*	0%	-	*0%*
0%	-	*0%*	*0%*	*0%*	0%	-	*0%*
0%	-	*0%*	*0%*	*0%*	0%	-	*0%*
0%	-	*0%*	*0%*	*0%*	0%	-	*0%*
0%	-	*0%*	*0%*	*0%*	0%	-	*0%*
0%	-	*0%*	*0%*	*0%*	0%	-	*0%*
0%	-	*0%*	*0%*	*0%*	0%	-	*0%*
0%	-	*0%*	*0%*	*0%*	0%	-	*0%*
0%	-	*0%*	*0%*	*0%*	0%	-	*0%*
0%	-	*0%*	*0%*	*0%*	0%	-	*0%*
0%	-	*0%*	*0%*	*0%*	0%	-	*0%*
0%	-	*0%*	*0%*	*0%*	0%	-	*0%*
0%	-	*0%*	*0%*	*0%*	0%	-	*0%*
0%	-	*0%*	*0%*	*0%*	0%	-	*0%*
0%	-	*0%*	*0%*	*0%*	0%	-	*0%*
0%	-	*0%*	*0%*	*0%*	0%	-	*0%*
0%	-	*0%*	*0%*	*0%*	0%	-	*0%*
0%	-	*0%*	*0%*	*0%*	0%	-	*0%*
0%	-	*0%*	*0%*	*0%*	0%	-	*0%*

0%	-	0%	0%	0%	0%	-	0%
0%	-	0%	0%	0%	0%	-	0%
0%	-	0%	0%	0%	0%	-	0%
0%	-	0%	0%	0%	0%	-	0%
0%	-	0%	0%	0%	0%	-	0%
0%	-	0%	0%	0%	0%	-	0%
0%	-	0%	0%	0%	0%	-	0%
0%	-	0%	0%	0%	0%	-	0%
0%	-	0%	0%	0%	0%	-	0%
0%	-	0%	0%	0%	0%	-	0%
0%	-	0%	0%	0%	0%	-	0%
0%	-	0%	0%	0%	0%	-	0%
0%	-	0%	0%	0%	0%	-	0%
0%	-	0%	0%	0%	0%	-	0%
0%	-	0%	0%	0%	0%	-	0%
0%	-	0%	0%	0%	0%	-	0%
0%	-	0%	0%	0%	0%	-	0%
0%	-	0%	0%	0%	0%	-	0%
0%	-	0%	0%	0%	0%	-	0%
0%	-	0%	0%	0%	0%	-	0%
0%	-	0%	0%	0%	0%	-	0%
0%	-	0%	0%	0%	0%	-	0%
0%	-	0%	0%	0%	0%	-	0%
0%	-	0%	0%	0%	0%	-	0%
0%	-	0%	0%	0%	15%	8,64	0%
0%	-	0%	0%	0%	0%	-	0%
0%	-	0%	0%	0%	0%	-	0%
0%	-	0%	0%	0%	0%	-	0%
0%	-	0%	0%	0%	10%	4,90	100%
0%	-	0%	0%	0%	0%	-	0%
0%	-	0%	0%	0%	0%	-	0%
0%	-	0%	0%	0%	0%	-	0%
0%	-	0%	0%	0%	0%	-	0%
0%	-	0%	0%	0%	0%	-	0%
0%	-	0%	0%	0%	0%	-	0%
0%	-	0%	0%	0%	0%	-	0%
0%	-	0%	0%	0%	0%	-	0%
22%	-	100%	0%	0%	13%	-	50%
0%	-	0%	0%	0%	0%	-	0%
0%	-	0%	0%	0%	0%	-	0%
0%	-	0%	0%	0%	0%	-	0%
0%	-	0%	0%	0%	0%	-	0%
0%	-	0%	0%	0%	0%	-	0%
0%	-	0%	0%	0%	0%	-	0%
0%	-	0%	0%	0%	0%	-	0%
16%	7,51	100%	0%	0%	0%	-	0%
0%	-	0%	0%	0%	30%	13,23	0%
0%	-	0%	0%	0%	0%	-	0%
0%	-	0%	0%	0%	0%	-	0%
100%	4,90	0%	100%	0%	0%	-	0%
0%	-	0%	0%	0%	0%	-	0%
0%	-	0%	0%	0%	0%	-	0%
0%	-	0%	0%	0%	0%	-	0%
0%	-	0%	0%	0%	0%	-	0%
0%	-	0%	0%	0%	0%	-	0%
0%	-	0%	0%	0%	0%	-	0%
0%	-	0%	0%	0%	0%	-	0%
0%	-	0%	0%	0%	0%	-	0%
0%	-	0%	0%	0%	0%	-	0%
0%	-	0%	0%	0%	0%	-	0%
0%	-	0%	0%	0%	0%	-	0%
0%	-	0%	0%	0%	0%	-	0%
0%	-	0%	0%	0%	0%	-	0%
0%	-	0%	0%	0%	0%	-	0%
0%	-	0%	0%	0%	0%	-	0%
0%	-	0%	0%	0%	0%	-	0%

0%	-	*0%*	*0%*	*0%*	0%	-	*0%*
0%	-	*0%*	*0%*	*0%*	0%	-	*0%*
0%	-	*0%*	*0%*	*0%*	0%	-	*0%*
0%	-	*0%*	*0%*	*0%*	0%	-	*0%*
0%	-	*0%*	*0%*	*0%*	0%	-	*0%*
0%	-	*0%*	*0%*	*0%*	0%	-	*0%*
0%	-	*0%*	*0%*	*0%*	0%	-	*0%*
0%	-	*0%*	*0%*	*0%*	0%	-	*0%*
0%	-	*0%*	*0%*	*0%*	0%	-	*0%*
0%	-	*0%*	*0%*	*0%*	0%	-	*0%*
0%	-	*0%*	*0%*	*0%*	0%	-	*0%*
0%	-	*0%*	*0%*	*0%*	0%	-	*0%*
0%	-	*0%*	*0%*	*0%*	0%	-	*0%*
0%	-	*0%*	*0%*	*0%*	0%	-	*0%*
0%	-	*0%*	*0%*	*0%*	0%	-	*0%*
0%	-	*0%*	*0%*	*0%*	0%	-	*0%*
0%	-	*0%*	*0%*	*0%*	0%	-	*0%*
0%	-	*0%*	*0%*	*0%*	0%	-	*0%*
0%	-	*0%*	*0%*	*0%*	0%	-	*0%*
0%	-	*0%*	*0%*	*0%*	0%	-	*0%*
0%	-	*0%*	*0%*	*0%*	0%	-	*0%*
0%	-	*0%*	*0%*	*0%*	0%	-	*0%*
0%	-	*0%*	*0%*	*0%*	0%	-	*0%*
0%	-	*0%*	*0%*	*0%*	0%	-	*0%*
0%	-	*0%*	*0%*	*0%*	0%	-	*0%*
0%	-	*0%*	*0%*	*0%*	0%	-	*0%*
0%	-	*0%*	*0%*	*0%*	0%	-	*0%*
0%	-	*0%*	*0%*	*0%*	0%	-	*0%*
0%	-	*0%*	*0%*	*0%*	0%	-	*0%*
0%	-	*0%*	*0%*	*0%*	0%	-	*0%*
0%	-	*0%*	*0%*	*0%*	0%	-	*0%*
0%	-	*0%*	*0%*	*0%*	0%	-	*0%*
0%	-	*0%*	*0%*	*0%*	0%	-	*0%*
0%	-	*0%*	*0%*	*0%*	0%	-	*0%*
0%	-	*0%*	*0%*	*0%*	0%	-	*0%*
0%	-	*0%*	*0%*	*0%*	0%	-	*0%*
0%	-	*0%*	*0%*	*0%*	0%	-	*0%*
0%	-	*0%*	*0%*	*0%*	0%	-	*0%*
0%	-	*0%*	*0%*	*0%*	0%	-	*0%*
0%	-	*0%*	*0%*	*0%*	0%	-	*0%*
0%	-	*0%*	*0%*	*0%*	0%	-	*0%*
0%	-	*0%*	*0%*	*0%*	0%	-	*0%*
0%	-	*0%*	*0%*	*0%*	0%	-	*0%*
0%	-	*0%*	*0%*	*0%*	0%	-	*0%*
0%	-	*0%*	*0%*	*0%*	34%	10,88	*100%*
0%	-	*0%*	*0%*	*0%*	0%	-	*0%*
0%	-	*0%*	*0%*	*0%*	0%	-	*0%*
0%	-	*0%*	*0%*	*0%*	0%	-	*0%*
15%	1,80	*100%*	*0%*	*0%*	0%	-	*0%*
0%	-	*0%*	*0%*	*0%*	0%	-	*0%*
0%	-	*0%*	*0%*	*0%*	0%	-	*0%*
0%	-	*0%*	*0%*	*0%*	0%	-	*0%*
0%	-	*0%*	*0%*	*0%*	0%	-	*0%*
0%	-	*0%*	*0%*	*0%*	0%	-	*0%*
0%	-	*0%*	*0%*	*0%*	100%	14,00	*50%*
0%	-	*0%*	*0%*	*0%*	0%	-	*0%*
0%	-	*0%*	*0%*	*0%*	46%	25,76	*18%*
0%	-	*0%*	*0%*	*0%*	0%	-	*0%*
0%	-	*0%*	*0%*	*0%*	0%	-	*0%*
0%	-	*0%*	*0%*	*0%*	0%	-	*0%*
0%	-	*0%*	*0%*	*0%*	0%	-	*0%*
0%	-	*0%*	*0%*	*0%*	0%	-	*0%*
0%	-	*0%*	*0%*	*0%*	0%	-	*0%*
0%	-	*0%*	*0%*	*0%*	0%	-	*0%*
0%	-	*0%*	*0%*	*0%*	0%	-	*0%*
0%	-	*0%*	*0%*	*0%*	0%	-	*0%*

0%	-	0%	0%	0%	0%	-	0%
0%	-	0%	0%	0%	0%	-	0%
0%	-	0%	0%	0%	0%	-	0%
0%	-	0%	0%	0%	0%	-	0%
0%	-	0%	0%	0%	0%	-	0%
0%	-	0%	0%	0%	0%	-	0%
0%	-	0%	0%	0%	0%	-	0%
0%	-	0%	0%	0%	0%	-	0%
0%	-	0%	0%	0%	0%	-	0%
0%	-	0%	0%	0%	0%	-	0%
0%	-	0%	0%	0%	0%	-	0%
0%	-	0%	0%	0%	0%	-	0%
0%	-	0%	0%	0%	0%	-	0%
0%	-	0%	0%	0%	0%	-	0%
0%	-	0%	0%	0%	0%	-	0%
0%	-	0%	0%	0%	0%	-	0%
0%	-	0%	0%	0%	0%	-	0%
0%	-	0%	0%	0%	0%	-	0%
0%	-	0%	0%	0%	0%	-	0%
0%	-	0%	0%	0%	0%	-	0%
0%	-	0%	0%	0%	0%	-	0%
0%	-	0%	0%	0%	0%	-	0%
0%	-	0%	0%	0%	0%	-	0%
0%	-	0%	0%	0%	0%	-	0%
0%	-	0%	0%	0%	0%	-	0%
0%	-	0%	0%	0%	0%	-	0%
0%	-	0%	0%	0%	0%	-	0%
0%	-	0%	0%	0%	0%	-	0%
0%	-	0%	0%	0%	0%	-	0%
0%	-	0%	0%	0%	0%	-	0%
0%	-	0%	0%	0%	0%	-	0%
0%	-	0%	0%	0%	0%	-	0%
0%	-	0%	0%	0%	0%	-	0%
0%	-	0%	0%	0%	0%	-	0%
0%	-	0%	0%	0%	0%	-	0%
0%	-	0%	0%	0%	0%	-	0%
0%	-	0%	0%	0%	0%	-	0%
0%	-	0%	0%	0%	0%	-	0%
0%	-	0%	0%	0%	0%	-	0%
0%	-	0%	0%	0%	0%	-	0%
0%	-	0%	0%	0%	0%	-	0%
0%	-	0%	0%	0%	0%	-	0%
0%	-	0%	0%	0%	0%	-	0%
0%	-	0%	0%	0%	0%	-	0%
0%	-	0%	0%	0%	0%	-	0%
0%	-	0%	0%	0%	0%	-	0%
0%	-	0%	0%	0%	0%	-	0%
0%	-	0%	0%	0%	0%	-	0%
0%	-	0%	0%	0%	0%	-	0%
0%	-	0%	0%	0%	0%	-	0%
0%	-	0%	0%	0%	0%	-	0%
0%	-	0%	0%	0%	0%	-	0%
0%	-	0%	0%	0%	0%	-	0%
0%	-	0%	0%	0%	0%	-	0%
0%	-	0%	0%	0%	0%	-	0%
0%	-	0%	0%	0%	0%	-	0%
0%	-	0%	0%	0%	12%	6,00	100%
0%	-	0%	0%	0%	0%	-	0%
0%	-	0%	0%	0%	0%	-	0%
0%	-	0%	0%	0%	0%	-	0%
0%	-	0%	0%	0%	0%	-	0%
0%	-	0%	0%	0%	0%	-	0%
0%	-	0%	0%	0%	0%	-	0%
0%	-	0%	0%	0%	0%	-	0%
0%	-	0%	0%	0%	0%	-	0%
0%	-	0%	0%	0%	31%	11,00	0%

0%	-	0%	0%	0%	0%	-	0%
0%	-	0%	0%	0%	0%	-	0%
0%	-	0%	0%	0%	0%	-	0%
0%	-	0%	0%	0%	0%	-	0%
0%	-	0%	0%	0%	0%	-	0%
0%	-	0%	0%	0%	0%	-	0%
0%	-	0%	0%	0%	0%	-	0%
0%	-	0%	0%	0%	0%	-	0%
0%	-	0%	0%	0%	0%	-	0%
0%	-	0%	0%	0%	0%	-	0%
0%	-	0%	0%	0%	0%	-	0%
0%	-	0%	0%	0%	0%	-	0%
0%	-	0%	0%	0%	0%	-	0%
0%	-	0%	0%	0%	0%	-	0%
0%	-	0%	0%	0%	0%	-	0%
0%	-	0%	0%	0%	0%	-	0%
0%	-	0%	0%	0%	0%	-	0%
0%	-	0%	0%	0%	0%	-	0%
0%	-	0%	0%	0%	0%	-	0%
0%	-	0%	0%	0%	0%	-	0%
0%	-	0%	0%	0%	0%	-	0%
0%	-	0%	0%	0%	0%	-	0%
0%	-	0%	0%	0%	0%	-	0%
0%	-	0%	0%	0%	0%	-	0%
0%	-	0%	0%	0%	0%	-	0%
0%	-	0%	0%	0%	0%	-	0%
0%	-	0%	0%	0%	0%	-	0%
0%	-	0%	0%	0%	0%	-	0%
0%	-	0%	0%	0%	0%	-	0%
0%	-	0%	0%	0%	0%	-	0%
0%	-	0%	0%	0%	0%	-	0%
0%	-	0%	0%	0%	0%	-	0%
0%	-	0%	0%	0%	0%	-	0%
0%	-	0%	0%	0%	0%	-	0%
0%	-	0%	0%	0%	0%	-	0%
0%	-	0%	0%	0%	0%	-	0%
0%	-	0%	0%	0%	0%	-	0%
0%	-	0%	0%	0%	0%	-	0%
0%	-	0%	0%	0%	0%	-	0%
0%	-	0%	0%	0%	0%	-	0%
0%	-	0%	0%	0%	0%	-	0%
0%	-	0%	0%	0%	0%	-	0%
0%	-	0%	0%	0%	0%	-	0%
0%	-	0%	0%	0%	0%	-	0%
0%	-	0%	0%	0%	0%	-	0%
0%	-	0%	0%	0%	0%	-	0%
0%	-	0%	0%	0%	0%	-	0%
0%	-	0%	0%	0%	0%	-	0%
0%	-	0%	0%	0%	0%	-	0%
0%	-	0%	0%	0%	0%	-	0%
0%	-	0%	0%	0%	0%	-	0%
0%	-	0%	0%	0%	0%	-	0%
0%	-	0%	0%	0%	0%	-	0%
0%	-	0%	0%	0%	0%	-	0%
0%	-	0%	0%	0%	0%	-	0%
0%	-	0%	0%	0%	0%	-	0%
0%	-	0%	0%	0%	0%	-	0%
0%	-	0%	0%	0%	0%	-	0%
0%	-	0%	0%	0%	0%	-	0%
0%	-	0%	0%	0%	0%	-	0%
0%	-	0%	0%	0%	0%	-	0%
0%	-	0%	0%	0%	0%	-	0%
0%	-	0%	0%	0%	0%	-	0%
0%	-	0%	0%	0%	0%	-	0%
0%	-	0%	0%	0%	0%	-	0%
0%	-	0%	0%	0%	0%	-	0%

0%	-	*0%*	*0%*	*0%*	0%	-	*0%*
0%	-	*0%*	*0%*	*0%*	0%	-	*0%*
0%	-	*0%*	*0%*	*0%*	0%	-	*0%*
0%	-	*0%*	*0%*	*0%*	0%	-	*0%*
0%	-	*0%*	*0%*	*0%*	0%	-	*0%*
0%	-	*0%*	*0%*	*0%*	0%	-	*0%*
0%	-	*0%*	*0%*	*0%*	0%	-	*0%*
0%	-	*0%*	*0%*	*0%*	0%	-	*0%*
0%	-	*0%*	*0%*	*0%*	0%	-	*0%*
0%	-	*0%*	*0%*	*0%*	0%	-	*0%*
0%	-	*0%*	*0%*	*0%*	0%	-	*0%*
0%	-	*0%*	*0%*	*0%*	0%	-	*0%*
0%	-	*0%*	*0%*	*0%*	0%	-	*0%*
0%	-	*0%*	*0%*	*0%*	0%	-	*0%*
0%	-	*0%*	*0%*	*0%*	0%	-	*0%*
0%	-	*0%*	*0%*	*0%*	0%	-	*0%*
0%	-	*0%*	*0%*	*0%*	0%	-	*0%*
0%	-	*0%*	*0%*	*0%*	0%	-	*0%*
0%	-	*0%*	*0%*	*0%*	0%	-	*0%*
0%	-	*0%*	*0%*	*0%*	0%	-	*0%*
0%	-	*0%*	*0%*	*0%*	0%	-	*0%*
0%	-	*0%*	*0%*	*0%*	0%	-	*0%*
0%	-	*0%*	*0%*	*0%*	0%	-	*0%*
0%	-	*0%*	*0%*	*0%*	0%	-	*0%*
0%	-	*0%*	*0%*	*0%*	0%	-	*0%*
0%	-	*0%*	*0%*	*0%*	0%	-	*0%*
0%	-	*0%*	*0%*	*0%*	0%	-	*0%*
0%	-	*0%*	*0%*	*0%*	0%	-	*0%*
0%	-	*0%*	*0%*	*0%*	0%	-	*0%*
0%	-	*0%*	*0%*	*0%*	0%	-	*0%*
0%	-	*0%*	*0%*	*0%*	0%	-	*0%*
0%	-	*0%*	*0%*	*0%*	0%	-	*0%*
0%	-	*0%*	*0%*	*0%*	0%	-	*0%*
0%	-	*0%*	*0%*	*0%*	0%	-	*0%*
0%	-	*0%*	*0%*	*0%*	0%	-	*0%*
0%	-	*0%*	*0%*	*0%*	29%	4,06	*0%*
0%	-	*0%*	*0%*	*0%*	0%	-	*0%*
0%	-	*0%*	*0%*	*0%*	0%	-	*0%*
0%	-	*0%*	*0%*	*0%*	0%	-	*0%*
0%	-	*0%*	*0%*	*0%*	5%	1,70	*100%*
0%	-	*0%*	*0%*	*0%*	35%	12,25	*50%*
0%	-	*0%*	*0%*	*0%*	26%	5,00	*100%*
0%	-	*0%*	*0%*	*0%*	0%	-	*0%*
0%	-	*0%*	*0%*	*0%*	100%	9,00	*100%*
0%	-	*0%*	*0%*	*0%*	0%	-	*0%*
0%	-	*0%*	*0%*	*0%*	0%	-	*0%*
0%	-	*0%*	*0%*	*0%*	0%	-	*0%*
0%	-	*0%*	*0%*	*0%*	0%	-	*0%*
0%	-	*0%*	*0%*	*0%*	0%	-	*0%*
0%	-	*0%*	*0%*	*0%*	0%	-	*0%*
0%	-	*0%*	*0%*	*0%*	0%	-	*0%*
0%	-	*0%*	*0%*	*0%*	0%	-	*0%*
0%	-	*0%*	*0%*	*0%*	0%	-	*0%*
0%	-	*0%*	*0%*	*0%*	0%	-	*0%*
0%	-	*0%*	*0%*	*0%*	0%	-	*0%*
0%	-	*0%*	*0%*	*0%*	0%	-	*0%*
0%	-	*0%*	*0%*	*0%*	0%	-	*0%*
0%	-	*0%*	*0%*	*0%*	20%	19,20	*100%*
0%	-	*0%*	*0%*	*0%*	0%	-	*0%*
0%	-	*0%*	*0%*	*0%*	0%	-	*0%*
0%	-	*0%*	*0%*	*0%*	0%	-	*0%*
0%	-	*0%*	*0%*	*0%*	0%	-	*0%*
0%	-	*0%*	*0%*	*0%*	0%	-	*0%*
0%	-	*0%*	*0%*	*0%*	0%	-	*0%*
0%	-	*0%*	*0%*	*0%*	0%	-	*0%*
0%	-	*0%*	*0%*	*0%*	0%	-	*0%*

0%	-	*0%*	*0%*	*0%*	6%	1,62	*0%*
0%	-	*0%*	*0%*	*0%*	0%	-	*0%*
0%	-	*0%*	*0%*	*0%*	100%	3,43	*100%*
0%	-	*0%*	*0%*	*0%*	0%	-	*0%*
0%	-	*0%*	*0%*	*0%*	0%	-	*0%*
0%	-	*0%*	*0%*	*0%*	0%	-	*0%*
0%	-	*0%*	*0%*	*0%*	15%	12,38	*40%*
0%	-	*0%*	*0%*	*0%*	0%	-	*0%*
0%	-	*0%*	*0%*	*0%*	0%	-	*0%*
0%	-	*0%*	*0%*	*0%*	0%	-	*0%*
0%	-	*0%*	*0%*	*0%*	0%	-	*0%*
0%	-	*0%*	*0%*	*0%*	0%	-	*0%*
0%	-	*0%*	*0%*	*0%*	0%	-	*0%*
0%	-	*0%*	*0%*	*0%*	0%	-	*0%*
0%	-	*0%*	*0%*	*0%*	0%	-	*0%*
0%	-	*0%*	*0%*	*0%*	0%	-	*0%*
0%	-	*0%*	*0%*	*0%*	0%	-	*0%*
0%	-	*0%*	*0%*	*0%*	0%	-	*0%*
0%	-	*0%*	*0%*	*0%*	0%	-	*0%*
0%	-	*0%*	*0%*	*0%*	0%	-	*0%*
0%	-	*0%*	*0%*	*0%*	0%	-	*0%*
0%	-	*0%*	*0%*	*0%*	0%	-	*0%*
0%	-	*0%*	*0%*	*0%*	0%	-	*0%*
0%	-	*0%*	*0%*	*0%*	0%	-	*0%*
0%	-	*0%*	*0%*	*0%*	0%	-	*0%*
0%	-	*0%*	*0%*	*0%*	0%	-	*0%*
0%	-	*0%*	*0%*	*0%*	0%	-	*0%*
0%	-	*0%*	*0%*	*0%*	0%	-	*0%*
0%	-	*0%*	*0%*	*0%*	4%	1,64	*100%*
0%	-	*0%*	*0%*	*0%*	0%	-	*0%*
0%	-	*0%*	*0%*	*0%*	0%	-	*0%*
0%	-	*0%*	*0%*	*0%*	0%	-	*0%*
0%	-	*0%*	*0%*	*0%*	0%	-	*0%*
0%	-	*0%*	*0%*	*0%*	0%	-	*0%*
0%	-	*0%*	*0%*	*0%*	0%	-	*0%*
0%	-	*0%*	*0%*	*0%*	0%	-	*0%*
0%	-	*0%*	*0%*	*0%*	0%	-	*0%*
0%	-	*0%*	*0%*	*0%*	0%	-	*0%*
0%	-	*0%*	*0%*	*0%*	0%	-	*0%*
0%	-	*0%*	*0%*	*0%*	0%	-	*0%*
0%	-	*0%*	*0%*	*0%*	0%	-	*0%*
0%	-	*0%*	*0%*	*0%*	0%	-	*0%*
0%	-	*0%*	*0%*	*0%*	0%	-	*0%*
0%	-	*0%*	*0%*	*0%*	25%	3,50	*100%*
0%	-	*0%*	*0%*	*0%*	0%	-	*0%*
0%	-	*0%*	*0%*	*0%*	0%	-	*0%*
0%	-	*0%*	*0%*	*0%*	0%	-	*0%*
0%	-	*0%*	*0%*	*0%*	0%	-	*0%*
0%	-	*0%*	*0%*	*0%*	0%	-	*0%*
0%	-	*0%*	*0%*	*0%*	0%	-	*0%*
0%	-	*0%*	*0%*	*0%*	0%	-	*0%*
0%	-	*0%*	*0%*	*0%*	0%	-	*0%*
0%	-	*0%*	*0%*	*0%*	0%	-	*0%*
0%	-	*0%*	*0%*	*0%*	0%	-	*0%*
0%	-	*0%*	*0%*	*0%*	0%	-	*0%*
0%	-	*0%*	*0%*	*0%*	0%	-	*0%*
0%	-	*0%*	*0%*	*0%*	0%	-	*0%*
0%	-	*0%*	*0%*	*0%*	0%	-	*0%*
0%	-	*0%*	*0%*	*0%*	0%	-	*0%*
0%	-	*0%*	*0%*	*0%*	0%	-	*0%*
0%	-	*0%*	*0%*	*0%*	0%	-	*0%*
0%	-	*0%*	*0%*	*0%*	0%	-	*0%*
0%	-	*0%*	*0%*	*0%*	0%	-	*0%*
0%	-	*0%*	*0%*	*0%*	0%	-	*0%*
0%	-	*0%*	*0%*	*0%*	0%	-	*0%*
0%	-	*0%*	*0%*	*0%*	0%	-	*0%*
0%	-	*0%*	*0%*	*0%*	0%	-	*0%*

0%	-	0%	0%	0%	0%	-	0%
0%	-	0%	0%	0%	0%	-	0%
0%	-	0%	0%	0%	0%	-	0%
0%	-	0%	0%	0%	0%	-	0%
0%	-	0%	0%	0%	0%	-	0%
0%	-	0%	0%	0%	0%	-	0%
0%	-	0%	0%	0%	0%	-	0%
0%	-	0%	0%	0%	0%	-	0%
0%	-	0%	0%	0%	0%	-	0%
0%	-	0%	0%	0%	0%	-	0%
0%	-	0%	0%	0%	0%	-	0%
0%	-	0%	0%	0%	0%	-	0%
0%	-	0%	0%	0%	0%	-	0%
0%	-	0%	0%	0%	0%	-	0%
0%	-	0%	0%	0%	0%	-	0%
0%	-	0%	0%	0%	0%	-	0%
0%	-	0%	0%	0%	0%	-	0%
0%	-	0%	0%	0%	0%	-	0%
0%	-	0%	0%	0%	25%	7,00	100%
0%	-	0%	0%	0%	0%	-	0%
0%	-	0%	0%	0%	0%	-	0%
0%	-	0%	0%	0%	0%	-	0%
0%	-	0%	0%	0%	0%	-	0%
0%	-	0%	0%	0%	0%	-	0%
0%	-	0%	0%	0%	0%	-	0%
0%	-	0%	0%	0%	0%	-	0%
0%	-	0%	0%	0%	0%	-	0%
0%	-	0%	0%	0%	0%	-	0%
0%	-	0%	0%	0%	0%	-	0%
0%	-	0%	0%	0%	95%	-	0%
0%	-	0%	0%	0%	8%	1,90	100%
0%	-	0%	0%	0%	0%	-	0%
0%	-	0%	0%	0%	0%	-	0%
0%	-	0%	0%	0%	0%	-	0%
0%	-	0%	0%	0%	0%	-	0%
0%	-	0%	0%	0%	0%	-	0%
0%	-	0%	0%	0%	0%	-	0%
0%	-	0%	0%	0%	0%	-	0%
0%	-	0%	0%	0%	0%	-	0%
0%	-	0%	0%	0%	0%	-	0%
0%	-	0%	0%	0%	100%	7,30	0%
0%	-	0%	0%	0%	0%	-	0%
0%	-	0%	0%	0%	0%	-	0%
0%	-	0%	0%	0%	0%	-	0%
0%	-	0%	0%	0%	0%	-	0%
0%	-	0%	0%	0%	0%	-	0%
0%	-	0%	0%	0%	0%	-	0%
0%	-	0%	0%	0%	0%	-	0%
0%	-	0%	0%	0%	0%	-	0%
0%	-	0%	0%	0%	55%	8,25	15%
0%	-	0%	0%	0%	16%	9,12	100%
0%	-	0%	0%	0%	0%	-	0%
0%	-	0%	0%	0%	0%	-	0%
0%	-	0%	0%	0%	0%	-	0%
0%	-	0%	0%	0%	0%	-	0%
0%	-	0%	0%	0%	0%	-	0%
0%	-	0%	0%	0%	0%	-	0%
0%	-	0%	0%	0%	0%	-	0%
0%	-	0%	0%	0%	0%	-	0%
0%	-	0%	0%	0%	0%	-	0%
0%	-	0%	0%	0%	0%	-	0%
0%	-	0%	0%	0%	0%	-	0%
0%	-	0%	0%	0%	0%	-	0%
0%	-	0%	0%	0%	0%	-	0%
0%	-	0%	0%	0%	35%	22,00	100%
0%	-	0%	0%	0%	0%	-	0%

0%	-	*0%*	*0%*	*0%*	0%	-	*0%*
0%	-	*0%*	*0%*	*0%*	0%	-	*0%*
0%	-	*0%*	*0%*	*0%*	0%	-	*0%*
0%	-	*0%*	*0%*	*0%*	0%	-	*0%*
0%	-	*0%*	*0%*	*0%*	0%	-	*0%*
0%	-	*0%*	*0%*	*0%*	0%	-	*0%*
0%	-	*0%*	*0%*	*0%*	0%	-	*0%*
0%	-	*0%*	*0%*	*0%*	0%	-	*0%*
0%	-	*0%*	*0%*	*0%*	0%	-	*0%*
0%	-	*0%*	*0%*	*0%*	0%	-	*0%*
0%	-	*0%*	*0%*	*0%*	0%	-	*0%*
0%	-	*0%*	*0%*	*0%*	0%	-	*0%*
0%	-	*0%*	*0%*	*0%*	0%	-	*0%*
0%	-	*0%*	*0%*	*0%*	0%	-	*0%*
0%	-	*0%*	*0%*	*0%*	0%	-	*0%*
0%	-	*0%*	*0%*	*0%*	0%	-	*0%*
0%	-	*0%*	*0%*	*0%*	0%	-	*0%*
0%	-	*0%*	*0%*	*0%*	0%	-	*0%*
0%	-	*0%*	*0%*	*0%*	0%	-	*0%*
0%	-	*0%*	*0%*	*0%*	0%	-	*0%*
0%	-	*0%*	*0%*	*0%*	0%	-	*0%*
0%	-	*0%*	*0%*	*0%*	0%	-	*0%*
0%	-	*0%*	*0%*	*0%*	0%	-	*0%*
0%	-	*0%*	*0%*	*0%*	0%	-	*0%*
0%	-	*0%*	*0%*	*0%*	0%	-	*0%*
0%	-	*0%*	*0%*	*0%*	0%	-	*0%*

% Neutral	% Coral wins	Dictyota % of interaction	Total length in interaction with Dictyota	% Algae win	% Neutral	% Coral wins
0%	0%	0%	-	0%	0%	0%
0%	0%	10%	-	0%	100%	0%
0%	0%	0%	-	0%	0%	0%
0%	0%	0%	-	0%	0%	0%
0%	0%	100%	-	50%	50%	0%
0%	0%	0%	-	0%	0%	0%
0%	0%	0%	-	0%	0%	0%
0%	0%	0%	-	0%	0%	0%
0%	0%	0%	-	0%	0%	0%
0%	0%	0%	-	0%	0%	0%
0%	0%	0%	-	0%	0%	0%
0%	0%	0%	-	0%	0%	0%
0%	0%	0%	-	0%	0%	0%
0%	0%	0%	-	0%	0%	0%
0%	0%	0%	-	0%	0%	0%
0%	0%	0%	-	0%	0%	0%
0%	0%	0%	-	0%	0%	0%
0%	0%	0%	-	0%	0%	0%
0%	0%	0%	-	0%	0%	0%
0%	0%	0%	-	0%	0%	0%
0%	0%	0%	-	0%	0%	0%
0%	0%	0%	-	0%	0%	0%
0%	0%	0%	-	0%	0%	0%
0%	0%	0%	-	0%	0%	0%
0%	0%	0%	-	0%	0%	0%
0%	0%	0%	-	0%	0%	0%
0%	0%	0%	-	0%	0%	0%
0%	0%	0%	-	0%	0%	0%
0%	0%	0%	-	0%	0%	0%
0%	0%	0%	-	0%	0%	0%
0%	0%	0%	-	0%	0%	0%
0%	0%	0%	-	0%	0%	0%
0%	0%	0%	-	0%	0%	0%
0%	0%	0%	-	0%	0%	0%
0%	0%	0%	-	0%	0%	0%
0%	0%	0%	-	0%	0%	0%
0%	0%	0%	-	0%	0%	0%
0%	0%	0%	-	0%	0%	0%
0%	0%	0%	-	0%	0%	0%
0%	0%	0%	-	0%	0%	0%
40%	0%	0%	-	0%	0%	0%
0%	0%	0%	-	0%	0%	0%
0%	0%	0%	-	0%	0%	0%
0%	0%	5%	1,98	80%	20%	0%
0%	0%	30%	6,12	0%	0%	100%
0%	0%	20%	3,06	30%	70%	0%
0%	0%	0%	-	0%	0%	0%
0%	0%	0%	-	0%	0%	0%
0%	0%	0%	-	0%	0%	0%
0%	0%	0%	-	0%	0%	0%
0%	0%	0%	-	0%	0%	0%
0%	0%	0%	-	0%	0%	0%
0%	0%	0%	-	0%	0%	0%
0%	0%	0%	-	0%	0%	0%
0%	0%	10%	4,23	0%	0%	100%
80%	0%	0%	-	0%	0%	0%
0%	0%	0%	-	0%	0%	0%
0%	0%	0%	-	0%	0%	0%
0%	0%	0%	-	0%	0%	0%
0%	0%	0%	-	0%	0%	0%
0%	0%	0%	-	0%	0%	0%
0%	0%	0%	-	0%	0%	0%

0%	0%	0%	-	0%	0%	0%
0%	0%	0%	-	0%	0%	0%
0%	0%	0%	-	0%	0%	0%
0%	0%	0%	-	0%	0%	0%
0%	0%	0%	-	0%	0%	0%
0%	0%	0%	-	0%	0%	0%
80%	0%	0%	-	0%	0%	0%
0%	0%	20%	5,70	0%	100%	0%
0%	0%	10%	1,12	0%	100%	0%
0%	0%	0%	-	0%	0%	0%
0%	0%	0%	-	0%	0%	0%
0%	0%	0%	-	0%	0%	0%
0%	0%	16%	0,67	0%	100%	0%
0%	0%	0%	-	0%	0%	0%
0%	0%	0%	-	0%	0%	0%
0%	0%	0%	-	0%	0%	0%
100%	0%	0%	-	0%	0%	0%
0%	0%	0%	-	0%	0%	0%
0%	0%	0%	-	0%	0%	0%
0%	0%	0%	-	0%	0%	0%
0%	0%	0%	-	0%	0%	0%
0%	0%	0%	-	0%	0%	0%
0%	0%	0%	-	0%	0%	0%
0%	0%	0%	-	0%	0%	0%
100%	0%	20%	0,77	0%	100%	0%
0%	0%	0%	-	0%	0%	0%
0%	0%	0%	-	0%	0%	0%
0%	0%	0%	-	0%	0%	0%
0%	0%	15%	1,23	0%	100%	0%
0%	0%	0%	-	0%	0%	0%
0%	0%	0%	-	0%	0%	0%
0%	0%	0%	-	0%	0%	0%
0%	0%	0%	-	0%	0%	0%
0%	0%	0%	-	0%	0%	0%
0%	0%	0%	-	0%	0%	0%
0%	0%	0%	-	0%	0%	0%
0%	0%	0%	-	0%	0%	0%
0%	0%	0%	-	0%	0%	0%
0%	0%	0%	-	0%	0%	0%
0%	0%	0%	-	0%	0%	0%
0%	0%	0%	-	0%	0%	0%
0%	0%	0%	-	0%	0%	0%
0%	0%	0%	-	0%	0%	0%
0%	0%	0%	-	0%	0%	0%
0%	0%	0%	-	0%	0%	0%
0%	0%	0%	-	0%	0%	0%
0%	0%	0%	-	0%	0%	0%
0%	0%	0%	-	0%	0%	0%
0%	0%	0%	-	0%	0%	0%
0%	0%	20%	-	50%	0%	50%
0%	0%	0%	-	0%	0%	0%
0%	0%	0%	-	0%	0%	0%
0%	0%	0%	-	0%	0%	0%
0%	0%	0%	-	0%	0%	0%
0%	0%	0%	-	0%	0%	0%
0%	0%	0%	-	0%	0%	0%
0%	0%	0%	-	0%	0%	0%
0%	0%	0%	-	0%	0%	0%
0%	0%	0%	-	0%	0%	0%
0%	0%	0%	-	0%	0%	0%
0%	0%	0%	-	0%	0%	0%
0%	0%	0%	-	0%	0%	0%
0%	0%	0%	-	0%	0%	0%
0%	0%	0%	-	0%	0%	0%
0%	0%	0%	-	0%	0%	0%
0%	0%	0%	-	0%	0%	0%

0%	*0%*	0%	-	*0%*	*0%*	*0%*
0%	*0%*	30%	2,70	*0%*	*100%*	*0%*
0%	*0%*	0%	-	*0%*	*0%*	*0%*
0%	*0%*	0%	-	*0%*	*0%*	*0%*
0%	*0%*	0%	-	*0%*	*0%*	*0%*
0%	*0%*	0%	-	*0%*	*0%*	*0%*
0%	*0%*	0%	-	*0%*	*0%*	*0%*
0%	*0%*	0%	-	*0%*	*0%*	*0%*
0%	*0%*	0%	-	*0%*	*0%*	*0%*
0%	*0%*	0%	-	*0%*	*0%*	*0%*
0%	*0%*	0%	-	*0%*	*0%*	*0%*
0%	*0%*	0%	-	*0%*	*0%*	*0%*
0%	*0%*	0%	-	*0%*	*0%*	*0%*
0%	*0%*	0%	-	*0%*	*0%*	*0%*
45%	*0%*	0%	-	*0%*	*0%*	*0%*
40%	*60%*	0%	-	*0%*	*0%*	*0%*
25%	*0%*	0%	-	*0%*	*0%*	*0%*
0%	*0%*	0%	-	*0%*	*0%*	*0%*
0%	*0%*	0%	-	*0%*	*0%*	*0%*
0%	*0%*	0%	-	*0%*	*0%*	*0%*
0%	*0%*	0%	-	*0%*	*0%*	*0%*
0%	*0%*	0%	-	*0%*	*0%*	*0%*
20%	*0%*	0%	-	*0%*	*0%*	*0%*
0%	*0%*	0%	-	*0%*	*0%*	*0%*
0%	*0%*	0%	-	*0%*	*0%*	*0%*
0%	*0%*	0%	-	*0%*	*0%*	*0%*
0%	*0%*	0%	-	*0%*	*0%*	*0%*
0%	*0%*	0%	-	*0%*	*0%*	*0%*
100%	*0%*	0%	-	*0%*	*0%*	*0%*
0%	*0%*	0%	-	*0%*	*0%*	*0%*
0%	*0%*	0%	-	*0%*	*0%*	*0%*
0%	*0%*	0%	-	*0%*	*0%*	*0%*
0%	*0%*	0%	-	*0%*	*0%*	*0%*
0%	*0%*	0%	-	*0%*	*0%*	*0%*
0%	*0%*	0%	-	*0%*	*0%*	*0%*
0%	*0%*	0%	-	*0%*	*0%*	*0%*
0%	*0%*	0%	-	*0%*	*0%*	*0%*
0%	*0%*	0%	-	*0%*	*0%*	*0%*
0%	*0%*	0%	-	*0%*	*0%*	*0%*
0%	*0%*	0%	-	*0%*	*0%*	*0%*
0%	*0%*	5%	-	*50%*	*50%*	*0%*
0%	*0%*	25%	-	*100%*	*0%*	*0%*
100%	*0%*	0%	-	*0%*	*0%*	*0%*
0%	*0%*	0%	-	*0%*	*0%*	*0%*
0%	*0%*	0%	-	*0%*	*0%*	*0%*
0%	*0%*	0%	-	*0%*	*0%*	*0%*
100%	*0%*	0%	-	*0%*	*0%*	*0%*
0%	*0%*	0%	-	*0%*	*0%*	*0%*
0%	*0%*	0%	-	*0%*	*0%*	*0%*
0%	*0%*	0%	-	*0%*	*0%*	*0%*
0%	*0%*	0%	-	*0%*	*0%*	*0%*
0%	*0%*	0%	-	*0%*	*0%*	*0%*
0%	*0%*	0%	-	*0%*	*0%*	*0%*
0%	*0%*	0%	-	*0%*	*0%*	*0%*
0%	*0%*	0%	-	*0%*	*0%*	*0%*
0%	*0%*	0%	-	*0%*	*0%*	*0%*
0%	*0%*	0%	-	*0%*	*0%*	*0%*
0%	*0%*	0%	-	*0%*	*0%*	*0%*
0%	*0%*	0%	-	*0%*	*0%*	*0%*
0%	*0%*	0%	-	*0%*	*0%*	*0%*
0%	*0%*	0%	-	*0%*	*0%*	*0%*
0%	*0%*	0%	-	*0%*	*0%*	*0%*
40%	*0%*	0%	-	*0%*	*0%*	*0%*
100%	*0%*	0%	-	*0%*	*0%*	*0%*
0%	*0%*	0%	-	*0%*	*0%*	*0%*
0%	*0%*	0%	-	*0%*	*0%*	*0%*

0%	0%	0%	-	0%	0%	0%
0%	0%	0%	-	0%	0%	0%
0%	0%	0%	-	0%	0%	0%
0%	0%	0%	-	0%	0%	0%
0%	0%	0%	-	0%	0%	0%
0%	0%	0%	-	0%	0%	0%
0%	0%	0%	-	0%	0%	0%
0%	0%	0%	-	0%	0%	0%
0%	0%	0%	-	0%	0%	0%
0%	0%	0%	-	0%	0%	0%
70%	15%	25%	25,30	15%	70%	15%
0%	0%	0%	-	0%	0%	0%
0%	0%	0%	-	0%	0%	0%
0%	0%	0%	-	0%	0%	0%
0%	0%	100%	7,04	0%	100%	0%
0%	0%	40%	-	0%	100%	0%
0%	0%	0%	-	0%	0%	0%
0%	0%	30%	-	0%	100%	0%
0%	0%	0%	-	0%	0%	0%
0%	0%	0%	-	0%	0%	0%
0%	0%	0%	-	0%	0%	0%
0%	0%	0%	-	0%	0%	0%
0%	0%	0%	-	0%	0%	0%
0%	0%	0%	-	0%	0%	0%
0%	0%	0%	-	0%	0%	0%
0%	0%	0%	-	0%	0%	0%
0%	0%	0%	-	0%	0%	0%
0%	0%	0%	-	0%	0%	0%
0%	0%	0%	-	0%	0%	0%
25%	0%	0%	-	0%	0%	0%
0%	0%	0%	-	0%	0%	0%
0%	0%	0%	-	0%	0%	0%
0%	0%	0%	-	0%	0%	0%
0%	0%	0%	-	0%	0%	0%
0%	0%	0%	-	0%	0%	0%
100%	0%	0%	-	0%	0%	0%
40%	0%	0%	-	0%	0%	0%
0%	0%	0%	-	0%	0%	0%
0%	0%	0%	-	0%	0%	0%
0%	0%	0%	-	0%	0%	0%
0%	0%	0%	-	0%	0%	0%
100%	0%	0%	-	0%	0%	0%
0%	0%	0%	-	0%	0%	0%
0%	0%	0%	-	0%	0%	0%
0%	0%	0%	-	0%	0%	0%
0%	0%	0%	-	0%	0%	0%
0%	0%	0%	-	0%	0%	0%
0%	0%	0%	-	0%	0%	0%
40%	60%	0%	-	0%	0%	0%
0%	0%	0%	-	0%	0%	0%
0%	0%	0%	-	0%	0%	0%
0%	0%	0%	-	0%	0%	0%
50%	0%	0%	-	0%	0%	0%
0%	0%	0%	-	0%	0%	0%
0%	0%	0%	-	0%	0%	0%
0%	0%	0%	-	0%	0%	0%
0%	0%	0%	-	0%	0%	0%
0%	0%	0%	-	0%	0%	0%
0%	0%	0%	-	0%	0%	0%
0%	0%	0%	-	0%	0%	0%
0%	0%	0%	-	0%	0%	0%
0%	0%	0%	-	0%	0%	0%
0%	0%	0%	-	0%	0%	0%
0%	0%	0%	-	0%	0%	0%
0%	0%	0%	-	0%	0%	0%
0%	0%	0%	-	0%	0%	0%

0%	0%	0%	-	0%	0%	0%
0%	0%	0%	-	0%	0%	0%
0%	0%	0%	-	0%	0%	0%
0%	0%	0%	-	0%	0%	0%
0%	0%	0%	-	0%	0%	0%
0%	0%	0%	-	0%	0%	0%
0%	0%	0%	-	0%	0%	0%
0%	0%	0%	-	0%	0%	0%
0%	0%	0%	-	0%	0%	0%
60%	0%	0%	-	0%	0%	0%
0%	0%	0%	-	0%	0%	0%
0%	0%	0%	-	0%	0%	0%
0%	0%	0%	-	0%	0%	0%
0%	0%	0%	-	0%	0%	0%
20%	0%	0%	-	0%	0%	0%
0%	0%	0%	-	0%	0%	0%
0%	0%	0%	-	0%	0%	0%
0%	0%	0%	-	0%	0%	0%
0%	0%	20%	4,08	0%	100%	0%
0%	0%	0%	-	0%	0%	0%
0%	0%	20%	14,70	100%	0%	0%
0%	0%	70%	11,62	15%	85%	0%
0%	0%	0%	-	0%	0%	0%
0%	0%	0%	-	0%	0%	0%
0%	0%	0%	-	0%	0%	0%
0%	0%	0%	-	0%	0%	0%
0%	0%	0%	-	0%	0%	0%
0%	0%	0%	-	0%	0%	0%
0%	0%	0%	-	0%	0%	0%
0%	0%	0%	-	0%	0%	0%
0%	0%	0%	-	0%	0%	0%
0%	0%	0%	-	0%	0%	0%
0%	0%	0%	-	0%	0%	0%
0%	0%	0%	-	0%	0%	0%
0%	0%	0%	-	0%	0%	0%
0%	0%	0%	-	0%	0%	0%
0%	0%	0%	-	0%	0%	0%
0%	0%	0%	-	0%	0%	0%
0%	0%	0%	-	0%	0%	0%
0%	0%	0%	-	0%	0%	0%
0%	0%	0%	-	0%	0%	0%
0%	0%	0%	-	0%	0%	0%
0%	0%	0%	-	0%	0%	0%
0%	0%	0%	-	0%	0%	0%
0%	0%	0%	-	0%	0%	0%
0%	0%	0%	-	0%	0%	0%
0%	0%	0%	-	0%	0%	0%
0%	0%	0%	-	0%	0%	0%
0%	0%	0%	-	0%	0%	0%
0%	0%	0%	-	0%	0%	0%
0%	0%	0%	-	0%	0%	0%
0%	0%	30%	6,30	90%	10%	0%
0%	0%	0%	-	0%	0%	0%
0%	0%	0%	-	0%	0%	0%
0%	0%	0%	-	0%	0%	0%
0%	0%	0%	-	0%	0%	0%
0%	0%	0%	-	0%	0%	0%
0%	0%	0%	-	0%	0%	0%
0%	0%	0%	-	0%	0%	0%
0%	0%	0%	-	0%	0%	0%
0%	0%	0%	-	0%	0%	0%
0%	0%	0%	-	0%	0%	0%
0%	0%	0%	-	0%	0%	0%
0%	0%	0%	-	0%	0%	0%
0%	0%	0%	-	0%	0%	0%
0%	0%	0%	-	0%	0%	0%

0%	0%	0%	-	0%	0%	0%
0%	0%	0%	-	0%	0%	0%
0%	0%	0%	-	0%	0%	0%
0%	0%	0%	-	0%	0%	0%
0%	0%	0%	-	0%	0%	0%
0%	0%	0%	-	0%	0%	0%
0%	0%	0%	-	0%	0%	0%
0%	0%	0%	-	0%	0%	0%
0%	0%	0%	-	0%	0%	0%
0%	0%	0%	-	0%	0%	0%
0%	0%	0%	-	0%	0%	0%
0%	0%	0%	-	0%	0%	0%
0%	0%	0%	-	0%	0%	0%
0%	0%	0%	-	0%	0%	0%
0%	0%	60%	42,12	30%	70%	0%
0%	0%	0%	-	0%	0%	0%
0%	0%	0%	-	0%	0%	0%
0%	0%	0%	-	0%	0%	0%
0%	0%	0%	-	0%	0%	0%
0%	0%	0%	-	0%	0%	0%
0%	0%	0%	-	0%	0%	0%
0%	0%	0%	-	0%	0%	0%
0%	0%	0%	-	0%	0%	0%
80%	0%	25%	8,50	100%	0%	0%
0%	0%	0%	-	0%	0%	0%
0%	0%	0%	-	0%	0%	0%
0%	0%	0%	-	0%	0%	0%
0%	0%	0%	-	0%	0%	0%
0%	0%	0%	-	0%	0%	0%
0%	0%	0%	-	0%	0%	0%
0%	0%	0%	-	0%	0%	0%
0%	0%	0%	-	0%	0%	0%
0%	0%	0%	-	0%	0%	0%
0%	0%	0%	-	0%	0%	0%
0%	0%	0%	-	0%	0%	0%
0%	0%	50%	7,60	20%	80%	0%
0%	0%	0%	-	0%	0%	0%
0%	0%	0%	-	0%	0%	0%
0%	0%	0%	-	0%	0%	0%
0%	0%	0%	-	0%	0%	0%
0%	0%	0%	-	0%	0%	0%
0%	0%	0%	-	0%	0%	0%
0%	0%	0%	-	0%	0%	0%
0%	0%	0%	-	0%	0%	0%
90%	0%	0%	-	0%	0%	0%
0%	0%	0%	-	0%	0%	0%
0%	0%	0%	-	0%	0%	0%
0%	0%	0%	-	0%	0%	0%
0%	0%	0%	-	0%	0%	0%
0%	0%	0%	-	0%	0%	0%
0%	0%	15%	4,08	50%	50%	0%
0%	0%	0%	-	0%	0%	0%
0%	0%	0%	-	0%	0%	0%
0%	0%	0%	-	0%	0%	0%
0%	0%	0%	-	0%	0%	0%
0%	0%	0%	-	0%	0%	0%
0%	0%	0%	-	0%	0%	0%
0%	0%	0%	-	0%	0%	0%
0%	0%	0%	-	0%	0%	0%
0%	0%	0%	-	0%	0%	0%
0%	0%	0%	-	0%	0%	0%
0%	0%	40%	4,40	100%	0%	0%
0%	0%	20%	8,20	0%	50%	50%
0%	0%	0%	-	0%	0%	0%
0%	0%	0%	-	0%	0%	0%
0%	0%	0%	-	0%	0%	0%

0%	*0%*	0%	-	*0%*	*0%*	*0%*
0%	*0%*	0%	-	*0%*	*0%*	*0%*
0%	*0%*	30%	5,94	*100%*	*0%*	*0%*
0%	*0%*	0%	-	*0%*	*0%*	*0%*
0%	*0%*	0%	-	*0%*	*0%*	*0%*
0%	*0%*	0%	-	*0%*	*0%*	*0%*
0%	*0%*	0%	-	*0%*	*0%*	*0%*
0%	*0%*	0%	-	*0%*	*0%*	*0%*
0%	*0%*	0%	-	*0%*	*0%*	*0%*
0%	*0%*	0%	-	*0%*	*0%*	*0%*
0%	*0%*	0%	-	*0%*	*0%*	*0%*
0%	*0%*	0%	-	*0%*	*0%*	*0%*
0%	*0%*	0%	-	*0%*	*0%*	*0%*
0%	*0%*	35%	5,60	*60%*	*40%*	*0%*
0%	*0%*	0%	-	*0%*	*0%*	*0%*
0%	*0%*	0%	-	*0%*	*0%*	*0%*
0%	*0%*	0%	-	*0%*	*0%*	*0%*
0%	*0%*	0%	-	*0%*	*0%*	*0%*
0%	*0%*	0%	-	*0%*	*0%*	*0%*
0%	*0%*	0%	-	*0%*	*0%*	*0%*
0%	*0%*	0%	-	*0%*	*0%*	*0%*
0%	*0%*	0%	-	*0%*	*0%*	*0%*
0%	*0%*	0%	-	*0%*	*0%*	*0%*
0%	*0%*	0%	-	*0%*	*0%*	*0%*
0%	*0%*	0%	-	*0%*	*0%*	*0%*
0%	*0%*	0%	-	*0%*	*0%*	*0%*
0%	*0%*	0%	-	*0%*	*0%*	*0%*
0%	*0%*	0%	-	*0%*	*0%*	*0%*
0%	*0%*	0%	-	*0%*	*0%*	*0%*
0%	*0%*	0%	-	*0%*	*0%*	*0%*
0%	*0%*	0%	-	*0%*	*0%*	*0%*
0%	*0%*	0%	-	*0%*	*0%*	*0%*
0%	*0%*	0%	-	*0%*	*0%*	*0%*
0%	*0%*	0%	-	*0%*	*0%*	*0%*
0%	*0%*	0%	-	*0%*	*0%*	*0%*
0%	*0%*	0%	-	*0%*	*0%*	*0%*
0%	*0%*	0%	-	*0%*	*0%*	*0%*
0%	*0%*	0%	-	*0%*	*0%*	*0%*
0%	*0%*	16%	13,76	*100%*	*0%*	*0%*
0%	*0%*	0%	-	*0%*	*0%*	*0%*
0%	*0%*	0%	-	*0%*	*0%*	*0%*
0%	*0%*	0%	-	*0%*	*0%*	*0%*
0%	*0%*	0%	-	*0%*	*0%*	*0%*
0%	*0%*	0%	-	*0%*	*0%*	*0%*
0%	*0%*	0%	-	*0%*	*0%*	*0%*
0%	*0%*	0%	-	*0%*	*0%*	*0%*
0%	*0%*	0%	-	*0%*	*0%*	*0%*
0%	*0%*	0%	-	*0%*	*0%*	*0%*
0%	*0%*	0%	-	*0%*	*0%*	*0%*
0%	*0%*	0%	-	*0%*	*0%*	*0%*
0%	*0%*	0%	-	*0%*	*0%*	*0%*
0%	*0%*	0%	-	*0%*	*0%*	*0%*
0%	*0%*	0%	-	*0%*	*0%*	*0%*
0%	*0%*	0%	-	*0%*	*0%*	*0%*
0%	*0%*	0%	-	*0%*	*0%*	*0%*
0%	*0%*	0%	-	*0%*	*0%*	*0%*
0%	*0%*	0%	-	*0%*	*0%*	*0%*
0%	*0%*	0%	-	*0%*	*0%*	*0%*
0%	*0%*	0%	-	*0%*	*0%*	*0%*
0%	*0%*	0%	-	*0%*	*0%*	*0%*
0%	*0%*	0%	-	*0%*	*0%*	*0%*
0%	*0%*	0%	-	*0%*	*0%*	*0%*
0%	*0%*	0%	-	*0%*	*0%*	*0%*
0%	*0%*	0%	-	*0%*	*0%*	*0%*
0%	*0%*	0%	-	*0%*	*0%*	*0%*
0%	*0%*	0%	-	*0%*	*0%*	*0%*

0%	*0%*	0%	-	*0%*	*0%*	*0%*
0%	*0%*	0%	-	*0%*	*0%*	*0%*
0%	*0%*	0%	-	*0%*	*0%*	*0%*
0%	*0%*	0%	-	*0%*	*0%*	*0%*
0%	*0%*	0%	-	*0%*	*0%*	*0%*
0%	*0%*	0%	-	*0%*	*0%*	*0%*
0%	*0%*	0%	-	*0%*	*0%*	*0%*
0%	*0%*	0%	-	*0%*	*0%*	*0%*
0%	*0%*	0%	-	*0%*	*0%*	*0%*
0%	*0%*	0%	-	*0%*	*0%*	*0%*
0%	*0%*	0%	-	*0%*	*0%*	*0%*
0%	*0%*	0%	-	*0%*	*0%*	*0%*
0%	*0%*	0%	-	*0%*	*0%*	*0%*
0%	*0%*	0%	-	*0%*	*0%*	*0%*
0%	*0%*	0%	-	*0%*	*0%*	*0%*
0%	*0%*	0%	-	*0%*	*0%*	*0%*
0%	*0%*	0%	-	*0%*	*0%*	*0%*
0%	*0%*	0%	-	*0%*	*0%*	*0%*
0%	*0%*	0%	-	*0%*	*0%*	*0%*
0%	*0%*	0%	-	*0%*	*0%*	*0%*
0%	*0%*	0%	-	*0%*	*0%*	*0%*
0%	*0%*	0%	-	*0%*	*0%*	*0%*
0%	*0%*	0%	-	*0%*	*0%*	*0%*
0%	*0%*	0%	-	*0%*	*0%*	*0%*
0%	*0%*	0%	-	*0%*	*0%*	*0%*
0%	*0%*	0%	-	*0%*	*0%*	*0%*
0%	*0%*	0%	-	*0%*	*0%*	*0%*
0%	*0%*	0%	-	*0%*	*0%*	*0%*
0%	*0%*	0%	-	*0%*	*0%*	*0%*
0%	*0%*	0%	-	*0%*	*0%*	*0%*
0%	*0%*	0%	-	*0%*	*0%*	*0%*
0%	*0%*	0%	-	*0%*	*0%*	*0%*
0%	*0%*	0%	-	*0%*	*0%*	*0%*
0%	*0%*	0%	-	*0%*	*0%*	*0%*
0%	*0%*	0%	-	*0%*	*0%*	*0%*
0%	*0%*	0%	-	*0%*	*0%*	*0%*
0%	*0%*	0%	-	*0%*	*0%*	*0%*
0%	*0%*	0%	-	*0%*	*0%*	*0%*
0%	*0%*	0%	-	*0%*	*0%*	*0%*
0%	*0%*	0%	-	*0%*	*0%*	*0%*
0%	*0%*	0%	-	*0%*	*0%*	*0%*
0%	*0%*	0%	-	*0%*	*0%*	*0%*
0%	*0%*	0%	-	*0%*	*0%*	*0%*
0%	*0%*	0%	-	*0%*	*0%*	*0%*
0%	*0%*	0%	-	*0%*	*0%*	*0%*
0%	*0%*	0%	-	*0%*	*0%*	*0%*
0%	*0%*	0%	-	*0%*	*0%*	*0%*
0%	*0%*	0%	-	*0%*	*0%*	*0%*
0%	*0%*	0%	-	*0%*	*0%*	*0%*
0%	*0%*	0%	-	*0%*	*0%*	*0%*
0%	*0%*	0%	-	*0%*	*0%*	*0%*
0%	*0%*	0%	-	*0%*	*0%*	*0%*
0%	*0%*	0%	-	*0%*	*0%*	*0%*
0%	*0%*	0%	-	*0%*	*0%*	*0%*
0%	*0%*	0%	-	*0%*	*0%*	*0%*
0%	*0%*	0%	-	*0%*	*0%*	*0%*
0%	*0%*	0%	-	*0%*	*0%*	*0%*
0%	*0%*	0%	-	*0%*	*0%*	*0%*
0%	*0%*	0%	-	*0%*	*0%*	*0%*
0%	*0%*	0%	-	*0%*	*0%*	*0%*
0%	*0%*	0%	-	*0%*	*0%*	*0%*
0%	*0%*	0%	-	*0%*	*0%*	*0%*
0%	*0%*	0%	-	*0%*	*0%*	*0%*
0%	*0%*	0%	-	*0%*	*0%*	*0%*
0%	*0%*	0%	-	*0%*	*0%*	*0%*
0%	*0%*	0%	-	*0%*	*0%*	*0%*

0%	*0%*	0%	-	*0%*	*0%*	*0%*
0%	*0%*	0%	-	*0%*	*0%*	*0%*
0%	*0%*	0%	-	*0%*	*0%*	*0%*
0%	*0%*	0%	-	*0%*	*0%*	*0%*
0%	*0%*	0%	-	*0%*	*0%*	*0%*
0%	*0%*	0%	-	*0%*	*0%*	*0%*
0%	*0%*	0%	-	*0%*	*0%*	*0%*
0%	*0%*	0%	-	*0%*	*0%*	*0%*
0%	*0%*	0%	-	*0%*	*0%*	*0%*
0%	*0%*	0%	-	*0%*	*0%*	*0%*
0%	*0%*	0%	-	*0%*	*0%*	*0%*
0%	*0%*	0%	-	*0%*	*0%*	*0%*
0%	*0%*	0%	-	*0%*	*0%*	*0%*
0%	*0%*	0%	-	*0%*	*0%*	*0%*
0%	*0%*	0%	-	*0%*	*0%*	*0%*
0%	*0%*	0%	-	*0%*	*0%*	*0%*
0%	*0%*	0%	-	*0%*	*0%*	*0%*
0%	*0%*	0%	-	*0%*	*0%*	*0%*
0%	*0%*	0%	-	*0%*	*0%*	*0%*
0%	*0%*	0%	-	*0%*	*0%*	*0%*
0%	*0%*	0%	-	*0%*	*0%*	*0%*
0%	*0%*	0%	-	*0%*	*0%*	*0%*
0%	*0%*	0%	-	*0%*	*0%*	*0%*
0%	*0%*	0%	-	*0%*	*0%*	*0%*
0%	*0%*	0%	-	*0%*	*0%*	*0%*
0%	*0%*	0%	-	*0%*	*0%*	*0%*
0%	*0%*	0%	-	*0%*	*0%*	*0%*
0%	*0%*	0%	-	*0%*	*0%*	*0%*
0%	*0%*	0%	-	*0%*	*0%*	*0%*
0%	*0%*	0%	-	*0%*	*0%*	*0%*
0%	*0%*	0%	-	*0%*	*0%*	*0%*
0%	*0%*	0%	-	*0%*	*0%*	*0%*
0%	*0%*	100%	3,40	*100%*	*0%*	*0%*
0%	*0%*	0%	-	*0%*	*0%*	*0%*
0%	*0%*	0%	-	*0%*	*0%*	*0%*
0%	*0%*	0%	-	*0%*	*0%*	*0%*
0%	*0%*	0%	-	*0%*	*0%*	*0%*
0%	*0%*	0%	-	*0%*	*0%*	*0%*
0%	*0%*	0%	-	*0%*	*0%*	*0%*
0%	*0%*	0%	-	*0%*	*0%*	*0%*
0%	*0%*	0%	-	*0%*	*0%*	*0%*
0%	*0%*	0%	-	*0%*	*0%*	*0%*
0%	*0%*	0%	-	*0%*	*0%*	*0%*
0%	*0%*	0%	-	*0%*	*0%*	*0%*
0%	*0%*	0%	-	*0%*	*0%*	*0%*
0%	*0%*	0%	-	*0%*	*0%*	*0%*
0%	*0%*	0%	-	*0%*	*0%*	*0%*
0%	*0%*	30%	-	*100%*	*0%*	*0%*
0%	*0%*	0%	-	*0%*	*0%*	*0%*
0%	*0%*	0%	-	*0%*	*0%*	*0%*
0%	*0%*	0%	-	*0%*	*0%*	*0%*
0%	*0%*	0%	-	*0%*	*0%*	*0%*
0%	*0%*	0%	-	*0%*	*0%*	*0%*
0%	*0%*	0%	-	*0%*	*0%*	*0%*
0%	*0%*	0%	-	*0%*	*0%*	*0%*
0%	*0%*	0%	-	*0%*	*0%*	*0%*
0%	*0%*	0%	-	*0%*	*0%*	*0%*
0%	*0%*	0%	-	*0%*	*0%*	*0%*
0%	*0%*	0%	-	*0%*	*0%*	*0%*
0%	*0%*	30%	38,76	*5%*	*10%*	*85%*
0%	*0%*	0%	-	*0%*	*0%*	*0%*
0%	*0%*	0%	-	*0%*	*0%*	*0%*
0%	*0%*	0%	-	*0%*	*0%*	*0%*
0%	*0%*	0%	-	*0%*	*0%*	*0%*
0%	*0%*	0%	-	*0%*	*0%*	*0%*
0%	*0%*	0%	-	*0%*	*0%*	*0%*

0%	0%	0%	-	0%	0%	0%
0%	0%	0%	-	0%	0%	0%
0%	0%	0%	-	0%	0%	0%
0%	0%	0%	-	0%	0%	0%
0%	0%	0%	-	0%	0%	0%
0%	0%	0%	-	0%	0%	0%
0%	0%	0%	-	0%	0%	0%
0%	0%	0%	-	0%	0%	0%
0%	0%	0%	-	0%	0%	0%
0%	0%	0%	-	0%	0%	0%
0%	0%	0%	-	0%	0%	0%
0%	0%	0%	-	0%	0%	0%
0%	0%	0%	-	0%	0%	0%
0%	0%	0%	-	0%	0%	0%
0%	0%	0%	-	0%	0%	0%
0%	0%	0%	-	0%	0%	0%
0%	0%	0%	-	0%	0%	0%
0%	0%	0%	-	0%	0%	0%
0%	0%	0%	-	0%	0%	0%
0%	0%	40%	-	65%	35%	0%
0%	0%	0%	-	0%	0%	0%
0%	0%	0%	-	0%	0%	0%
0%	0%	0%	-	0%	0%	0%
0%	0%	0%	-	0%	0%	0%
0%	0%	0%	-	0%	0%	0%
0%	0%	0%	-	0%	0%	0%
0%	0%	0%	-	0%	0%	0%
0%	0%	0%	-	0%	0%	0%
0%	0%	0%	-	0%	0%	0%
0%	0%	0%	-	0%	0%	0%
0%	0%	0%	-	0%	0%	0%
0%	0%	0%	-	0%	0%	0%
0%	0%	0%	-	0%	0%	0%
0%	0%	0%	-	0%	0%	0%
0%	0%	0%	-	0%	0%	0%
0%	0%	0%	-	0%	0%	0%
0%	0%	0%	-	0%	0%	0%
0%	0%	0%	-	0%	0%	0%
0%	0%	0%	-	0%	0%	0%
0%	0%	30%	-	60%	40%	0%
0%	0%	0%	-	0%	0%	0%
0%	0%	0%	-	0%	0%	0%
0%	0%	0%	-	0%	0%	0%
0%	0%	0%	-	0%	0%	0%
0%	0%	0%	-	0%	0%	0%
0%	0%	0%	-	0%	0%	0%
0%	0%	0%	-	0%	0%	0%
0%	0%	0%	-	0%	0%	0%
0%	0%	0%	-	0%	0%	0%
0%	0%	0%	-	0%	0%	0%
0%	0%	0%	-	0%	0%	0%
0%	0%	0%	-	0%	0%	0%
0%	0%	0%	-	0%	0%	0%
0%	0%	0%	-	0%	0%	0%
0%	0%	0%	-	0%	0%	0%
0%	0%	0%	-	0%	0%	0%
0%	0%	0%	-	0%	0%	0%
0%	0%	0%	-	0%	0%	0%
0%	0%	0%	-	0%	0%	0%
0%	0%	0%	-	0%	0%	0%
0%	0%	0%	-	0%	0%	0%
0%	0%	0%	-	0%	0%	0%
0%	0%	0%	-	0%	0%	0%
0%	0%	0%	-	0%	0%	0%
0%	0%	0%	-	0%	0%	0%
0%	0%	0%	-	0%	0%	0%

0%	0%	0%	-	0%	0%	0%
0%	0%	0%	-	0%	0%	0%
0%	0%	0%	-	0%	0%	0%
0%	0%	0%	-	0%	0%	0%
0%	0%	0%	-	0%	0%	0%
0%	0%	0%	-	0%	0%	0%
0%	0%	0%	-	0%	0%	0%
0%	0%	0%	-	0%	0%	0%
0%	0%	0%	-	0%	0%	0%
0%	0%	0%	-	0%	0%	0%
0%	0%	0%	-	0%	0%	0%
0%	0%	0%	-	0%	0%	0%
0%	0%	0%	-	0%	0%	0%
0%	0%	0%	-	0%	0%	0%
0%	0%	0%	-	0%	0%	0%
0%	0%	0%	-	0%	0%	0%
0%	0%	0%	-	0%	0%	0%
0%	0%	0%	-	0%	0%	0%
0%	0%	0%	-	0%	0%	0%
0%	0%	0%	-	0%	0%	0%
0%	0%	0%	-	0%	0%	0%
0%	0%	0%	-	0%	0%	0%
0%	0%	0%	-	0%	0%	0%
0%	0%	0%	-	0%	0%	0%
0%	0%	0%	-	0%	0%	0%
0%	0%	0%	-	0%	0%	0%
0%	0%	0%	-	0%	0%	0%
0%	0%	0%	-	0%	0%	0%
0%	0%	0%	-	0%	0%	0%
0%	0%	0%	-	0%	0%	0%
0%	0%	0%	-	0%	0%	0%
0%	0%	0%	-	0%	0%	0%
0%	0%	0%	-	0%	0%	0%
0%	0%	0%	-	0%	0%	0%
0%	0%	0%	-	0%	0%	0%
0%	0%	0%	-	0%	0%	0%
0%	0%	0%	-	0%	0%	0%
0%	0%	0%	-	0%	0%	0%
0%	0%	10%	-	100%	0%	0%
0%	0%	0%	-	0%	0%	0%
0%	0%	0%	-	0%	0%	0%
0%	0%	0%	-	0%	0%	0%
0%	0%	0%	-	0%	0%	0%
0%	0%	0%	-	0%	0%	0%
0%	0%	0%	-	0%	0%	0%
0%	0%	0%	-	0%	0%	0%
0%	0%	0%	-	0%	0%	0%
0%	0%	0%	-	0%	0%	0%
0%	0%	0%	-	0%	0%	0%
0%	0%	0%	-	0%	0%	0%
0%	0%	0%	-	0%	0%	0%
0%	0%	0%	-	0%	0%	0%
0%	0%	0%	-	0%	0%	0%
0%	0%	0%	-	0%	0%	0%
0%	0%	0%	-	0%	0%	0%
0%	0%	0%	-	0%	0%	0%
0%	0%	0%	-	0%	0%	0%
0%	0%	0%	-	0%	0%	0%
0%	0%	0%	-	0%	0%	0%
0%	0%	0%	-	0%	0%	0%
0%	0%	25%	-	100%	0%	0%
0%	0%	0%	-	0%	0%	0%
0%	0%	0%	-	0%	0%	0%
0%	0%	0%	-	0%	0%	0%
0%	0%	0%	-	0%	0%	0%
0%	0%	0%	-	0%	0%	0%

0%	*0%*	0%	-	*0%*	*0%*	*0%*
0%	*0%*	31%	11,00	*50%*	*50%*	*0%*
0%	*0%*	100%	-	*100%*	*0%*	*0%*
50%	*0%*	0%	-	*0%*	*0%*	*0%*
0%	*0%*	0%	-	*0%*	*0%*	*0%*
0%	*0%*	0%	-	*0%*	*0%*	*0%*
0%	*0%*	0%	-	*0%*	*0%*	*0%*
0%	*0%*	0%	-	*0%*	*0%*	*0%*
0%	*0%*	70%	7,60	*100%*	*0%*	*0%*
0%	*0%*	0%	-	*0%*	*0%*	*0%*
0%	*0%*	0%	-	*0%*	*0%*	*0%*
0%	*0%*	0%	-	*0%*	*0%*	*0%*
0%	*0%*	0%	-	*0%*	*0%*	*0%*
0%	*0%*	0%	-	*0%*	*0%*	*0%*
0%	*0%*	0%	-	*0%*	*0%*	*0%*
0%	*0%*	0%	-	*0%*	*0%*	*0%*
0%	*0%*	0%	-	*0%*	*0%*	*0%*
0%	*0%*	0%	-	*0%*	*0%*	*0%*
0%	*0%*	0%	-	*0%*	*0%*	*0%*
0%	*0%*	0%	-	*0%*	*0%*	*0%*
0%	*0%*	69%	93,84	*100%*	*0%*	*0%*
0%	*0%*	72%	26,00	*100%*	*0%*	*0%*
0%	*0%*	0%	-	*0%*	*0%*	*0%*
0%	*0%*	0%	-	*0%*	*0%*	*0%*
100%	*0%*	0%	-	*0%*	*0%*	*0%*
0%	*0%*	0%	-	*0%*	*0%*	*0%*
0%	*0%*	0%	-	*0%*	*0%*	*0%*
0%	*0%*	27%	13,77	*100%*	*0%*	*0%*
0%	*0%*	0%	-	*0%*	*0%*	*0%*
0%	*0%*	0%	-	*0%*	*0%*	*0%*
0%	*0%*	0%	-	*0%*	*0%*	*0%*
0%	*0%*	0%	-	*0%*	*0%*	*0%*
0%	*0%*	0%	-	*0%*	*0%*	*0%*
0%	*0%*	0%	-	*0%*	*0%*	*0%*
0%	*0%*	0%	-	*0%*	*0%*	*0%*
0%	*0%*	0%	-	*0%*	*0%*	*0%*
0%	*0%*	0%	-	*0%*	*0%*	*0%*
0%	*0%*	0%	-	*0%*	*0%*	*0%*
0%	*0%*	0%	-	*0%*	*0%*	*0%*
0%	*0%*	0%	-	*0%*	*0%*	*0%*
100%	*0%*	0%	-	*0%*	*0%*	*0%*
0%	*0%*	0%	-	*0%*	*0%*	*0%*
100%	*0%*	0%	-	*0%*	*0%*	*0%*
0%	*0%*	0%	-	*0%*	*0%*	*0%*
0%	*0%*	0%	-	*0%*	*0%*	*0%*
0%	*0%*	0%	-	*0%*	*0%*	*0%*
0%	*0%*	0%	-	*0%*	*0%*	*0%*
0%	*0%*	0%	-	*0%*	*0%*	*0%*
0%	*0%*	0%	-	*0%*	*0%*	*0%*
0%	*0%*	0%	-	*0%*	*0%*	*0%*
0%	*0%*	0%	-	*0%*	*0%*	*0%*
0%	*0%*	0%	-	*0%*	*0%*	*0%*
0%	*0%*	0%	-	*0%*	*0%*	*0%*
0%	*0%*	0%	-	*0%*	*0%*	*0%*
0%	*0%*	0%	-	*0%*	*0%*	*0%*
0%	*0%*	0%	-	*0%*	*0%*	*0%*
0%	*0%*	0%	-	*0%*	*0%*	*0%*
0%	*0%*	0%	-	*0%*	*0%*	*0%*
0%	*0%*	0%	-	*0%*	*0%*	*0%*
0%	*0%*	0%	-	*0%*	*0%*	*0%*
0%	*0%*	0%	-	*0%*	*0%*	*0%*
0%	*0%*	0%	-	*0%*	*0%*	*0%*
0%	*0%*	0%	-	*0%*	*0%*	*0%*
0%	*0%*	0%	-	*0%*	*0%*	*0%*
0%	*0%*	0%	-	*0%*	*0%*	*0%*
0%	*0%*	0%	-	*0%*	*0%*	*0%*

0%	0%	0%	-	0%	0%	0%
0%	0%	0%	-	0%	0%	0%
0%	0%	0%	-	0%	0%	0%
0%	0%	0%	-	0%	0%	0%
0%	0%	0%	-	0%	0%	0%
0%	0%	0%	-	0%	0%	0%
0%	0%	0%	-	0%	0%	0%
0%	0%	0%	-	0%	0%	0%
0%	0%	0%	-	0%	0%	0%
0%	0%	0%	-	0%	0%	0%
0%	0%	0%	-	0%	0%	0%
0%	0%	0%	-	0%	0%	0%
0%	0%	0%	-	0%	0%	0%
0%	0%	0%	-	0%	0%	0%
0%	0%	0%	-	0%	0%	0%
0%	0%	0%	-	0%	0%	0%
0%	0%	0%	-	0%	0%	0%
0%	0%	0%	-	0%	0%	0%
0%	0%	0%	-	0%	0%	0%
0%	0%	0%	-	0%	0%	0%
0%	0%	100%	12,00	0%	40%	60%
0%	0%	0%	-	0%	0%	0%
0%	0%	0%	-	0%	0%	0%
0%	0%	0%	-	0%	0%	0%
100%	0%	0%	-	0%	0%	0%
0%	0%	0%	-	0%	0%	0%
0%	0%	0%	-	0%	0%	0%
0%	0%	0%	-	0%	0%	0%
0%	0%	8%	3,92	100%	0%	0%
0%	0%	0%	-	0%	0%	0%
0%	0%	0%	-	0%	0%	0%
0%	0%	0%	-	0%	0%	0%
0%	0%	0%	-	0%	0%	0%
0%	0%	0%	-	0%	0%	0%
0%	0%	0%	-	0%	0%	0%
0%	0%	0%	-	0%	0%	0%
0%	0%	0%	-	0%	0%	0%
50%	0%	0%	-	0%	0%	0%
0%	0%	0%	-	0%	0%	0%
0%	0%	0%	-	0%	0%	0%
0%	0%	0%	-	0%	0%	0%
0%	0%	0%	-	0%	0%	0%
0%	0%	0%	-	0%	0%	0%
0%	0%	0%	-	0%	0%	0%
0%	0%	0%	-	0%	0%	0%
0%	0%	0%	-	0%	0%	0%
100%	0%	0%	-	0%	0%	0%
0%	0%	20%	5,74	100%	0%	0%
0%	0%	0%	-	0%	0%	0%
0%	0%	0%	-	0%	0%	0%
0%	0%	0%	-	0%	0%	0%
0%	0%	0%	-	0%	0%	0%
0%	0%	0%	-	0%	0%	0%
0%	0%	0%	-	0%	0%	0%
0%	0%	0%	-	0%	0%	0%
0%	0%	0%	-	0%	0%	0%
0%	0%	0%	-	0%	0%	0%
0%	0%	0%	-	0%	0%	0%
0%	0%	0%	-	0%	0%	0%
0%	0%	0%	-	0%	0%	0%
0%	0%	0%	-	0%	0%	0%
0%	0%	0%	-	0%	0%	0%
0%	0%	0%	-	0%	0%	0%
0%	0%	0%	-	0%	0%	0%
0%	0%	0%	-	0%	0%	0%
0%	0%	0%	-	0%	0%	0%

0%	0%	0%	-	0%	0%	0%
0%	0%	0%	-	0%	0%	0%
0%	0%	0%	-	0%	0%	0%
0%	0%	0%	-	0%	0%	0%
0%	0%	0%	-	0%	0%	0%
0%	0%	0%	-	0%	0%	0%
0%	0%	0%	-	0%	0%	0%
0%	0%	0%	-	0%	0%	0%
0%	0%	0%	-	0%	0%	0%
0%	0%	0%	-	0%	0%	0%
0%	0%	0%	-	0%	0%	0%
0%	0%	0%	-	0%	0%	0%
0%	0%	0%	-	0%	0%	0%
0%	0%	0%	-	0%	0%	0%
0%	0%	0%	-	0%	0%	0%
0%	0%	0%	-	0%	0%	0%
0%	0%	0%	-	0%	0%	0%
0%	0%	0%	-	0%	0%	0%
0%	0%	0%	-	0%	0%	0%
0%	0%	0%	-	0%	0%	0%
0%	0%	0%	-	0%	0%	0%
0%	0%	0%	-	0%	0%	0%
0%	0%	0%	-	0%	0%	0%
0%	0%	0%	-	0%	0%	0%
0%	0%	0%	-	0%	0%	0%
0%	0%	0%	-	0%	0%	0%
0%	0%	0%	-	0%	0%	0%
0%	0%	0%	-	0%	0%	0%
0%	0%	0%	-	0%	0%	0%
0%	0%	0%	-	0%	0%	0%
0%	0%	0%	-	0%	0%	0%
0%	0%	0%	-	0%	0%	0%
0%	0%	0%	-	0%	0%	0%
0%	0%	0%	-	0%	0%	0%
0%	0%	0%	-	0%	0%	0%
0%	0%	0%	-	0%	0%	0%
0%	0%	0%	-	0%	0%	0%
0%	0%	0%	-	0%	0%	0%
0%	0%	0%	-	0%	0%	0%
0%	0%	0%	-	0%	0%	0%
0%	0%	0%	-	0%	0%	0%
0%	0%	0%	-	0%	0%	0%
0%	0%	0%	-	0%	0%	0%
0%	0%	0%	-	0%	0%	0%
0%	0%	0%	-	0%	0%	0%
0%	0%	0%	-	0%	0%	0%
0%	0%	0%	-	0%	0%	0%
0%	0%	0%	-	0%	0%	0%
0%	0%	0%	-	0%	0%	0%
0%	0%	0%	-	0%	0%	0%
0%	0%	0%	-	0%	0%	0%
0%	0%	0%	-	0%	0%	0%
0%	0%	0%	-	0%	0%	0%
0%	0%	0%	-	0%	0%	0%
50%	0%	0%	-	0%	0%	0%
0%	0%	0%	-	0%	0%	0%
82%	0%	0%	-	0%	0%	0%
0%	0%	0%	-	0%	0%	0%
0%	0%	0%	-	0%	0%	0%
0%	0%	0%	-	0%	0%	0%
0%	0%	0%	-	0%	0%	0%
0%	0%	0%	-	0%	0%	0%
0%	0%	0%	-	0%	0%	0%
0%	0%	0%	-	0%	0%	0%
0%	0%	0%	-	0%	0%	0%
0%	0%	0%	-	0%	0%	0%

0%	*0%*	0%	-	*0%*	*0%*	*0%*
0%	*0%*	0%	-	*0%*	*0%*	*0%*
0%	*0%*	0%	-	*0%*	*0%*	*0%*
0%	*0%*	0%	-	*0%*	*0%*	*0%*
0%	*0%*	0%	-	*0%*	*0%*	*0%*
0%	*0%*	0%	-	*0%*	*0%*	*0%*
0%	*0%*	0%	-	*0%*	*0%*	*0%*
0%	*0%*	0%	-	*0%*	*0%*	*0%*
0%	*0%*	0%	-	*0%*	*0%*	*0%*
0%	*0%*	0%	-	*0%*	*0%*	*0%*
0%	*0%*	0%	-	*0%*	*0%*	*0%*
0%	*0%*	0%	-	*0%*	*0%*	*0%*
0%	*0%*	0%	-	*0%*	*0%*	*0%*
0%	*0%*	0%	-	*0%*	*0%*	*0%*
0%	*0%*	0%	-	*0%*	*0%*	*0%*
0%	*0%*	0%	-	*0%*	*0%*	*0%*
0%	*0%*	0%	-	*0%*	*0%*	*0%*
0%	*0%*	0%	-	*0%*	*0%*	*0%*
0%	*0%*	0%	-	*0%*	*0%*	*0%*
0%	*0%*	0%	-	*0%*	*0%*	*0%*
0%	*0%*	0%	-	*0%*	*0%*	*0%*
0%	*0%*	0%	-	*0%*	*0%*	*0%*
0%	*0%*	0%	-	*0%*	*0%*	*0%*
0%	*0%*	0%	-	*0%*	*0%*	*0%*
0%	*0%*	30%	7,00	*80%*	*0%*	*20%*
0%	*0%*	0%	-	*0%*	*0%*	*0%*
0%	*0%*	0%	-	*0%*	*0%*	*0%*
0%	*0%*	0%	-	*0%*	*0%*	*0%*
0%	*0%*	0%	-	*0%*	*0%*	*0%*
0%	*0%*	0%	-	*0%*	*0%*	*0%*
0%	*0%*	0%	-	*0%*	*0%*	*0%*
0%	*0%*	0%	-	*0%*	*0%*	*0%*
0%	*0%*	0%	-	*0%*	*0%*	*0%*
0%	*0%*	0%	-	*0%*	*0%*	*0%*
0%	*0%*	0%	-	*0%*	*0%*	*0%*
0%	*0%*	0%	-	*0%*	*0%*	*0%*
0%	*0%*	0%	-	*0%*	*0%*	*0%*
0%	*0%*	0%	-	*0%*	*0%*	*0%*
0%	*0%*	0%	-	*0%*	*0%*	*0%*
0%	*0%*	0%	-	*0%*	*0%*	*0%*
0%	*0%*	0%	-	*0%*	*0%*	*0%*
0%	*0%*	0%	-	*0%*	*0%*	*0%*
0%	*0%*	0%	-	*0%*	*0%*	*0%*
0%	*0%*	0%	-	*0%*	*0%*	*0%*
0%	*0%*	0%	-	*0%*	*0%*	*0%*
0%	*0%*	0%	-	*0%*	*0%*	*0%*
0%	*0%*	0%	-	*0%*	*0%*	*0%*
0%	*0%*	0%	-	*0%*	*0%*	*0%*
0%	*0%*	0%	-	*0%*	*0%*	*0%*
0%	*0%*	0%	-	*0%*	*0%*	*0%*
0%	*0%*	0%	-	*0%*	*0%*	*0%*
0%	*0%*	0%	-	*0%*	*0%*	*0%*
0%	*0%*	0%	-	*0%*	*0%*	*0%*
0%	*0%*	0%	-	*0%*	*0%*	*0%*
0%	*0%*	0%	-	*0%*	*0%*	*0%*
0%	*0%*	0%	-	*0%*	*0%*	*0%*
0%	*0%*	0%	-	*0%*	*0%*	*0%*
0%	*0%*	0%	-	*0%*	*0%*	*0%*
0%	*0%*	0%	-	*0%*	*0%*	*0%*
0%	*0%*	0%	-	*0%*	*0%*	*0%*
0%	*0%*	0%	-	*0%*	*0%*	*0%*
0%	*0%*	32%	15,04	*100%*	*0%*	*0%*
0%	*0%*	0%	-	*0%*	*0%*	*0%*
0%	*0%*	0%	-	*0%*	*0%*	*0%*
0%	*0%*	0%	-	*0%*	*0%*	*0%*
100%	*0%*	23%	8,00	*25%*	*75%*	*0%*

0%	0%	0%	-	0%	0%	0%
0%	0%	0%	-	0%	0%	0%
0%	0%	0%	-	0%	0%	0%
0%	0%	0%	-	0%	0%	0%
0%	0%	0%	-	0%	0%	0%
0%	0%	0%	-	0%	0%	0%
0%	0%	0%	-	0%	0%	0%
0%	0%	0%	-	0%	0%	0%
0%	0%	0%	-	0%	0%	0%
0%	0%	0%	-	0%	0%	0%
0%	0%	0%	-	0%	0%	0%
0%	0%	0%	-	0%	0%	0%
0%	0%	0%	-	0%	0%	0%
0%	0%	0%	-	0%	0%	0%
0%	0%	0%	-	0%	0%	0%
0%	0%	0%	-	0%	0%	0%
0%	0%	0%	-	0%	0%	0%
0%	0%	0%	-	0%	0%	0%
0%	0%	0%	-	0%	0%	0%
0%	0%	0%	-	0%	0%	0%
0%	0%	0%	-	0%	0%	0%
0%	0%	0%	-	0%	0%	0%
0%	0%	0%	-	0%	0%	0%
0%	0%	0%	-	0%	0%	0%
0%	0%	0%	-	0%	0%	0%
0%	0%	0%	-	0%	0%	0%
0%	0%	0%	-	0%	0%	0%
0%	0%	0%	-	0%	0%	0%
0%	0%	0%	-	0%	0%	0%
0%	0%	0%	-	0%	0%	0%
0%	0%	0%	-	0%	0%	0%
0%	0%	0%	-	0%	0%	0%
0%	0%	0%	-	0%	0%	0%
0%	0%	0%	-	0%	0%	0%
0%	0%	0%	-	0%	0%	0%
0%	0%	0%	-	0%	0%	0%
0%	0%	0%	-	0%	0%	0%
0%	0%	0%	-	0%	0%	0%
0%	0%	0%	-	0%	0%	0%
0%	0%	0%	-	0%	0%	0%
0%	0%	0%	-	0%	0%	0%
0%	0%	0%	-	0%	0%	0%
0%	0%	0%	-	0%	0%	0%
0%	0%	0%	-	0%	0%	0%
0%	0%	0%	-	0%	0%	0%
0%	0%	0%	-	0%	0%	0%
0%	0%	0%	-	0%	0%	0%
0%	0%	0%	-	0%	0%	0%
0%	0%	0%	-	0%	0%	0%
0%	0%	0%	-	0%	0%	0%
0%	0%	0%	-	0%	0%	0%
0%	0%	0%	-	0%	0%	0%
0%	0%	0%	-	0%	0%	0%
0%	0%	0%	-	0%	0%	0%
0%	0%	0%	-	0%	0%	0%
0%	0%	0%	-	0%	0%	0%
0%	0%	0%	-	0%	0%	0%
0%	0%	0%	-	0%	0%	0%
0%	0%	0%	-	0%	0%	0%
0%	0%	0%	-	0%	0%	0%
0%	0%	0%	-	0%	0%	0%
0%	0%	0%	-	0%	0%	0%
0%	0%	0%	-	0%	0%	0%
0%	0%	0%	-	0%	0%	0%
0%	0%	0%	-	0%	0%	0%
0%	0%	0%	-	0%	0%	0%

0%	0%	0%	-	0%	0%	0%
0%	0%	15%	-	100%	0%	0%
0%	0%	0%	-	0%	0%	0%
0%	0%	0%	-	0%	0%	0%
0%	0%	0%	-	0%	0%	0%
0%	0%	0%	-	0%	0%	0%
0%	0%	0%	-	0%	0%	0%
0%	0%	0%	-	0%	0%	0%
0%	0%	0%	-	0%	0%	0%
0%	0%	0%	-	0%	0%	0%
0%	0%	0%	-	0%	0%	0%
0%	0%	10%	-	100%	0%	0%
0%	0%	0%	-	0%	0%	0%
0%	0%	0%	-	0%	0%	0%
0%	0%	20%	-	100%	0%	0%
0%	0%	0%	-	0%	0%	0%
0%	0%	0%	-	0%	0%	0%
0%	0%	0%	-	0%	0%	0%
0%	0%	15%	-	100%	0%	0%
0%	0%	0%	-	0%	0%	0%
0%	0%	0%	-	0%	0%	0%
0%	0%	0%	-	0%	0%	0%
0%	0%	0%	-	0%	0%	0%
0%	0%	0%	-	0%	0%	0%
0%	0%	80%	-	100%	0%	0%
0%	0%	0%	-	0%	0%	0%
0%	0%	0%	-	0%	0%	0%
0%	0%	0%	-	0%	0%	0%
0%	0%	0%	-	0%	0%	0%
0%	0%	0%	-	0%	0%	0%
0%	0%	0%	-	0%	0%	0%
0%	0%	0%	-	0%	0%	0%
0%	0%	0%	-	0%	0%	0%
0%	0%	0%	-	0%	0%	0%
0%	0%	0%	-	0%	0%	0%
100%	0%	0%	-	0%	0%	0%
0%	0%	0%	-	0%	0%	0%
0%	0%	0%	-	0%	0%	0%
0%	0%	0%	-	0%	0%	0%
0%	0%	0%	-	0%	0%	0%
50%	0%	0%	-	0%	0%	0%
0%	0%	0%	-	0%	0%	0%
0%	0%	0%	-	0%	0%	0%
0%	0%	0%	-	0%	0%	0%
0%	0%	0%	-	0%	0%	0%
0%	0%	0%	-	0%	0%	0%
0%	0%	0%	-	0%	0%	0%
0%	0%	0%	-	0%	0%	0%
0%	0%	0%	-	0%	0%	0%
0%	0%	0%	-	0%	0%	0%
0%	0%	0%	-	0%	0%	0%
0%	0%	0%	-	0%	0%	0%
0%	0%	0%	-	0%	0%	0%
0%	0%	0%	-	0%	0%	0%
0%	0%	0%	-	0%	0%	0%
0%	0%	0%	-	0%	0%	0%
0%	0%	0%	-	0%	0%	0%
0%	0%	0%	-	0%	0%	0%
0%	0%	0%	-	0%	0%	0%
0%	0%	0%	-	0%	0%	0%
0%	0%	0%	-	0%	0%	0%
0%	0%	0%	-	0%	0%	0%
0%	0%	0%	-	0%	0%	0%
0%	0%	0%	-	0%	0%	0%
0%	0%	0%	-	0%	0%	0%
0%	0%	0%	-	0%	0%	0%

0%	100%	0%	-	0%	0%	0%
0%	0%	0%	-	0%	0%	0%
0%	0%	0%	-	0%	0%	0%
0%	0%	0%	-	0%	0%	0%
0%	0%	0%	-	0%	0%	0%
0%	0%	0%	-	0%	0%	0%
60%	0%	0%	-	0%	0%	0%
0%	0%	0%	-	0%	0%	0%
0%	0%	0%	-	0%	0%	0%
0%	0%	0%	-	0%	0%	0%
0%	0%	0%	-	0%	0%	0%
0%	0%	0%	-	0%	0%	0%
0%	0%	0%	-	0%	0%	0%
0%	0%	0%	-	0%	0%	0%
0%	0%	0%	-	0%	0%	0%
0%	0%	0%	-	0%	0%	0%
0%	0%	0%	-	0%	0%	0%
0%	0%	0%	-	0%	0%	0%
0%	0%	0%	-	0%	0%	0%
0%	0%	0%	-	0%	0%	0%
0%	0%	0%	-	0%	0%	0%
0%	0%	0%	-	0%	0%	0%
0%	0%	0%	-	0%	0%	0%
0%	0%	0%	-	0%	0%	0%
0%	0%	0%	-	0%	0%	0%
0%	0%	0%	-	0%	0%	0%
0%	0%	0%	-	0%	0%	0%
0%	0%	0%	-	0%	0%	0%
0%	0%	0%	-	0%	0%	0%
0%	0%	0%	-	0%	0%	0%
0%	0%	0%	-	0%	0%	0%
0%	0%	0%	-	0%	0%	0%
0%	0%	0%	-	0%	0%	0%
0%	0%	0%	-	0%	0%	0%
0%	0%	0%	-	0%	0%	0%
0%	0%	0%	-	0%	0%	0%
0%	0%	0%	-	0%	0%	0%
0%	0%	0%	-	0%	0%	0%
0%	0%	0%	-	0%	0%	0%
0%	0%	0%	-	0%	0%	0%
0%	0%	0%	-	0%	0%	0%
0%	0%	0%	-	0%	0%	0%
0%	0%	0%	-	0%	0%	0%
0%	0%	0%	-	0%	0%	0%
0%	0%	0%	-	0%	0%	0%
0%	0%	0%	-	0%	0%	0%
0%	0%	0%	-	0%	0%	0%
0%	0%	0%	-	0%	0%	0%
0%	0%	0%	-	0%	0%	0%
0%	0%	0%	-	0%	0%	0%
0%	0%	0%	-	0%	0%	0%
0%	0%	0%	-	0%	0%	0%
0%	0%	0%	-	0%	0%	0%
0%	0%	0%	-	0%	0%	0%
0%	0%	0%	-	0%	0%	0%
0%	0%	0%	-	0%	0%	0%
0%	0%	0%	-	0%	0%	0%
0%	0%	0%	-	0%	0%	0%
0%	0%	0%	-	0%	0%	0%
0%	0%	0%	-	0%	0%	0%
0%	0%	0%	-	0%	0%	0%
0%	0%	0%	-	0%	0%	0%
0%	0%	0%	-	0%	0%	0%
0%	0%	0%	-	0%	0%	0%
0%	0%	0%	-	0%	0%	0%
0%	0%	0%	-	0%	0%	0%

0%	0%	0%	-	0%	0%	0%
0%	0%	0%	-	0%	0%	0%
0%	0%	0%	-	0%	0%	0%
0%	0%	0%	-	0%	0%	0%
0%	0%	0%	-	0%	0%	0%
0%	0%	0%	-	0%	0%	0%
0%	0%	0%	-	0%	0%	0%
0%	0%	0%	-	0%	0%	0%
0%	0%	0%	-	0%	0%	0%
0%	0%	30%	-	100%	0%	0%
0%	0%	0%	-	0%	0%	0%
0%	0%	0%	-	0%	0%	0%
0%	0%	0%	-	0%	0%	0%
0%	0%	0%	-	0%	0%	0%
0%	0%	0%	-	0%	0%	0%
0%	0%	0%	-	0%	0%	0%
0%	0%	0%	-	0%	0%	0%
0%	0%	0%	-	0%	0%	0%
0%	0%	0%	-	0%	0%	0%
0%	0%	0%	-	0%	0%	0%
0%	0%	0%	-	0%	0%	0%
0%	0%	42%	40,74	100%	0%	0%
0%	0%	0%	-	0%	0%	0%
0%	0%	0%	-	0%	0%	0%
0%	0%	0%	-	0%	0%	0%
0%	0%	0%	-	0%	0%	0%
0%	0%	0%	-	0%	0%	0%
0%	0%	0%	-	0%	0%	0%
0%	0%	0%	-	0%	0%	0%
100%	0%	0%	-	0%	0%	0%
0%	0%	0%	-	0%	0%	0%
0%	0%	0%	-	0%	0%	0%
0%	0%	0%	-	0%	0%	0%
0%	0%	0%	-	0%	0%	0%
0%	0%	0%	-	0%	0%	0%
0%	0%	0%	-	0%	0%	0%
0%	0%	0%	-	0%	0%	0%
0%	0%	0%	-	0%	0%	0%
0%	0%	0%	-	0%	0%	0%
0%	0%	0%	-	0%	0%	0%
100%	0%	0%	-	0%	0%	0%
0%	0%	0%	-	0%	0%	0%
0%	0%	0%	-	0%	0%	0%
0%	0%	0%	-	0%	0%	0%
0%	0%	0%	-	0%	0%	0%
0%	0%	0%	-	0%	0%	0%
0%	0%	0%	-	0%	0%	0%
0%	0%	0%	-	0%	0%	0%
0%	0%	0%	-	0%	0%	0%
45%	40%	0%	-	0%	0%	0%
0%	0%	0%	-	0%	0%	0%
0%	0%	0%	-	0%	0%	0%
0%	0%	0%	-	0%	0%	0%
0%	0%	0%	-	0%	0%	0%
0%	0%	64%	7,00	30%	70%	0%
0%	0%	0%	-	0%	0%	0%
0%	0%	0%	-	0%	0%	0%
0%	0%	0%	-	0%	0%	0%
0%	0%	0%	-	0%	0%	0%
0%	0%	0%	-	0%	0%	0%
0%	0%	0%	-	0%	0%	0%
0%	0%	0%	-	0%	0%	0%
0%	0%	0%	-	0%	0%	0%
0%	0%	0%	-	0%	0%	0%
0%	0%	0%	-	0%	0%	0%
0%	0%	0%	-	0%	0%	0%

0%	*0%*	0%	-	*0%*	*0%*	*0%*
0%	*0%*	0%	-	*0%*	*0%*	*0%*
0%	*0%*	0%	-	*0%*	*0%*	*0%*
0%	*0%*	0%	-	*0%*	*0%*	*0%*
0%	*0%*	0%	-	*0%*	*0%*	*0%*
0%	*0%*	0%	-	*0%*	*0%*	*0%*
0%	*0%*	0%	-	*0%*	*0%*	*0%*
0%	*0%*	0%	-	*0%*	*0%*	*0%*
0%	*0%*	0%	-	*0%*	*0%*	*0%*
0%	*0%*	0%	-	*0%*	*0%*	*0%*
0%	*0%*	0%	-	*0%*	*0%*	*0%*
0%	*0%*	0%	-	*0%*	*0%*	*0%*
0%	*0%*	0%	-	*0%*	*0%*	*0%*
0%	*0%*	0%	-	*0%*	*0%*	*0%*
0%	*0%*	0%	-	*0%*	*0%*	*0%*
0%	*0%*	0%	-	*0%*	*0%*	*0%*
0%	*0%*	0%	-	*0%*	*0%*	*0%*
0%	*0%*	0%	-	*0%*	*0%*	*0%*
0%	*0%*	0%	-	*0%*	*0%*	*0%*
0%	*0%*	0%	-	*0%*	*0%*	*0%*
0%	*0%*	0%	-	*0%*	*0%*	*0%*
0%	*0%*	0%	-	*0%*	*0%*	*0%*
0%	*0%*	0%	-	*0%*	*0%*	*0%*
0%	*0%*	0%	-	*0%*	*0%*	*0%*
0%	*0%*	0%	-	*0%*	*0%*	*0%*
0%	*0%*	0%	-	*0%*	*0%*	*0%*

Dictyosphaeria% of interaction	Total length in interaction with Dictyosphaeria	*% Algae win*	*% Neutral*	*% Coral wins*	Padina % of interaction	Total length in interaction with Padina
0%	-	*0%*	*0%*	*0%*	0%	-
0%	-	*0%*	*0%*	*0%*	0%	-
0%	-	*0%*	*0%*	*0%*	0%	-
0%	-	*0%*	*0%*	*0%*	0%	-
0%	-	*0%*	*0%*	*0%*	0%	-
0%	-	*0%*	*0%*	*0%*	0%	-
0%	-	*0%*	*0%*	*0%*	0%	-
0%	-	*0%*	*0%*	*0%*	0%	-
0%	-	*0%*	*0%*	*0%*	0%	-
0%	-	*0%*	*0%*	*0%*	0%	-
0%	-	*0%*	*0%*	*0%*	0%	-
0%	-	*0%*	*0%*	*0%*	0%	-
0%	-	*0%*	*0%*	*0%*	0%	-
0%	-	*0%*	*0%*	*0%*	0%	-
0%	-	*0%*	*0%*	*0%*	0%	-
0%	-	*0%*	*0%*	*0%*	0%	-
0%	-	*0%*	*0%*	*0%*	0%	-
0%	-	*0%*	*0%*	*0%*	0%	-
0%	-	*0%*	*0%*	*0%*	0%	-
0%	-	*0%*	*0%*	*0%*	20%	3,84
0%	-	*0%*	*0%*	*0%*	0%	-
0%	-	*0%*	*0%*	*0%*	0%	-
0%	-	*0%*	*0%*	*0%*	0%	-
0%	-	*0%*	*0%*	*0%*	0%	-
0%	-	*0%*	*0%*	*0%*	60%	8,19
0%	-	*0%*	*0%*	*0%*	0%	-
0%	-	*0%*	*0%*	*0%*	0%	-
0%	-	*0%*	*0%*	*0%*	10%	1,10
0%	-	*0%*	*0%*	*0%*	0%	-
0%	-	*0%*	*0%*	*0%*	10%	7,70
0%	-	*0%*	*0%*	*0%*	0%	-
0%	-	*0%*	*0%*	*0%*	0%	-
0%	-	*0%*	*0%*	*0%*	0%	-
0%	-	*0%*	*0%*	*0%*	0%	-
0%	-	*0%*	*0%*	*0%*	0%	-
0%	-	*0%*	*0%*	*0%*	0%	-
0%	-	*0%*	*0%*	*0%*	0%	-
0%	-	*0%*	*0%*	*0%*	0%	-
0%	-	*0%*	*0%*	*0%*	15%	15,75
0%	-	*0%*	*0%*	*0%*	10%	3,24
0%	-	*0%*	*0%*	*0%*	0%	-
0%	-	*0%*	*0%*	*0%*	0%	-
0%	-	*0%*	*0%*	*0%*	0%	-
0%	-	*0%*	*0%*	*0%*	5%	1,98
0%	-	*0%*	*0%*	*0%*	0%	-
0%	-	*0%*	*0%*	*0%*	0%	-
0%	-	*0%*	*0%*	*0%*	0%	-
0%	-	*0%*	*0%*	*0%*	0%	-
0%	-	*0%*	*0%*	*0%*	0%	-
0%	-	*0%*	*0%*	*0%*	0%	-
0%	-	*0%*	*0%*	*0%*	0%	-
0%	-	*0%*	*0%*	*0%*	35%	5,99
0%	-	*0%*	*0%*	*0%*	10%	10,40
0%	-	*0%*	*0%*	*0%*	0%	-
0%	-	*0%*	*0%*	*0%*	0%	-
0%	-	*0%*	*0%*	*0%*	0%	-
0%	-	*0%*	*0%*	*0%*	0%	-
0%	-	*0%*	*0%*	*0%*	5%	2,34
0%	-	*0%*	*0%*	*0%*	0%	-
0%	-	*0%*	*0%*	*0%*	0%	-
0%	-	*0%*	*0%*	*0%*	0%	-
0%	-	*0%*	*0%*	*0%*	0%	-

0%	-	*0%*	*0%*	*0%*	0%	-
0%	-	*0%*	*0%*	*0%*	0%	-
0%	-	*0%*	*0%*	*0%*	0%	-
0%	-	*0%*	*0%*	*0%*	0%	-
0%	-	*0%*	*0%*	*0%*	0%	-
0%	-	*0%*	*0%*	*0%*	0%	-
0%	-	*0%*	*0%*	*0%*	0%	-
0%	-	*38%*	*57%*	*5%*	0%	-
0%	-	*30%*	*70%*	*0%*	0%	-
0%	-	*0%*	*0%*	*0%*	0%	-
0%	-	*0%*	*0%*	*0%*	0%	-
0%	-	*0%*	*0%*	*0%*	0%	-
0%	-	*0%*	*0%*	*0%*	0%	-
0%	-	*0%*	*0%*	*0%*	0%	-
0%	-	*30%*	*70%*	*0%*	0%	-
0%	-	*0%*	*0%*	*0%*	23%	9,00
0%	-	*0%*	*0%*	*0%*	0%	-
0%	-	*0%*	*0%*	*0%*	0%	-
0%	-	*0%*	*0%*	*0%*	0%	-
0%	-	*0%*	*0%*	*0%*	0%	-
0%	-	*0%*	*0%*	*0%*	0%	-
0%	-	*0%*	*0%*	*0%*	0%	-
0%	-	*0%*	*0%*	*0%*	0%	-
0%	-	*0%*	*0%*	*0%*	0%	-
0%	-	*0%*	*0%*	*0%*	0%	-
0%	-	*0%*	*0%*	*0%*	0%	-
0%	-	*0%*	*0%*	*0%*	0%	-
0%	-	*0%*	*0%*	*0%*	0%	-
0%	-	*0%*	*0%*	*0%*	0%	-
0%	-	*0%*	*0%*	*0%*	0%	-
0%	-	*0%*	*0%*	*0%*	0%	-
0%	-	*0%*	*0%*	*0%*	0%	-
0%	-	*0%*	*0%*	*0%*	0%	-
0%	-	*0%*	*0%*	*0%*	0%	-
0%	-	*0%*	*0%*	*0%*	0%	-
0%	-	*0%*	*0%*	*0%*	0%	-
0%	-	*0%*	*0%*	*0%*	0%	-
0%	-	*0%*	*0%*	*0%*	0%	-
0%	-	*0%*	*0%*	*0%*	10%	1,71
0%	-	*0%*	*0%*	*0%*	0%	-
0%	-	*0%*	*0%*	*0%*	0%	-
0%	-	*0%*	*0%*	*0%*	0%	-
0%	-	*0%*	*0%*	*0%*	0%	-
0%	-	*0%*	*0%*	*0%*	0%	-
0%	-	*0%*	*0%*	*0%*	0%	-
0%	-	*0%*	*0%*	*0%*	5%	1,60
0%	-	*0%*	*0%*	*0%*	0%	-
0%	-	*0%*	*0%*	*0%*	0%	-
0%	-	*0%*	*0%*	*0%*	0%	-
0%	-	*0%*	*0%*	*0%*	0%	-
0%	-	*0%*	*0%*	*0%*	0%	-
0%	-	*0%*	*0%*	*0%*	0%	-
0%	-	*0%*	*0%*	*0%*	0%	-
0%	-	*0%*	*0%*	*0%*	0%	-
0%	-	*0%*	*0%*	*0%*	0%	-
0%	-	*0%*	*0%*	*0%*	0%	-
0%	-	*0%*	*0%*	*0%*	0%	-
0%	-	*0%*	*0%*	*0%*	0%	-
0%	-	*0%*	*0%*	*0%*	0%	-
0%	-	*0%*	*0%*	*0%*	0%	-
0%	-	*0%*	*0%*	*0%*	0%	-
0%	-	*0%*	*0%*	*0%*	0%	-
0%	-	*0%*	*0%*	*0%*	0%	-
0%	-	*0%*	*0%*	*0%*	0%	-
0%	-	*0%*	*0%*	*0%*	0%	-
0%	-	*0%*	*0%*	*0%*	0%	-

0%	-	*0%*	*0%*	*0%*	0%	-
0%	-	*0%*	*0%*	*0%*	0%	-
0%	-	*0%*	*0%*	*0%*	0%	-
0%	-	*0%*	*0%*	*0%*	0%	-
0%	-	*0%*	*0%*	*0%*	0%	-
0%	-	*0%*	*0%*	*0%*	0%	-
0%	-	*0%*	*0%*	*0%*	0%	-
0%	-	*0%*	*0%*	*0%*	0%	-
0%	-	*0%*	*0%*	*0%*	0%	-
0%	-	*0%*	*0%*	*0%*	0%	-
0%	-	*0%*	*0%*	*0%*	0%	-
0%	-	*0%*	*0%*	*0%*	0%	-
0%	-	*0%*	*0%*	*0%*	0%	-
0%	-	*0%*	*0%*	*0%*	0%	-
0%	-	*0%*	*0%*	*0%*	0%	-
0%	-	*0%*	*0%*	*0%*	0%	-
0%	-	*0%*	*0%*	*0%*	0%	-
0%	-	*0%*	*0%*	*0%*	0%	-
0%	-	*0%*	*0%*	*0%*	0%	-
0%	-	*0%*	*0%*	*0%*	0%	-
0%	-	*0%*	*0%*	*0%*	0%	-
0%	-	*0%*	*0%*	*0%*	0%	-
0%	-	*0%*	*0%*	*0%*	0%	-
0%	-	*0%*	*0%*	*0%*	0%	-
0%	-	*0%*	*0%*	*0%*	0%	-
0%	-	*0%*	*0%*	*0%*	0%	-
0%	-	*0%*	*0%*	*0%*	0%	-
0%	-	*0%*	*0%*	*0%*	0%	-
0%	-	*0%*	*0%*	*0%*	0%	-
0%	-	*0%*	*0%*	*0%*	0%	-
0%	-	*0%*	*0%*	*0%*	0%	-
0%	-	*0%*	*0%*	*0%*	0%	-
0%	-	*0%*	*0%*	*0%*	0%	-
0%	-	*0%*	*0%*	*0%*	0%	-
0%	-	*0%*	*0%*	*0%*	0%	-
0%	-	*0%*	*0%*	*0%*	0%	-
0%	-	*0%*	*0%*	*0%*	0%	-
0%	-	*0%*	*0%*	*0%*	0%	-
0%	-	*0%*	*0%*	*0%*	0%	-
0%	-	*0%*	*0%*	*0%*	0%	-
0%	-	*0%*	*0%*	*0%*	0%	-
0%	-	*0%*	*0%*	*0%*	0%	-
0%	-	*0%*	*0%*	*0%*	0%	-
0%	-	*0%*	*0%*	*0%*	0%	-
0%	-	*0%*	*0%*	*0%*	0%	-
0%	-	*0%*	*0%*	*0%*	0%	-
0%	-	*0%*	*0%*	*0%*	0%	-
0%	-	*0%*	*0%*	*0%*	15%	5,85
0%	-	*0%*	*0%*	*0%*	0%	-
0%	-	*0%*	*0%*	*0%*	0%	-
0%	-	*0%*	*0%*	*0%*	0%	-
0%	-	*0%*	*0%*	*0%*	0%	-
0%	-	*0%*	*0%*	*0%*	0%	-
0%	-	*0%*	*0%*	*0%*	0%	-
0%	-	*0%*	*0%*	*0%*	0%	-
0%	-	*0%*	*0%*	*0%*	0%	-
0%	-	*0%*	*0%*	*0%*	0%	-
0%	-	*0%*	*0%*	*0%*	0%	-
0%	-	*0%*	*0%*	*0%*	0%	-
0%	-	*0%*	*0%*	*0%*	0%	-
0%	-	*0%*	*0%*	*0%*	0%	-
0%	-	*0%*	*0%*	*0%*	0%	-
0%	-	*0%*	*0%*	*0%*	0%	-
0%	-	*0%*	*0%*	*0%*	0%	-
0%	-	*0%*	*0%*	*0%*	0%	-
0%	-	*0%*	*0%*	*0%*	0%	-

0%	-	0%	0%	0%	0%	-
0%	-	0%	0%	0%	0%	-
0%	-	0%	0%	0%	0%	-
0%	-	0%	0%	0%	0%	-
0%	-	0%	0%	0%	0%	-
0%	-	0%	0%	0%	0%	-
0%	-	0%	0%	0%	0%	-
0%	-	0%	0%	0%	0%	-
0%	-	0%	0%	0%	0%	-
0%	-	0%	0%	0%	0%	-
0%	-	0%	0%	0%	10%	10,12
0%	-	0%	0%	0%	0%	-
0%	-	0%	0%	0%	0%	-
0%	-	0%	0%	0%	0%	-
0%	-	0%	0%	0%	0%	-
0%	-	0%	0%	0%	0%	-
0%	-	0%	0%	0%	0%	-
0%	-	0%	0%	0%	0%	-
0%	-	0%	0%	0%	0%	-
0%	-	0%	0%	0%	0%	-
0%	-	0%	0%	0%	0%	-
0%	-	0%	0%	0%	0%	-
0%	-	0%	0%	0%	0%	-
0%	-	0%	0%	0%	10%	2,52
0%	-	0%	0%	0%	0%	-
0%	-	0%	0%	0%	0%	-
0%	-	0%	0%	0%	0%	-
0%	-	0%	0%	0%	0%	-
0%	-	0%	0%	0%	0%	-
0%	-	0%	0%	0%	0%	-
0%	-	0%	0%	0%	0%	-
0%	-	0%	0%	0%	0%	-
0%	-	0%	0%	0%	0%	-
0%	-	0%	0%	0%	0%	-
0%	-	0%	0%	0%	10%	6,00
0%	-	0%	0%	0%	0%	-
0%	-	0%	0%	0%	0%	-
0%	-	0%	0%	0%	0%	-
0%	-	0%	0%	0%	0%	-
0%	-	0%	0%	0%	0%	-
0%	-	0%	0%	0%	0%	-
0%	-	0%	0%	0%	0%	-
0%	-	0%	0%	0%	0%	-
0%	-	0%	0%	0%	0%	-
0%	-	0%	0%	0%	0%	-
0%	-	0%	0%	0%	0%	-
0%	-	0%	0%	0%	5%	5,30
0%	-	0%	0%	0%	0%	-
0%	-	0%	0%	0%	15%	1,73
0%	-	0%	0%	0%	0%	-
0%	-	0%	0%	0%	5%	0,65
0%	-	0%	0%	0%	0%	-
0%	-	0%	0%	0%	0%	-
0%	-	0%	0%	0%	0%	-
0%	-	0%	0%	0%	0%	-
0%	-	0%	0%	0%	0%	-
0%	-	0%	0%	0%	0%	-
0%	-	0%	0%	0%	0%	-
0%	-	0%	0%	0%	0%	-
0%	-	0%	0%	0%	0%	-
0%	-	0%	0%	0%	0%	-
0%	-	0%	0%	0%	0%	-
0%	-	0%	0%	0%	0%	-
0%	-	0%	0%	0%	0%	-
0%	-	0%	0%	0%	0%	-
0%	-	0%	0%	0%	0%	-

0%	-	*0%*	*0%*	*0%*	0%	-
0%	-	*0%*	*0%*	*0%*	0%	-
0%	-	*0%*	*0%*	*0%*	0%	-
0%	-	*0%*	*0%*	*0%*	0%	-
0%	-	*0%*	*0%*	*0%*	0%	-
0%	-	*0%*	*0%*	*0%*	0%	-
0%	-	*0%*	*0%*	*0%*	0%	-
0%	-	*0%*	*0%*	*0%*	0%	-
0%	-	*0%*	*0%*	*0%*	0%	-
0%	-	*0%*	*0%*	*0%*	0%	-
0%	-	*0%*	*0%*	*0%*	0%	-
0%	-	*0%*	*0%*	*0%*	0%	-
0%	-	*0%*	*0%*	*0%*	0%	-
0%	-	*0%*	*0%*	*0%*	0%	-
0%	-	*0%*	*0%*	*0%*	0%	-
0%	-	*0%*	*0%*	*0%*	0%	-
0%	-	*0%*	*0%*	*0%*	0%	-
0%	-	*0%*	*0%*	*0%*	0%	-
0%	-	*0%*	*0%*	*0%*	0%	-
0%	-	*0%*	*0%*	*0%*	0%	-
0%	-	*0%*	*0%*	*0%*	0%	-
0%	-	*0%*	*0%*	*0%*	0%	-
0%	-	*0%*	*0%*	*0%*	0%	-
0%	-	*0%*	*0%*	*0%*	0%	-
0%	-	*0%*	*0%*	*0%*	0%	-
0%	-	*0%*	*0%*	*0%*	0%	-
0%	-	*0%*	*0%*	*0%*	0%	-
0%	-	*0%*	*0%*	*0%*	60%	12,09
0%	-	*0%*	*0%*	*0%*	0%	-
0%	-	*0%*	*0%*	*0%*	0%	-
0%	-	*0%*	*0%*	*0%*	0%	-
0%	-	*0%*	*0%*	*0%*	0%	-
0%	-	*0%*	*0%*	*0%*	0%	-
0%	-	*0%*	*0%*	*0%*	20%	2,76
0%	-	*0%*	*0%*	*0%*	0%	-
0%	-	*0%*	*0%*	*0%*	0%	-
0%	-	*0%*	*0%*	*0%*	0%	-
0%	-	*0%*	*0%*	*0%*	0%	-
0%	-	*0%*	*0%*	*0%*	0%	-
0%	-	*0%*	*0%*	*0%*	0%	-
0%	-	*0%*	*0%*	*0%*	0%	-
0%	-	*0%*	*0%*	*0%*	0%	-
0%	-	*0%*	*0%*	*0%*	0%	-
0%	-	*0%*	*0%*	*0%*	0%	-
0%	-	*0%*	*0%*	*0%*	0%	-
0%	-	*0%*	*0%*	*0%*	0%	-
0%	-	*0%*	*0%*	*0%*	0%	-
0%	-	*0%*	*0%*	*0%*	0%	-
0%	-	*0%*	*0%*	*0%*	0%	-
0%	-	*0%*	*0%*	*0%*	60%	23,52
0%	-	*0%*	*0%*	*0%*	0%	-
0%	-	*0%*	*0%*	*0%*	50%	10,50
0%	-	*0%*	*0%*	*0%*	0%	-
0%	-	*0%*	*0%*	*0%*	0%	-
0%	-	*0%*	*0%*	*0%*	0%	-
0%	-	*0%*	*0%*	*0%*	0%	-
0%	-	*0%*	*0%*	*0%*	5%	0,93
0%	-	*0%*	*0%*	*0%*	0%	-
0%	-	*0%*	*0%*	*0%*	0%	-
0%	-	*0%*	*0%*	*0%*	0%	-
0%	-	*0%*	*0%*	*0%*	19%	2,09
0%	-	*0%*	*0%*	*0%*	0%	-
0%	-	*0%*	*0%*	*0%*	0%	-
0%	-	*0%*	*0%*	*0%*	20%	2,40
0%	-	*0%*	*0%*	*0%*	0%	-
0%	-	*0%*	*0%*	*0%*	0%	-

0%	-	*0%*	*0%*	*0%*	30%	24,24
0%	-	*0%*	*0%*	*0%*	0%	-
0%	-	*0%*	*0%*	*0%*	0%	-
0%	-	*0%*	*0%*	*0%*	40%	5,28
0%	-	*0%*	*0%*	*0%*	0%	-
0%	-	*0%*	*0%*	*0%*	0%	-
0%	-	*0%*	*0%*	*0%*	0%	-
0%	-	*0%*	*0%*	*0%*	0%	-
0%	-	*0%*	*0%*	*0%*	0%	-
0%	-	*0%*	*0%*	*0%*	0%	-
0%	-	*0%*	*0%*	*0%*	0%	-
0%	-	*0%*	*0%*	*0%*	25%	7,00
0%	-	*0%*	*0%*	*0%*	0%	-
0%	-	*0%*	*0%*	*0%*	0%	-
0%	-	*0%*	*0%*	*0%*	0%	-
0%	-	*0%*	*0%*	*0%*	0%	-
0%	-	*0%*	*0%*	*0%*	0%	-
0%	-	*0%*	*0%*	*0%*	0%	-
0%	-	*0%*	*0%*	*0%*	0%	-
0%	-	*0%*	*0%*	*0%*	0%	-
0%	-	*0%*	*0%*	*0%*	20%	4,08
0%	-	*0%*	*0%*	*0%*	0%	-
0%	-	*0%*	*0%*	*0%*	0%	-
0%	-	*0%*	*0%*	*0%*	0%	-
0%	-	*0%*	*0%*	*0%*	0%	-
0%	-	*0%*	*0%*	*0%*	0%	-
0%	-	*0%*	*0%*	*0%*	0%	-
0%	-	*0%*	*0%*	*0%*	0%	-
0%	-	*0%*	*0%*	*0%*	0%	-
0%	-	*0%*	*0%*	*0%*	0%	-
0%	-	*0%*	*0%*	*0%*	0%	-
0%	-	*0%*	*0%*	*0%*	0%	-
0%	-	*0%*	*0%*	*0%*	0%	-
0%	-	*0%*	*0%*	*0%*	0%	-
0%	-	*0%*	*0%*	*0%*	0%	-
0%	-	*0%*	*0%*	*0%*	0%	-
0%	-	*0%*	*0%*	*0%*	0%	-
0%	-	*0%*	*0%*	*0%*	0%	-
0%	-	*0%*	*0%*	*0%*	0%	-
0%	-	*0%*	*0%*	*0%*	0%	-
0%	-	*0%*	*0%*	*0%*	0%	-
0%	-	*0%*	*0%*	*0%*	0%	-
0%	-	*0%*	*0%*	*0%*	0%	-
0%	-	*0%*	*0%*	*0%*	0%	-
0%	-	*0%*	*0%*	*0%*	0%	-
0%	-	*0%*	*0%*	*0%*	0%	-
0%	-	*0%*	*0%*	*0%*	0%	-
0%	-	*0%*	*0%*	*0%*	0%	-
0%	-	*0%*	*0%*	*0%*	0%	-
0%	-	*0%*	*0%*	*0%*	0%	-
0%	-	*0%*	*0%*	*0%*	15%	4,08
0%	-	*0%*	*0%*	*0%*	5%	3,35
0%	-	*0%*	*0%*	*0%*	0%	-
0%	-	*0%*	*0%*	*0%*	0%	-
0%	-	*0%*	*0%*	*0%*	0%	-
0%	-	*0%*	*0%*	*0%*	0%	-
0%	-	*0%*	*0%*	*0%*	0%	-
0%	-	*0%*	*0%*	*0%*	0%	-
0%	-	*0%*	*0%*	*0%*	0%	-
0%	-	*0%*	*0%*	*0%*	0%	-
0%	-	*0%*	*0%*	*0%*	10%	3,30
0%	-	*0%*	*0%*	*0%*	0%	-
0%	-	*0%*	*0%*	*0%*	0%	-
0%	-	*0%*	*0%*	*0%*	0%	-
0%	-	*0%*	*0%*	*0%*	0%	-
0%	-	*0%*	*0%*	*0%*	15%	2,40

0%	-	0%	0%	0%	0%	-
0%	-	0%	0%	0%	0%	-
0%	-	0%	0%	0%	0%	-
0%	-	0%	0%	0%	0%	-
0%	-	0%	0%	0%	0%	-
0%	-	0%	0%	0%	0%	-
0%	-	0%	0%	0%	0%	-
0%	-	0%	0%	0%	0%	-
0%	-	0%	0%	0%	0%	-
0%	-	0%	0%	0%	0%	-
0%	-	0%	0%	0%	0%	-
0%	-	0%	0%	0%	0%	-
0%	-	0%	0%	0%	0%	-
0%	-	0%	0%	0%	0%	-
0%	-	0%	0%	0%	0%	-
0%	-	0%	0%	0%	0%	-
0%	-	0%	0%	0%	0%	-
0%	-	0%	0%	0%	0%	-
0%	-	0%	0%	0%	0%	-
0%	-	0%	0%	0%	0%	-
0%	-	0%	0%	0%	0%	-
0%	-	0%	0%	0%	34%	5,24
0%	-	0%	0%	0%	0%	-
0%	-	0%	0%	0%	0%	-
0%	-	0%	0%	0%	0%	-
0%	-	0%	0%	0%	0%	-
0%	-	0%	0%	0%	20%	3,52
0%	-	0%	0%	0%	0%	-
0%	-	0%	0%	0%	0%	-
0%	-	0%	0%	0%	0%	-
0%	-	0%	0%	0%	0%	-
0%	-	0%	0%	0%	0%	-
0%	-	0%	0%	0%	0%	-
0%	-	0%	0%	0%	0%	-
0%	-	0%	0%	0%	0%	-
0%	-	0%	0%	0%	0%	-
0%	-	0%	0%	0%	0%	-
0%	-	0%	0%	0%	0%	-
0%	-	0%	0%	0%	0%	-
0%	-	0%	0%	0%	0%	-
0%	-	0%	0%	0%	0%	-
0%	-	0%	0%	0%	5%	0,80
0%	-	0%	0%	0%	0%	-
0%	-	0%	0%	0%	0%	-
0%	-	0%	0%	0%	0%	-
0%	-	0%	0%	0%	0%	-
0%	-	0%	0%	0%	0%	-
0%	-	0%	0%	0%	0%	-
0%	-	0%	0%	0%	0%	-
0%	-	0%	0%	0%	0%	-
0%	-	0%	0%	0%	0%	-
0%	-	0%	0%	0%	0%	-
0%	-	0%	0%	0%	0%	-
0%	-	0%	0%	0%	0%	-
0%	-	0%	0%	0%	0%	-
0%	-	0%	0%	0%	0%	-
0%	-	0%	0%	0%	0%	-
0%	-	0%	0%	0%	5%	1,17
0%	-	0%	0%	0%	0%	-
0%	-	0%	0%	0%	0%	-
0%	-	0%	0%	0%	0%	-
0%	-	0%	0%	0%	0%	-
0%	-	0%	0%	0%	0%	-
0%	-	0%	0%	0%	0%	-
0%	-	0%	0%	0%	0%	-
0%	-	0%	0%	0%	0%	-

0%	-	0%	0%	0%	0%	-
0%	-	0%	0%	0%	0%	-
0%	-	0%	0%	0%	0%	-
0%	-	0%	0%	0%	0%	-
0%	-	0%	0%	0%	0%	-
0%	-	0%	0%	0%	0%	-
0%	-	0%	0%	0%	0%	-
0%	-	0%	0%	0%	0%	-
0%	-	0%	0%	0%	0%	-
0%	-	0%	0%	0%	15%	2,83
0%	-	0%	0%	0%	0%	-
0%	-	0%	0%	0%	0%	-
0%	-	0%	0%	0%	0%	-
0%	-	0%	0%	0%	0%	-
0%	-	0%	0%	0%	0%	-
0%	-	0%	0%	0%	0%	-
0%	-	0%	0%	0%	0%	-
0%	-	0%	0%	0%	0%	-
0%	-	0%	0%	0%	0%	-
0%	-	0%	0%	0%	0%	-
0%	-	0%	0%	0%	0%	-
0%	-	0%	0%	0%	0%	-
0%	-	0%	0%	0%	0%	-
0%	-	0%	0%	0%	0%	-
0%	-	0%	0%	0%	0%	-
0%	-	0%	0%	0%	0%	-
0%	-	0%	0%	0%	0%	-
0%	-	0%	0%	0%	0%	-
0%	-	0%	0%	0%	0%	-
0%	-	0%	0%	0%	0%	-
0%	-	0%	0%	0%	0%	-
0%	-	0%	0%	0%	0%	-
0%	-	0%	0%	0%	0%	-
0%	-	0%	0%	0%	0%	-
0%	-	0%	0%	0%	0%	-
0%	-	0%	0%	0%	0%	-
0%	-	0%	0%	0%	0%	-
0%	-	0%	0%	0%	0%	-
0%	-	0%	0%	0%	0%	-
0%	-	0%	0%	0%	0%	-
0%	-	0%	0%	0%	0%	-
0%	-	0%	0%	0%	0%	-
0%	-	0%	0%	0%	0%	-
0%	-	0%	0%	0%	0%	-
0%	-	0%	0%	0%	0%	-
0%	-	0%	0%	0%	0%	-
0%	-	0%	0%	0%	0%	-
0%	-	0%	0%	0%	0%	-
0%	-	0%	0%	0%	0%	-
0%	-	0%	0%	0%	100%	-
0%	-	0%	0%	0%	0%	-
0%	-	0%	0%	0%	0%	-
0%	-	0%	0%	0%	0%	-
0%	-	0%	0%	0%	0%	-
0%	-	0%	0%	0%	0%	-
0%	-	0%	0%	0%	0%	-
0%	-	0%	0%	0%	0%	-
0%	-	0%	0%	0%	0%	-
0%	-	0%	0%	0%	0%	-
0%	-	0%	0%	0%	0%	-
0%	-	0%	0%	0%	0%	-
0%	-	0%	0%	0%	0%	-
0%	-	0%	0%	0%	0%	-
0%	-	0%	0%	0%	0%	-
0%	-	0%	0%	0%	0%	-
0%	-	0%	0%	0%	0%	-

0%	-	*0%*	*0%*	*0%*	0%	-
0%	-	*0%*	*0%*	*0%*	0%	-
0%	-	*0%*	*0%*	*0%*	0%	-
0%	-	*0%*	*0%*	*0%*	0%	-
0%	-	*0%*	*0%*	*0%*	0%	-
0%	-	*0%*	*0%*	*0%*	0%	-
0%	-	*0%*	*0%*	*0%*	0%	-
0%	-	*0%*	*0%*	*0%*	0%	-
0%	-	*0%*	*0%*	*0%*	0%	-
0%	-	*0%*	*0%*	*0%*	0%	-
0%	-	*0%*	*0%*	*0%*	0%	-
0%	-	*0%*	*0%*	*0%*	0%	-
0%	-	*0%*	*0%*	*0%*	0%	-
0%	-	*0%*	*0%*	*0%*	0%	-
0%	-	*0%*	*0%*	*0%*	0%	-
0%	-	*0%*	*0%*	*0%*	16%	0,79
0%	-	*0%*	*0%*	*0%*	0%	-
0%	-	*0%*	*0%*	*0%*	0%	-
0%	-	*0%*	*0%*	*0%*	0%	-
0%	-	*0%*	*0%*	*0%*	0%	-
0%	-	*0%*	*0%*	*0%*	0%	-
0%	-	*0%*	*0%*	*0%*	7%	2,03
0%	-	*0%*	*0%*	*0%*	0%	-
0%	-	*0%*	*0%*	*0%*	0%	-
0%	-	*0%*	*0%*	*0%*	0%	-
0%	-	*0%*	*0%*	*0%*	0%	-
0%	-	*0%*	*0%*	*0%*	0%	-
0%	-	*0%*	*0%*	*0%*	0%	-
0%	-	*0%*	*0%*	*0%*	0%	-
0%	-	*0%*	*0%*	*0%*	0%	-
0%	-	*0%*	*0%*	*0%*	0%	-
0%	-	*0%*	*0%*	*0%*	0%	-
0%	-	*0%*	*0%*	*0%*	0%	-
0%	-	*0%*	*0%*	*0%*	0%	-
0%	-	*0%*	*0%*	*0%*	0%	-
0%	-	*0%*	*0%*	*0%*	0%	-
0%	-	*0%*	*0%*	*0%*	0%	-
0%	-	*0%*	*0%*	*0%*	0%	-
0%	-	*0%*	*0%*	*0%*	0%	-
0%	-	*0%*	*0%*	*0%*	0%	-
0%	-	*0%*	*0%*	*0%*	0%	-
0%	-	*0%*	*0%*	*0%*	0%	-
0%	-	*0%*	*0%*	*0%*	0%	-
0%	-	*0%*	*0%*	*0%*	0%	-
0%	-	*0%*	*0%*	*0%*	0%	-
0%	-	*0%*	*0%*	*0%*	0%	-
0%	-	*0%*	*0%*	*0%*	0%	-
0%	-	*0%*	*0%*	*0%*	0%	-
0%	-	*0%*	*0%*	*0%*	0%	-
0%	-	*0%*	*0%*	*0%*	0%	-
0%	-	*0%*	*0%*	*0%*	0%	-
0%	-	*0%*	*0%*	*0%*	0%	-
0%	-	*0%*	*0%*	*0%*	0%	-
0%	-	*0%*	*0%*	*0%*	0%	-
0%	-	*0%*	*0%*	*0%*	0%	-
0%	-	*0%*	*0%*	*0%*	0%	-
0%	-	*0%*	*0%*	*0%*	0%	-
0%	-	*0%*	*0%*	*0%*	0%	-
0%	-	*0%*	*0%*	*0%*	0%	-
0%	-	*0%*	*0%*	*0%*	0%	-
0%	-	*0%*	*0%*	*0%*	0%	-
0%	-	*0%*	*0%*	*0%*	0%	-
0%	-	*0%*	*0%*	*0%*	0%	-
0%	-	*0%*	*0%*	*0%*	0%	-
0%	-	*0%*	*0%*	*0%*	0%	-
0%	-	*0%*	*0%*	*0%*	0%	-

0%	-	*0%*	*0%*	*0%*	0%	-
0%	-	*0%*	*0%*	*0%*	0%	-
0%	-	*0%*	*0%*	*0%*	0%	-
0%	-	*0%*	*0%*	*0%*	0%	-
0%	-	*0%*	*0%*	*0%*	0%	-
0%	-	*0%*	*0%*	*0%*	0%	-
0%	-	*0%*	*0%*	*0%*	0%	-
0%	-	*0%*	*0%*	*0%*	0%	-
0%	-	*0%*	*0%*	*0%*	0%	-
0%	-	*0%*	*0%*	*0%*	0%	-
0%	-	*0%*	*0%*	*0%*	0%	-
0%	-	*0%*	*0%*	*0%*	0%	-
0%	-	*0%*	*0%*	*0%*	0%	-
0%	-	*0%*	*0%*	*0%*	0%	-
0%	-	*0%*	*0%*	*0%*	0%	-
0%	-	*0%*	*0%*	*0%*	0%	-
0%	-	*0%*	*0%*	*0%*	0%	-
0%	-	*0%*	*0%*	*0%*	0%	-
0%	-	*0%*	*0%*	*0%*	0%	-
0%	-	*0%*	*0%*	*0%*	0%	-
0%	-	*0%*	*0%*	*0%*	0%	-
0%	-	*0%*	*0%*	*0%*	0%	-
0%	-	*0%*	*0%*	*0%*	0%	-
0%	-	*0%*	*0%*	*0%*	0%	-
0%	-	*0%*	*0%*	*0%*	0%	-
0%	-	*0%*	*0%*	*0%*	0%	-
0%	-	*0%*	*0%*	*0%*	0%	-
0%	-	*0%*	*0%*	*0%*	0%	-
0%	-	*0%*	*0%*	*0%*	0%	-
0%	-	*0%*	*0%*	*0%*	0%	-
0%	-	*0%*	*0%*	*0%*	0%	-
0%	-	*0%*	*0%*	*0%*	0%	-
0%	-	*0%*	*0%*	*0%*	0%	-
0%	-	*0%*	*0%*	*0%*	0%	-
0%	-	*0%*	*0%*	*0%*	0%	-
0%	-	*0%*	*0%*	*0%*	0%	-
0%	-	*0%*	*0%*	*0%*	0%	-
0%	-	*0%*	*0%*	*0%*	0%	-
0%	-	*0%*	*0%*	*0%*	0%	-
0%	-	*0%*	*0%*	*0%*	0%	-
0%	-	*0%*	*0%*	*0%*	0%	-
0%	-	*0%*	*0%*	*0%*	0%	-
0%	-	*0%*	*0%*	*0%*	0%	-
0%	-	*0%*	*0%*	*0%*	0%	-
0%	-	*0%*	*0%*	*0%*	0%	-
0%	-	*0%*	*0%*	*0%*	0%	-
0%	-	*0%*	*0%*	*0%*	0%	-
0%	-	*0%*	*0%*	*0%*	0%	-
0%	-	*0%*	*0%*	*0%*	0%	-
0%	-	*0%*	*0%*	*0%*	0%	-
0%	-	*0%*	*0%*	*0%*	0%	-
0%	-	*0%*	*0%*	*0%*	0%	-
0%	-	*0%*	*0%*	*0%*	0%	-
0%	-	*0%*	*0%*	*0%*	0%	-
0%	-	*0%*	*0%*	*0%*	0%	-
0%	-	*0%*	*0%*	*0%*	0%	-
0%	-	*0%*	*0%*	*0%*	0%	-
0%	-	*0%*	*0%*	*0%*	0%	-
0%	-	*0%*	*0%*	*0%*	0%	-
0%	-	*0%*	*0%*	*0%*	0%	-
0%	-	*0%*	*0%*	*0%*	0%	-
0%	-	*0%*	*0%*	*0%*	0%	-
0%	-	*0%*	*0%*	*0%*	0%	-
0%	-	*0%*	*0%*	*0%*	0%	-
0%	-	*0%*	*0%*	*0%*	0%	-
0%	-	*0%*	*0%*	*0%*	0%	-

0%	-	*0%*	*0%*	*0%*	0%	-
0%	-	*0%*	*0%*	*0%*	0%	-
0%	-	*0%*	*0%*	*0%*	0%	-
0%	-	*0%*	*0%*	*0%*	0%	-
0%	-	*0%*	*0%*	*0%*	0%	-
0%	-	*0%*	*0%*	*0%*	0%	-
0%	-	*0%*	*0%*	*0%*	0%	-
0%	-	*0%*	*0%*	*0%*	0%	-
0%	-	*0%*	*0%*	*0%*	0%	-
0%	-	*0%*	*0%*	*0%*	0%	-
0%	-	*0%*	*0%*	*0%*	0%	-
0%	-	*0%*	*0%*	*0%*	0%	-
0%	-	*0%*	*0%*	*0%*	0%	-
0%	-	*0%*	*0%*	*0%*	0%	-
0%	-	*0%*	*0%*	*0%*	0%	-
0%	-	*0%*	*0%*	*0%*	0%	-
0%	-	*0%*	*0%*	*0%*	0%	-
0%	-	*0%*	*0%*	*0%*	0%	-
0%	-	*0%*	*0%*	*0%*	0%	-
0%	-	*0%*	*0%*	*0%*	0%	-
0%	-	*0%*	*0%*	*0%*	0%	-
0%	-	*0%*	*0%*	*0%*	0%	-
0%	-	*0%*	*0%*	*0%*	0%	-
0%	-	*0%*	*0%*	*0%*	0%	-
0%	-	*0%*	*0%*	*0%*	0%	-
0%	-	*0%*	*0%*	*0%*	0%	-
0%	-	*0%*	*0%*	*0%*	0%	-
0%	-	*0%*	*0%*	*0%*	0%	-
0%	-	*0%*	*0%*	*0%*	0%	-
0%	-	*0%*	*0%*	*0%*	0%	-
0%	-	*0%*	*0%*	*0%*	0%	-
0%	-	*0%*	*0%*	*0%*	0%	-
0%	-	*0%*	*0%*	*0%*	0%	-
0%	-	*0%*	*0%*	*0%*	0%	-
0%	-	*0%*	*0%*	*0%*	0%	-
0%	-	*0%*	*0%*	*0%*	0%	-
0%	-	*0%*	*0%*	*0%*	0%	-
0%	-	*0%*	*0%*	*0%*	0%	-
0%	-	*0%*	*0%*	*0%*	0%	-
0%	-	*0%*	*0%*	*0%*	0%	-
0%	-	*0%*	*0%*	*0%*	0%	-
0%	-	*0%*	*0%*	*0%*	0%	-
0%	-	*0%*	*0%*	*0%*	0%	-
0%	-	*0%*	*0%*	*0%*	0%	-
0%	-	*0%*	*0%*	*0%*	0%	-
0%	-	*0%*	*0%*	*0%*	0%	-
0%	-	*0%*	*0%*	*0%*	0%	-
0%	-	*0%*	*0%*	*0%*	0%	-
0%	-	*0%*	*0%*	*0%*	0%	-
0%	-	*0%*	*0%*	*0%*	0%	-
0%	-	*0%*	*0%*	*0%*	0%	-
0%	-	*0%*	*0%*	*0%*	0%	-
0%	-	*0%*	*0%*	*0%*	0%	-
0%	-	*0%*	*0%*	*0%*	0%	-
0%	-	*0%*	*0%*	*0%*	0%	-
0%	-	*0%*	*0%*	*0%*	0%	-
0%	-	*0%*	*0%*	*0%*	0%	-
0%	-	*0%*	*0%*	*0%*	0%	-
0%	-	*0%*	*0%*	*0%*	0%	-
0%	-	*0%*	*0%*	*0%*	0%	-
0%	-	*0%*	*0%*	*0%*	0%	-
0%	-	*0%*	*0%*	*0%*	0%	-
0%	-	*0%*	*0%*	*0%*	0%	-
0%	-	*0%*	*0%*	*0%*	0%	-
0%	-	*0%*	*0%*	*0%*	0%	-
0%	-	*0%*	*0%*	*0%*	0%	-

0%	-	*0%*	*0%*	*0%*	0%	-
0%	-	*0%*	*0%*	*0%*	0%	-
0%	-	*0%*	*0%*	*0%*	0%	-
0%	-	*0%*	*0%*	*0%*	0%	-
0%	-	*0%*	*0%*	*0%*	0%	-
0%	-	*0%*	*0%*	*0%*	0%	-
0%	-	*0%*	*0%*	*0%*	0%	-
0%	-	*0%*	*0%*	*0%*	0%	-
0%	-	*0%*	*0%*	*0%*	0%	-
0%	-	*0%*	*0%*	*0%*	0%	-
0%	-	*0%*	*0%*	*0%*	0%	-
0%	-	*0%*	*0%*	*0%*	0%	-
0%	-	*0%*	*0%*	*0%*	0%	-
0%	-	*0%*	*0%*	*0%*	0%	-
0%	-	*0%*	*0%*	*0%*	0%	-
0%	-	*0%*	*0%*	*0%*	0%	-
0%	-	*0%*	*0%*	*0%*	0%	-
0%	-	*0%*	*0%*	*0%*	15%	5,85
0%	-	*0%*	*0%*	*0%*	0%	-
0%	-	*0%*	*0%*	*0%*	9%	2,88
0%	-	*0%*	*0%*	*0%*	0%	-
0%	-	*0%*	*0%*	*0%*	0%	-
0%	-	*0%*	*0%*	*0%*	0%	-
0%	-	*0%*	*0%*	*0%*	0%	-
0%	-	*0%*	*0%*	*0%*	0%	-
0%	-	*0%*	*0%*	*0%*	5%	1,25
0%	-	*0%*	*0%*	*0%*	0%	-
0%	-	*0%*	*0%*	*0%*	0%	-
0%	-	*0%*	*0%*	*0%*	0%	-
0%	-	*0%*	*0%*	*0%*	11%	2,97
0%	-	*0%*	*0%*	*0%*	0%	-
0%	-	*0%*	*0%*	*0%*	0%	-
0%	-	*0%*	*0%*	*0%*	0%	-
0%	-	*0%*	*0%*	*0%*	0%	-
0%	-	*0%*	*0%*	*0%*	0%	-
0%	-	*0%*	*0%*	*0%*	0%	-
0%	-	*0%*	*0%*	*0%*	0%	-
0%	-	*0%*	*0%*	*0%*	0%	-
0%	-	*0%*	*0%*	*0%*	0%	-
0%	-	*0%*	*0%*	*0%*	0%	-
0%	-	*0%*	*0%*	*0%*	0%	-
0%	-	*0%*	*0%*	*0%*	0%	-
0%	-	*0%*	*0%*	*0%*	0%	-
0%	-	*0%*	*0%*	*0%*	0%	-
0%	-	*0%*	*0%*	*0%*	0%	-
0%	-	*0%*	*0%*	*0%*	0%	-
0%	-	*0%*	*0%*	*0%*	0%	-
0%	-	*0%*	*0%*	*0%*	0%	-
0%	-	*0%*	*0%*	*0%*	0%	-
0%	-	*0%*	*0%*	*0%*	0%	-
0%	-	*0%*	*0%*	*0%*	0%	-
0%	-	*0%*	*0%*	*0%*	0%	-
0%	-	*0%*	*0%*	*0%*	0%	-
0%	-	*0%*	*0%*	*0%*	0%	-
0%	-	*0%*	*0%*	*0%*	0%	-
0%	-	*0%*	*0%*	*0%*	0%	-
0%	-	*0%*	*0%*	*0%*	0%	-
0%	-	*0%*	*0%*	*0%*	0%	-
0%	-	*0%*	*0%*	*0%*	0%	-
0%	-	*0%*	*0%*	*0%*	0%	-
0%	-	*0%*	*0%*	*0%*	0%	-
0%	-	*0%*	*0%*	*0%*	0%	-
0%	-	*0%*	*0%*	*0%*	0%	-
0%	-	*0%*	*0%*	*0%*	0%	-
0%	-	*0%*	*0%*	*0%*	0%	-
0%	-	*0%*	*0%*	*0%*	0%	-

0%	-	0%	0%	0%	0%	-
0%	-	0%	0%	0%	0%	-
0%	-	0%	0%	0%	0%	-
0%	-	0%	0%	0%	0%	-
0%	-	0%	0%	0%	0%	-
0%	-	0%	0%	0%	0%	-
0%	-	0%	0%	0%	0%	-
0%	-	0%	0%	0%	0%	-
0%	-	0%	0%	0%	0%	-
0%	-	0%	0%	0%	0%	-
0%	-	0%	0%	0%	0%	-
0%	-	0%	0%	0%	0%	-
0%	-	0%	0%	0%	0%	-
0%	-	0%	0%	0%	0%	-
0%	-	0%	0%	0%	0%	-
0%	-	0%	0%	0%	0%	-
0%	-	0%	0%	0%	0%	-
0%	-	0%	0%	0%	0%	-
0%	-	0%	0%	0%	0%	-
0%	-	0%	0%	0%	0%	-
0%	-	0%	0%	0%	0%	-
0%	-	0%	0%	0%	0%	-
0%	-	0%	0%	0%	0%	-
0%	-	0%	0%	0%	0%	-
0%	-	0%	0%	0%	0%	-
0%	-	0%	0%	0%	0%	-
0%	-	0%	0%	0%	0%	-
0%	-	0%	0%	0%	0%	-
0%	-	0%	0%	0%	0%	-
0%	-	0%	0%	0%	0%	-
0%	-	0%	0%	0%	0%	-
0%	-	0%	0%	0%	0%	-
0%	-	0%	0%	0%	0%	-
0%	-	0%	0%	0%	0%	-
0%	-	0%	0%	0%	0%	-
0%	-	0%	0%	0%	35%	12,39
0%	-	0%	0%	0%	10%	2,22
0%	-	0%	0%	0%	0%	-
0%	-	0%	0%	0%	0%	-
0%	-	0%	0%	0%	0%	-
0%	-	0%	0%	0%	0%	-
0%	-	0%	0%	0%	0%	-
0%	-	0%	0%	0%	0%	-
0%	-	0%	0%	0%	0%	-
0%	-	0%	0%	0%	0%	-
0%	-	0%	0%	0%	0%	-
0%	-	0%	0%	0%	0%	-
0%	-	0%	0%	0%	0%	-
0%	-	0%	0%	0%	0%	-
0%	-	0%	0%	0%	0%	-
0%	-	0%	0%	0%	0%	-
0%	-	0%	0%	0%	0%	-
0%	-	0%	0%	0%	0%	-
0%	-	0%	0%	0%	0%	-
0%	-	0%	0%	0%	0%	-
0%	-	0%	0%	0%	0%	-
0%	-	0%	0%	0%	0%	-
0%	-	0%	0%	0%	0%	-
0%	-	0%	0%	0%	0%	-
0%	-	0%	0%	0%	0%	-
0%	-	0%	0%	0%	0%	-
0%	-	0%	0%	0%	0%	-
0%	-	0%	0%	0%	0%	-
0%	-	0%	0%	0%	0%	-
0%	-	0%	0%	0%	0%	-
0%	-	0%	0%	0%	0%	-

0%	-	0%	0%	0%	0%	-
0%	-	0%	0%	0%	0%	-
0%	-	0%	0%	0%	0%	-
0%	-	0%	0%	0%	0%	-
0%	-	0%	0%	0%	0%	-
0%	-	0%	0%	0%	0%	-
0%	-	0%	0%	0%	0%	-
0%	-	0%	0%	0%	0%	-
0%	-	0%	0%	0%	0%	-
0%	-	0%	0%	0%	0%	-
0%	-	0%	0%	0%	0%	-
0%	-	0%	0%	0%	0%	-
0%	-	0%	0%	0%	0%	-
0%	-	0%	0%	0%	0%	-
0%	-	0%	0%	0%	0%	-
0%	-	0%	0%	0%	0%	-
0%	-	0%	0%	0%	0%	-
0%	-	0%	0%	0%	0%	-
0%	-	0%	0%	0%	0%	-
0%	-	0%	0%	0%	0%	-
0%	-	0%	0%	0%	0%	-
0%	-	0%	0%	0%	0%	-
0%	-	0%	0%	0%	0%	-
0%	-	0%	0%	0%	0%	-
0%	-	0%	0%	0%	0%	-
0%	-	0%	0%	0%	0%	-
0%	-	0%	0%	0%	0%	-
0%	-	0%	0%	0%	0%	-
0%	-	0%	0%	0%	0%	-
0%	-	0%	0%	0%	0%	-
0%	-	0%	0%	0%	0%	-
0%	-	0%	0%	0%	0%	-
0%	-	0%	0%	0%	0%	-
0%	-	0%	0%	0%	0%	-
0%	-	0%	0%	0%	0%	-
0%	-	0%	0%	0%	0%	-
0%	-	0%	0%	0%	0%	-
0%	-	0%	0%	0%	0%	-
0%	-	0%	0%	0%	0%	-
0%	-	0%	0%	0%	0%	-
0%	-	0%	0%	0%	0%	-
0%	-	0%	0%	0%	0%	-
0%	-	0%	0%	0%	0%	-
0%	-	0%	0%	0%	0%	-
0%	-	0%	0%	0%	0%	-
0%	-	0%	0%	0%	0%	-
0%	-	0%	0%	0%	0%	-
0%	-	0%	0%	0%	0%	-
0%	-	0%	0%	0%	0%	-
0%	-	0%	0%	0%	0%	-
0%	-	0%	0%	0%	0%	-
0%	-	0%	0%	0%	0%	-
0%	-	0%	0%	0%	0%	-
0%	-	0%	0%	0%	0%	-
0%	-	0%	0%	0%	0%	-
0%	-	0%	0%	0%	0%	-
0%	-	0%	0%	0%	0%	-
0%	-	0%	0%	0%	0%	-
0%	-	0%	0%	0%	0%	-
0%	-	0%	0%	0%	0%	-
0%	-	0%	0%	0%	0%	-
0%	-	0%	0%	0%	0%	-
0%	-	0%	0%	0%	0%	-
0%	-	0%	0%	0%	0%	-
0%	-	0%	0%	0%	0%	-
0%	-	0%	0%	0%	0%	-

0%	-	0%	0%	0%	0%	-
0%	-	0%	0%	0%	0%	-
0%	-	0%	0%	0%	0%	-
0%	-	0%	0%	0%	0%	-
0%	-	0%	0%	0%	0%	-
0%	-	0%	0%	0%	0%	-
0%	-	0%	0%	0%	0%	-
0%	-	0%	0%	0%	0%	-
0%	-	0%	0%	0%	0%	-
0%	-	0%	0%	0%	0%	-
0%	-	0%	0%	0%	0%	-
0%	-	0%	0%	0%	0%	-
0%	-	0%	0%	0%	0%	-
0%	-	0%	0%	0%	0%	-
0%	-	0%	0%	0%	0%	-
0%	-	0%	0%	0%	0%	-
0%	-	0%	0%	0%	0%	-
0%	-	0%	0%	0%	0%	-
0%	-	0%	0%	0%	0%	-
0%	-	0%	0%	0%	0%	-
0%	-	0%	0%	0%	0%	-
0%	-	0%	0%	0%	0%	-
0%	-	0%	0%	0%	0%	-
0%	-	0%	0%	0%	0%	-
0%	-	0%	0%	0%	0%	-
0%	-	0%	0%	0%	0%	-
0%	-	0%	0%	0%	0%	-
0%	-	0%	0%	0%	0%	-
0%	-	0%	0%	0%	0%	-
0%	-	0%	0%	0%	0%	-
0%	-	0%	0%	0%	0%	-
0%	-	0%	0%	0%	0%	-
0%	-	0%	0%	0%	0%	-
0%	-	0%	0%	0%	0%	-
0%	-	0%	0%	0%	0%	-
0%	-	0%	0%	0%	0%	-
0%	-	0%	0%	0%	0%	-
0%	-	0%	0%	0%	0%	-
0%	-	0%	0%	0%	0%	-
0%	-	0%	0%	0%	0%	-
0%	-	0%	0%	0%	0%	-
0%	-	0%	0%	0%	0%	-
0%	-	0%	0%	0%	0%	-
0%	-	0%	0%	0%	0%	-
0%	-	0%	0%	0%	0%	-
0%	-	0%	0%	0%	0%	-
0%	-	0%	0%	0%	0%	-
0%	-	0%	0%	0%	0%	-
0%	-	0%	0%	0%	0%	-
0%	-	0%	0%	0%	0%	-
0%	-	0%	0%	0%	0%	-
0%	-	0%	0%	0%	0%	-
0%	-	0%	0%	0%	0%	-
0%	-	0%	0%	0%	0%	-
0%	-	0%	0%	0%	0%	-
0%	-	0%	0%	0%	0%	-
0%	-	0%	0%	0%	0%	-
0%	-	0%	0%	0%	0%	-
0%	-	0%	0%	0%	0%	-
0%	-	0%	0%	0%	0%	-
0%	-	0%	0%	0%	0%	-
0%	-	0%	0%	0%	0%	-
0%	-	0%	0%	0%	0%	-
0%	-	0%	0%	0%	0%	-
0%	-	0%	0%	0%	0%	-
0%	-	0%	0%	0%	0%	-

0%	-	*0%*	*0%*	*0%*	0%	-
0%	-	*0%*	*0%*	*0%*	0%	-
0%	-	*0%*	*0%*	*0%*	0%	-
0%	-	*0%*	*0%*	*0%*	0%	-
0%	-	*0%*	*0%*	*0%*	0%	-
0%	-	*0%*	*0%*	*0%*	0%	-
0%	-	*0%*	*0%*	*0%*	0%	-
0%	-	*0%*	*0%*	*0%*	0%	-
0%	-	*0%*	*0%*	*0%*	0%	-
0%	-	*0%*	*0%*	*0%*	0%	-
0%	-	*0%*	*0%*	*0%*	0%	-
0%	-	*0%*	*0%*	*0%*	0%	-
0%	-	*0%*	*0%*	*0%*	0%	-
0%	-	*0%*	*0%*	*0%*	0%	-
0%	-	*0%*	*0%*	*0%*	0%	-
0%	-	*0%*	*0%*	*0%*	0%	-
0%	-	*0%*	*0%*	*0%*	0%	-
0%	-	*0%*	*0%*	*0%*	0%	-
0%	-	*0%*	*0%*	*0%*	0%	-
0%	-	*0%*	*0%*	*0%*	0%	-
0%	-	*0%*	*0%*	*0%*	0%	-
0%	-	*0%*	*0%*	*0%*	0%	-
0%	-	*0%*	*0%*	*0%*	0%	-
0%	-	*0%*	*0%*	*0%*	0%	-
0%	-	*0%*	*0%*	*0%*	0%	-
0%	-	*0%*	*0%*	*0%*	0%	-
0%	-	*0%*	*0%*	*0%*	0%	-
0%	-	*0%*	*0%*	*0%*	0%	-
0%	-	*0%*	*0%*	*0%*	0%	-
0%	-	*0%*	*0%*	*0%*	0%	-
0%	-	*0%*	*0%*	*0%*	0%	-
0%	-	*0%*	*0%*	*0%*	0%	-
0%	-	*0%*	*0%*	*0%*	0%	-
0%	-	*0%*	*0%*	*0%*	0%	-
0%	-	*0%*	*0%*	*0%*	0%	-
0%	-	*0%*	*0%*	*0%*	0%	-
0%	-	*0%*	*0%*	*0%*	0%	-
0%	-	*0%*	*0%*	*0%*	0%	-
0%	-	*0%*	*0%*	*0%*	0%	-
0%	-	*0%*	*0%*	*0%*	0%	-
0%	-	*0%*	*0%*	*0%*	0%	-
0%	-	*0%*	*0%*	*0%*	10%	2,50
0%	-	*0%*	*0%*	*0%*	0%	-
0%	-	*0%*	*0%*	*0%*	0%	-
0%	-	*0%*	*0%*	*0%*	0%	-
0%	-	*0%*	*0%*	*0%*	0%	-
0%	-	*0%*	*0%*	*0%*	0%	-
0%	-	*0%*	*0%*	*0%*	0%	-
0%	-	*0%*	*0%*	*0%*	0%	-
0%	-	*0%*	*0%*	*0%*	0%	-
0%	-	*0%*	*0%*	*0%*	0%	-
0%	-	*0%*	*0%*	*0%*	0%	-
0%	-	*0%*	*0%*	*0%*	0%	-
0%	-	*0%*	*0%*	*0%*	0%	-
0%	-	*0%*	*0%*	*0%*	0%	-
0%	-	*0%*	*0%*	*0%*	0%	-
0%	-	*0%*	*0%*	*0%*	0%	-
0%	-	*0%*	*0%*	*0%*	0%	-
0%	-	*0%*	*0%*	*0%*	0%	-
0%	-	*0%*	*0%*	*0%*	0%	-
0%	-	*0%*	*0%*	*0%*	0%	-
0%	-	*0%*	*0%*	*0%*	0%	-
0%	-	*0%*	*0%*	*0%*	0%	-
0%	-	*0%*	*0%*	*0%*	0%	-
0%	-	*0%*	*0%*	*0%*	0%	-
0%	-	*0%*	*0%*	*0%*	0%	-

0%	-	0%	0%	0%	0%	-
0%	-	0%	0%	0%	0%	-
0%	-	0%	0%	0%	0%	-
0%	-	0%	0%	0%	0%	-
0%	-	0%	0%	0%	0%	-
0%	-	0%	0%	0%	0%	-
0%	-	0%	0%	0%	0%	-
0%	-	0%	0%	0%	0%	-
0%	-	0%	0%	0%	0%	-
0%	-	0%	0%	0%	0%	-
0%	-	0%	0%	0%	0%	-
0%	-	0%	0%	0%	0%	-
0%	-	0%	0%	0%	0%	-
0%	-	0%	0%	0%	0%	-
0%	-	0%	0%	0%	0%	-
0%	-	0%	0%	0%	0%	-
0%	-	0%	0%	0%	0%	-
0%	-	0%	0%	0%	0%	-
0%	-	0%	0%	0%	0%	-
0%	-	0%	0%	0%	0%	-
0%	-	0%	0%	0%	0%	-
0%	-	0%	0%	0%	0%	-
0%	-	0%	0%	0%	0%	-
0%	-	0%	0%	0%	0%	-
0%	-	0%	0%	0%	0%	-
0%	-	0%	0%	0%	0%	-
0%	-	0%	0%	0%	0%	-
0%	-	0%	0%	0%	0%	-
0%	-	0%	0%	0%	0%	-
0%	-	0%	0%	0%	0%	-
0%	-	0%	0%	0%	0%	-
0%	-	0%	0%	0%	0%	-
0%	-	0%	0%	0%	0%	-
0%	-	0%	0%	0%	0%	-
0%	-	0%	0%	0%	0%	-
0%	-	0%	0%	0%	0%	-
0%	-	0%	0%	0%	0%	-
0%	-	0%	0%	0%	0%	-
0%	-	0%	0%	0%	0%	-
0%	-	0%	0%	0%	0%	-
0%	-	0%	0%	0%	0%	-
0%	-	0%	0%	0%	0%	-
0%	-	0%	0%	0%	0%	-
0%	-	0%	0%	0%	0%	-
0%	-	0%	0%	0%	0%	-
0%	-	0%	0%	0%	0%	-
0%	-	0%	0%	0%	0%	-
0%	-	0%	0%	0%	0%	-
0%	-	0%	0%	0%	0%	-
0%	-	0%	0%	0%	0%	-
0%	-	0%	0%	0%	0%	-
0%	-	0%	0%	0%	0%	-
0%	-	0%	0%	0%	0%	-
0%	-	0%	0%	0%	0%	-
0%	-	0%	0%	0%	0%	-
0%	-	0%	0%	0%	0%	-
0%	-	0%	0%	0%	0%	-
0%	-	0%	0%	0%	0%	-
0%	-	0%	0%	0%	0%	-
0%	-	0%	0%	0%	0%	-
0%	-	0%	0%	0%	0%	-
0%	-	0%	0%	0%	0%	-
0%	-	0%	0%	0%	0%	-
0%	-	0%	0%	0%	0%	-
0%	-	0%	0%	0%	0%	-
0%	-	0%	0%	0%	0%	-

0%	-	*0%*	*0%*	*0%*	0%	-
0%	-	*0%*	*0%*	*0%*	0%	-
0%	-	*0%*	*0%*	*0%*	0%	-
0%	-	*0%*	*0%*	*0%*	0%	-
0%	-	*0%*	*0%*	*0%*	0%	-
0%	-	*0%*	*0%*	*0%*	0%	-
0%	-	*0%*	*0%*	*0%*	0%	-
0%	-	*0%*	*0%*	*0%*	0%	-
0%	-	*0%*	*0%*	*0%*	0%	-
0%	-	*0%*	*0%*	*0%*	0%	-
0%	-	*0%*	*0%*	*0%*	0%	-
0%	-	*0%*	*0%*	*0%*	0%	-
0%	-	*0%*	*0%*	*0%*	0%	-
0%	-	*0%*	*0%*	*0%*	0%	-
0%	-	*0%*	*0%*	*0%*	0%	-
0%	-	*0%*	*0%*	*0%*	0%	-
0%	-	*0%*	*0%*	*0%*	0%	-
0%	-	*0%*	*0%*	*0%*	0%	-
0%	-	*0%*	*0%*	*0%*	0%	-
0%	-	*0%*	*0%*	*0%*	0%	-
0%	-	*0%*	*0%*	*0%*	0%	-
0%	-	*0%*	*0%*	*0%*	0%	-
0%	-	*0%*	*0%*	*0%*	0%	-
0%	-	*0%*	*0%*	*0%*	0%	-
0%	-	*0%*	*0%*	*0%*	0%	-
0%	-	*0%*	*0%*	*0%*	0%	-
0%	-	*0%*	*0%*	*0%*	0%	-
0%	-	*0%*	*0%*	*0%*	0%	-
0%	-	*0%*	*0%*	*0%*	0%	-
0%	-	*0%*	*0%*	*0%*	0%	-
0%	-	*0%*	*0%*	*0%*	0%	-
0%	-	*0%*	*0%*	*0%*	0%	-
0%	-	*0%*	*0%*	*0%*	0%	-
0%	-	*0%*	*0%*	*0%*	0%	-
0%	-	*0%*	*0%*	*0%*	0%	-
0%	-	*0%*	*0%*	*0%*	0%	-
0%	-	*0%*	*0%*	*0%*	0%	-
0%	-	*0%*	*0%*	*0%*	0%	-
0%	-	*0%*	*0%*	*0%*	0%	-
0%	-	*0%*	*0%*	*0%*	0%	-
0%	-	*0%*	*0%*	*0%*	0%	-
0%	-	*0%*	*0%*	*0%*	0%	-
0%	-	*0%*	*0%*	*0%*	0%	-
0%	-	*0%*	*0%*	*0%*	0%	-
0%	-	*0%*	*0%*	*0%*	0%	-
0%	-	*0%*	*0%*	*0%*	0%	-
0%	-	*0%*	*0%*	*0%*	0%	-
0%	-	*0%*	*0%*	*0%*	0%	-
0%	-	*0%*	*0%*	*0%*	0%	-
0%	-	*0%*	*0%*	*0%*	0%	-
0%	-	*0%*	*0%*	*0%*	0%	-
0%	-	*0%*	*0%*	*0%*	0%	-
0%	-	*0%*	*0%*	*0%*	0%	-
0%	-	*0%*	*0%*	*0%*	0%	-
0%	-	*0%*	*0%*	*0%*	0%	-
0%	-	*0%*	*0%*	*0%*	0%	-
0%	-	*0%*	*0%*	*0%*	0%	-
0%	-	*0%*	*0%*	*0%*	0%	-
0%	-	*0%*	*0%*	*0%*	0%	-
0%	-	*0%*	*0%*	*0%*	0%	-
0%	-	*0%*	*0%*	*0%*	0%	-
0%	-	*0%*	*0%*	*0%*	0%	-
0%	-	*0%*	*0%*	*0%*	0%	-
0%	-	*0%*	*0%*	*0%*	0%	-
0%	-	*0%*	*0%*	*0%*	0%	-
0%	-	*0%*	*0%*	*0%*	0%	-

0%	-	*0%*	*0%*	*0%*	0%	-
0%	-	*0%*	*0%*	*0%*	0%	-
0%	-	*0%*	*0%*	*0%*	0%	-
0%	-	*0%*	*0%*	*0%*	0%	-
0%	-	*0%*	*0%*	*0%*	0%	-
0%	-	*0%*	*0%*	*0%*	0%	-
0%	-	*0%*	*0%*	*0%*	0%	-
0%	-	*0%*	*0%*	*0%*	0%	-
0%	-	*0%*	*0%*	*0%*	0%	-
0%	-	*0%*	*0%*	*0%*	0%	-
0%	-	*0%*	*0%*	*0%*	0%	-
0%	-	*0%*	*0%*	*0%*	0%	-
0%	-	*0%*	*0%*	*0%*	0%	-
0%	-	*0%*	*0%*	*0%*	0%	-
0%	-	*0%*	*0%*	*0%*	0%	-
0%	-	*0%*	*0%*	*0%*	0%	-
0%	-	*0%*	*0%*	*0%*	0%	-
0%	-	*0%*	*0%*	*0%*	0%	-
0%	-	*0%*	*0%*	*0%*	0%	-
0%	-	*0%*	*0%*	*0%*	0%	-
0%	-	*0%*	*0%*	*0%*	0%	-
0%	-	*0%*	*0%*	*0%*	0%	-
0%	-	*0%*	*0%*	*0%*	0%	-
0%	-	*0%*	*0%*	*0%*	0%	-
0%	-	*0%*	*0%*	*0%*	0%	-
0%	-	*0%*	*0%*	*0%*	0%	-
0%	-	*0%*	*0%*	*0%*	0%	-
0%	-	*0%*	*0%*	*0%*	0%	-
0%	-	*0%*	*0%*	*0%*	0%	-
0%	-	*0%*	*0%*	*0%*	0%	-
0%	-	*0%*	*0%*	*0%*	0%	-
0%	-	*0%*	*0%*	*0%*	0%	-
0%	-	*0%*	*0%*	*0%*	0%	-
0%	-	*0%*	*0%*	*0%*	0%	-
0%	-	*0%*	*0%*	*0%*	0%	-
0%	-	*0%*	*0%*	*0%*	0%	-
0%	-	*0%*	*0%*	*0%*	0%	-
0%	-	*0%*	*0%*	*0%*	0%	-
0%	-	*0%*	*0%*	*0%*	0%	-
0%	-	*0%*	*0%*	*0%*	0%	-
0%	-	*0%*	*0%*	*0%*	0%	-
0%	-	*0%*	*0%*	*0%*	0%	-
0%	-	*0%*	*0%*	*0%*	0%	-
0%	-	*0%*	*0%*	*0%*	0%	-
0%	-	*0%*	*0%*	*0%*	0%	-
0%	-	*0%*	*0%*	*0%*	0%	-
0%	-	*0%*	*0%*	*0%*	0%	-
0%	-	*0%*	*0%*	*0%*	0%	-
0%	-	*0%*	*0%*	*0%*	0%	-
0%	-	*0%*	*0%*	*0%*	0%	-
0%	-	*0%*	*0%*	*0%*	0%	-
0%	-	*0%*	*0%*	*0%*	0%	-
0%	-	*0%*	*0%*	*0%*	0%	-
0%	-	*0%*	*0%*	*0%*	0%	-
0%	-	*0%*	*0%*	*0%*	0%	-
0%	-	*0%*	*0%*	*0%*	0%	-
0%	-	*0%*	*0%*	*0%*	0%	-
0%	-	*0%*	*0%*	*0%*	0%	-
0%	-	*0%*	*0%*	*0%*	0%	-
0%	-	*0%*	*0%*	*0%*	0%	-
0%	-	*0%*	*0%*	*0%*	0%	-
0%	-	*0%*	*0%*	*0%*	0%	-
0%	-	*0%*	*0%*	*0%*	0%	-
0%	-	*0%*	*0%*	*0%*	0%	-
0%	-	*0%*	*0%*	*0%*	0%	-
0%	-	*0%*	*0%*	*0%*	0%	-

0%	-	*0%*	*0%*	*0%*	0%	-
0%	-	*0%*	*0%*	*0%*	0%	-
0%	-	*0%*	*0%*	*0%*	0%	-
0%	-	*0%*	*0%*	*0%*	0%	-
0%	-	*0%*	*0%*	*0%*	0%	-
0%	-	*0%*	*0%*	*0%*	0%	-
0%	-	*0%*	*0%*	*0%*	0%	-
0%	-	*0%*	*0%*	*0%*	0%	-
0%	-	*0%*	*0%*	*0%*	0%	-
0%	-	*0%*	*0%*	*0%*	0%	-
0%	-	*0%*	*0%*	*0%*	82%	12,79
0%	-	*0%*	*0%*	*0%*	0%	-
0%	-	*0%*	*0%*	*0%*	0%	-
0%	-	*0%*	*0%*	*0%*	0%	-
0%	-	*0%*	*0%*	*0%*	0%	-
0%	-	*0%*	*0%*	*0%*	0%	-
0%	-	*0%*	*0%*	*0%*	0%	-
0%	-	*0%*	*0%*	*0%*	0%	-
0%	-	*0%*	*0%*	*0%*	19%	6,08
0%	-	*0%*	*0%*	*0%*	0%	-
0%	-	*0%*	*0%*	*0%*	100%	5,00
0%	-	*0%*	*0%*	*0%*	0%	-
0%	-	*0%*	*0%*	*0%*	0%	-
0%	-	*0%*	*0%*	*0%*	0%	-
0%	-	*0%*	*0%*	*0%*	0%	-
0%	-	*0%*	*0%*	*0%*	0%	-

% Algae win	% Neutral	% Coral wins	Halimeda % of interaction	Total length in interaction with Halimeda	% Algae win	% Neutral	% Coral wins
0%	0%	0%	0%	-	0%	0%	0%
0%	0%	0%	0%	-	0%	0%	0%
0%	0%	0%	0%	-	0%	0%	0%
0%	0%	0%	0%	-	0%	0%	0%
0%	0%	0%	0%	-	0%	0%	0%
0%	0%	0%	0%	-	0%	0%	0%
0%	0%	0%	0%	-	0%	0%	0%
0%	0%	0%	0%	-	0%	0%	0%
0%	0%	0%	0%	-	0%	0%	0%
0%	0%	0%	0%	-	0%	0%	0%
0%	0%	0%	0%	-	0%	0%	0%
0%	0%	0%	0%	-	0%	0%	0%
0%	0%	0%	0%	-	0%	0%	0%
0%	0%	0%	0%	-	0%	0%	0%
0%	0%	0%	0%	-	0%	0%	0%
0%	0%	0%	0%	-	0%	0%	0%
0%	0%	0%	0%	-	0%	0%	0%
0%	0%	0%	0%	-	0%	0%	0%
0%	0%	0%	0%	-	0%	0%	0%
100%	0%	0%	0%	-	0%	0%	0%
0%	0%	0%	0%	-	0%	0%	0%
0%	0%	0%	0%	-	0%	0%	0%
0%	0%	0%	0%	-	0%	0%	0%
0%	0%	0%	0%	-	0%	0%	0%
50%	50%	0%	0%	-	0%	0%	0%
0%	0%	0%	0%	-	0%	0%	0%
0%	0%	0%	0%	-	0%	0%	0%
0%	100%	0%	0%	-	0%	0%	0%
0%	0%	0%	0%	-	0%	0%	0%
20%	80%	0%	0%	-	0%	0%	0%
0%	0%	0%	0%	-	0%	0%	0%
0%	0%	0%	0%	-	0%	0%	0%
0%	0%	0%	0%	-	0%	0%	0%
0%	0%	0%	0%	-	0%	0%	0%
0%	0%	0%	0%	-	0%	0%	0%
0%	0%	0%	0%	-	0%	0%	0%
0%	0%	0%	0%	-	0%	0%	0%
0%	0%	0%	0%	-	0%	0%	0%
100%	0%	0%	0%	-	0%	0%	0%
0%	100%	0%	0%	-	0%	0%	0%
0%	0%	0%	20%	25,96	60%	40%	0%
0%	0%	0%	0%	-	0%	0%	0%
0%	0%	0%	0%	-	0%	0%	0%
0%	100%	0%	0%	-	0%	0%	0%
0%	0%	0%	0%	-	0%	0%	0%
0%	0%	0%	0%	-	0%	0%	0%
0%	0%	0%	0%	-	0%	0%	0%
0%	0%	0%	0%	-	0%	0%	0%
0%	0%	0%	0%	-	0%	0%	0%
0%	0%	0%	0%	-	0%	0%	0%
0%	0%	0%	0%	-	0%	0%	0%
100%	0%	0%	0%	-	0%	0%	0%
60%	40%	0%	20%	20,80	0%	100%	0%
0%	0%	0%	0%	-	0%	0%	0%
0%	0%	0%	0%	-	0%	0%	0%
0%	0%	0%	0%	-	0%	0%	0%
0%	0%	0%	0%	-	0%	0%	0%
100%	0%	0%	0%	-	0%	0%	0%
0%	0%	0%	0%	-	0%	0%	0%
0%	0%	0%	0%	-	0%	0%	0%
0%	0%	0%	0%	-	0%	0%	0%
0%	0%	0%	0%	-	0%	0%	0%

0%	0%	0%	0%	-	0%	0%	0%
0%	0%	0%	0%	-	0%	0%	0%
0%	0%	0%	0%	-	0%	0%	0%
0%	0%	0%	0%	-	0%	0%	0%
0%	0%	0%	0%	-	0%	0%	0%
0%	0%	0%	0%	-	0%	0%	0%
0%	0%	0%	5%	1,83	0%	100%	0%
0%	0%	0%	0%	-	0%	0%	0%
0%	0%	0%	0%	-	0%	0%	0%
0%	0%	0%	0%	-	0%	0%	0%
0%	0%	0%	0%	-	0%	0%	0%
0%	0%	0%	0%	-	0%	0%	0%
0%	0%	0%	0%	-	0%	0%	0%
0%	0%	0%	0%	-	0%	0%	0%
0%	0%	0%	0%	-	0%	0%	0%
100%	0%	0%	14%	5,38	0%	100%	0%
0%	0%	0%	0%	-	0%	0%	0%
0%	0%	0%	0%	-	0%	0%	0%
0%	0%	0%	0%	-	0%	0%	0%
0%	0%	0%	15%	3,60	35%	30%	35%
0%	0%	0%	0%	-	0%	0%	0%
0%	0%	0%	0%	-	0%	0%	0%
0%	0%	0%	0%	-	0%	0%	0%
0%	0%	0%	0%	-	0%	0%	0%
0%	0%	0%	10%	0,38	0%	100%	0%
0%	0%	0%	0%	-	0%	0%	0%
0%	0%	0%	0%	-	0%	0%	0%
0%	0%	0%	0%	-	0%	0%	0%
0%	0%	0%	0%	-	0%	0%	0%
0%	0%	0%	0%	-	0%	0%	0%
0%	0%	0%	0%	-	0%	0%	0%
0%	0%	0%	0%	-	0%	0%	0%
0%	0%	0%	0%	-	0%	0%	0%
0%	0%	0%	0%	-	0%	0%	0%
0%	0%	0%	0%	-	0%	0%	0%
0%	0%	0%	0%	-	0%	0%	0%
0%	0%	0%	0%	-	0%	0%	0%
0%	0%	0%	0%	-	0%	0%	0%
50%	50%	0%	0%	-	0%	0%	0%
0%	0%	0%	0%	-	0%	0%	0%
0%	0%	0%	0%	-	0%	0%	0%
0%	0%	0%	100%	12,42	0%	0%	100%
0%	0%	0%	0%	-	0%	0%	0%
0%	0%	0%	0%	-	0%	0%	0%
0%	0%	0%	0%	-	0%	0%	0%
100%	0%	0%	0%	-	0%	0%	0%
0%	0%	0%	0%	-	0%	0%	0%
0%	0%	0%	0%	-	0%	0%	0%
0%	0%	0%	0%	-	0%	0%	0%
0%	0%	0%	0%	-	0%	0%	0%
0%	0%	0%	0%	-	0%	0%	0%
0%	0%	0%	0%	-	0%	0%	0%
0%	0%	0%	0%	-	0%	0%	0%
0%	0%	0%	0%	-	0%	0%	0%
0%	0%	0%	0%	-	0%	0%	0%
0%	0%	0%	0%	-	0%	0%	0%
0%	0%	0%	0%	-	0%	0%	0%
0%	0%	0%	0%	-	0%	0%	0%
0%	0%	0%	0%	-	0%	0%	0%
0%	0%	0%	0%	-	0%	0%	0%
0%	0%	0%	0%	-	0%	0%	0%
0%	0%	0%	0%	-	0%	0%	0%
0%	0%	0%	0%	-	0%	0%	0%
0%	0%	0%	0%	-	0%	0%	0%
0%	0%	0%	0%	-	0%	0%	0%
0%	0%	0%	0%	-	0%	0%	0%

0%	0%	0%	0%	-	0%	0%	0%
0%	0%	0%	0%	-	0%	0%	0%
0%	0%	0%	0%	-	0%	0%	0%
0%	0%	0%	0%	-	0%	0%	0%
0%	0%	0%	0%	-	0%	0%	0%
0%	0%	0%	0%	-	0%	0%	0%
0%	0%	0%	0%	-	0%	0%	0%
0%	0%	0%	0%	-	0%	0%	0%
0%	0%	0%	0%	-	0%	0%	0%
0%	0%	0%	0%	-	0%	0%	0%
0%	0%	0%	0%	-	0%	0%	0%
0%	0%	0%	0%	-	0%	0%	0%
0%	0%	0%	0%	-	0%	0%	0%
0%	0%	0%	0%	-	0%	0%	0%
0%	0%	0%	0%	-	0%	0%	0%
0%	0%	0%	0%	-	0%	0%	0%
0%	0%	0%	0%	-	0%	0%	0%
0%	0%	0%	0%	-	0%	0%	0%
0%	0%	0%	0%	-	0%	0%	0%
0%	0%	0%	0%	-	0%	0%	0%
0%	0%	0%	0%	-	0%	0%	0%
0%	0%	0%	0%	-	0%	0%	0%
0%	0%	0%	0%	-	0%	0%	0%
0%	0%	0%	0%	-	0%	0%	0%
0%	0%	0%	0%	-	0%	0%	0%
0%	0%	0%	0%	-	0%	0%	0%
0%	0%	0%	0%	-	0%	0%	0%
0%	0%	0%	0%	-	0%	0%	0%
0%	0%	0%	0%	-	0%	0%	0%
0%	0%	0%	0%	-	0%	0%	0%
0%	0%	0%	0%	-	0%	0%	0%
0%	0%	0%	0%	-	0%	0%	0%
0%	0%	0%	0%	-	0%	0%	0%
0%	0%	0%	0%	-	0%	0%	0%
0%	0%	0%	0%	-	0%	0%	0%
0%	0%	0%	0%	-	0%	0%	0%
0%	0%	0%	0%	-	0%	0%	0%
0%	0%	0%	0%	-	0%	0%	0%
0%	0%	0%	0%	-	0%	0%	0%
0%	0%	0%	0%	-	0%	0%	0%
0%	0%	0%	0%	-	0%	0%	0%
0%	0%	0%	0%	-	0%	0%	0%
0%	0%	0%	30%	-	0%	100%	0%
0%	0%	0%	0%	-	0%	0%	0%
0%	0%	0%	0%	-	0%	0%	0%
0%	0%	0%	0%	-	0%	0%	0%
0%	0%	0%	0%	-	0%	0%	0%
100%	0%	0%	0%	-	0%	0%	0%
0%	0%	0%	0%	-	0%	0%	0%
0%	0%	0%	0%	-	0%	0%	0%
0%	0%	0%	0%	-	0%	0%	0%
0%	0%	0%	0%	-	0%	0%	0%
0%	0%	0%	0%	-	0%	0%	0%
0%	0%	0%	0%	-	0%	0%	0%
0%	0%	0%	0%	-	0%	0%	0%
0%	0%	0%	0%	-	0%	0%	0%
0%	0%	0%	0%	-	0%	0%	0%
0%	0%	0%	0%	-	0%	0%	0%
0%	0%	0%	0%	-	0%	0%	0%
0%	0%	0%	0%	-	0%	0%	0%
0%	0%	0%	0%	-	0%	0%	0%
0%	0%	0%	0%	-	0%	0%	0%
0%	0%	0%	0%	-	0%	0%	0%
0%	0%	0%	0%	-	0%	0%	0%
0%	0%	0%	0%	-	0%	0%	0%
0%	0%	0%	0%	-	0%	0%	0%

0%	0%	0%	0%	-	0%	0%	0%
0%	0%	0%	0%	-	0%	0%	0%
0%	0%	0%	0%	-	0%	0%	0%
0%	0%	0%	0%	-	0%	0%	0%
0%	0%	0%	0%	-	0%	0%	0%
0%	0%	0%	0%	-	0%	0%	0%
0%	0%	0%	0%	-	0%	0%	0%
0%	0%	0%	0%	-	0%	0%	0%
0%	0%	0%	0%	-	0%	0%	0%
0%	0%	0%	0%	-	0%	0%	0%
15%	70%	15%	10%	10,12	15%	70%	15%
0%	0%	0%	0%	-	0%	0%	0%
0%	0%	0%	0%	-	0%	0%	0%
0%	0%	0%	0%	-	0%	0%	0%
0%	0%	0%	0%	-	0%	0%	0%
0%	0%	0%	0%	-	0%	0%	0%
0%	0%	0%	0%	-	0%	0%	0%
0%	0%	0%	0%	-	0%	0%	0%
0%	0%	0%	0%	-	0%	0%	0%
0%	0%	0%	0%	-	0%	0%	0%
0%	0%	0%	0%	-	0%	0%	0%
0%	0%	0%	0%	-	0%	0%	0%
0%	0%	0%	0%	-	0%	0%	0%
0%	100%	0%	0%	-	0%	0%	0%
0%	0%	0%	0%	-	0%	0%	0%
0%	0%	0%	0%	-	0%	0%	0%
0%	0%	0%	0%	-	0%	0%	0%
0%	0%	0%	0%	-	0%	0%	0%
0%	0%	0%	10%	1,90	100%	0%	0%
0%	0%	0%	0%	-	0%	0%	0%
0%	0%	0%	0%	-	0%	0%	0%
0%	0%	0%	0%	-	0%	0%	0%
0%	0%	0%	0%	-	0%	0%	0%
0%	0%	0%	0%	-	0%	0%	0%
0%	0%	0%	0%	-	0%	0%	0%
0%	0%	0%	0%	-	0%	0%	0%
0%	0%	0%	0%	-	0%	0%	0%
0%	0%	0%	0%	-	0%	0%	0%
0%	0%	0%	0%	-	0%	0%	0%
0%	0%	0%	0%	-	0%	0%	0%
0%	0%	0%	0%	-	0%	0%	0%
0%	0%	0%	0%	-	0%	0%	0%
0%	0%	0%	0%	-	0%	0%	0%
0%	0%	0%	0%	-	0%	0%	0%
0%	0%	0%	0%	-	0%	0%	0%
0%	0%	0%	0%	-	0%	0%	0%
0%	100%	0%	0%	-	0%	0%	0%
0%	0%	0%	20%	2,60	100%	0%	0%
0%	100%	0%	0%	-	0%	0%	0%
0%	0%	0%	0%	-	0%	0%	0%
0%	100%	0%	0%	-	0%	0%	0%
0%	0%	0%	0%	-	0%	0%	0%
0%	0%	0%	0%	-	0%	0%	0%
0%	0%	0%	0%	-	0%	0%	0%
0%	0%	0%	0%	-	0%	0%	0%
0%	0%	0%	0%	-	0%	0%	0%
0%	0%	0%	0%	-	0%	0%	0%
0%	0%	0%	0%	-	0%	0%	0%
0%	0%	0%	0%	-	0%	0%	0%
0%	0%	0%	0%	-	0%	0%	0%
0%	0%	0%	0%	-	0%	0%	0%
0%	0%	0%	0%	-	0%	0%	0%
0%	0%	0%	0%	-	0%	0%	0%
0%	0%	0%	0%	-	0%	0%	0%
0%	0%	0%	0%	-	0%	0%	0%
0%	0%	0%	0%	-	0%	0%	0%

0%	0%	0%	0%	-	0%	0%	0%
0%	0%	0%	0%	-	0%	0%	0%
0%	0%	0%	0%	-	0%	0%	0%
0%	0%	0%	0%	-	0%	0%	0%
0%	0%	0%	0%	-	0%	0%	0%
0%	0%	0%	0%	-	0%	0%	0%
0%	0%	0%	0%	-	0%	0%	0%
0%	0%	0%	0%	-	0%	0%	0%
0%	0%	0%	0%	-	0%	0%	0%
0%	0%	0%	0%	-	0%	0%	0%
0%	0%	0%	0%	-	0%	0%	0%
0%	0%	0%	0%	-	0%	0%	0%
0%	0%	0%	0%	-	0%	0%	0%
0%	0%	0%	0%	-	0%	0%	0%
0%	0%	0%	0%	-	0%	0%	0%
0%	0%	0%	0%	-	0%	0%	0%
0%	0%	0%	0%	-	0%	0%	0%
0%	0%	0%	0%	-	0%	0%	0%
0%	0%	0%	0%	-	0%	0%	0%
0%	0%	0%	0%	-	0%	0%	0%
0%	0%	0%	0%	-	0%	0%	0%
0%	0%	0%	0%	-	0%	0%	0%
0%	0%	0%	0%	-	0%	0%	0%
0%	0%	0%	0%	-	0%	0%	0%
0%	0%	0%	0%	-	0%	0%	0%
0%	0%	0%	0%	-	0%	0%	0%
0%	0%	0%	0%	-	0%	0%	0%
100%	0%	0%	0%	-	0%	0%	0%
0%	0%	0%	0%	-	0%	0%	0%
0%	0%	0%	0%	-	0%	0%	0%
0%	0%	0%	0%	-	0%	0%	0%
0%	0%	0%	0%	-	0%	0%	0%
0%	0%	0%	0%	-	0%	0%	0%
100%	0%	0%	0%	-	0%	0%	0%
0%	0%	0%	0%	-	0%	0%	0%
0%	0%	0%	0%	-	0%	0%	0%
0%	0%	0%	0%	-	0%	0%	0%
0%	0%	0%	0%	-	0%	0%	0%
0%	0%	0%	0%	-	0%	0%	0%
0%	0%	0%	0%	-	0%	0%	0%
0%	0%	0%	0%	-	0%	0%	0%
0%	0%	0%	0%	-	0%	0%	0%
0%	0%	0%	0%	-	0%	0%	0%
0%	0%	0%	0%	-	0%	0%	0%
0%	0%	0%	0%	-	0%	0%	0%
0%	0%	0%	0%	-	0%	0%	0%
0%	0%	0%	0%	-	0%	0%	0%
0%	0%	0%	0%	-	0%	0%	0%
0%	0%	0%	0%	-	0%	0%	0%
100%	0%	0%	0%	-	0%	0%	0%
0%	0%	0%	0%	-	0%	0%	0%
100%	0%	0%	0%	-	0%	0%	0%
0%	0%	0%	0%	-	0%	0%	0%
0%	0%	0%	0%	-	0%	0%	0%
0%	0%	0%	0%	-	0%	0%	0%
0%	0%	0%	0%	-	0%	0%	0%
100%	0%	0%	0%	-	0%	0%	0%
0%	0%	0%	0%	-	0%	0%	0%
0%	0%	0%	0%	-	0%	0%	0%
0%	0%	0%	0%	-	0%	0%	0%
100%	0%	0%	0%	-	0%	0%	0%
0%	0%	0%	0%	-	0%	0%	0%
0%	0%	0%	0%	-	0%	0%	0%
100%	0%	0%	0%	-	0%	0%	0%
0%	0%	0%	0%	-	0%	0%	0%
0%	0%	0%	0%	-	0%	0%	0%

100%	0%	0%	10%	8,08	100%	0%	0%
0%	0%	0%	0%	-	0%	0%	0%
0%	0%	0%	0%	-	0%	0%	0%
0%	100%	0%	0%	-	0%	0%	0%
0%	0%	0%	0%	-	0%	0%	0%
0%	0%	0%	0%	-	0%	0%	0%
0%	0%	0%	0%	-	0%	0%	0%
0%	0%	0%	0%	-	0%	0%	0%
0%	0%	0%	0%	-	0%	0%	0%
0%	0%	0%	30%	6,61	100%	0%	0%
0%	0%	0%	0%	-	0%	0%	0%
0%	100%	0%	0%	-	0%	0%	0%
0%	0%	0%	0%	-	0%	0%	0%
0%	0%	0%	10%	4,30	0%	100%	0%
0%	0%	0%	0%	-	0%	0%	0%
0%	0%	0%	0%	-	0%	0%	0%
0%	0%	0%	0%	-	0%	0%	0%
0%	0%	0%	0%	-	0%	0%	0%
0%	0%	0%	20%	2,98	0%	100%	0%
0%	0%	0%	0%	-	0%	0%	0%
100%	0%	0%	0%	-	0%	0%	0%
0%	0%	0%	0%	-	0%	0%	0%
0%	0%	0%	0%	-	0%	0%	0%
0%	0%	0%	0%	-	0%	0%	0%
0%	0%	0%	0%	-	0%	0%	0%
0%	0%	0%	0%	-	0%	0%	0%
0%	0%	0%	0%	-	0%	0%	0%
0%	0%	0%	0%	-	0%	0%	0%
0%	0%	0%	0%	-	0%	0%	0%
0%	0%	0%	0%	-	0%	0%	0%
0%	0%	0%	0%	-	0%	0%	0%
0%	0%	0%	0%	-	0%	0%	0%
0%	0%	0%	0%	-	0%	0%	0%
0%	0%	0%	0%	-	0%	0%	0%
0%	0%	0%	0%	-	0%	0%	0%
0%	0%	0%	0%	-	0%	0%	0%
0%	0%	0%	0%	-	0%	0%	0%
0%	0%	0%	0%	-	0%	0%	0%
0%	0%	0%	0%	-	0%	0%	0%
0%	0%	0%	0%	-	0%	0%	0%
0%	0%	0%	0%	-	0%	0%	0%
0%	0%	0%	0%	-	0%	0%	0%
0%	0%	0%	0%	-	0%	0%	0%
0%	0%	0%	0%	-	0%	0%	0%
0%	0%	0%	0%	-	0%	0%	0%
0%	0%	0%	0%	-	0%	0%	0%
0%	0%	0%	0%	-	0%	0%	0%
0%	0%	0%	0%	-	0%	0%	0%
0%	0%	0%	0%	-	0%	0%	0%
0%	0%	0%	0%	-	0%	0%	0%
0%	100%	0%	0%	-	0%	0%	0%
50%	50%	0%	5%	3,35	60%	40%	0%
0%	0%	0%	10%	2,40	100%	0%	0%
0%	0%	0%	0%	-	0%	0%	0%
0%	0%	0%	0%	-	0%	0%	0%
0%	0%	0%	0%	-	0%	0%	0%
0%	0%	0%	0%	-	0%	0%	0%
0%	0%	0%	0%	-	0%	0%	0%
0%	0%	0%	0%	-	0%	0%	0%
0%	0%	0%	0%	-	0%	0%	0%
100%	0%	0%	0%	-	0%	0%	0%
0%	0%	0%	0%	-	0%	0%	0%
0%	0%	0%	0%	-	0%	0%	0%
0%	0%	0%	0%	-	0%	0%	0%
0%	0%	0%	0%	-	0%	0%	0%
0%	100%	0%	15%	2,40	0%	100%	0%

0%	0%	0%	0%	-	0%	0%	0%
0%	0%	0%	0%	-	0%	0%	0%
0%	0%	0%	0%	-	0%	0%	0%
0%	0%	0%	0%	-	0%	0%	0%
0%	0%	0%	0%	-	0%	0%	0%
0%	0%	0%	0%	-	0%	0%	0%
0%	0%	0%	0%	-	0%	0%	0%
0%	0%	0%	0%	-	0%	0%	0%
0%	0%	0%	0%	-	0%	0%	0%
0%	0%	0%	0%	-	0%	0%	0%
0%	0%	0%	0%	-	0%	0%	0%
0%	0%	0%	0%	-	0%	0%	0%
0%	0%	0%	0%	-	0%	0%	0%
0%	0%	0%	0%	-	0%	0%	0%
0%	0%	0%	0%	-	0%	0%	0%
0%	0%	0%	0%	-	0%	0%	0%
0%	0%	0%	0%	-	0%	0%	0%
0%	0%	0%	0%	-	0%	0%	0%
0%	0%	0%	0%	-	0%	0%	0%
0%	0%	0%	0%	-	0%	0%	0%
0%	0%	0%	0%	-	0%	0%	0%
30%	70%	0%	0%	-	0%	0%	0%
0%	0%	0%	0%	-	0%	0%	0%
0%	0%	0%	0%	-	0%	0%	0%
0%	0%	0%	0%	-	0%	0%	0%
0%	0%	0%	0%	-	0%	0%	0%
0%	100%	0%	0%	-	0%	0%	0%
0%	0%	0%	0%	-	0%	0%	0%
0%	0%	0%	0%	-	0%	0%	0%
0%	0%	0%	0%	-	0%	0%	0%
0%	0%	0%	0%	-	0%	0%	0%
0%	0%	0%	0%	-	0%	0%	0%
0%	0%	0%	0%	-	0%	0%	0%
0%	0%	0%	0%	-	0%	0%	0%
0%	0%	0%	0%	-	0%	0%	0%
0%	0%	0%	0%	-	0%	0%	0%
0%	0%	0%	0%	-	0%	0%	0%
0%	0%	0%	0%	-	0%	0%	0%
0%	0%	0%	0%	-	0%	0%	0%
0%	0%	0%	0%	-	0%	0%	0%
0%	0%	0%	0%	-	0%	0%	0%
100%	0%	0%	0%	-	0%	0%	0%
0%	0%	0%	0%	-	0%	0%	0%
0%	0%	0%	0%	-	0%	0%	0%
0%	0%	0%	0%	-	0%	0%	0%
0%	0%	0%	0%	-	0%	0%	0%
0%	0%	0%	0%	-	0%	0%	0%
0%	0%	0%	0%	-	0%	0%	0%
0%	0%	0%	0%	-	0%	0%	0%
0%	0%	0%	0%	-	0%	0%	0%
0%	0%	0%	0%	-	0%	0%	0%
0%	0%	0%	0%	-	0%	0%	0%
0%	0%	0%	0%	-	0%	0%	0%
0%	0%	0%	0%	-	0%	0%	0%
0%	0%	0%	0%	-	0%	0%	0%
0%	0%	0%	0%	-	0%	0%	0%
0%	0%	0%	0%	-	0%	0%	0%
100%	0%	0%	0%	-	0%	0%	0%
0%	0%	0%	0%	-	0%	0%	0%
0%	0%	0%	0%	-	0%	0%	0%
0%	0%	0%	0%	-	0%	0%	0%
0%	0%	0%	0%	-	0%	0%	0%
0%	0%	0%	0%	-	0%	0%	0%
0%	0%	0%	0%	-	0%	0%	0%
0%	0%	0%	0%	-	0%	0%	0%
0%	0%	0%	0%	-	0%	0%	0%

0%	0%	0%	0%	-	0%	0%	0%
0%	0%	0%	0%	-	0%	0%	0%
0%	0%	0%	0%	-	0%	0%	0%
0%	0%	0%	0%	-	0%	0%	0%
0%	0%	0%	0%	-	0%	0%	0%
0%	0%	0%	0%	-	0%	0%	0%
0%	0%	0%	0%	-	0%	0%	0%
0%	0%	0%	0%	-	0%	0%	0%
0%	0%	0%	0%	-	0%	0%	0%
100%	0%	0%	0%	-	0%	0%	0%
0%	0%	0%	0%	-	0%	0%	0%
0%	0%	0%	0%	-	0%	0%	0%
0%	0%	0%	0%	-	0%	0%	0%
0%	0%	0%	0%	-	0%	0%	0%
0%	0%	0%	0%	-	0%	0%	0%
0%	0%	0%	0%	-	0%	0%	0%
0%	0%	0%	0%	-	0%	0%	0%
0%	0%	0%	0%	-	0%	0%	0%
0%	0%	0%	0%	-	0%	0%	0%
0%	0%	0%	0%	-	0%	0%	0%
0%	0%	0%	0%	-	0%	0%	0%
0%	0%	0%	0%	-	0%	0%	0%
0%	0%	0%	0%	-	0%	0%	0%
0%	0%	0%	0%	-	0%	0%	0%
0%	0%	0%	0%	-	0%	0%	0%
0%	0%	0%	0%	-	0%	0%	0%
0%	0%	0%	0%	-	0%	0%	0%
0%	0%	0%	0%	-	0%	0%	0%
0%	0%	0%	0%	-	0%	0%	0%
0%	0%	0%	0%	-	0%	0%	0%
0%	0%	0%	0%	-	0%	0%	0%
0%	0%	0%	0%	-	0%	0%	0%
0%	0%	0%	0%	-	0%	0%	0%
0%	0%	0%	0%	-	0%	0%	0%
0%	0%	0%	0%	-	0%	0%	0%
0%	0%	0%	0%	-	0%	0%	0%
0%	0%	0%	0%	-	0%	0%	0%
0%	0%	0%	0%	-	0%	0%	0%
0%	0%	0%	0%	-	0%	0%	0%
0%	0%	0%	0%	-	0%	0%	0%
0%	0%	0%	0%	-	0%	0%	0%
0%	0%	0%	0%	-	0%	0%	0%
0%	0%	0%	0%	-	0%	0%	0%
0%	0%	0%	0%	-	0%	0%	0%
0%	0%	0%	0%	-	0%	0%	0%
0%	0%	0%	0%	-	0%	0%	0%
0%	0%	0%	0%	-	0%	0%	0%
0%	0%	0%	0%	-	0%	0%	0%
0%	0%	0%	0%	-	0%	0%	0%
100%	0%	0%	0%	-	0%	0%	0%
0%	0%	0%	0%	-	0%	0%	0%
0%	0%	0%	0%	-	0%	0%	0%
0%	0%	0%	0%	-	0%	0%	0%
0%	0%	0%	0%	-	0%	0%	0%
0%	0%	0%	0%	-	0%	0%	0%
0%	0%	0%	0%	-	0%	0%	0%
0%	0%	0%	0%	-	0%	0%	0%
0%	0%	0%	100%	4,40	0%	100%	0%
0%	0%	0%	0%	-	0%	0%	0%
0%	0%	0%	5%	1,29	100%	0%	0%
0%	0%	0%	0%	-	0%	0%	0%
0%	0%	0%	0%	-	0%	0%	0%
0%	0%	0%	0%	-	0%	0%	0%
0%	0%	0%	0%	-	0%	0%	0%
0%	0%	0%	0%	-	0%	0%	0%
0%	0%	0%	0%	-	0%	0%	0%

0%	0%	0%	0%	-	0%	0%	0%
0%	0%	0%	0%	-	0%	0%	0%
0%	0%	0%	0%	-	0%	0%	0%
0%	0%	0%	0%	-	0%	0%	0%
0%	0%	0%	0%	-	0%	0%	0%
0%	0%	0%	0%	-	0%	0%	0%
0%	0%	0%	0%	-	0%	0%	0%
0%	0%	0%	0%	-	0%	0%	0%
0%	0%	0%	0%	-	0%	0%	0%
0%	0%	0%	0%	-	0%	0%	0%
0%	0%	0%	0%	-	0%	0%	0%
0%	0%	0%	0%	-	0%	0%	0%
0%	0%	0%	0%	-	0%	0%	0%
0%	0%	0%	0%	-	0%	0%	0%
0%	0%	0%	0%	-	0%	0%	0%
100%	0%	0%	0%	-	0%	0%	0%
0%	0%	0%	0%	-	0%	0%	0%
0%	0%	0%	0%	-	0%	0%	0%
0%	0%	0%	0%	-	0%	0%	0%
0%	0%	0%	0%	-	0%	0%	0%
0%	0%	0%	0%	-	0%	0%	0%
0%	100%	0%	0%	-	0%	0%	0%
0%	0%	0%	0%	-	0%	0%	0%
0%	0%	0%	0%	-	0%	0%	0%
0%	0%	0%	0%	-	0%	0%	0%
0%	0%	0%	0%	-	0%	0%	0%
0%	0%	0%	0%	-	0%	0%	0%
0%	0%	0%	0%	-	0%	0%	0%
0%	0%	0%	0%	-	0%	0%	0%
0%	0%	0%	0%	-	0%	0%	0%
0%	0%	0%	0%	-	0%	0%	0%
0%	0%	0%	0%	-	0%	0%	0%
0%	0%	0%	0%	-	0%	0%	0%
0%	0%	0%	0%	-	0%	0%	0%
0%	0%	0%	0%	-	0%	0%	0%
0%	0%	0%	0%	-	0%	0%	0%
0%	0%	0%	0%	-	0%	0%	0%
0%	0%	0%	0%	-	0%	0%	0%
0%	0%	0%	0%	-	0%	0%	0%
0%	0%	0%	0%	-	0%	0%	0%
0%	0%	0%	0%	-	0%	0%	0%
0%	0%	0%	0%	-	0%	0%	0%
0%	0%	0%	0%	-	0%	0%	0%
0%	0%	0%	0%	-	0%	0%	0%
0%	0%	0%	0%	-	0%	0%	0%
0%	0%	0%	0%	-	0%	0%	0%
0%	0%	0%	0%	-	0%	0%	0%
0%	0%	0%	0%	-	0%	0%	0%
0%	0%	0%	0%	-	0%	0%	0%
0%	0%	0%	0%	-	0%	0%	0%
0%	0%	0%	0%	-	0%	0%	0%
0%	0%	0%	0%	-	0%	0%	0%
0%	0%	0%	0%	-	0%	0%	0%
0%	0%	0%	0%	-	0%	0%	0%
0%	0%	0%	0%	-	0%	0%	0%
0%	0%	0%	0%	-	0%	0%	0%
0%	0%	0%	0%	-	0%	0%	0%
0%	0%	0%	0%	-	0%	0%	0%
0%	0%	0%	0%	-	0%	0%	0%
0%	0%	0%	0%	-	0%	0%	0%
0%	0%	0%	0%	-	0%	0%	0%
0%	0%	0%	0%	-	0%	0%	0%
0%	0%	0%	0%	-	0%	0%	0%
0%	0%	0%	0%	-	0%	0%	0%
0%	0%	0%	0%	-	0%	0%	0%
0%	0%	0%	0%	-	0%	0%	0%

0%	0%	0%	0%	-	0%	0%	0%
0%	0%	0%	0%	-	0%	0%	0%
0%	0%	0%	0%	-	0%	0%	0%
0%	0%	0%	0%	-	0%	0%	0%
0%	0%	0%	0%	-	0%	0%	0%
0%	0%	0%	0%	-	0%	0%	0%
0%	0%	0%	0%	-	0%	0%	0%
0%	0%	0%	0%	-	0%	0%	0%
0%	0%	0%	0%	-	0%	0%	0%
0%	0%	0%	0%	-	0%	0%	0%
0%	0%	0%	0%	-	0%	0%	0%
0%	0%	0%	0%	-	0%	0%	0%
0%	0%	0%	0%	-	0%	0%	0%
0%	0%	0%	0%	-	0%	0%	0%
0%	0%	0%	0%	-	0%	0%	0%
0%	0%	0%	0%	-	0%	0%	0%
0%	0%	0%	0%	-	0%	0%	0%
0%	0%	0%	0%	-	0%	0%	0%
0%	0%	0%	0%	-	0%	0%	0%
0%	0%	0%	0%	-	0%	0%	0%
0%	0%	0%	0%	-	0%	0%	0%
0%	0%	0%	40%	-	0%	100%	0%
0%	0%	0%	0%	-	0%	0%	0%
0%	0%	0%	0%	-	0%	0%	0%
0%	0%	0%	0%	-	0%	0%	0%
0%	0%	0%	0%	-	0%	0%	0%
0%	0%	0%	0%	-	0%	0%	0%
0%	0%	0%	0%	-	0%	0%	0%
0%	0%	0%	0%	-	0%	0%	0%
0%	0%	0%	0%	-	0%	0%	0%
0%	0%	0%	0%	-	0%	0%	0%
0%	0%	0%	0%	-	0%	0%	0%
0%	0%	0%	0%	-	0%	0%	0%
0%	0%	0%	0%	-	0%	0%	0%
0%	0%	0%	0%	-	0%	0%	0%
0%	0%	0%	0%	-	0%	0%	0%
0%	0%	0%	0%	-	0%	0%	0%
0%	0%	0%	0%	-	0%	0%	0%
0%	0%	0%	0%	-	0%	0%	0%
0%	0%	0%	0%	-	0%	0%	0%
0%	0%	0%	0%	-	0%	0%	0%
0%	0%	0%	0%	-	0%	0%	0%
0%	0%	0%	0%	-	0%	0%	0%
0%	0%	0%	0%	-	0%	0%	0%
0%	0%	0%	0%	-	0%	0%	0%
0%	0%	0%	0%	-	0%	0%	0%
0%	0%	0%	0%	-	0%	0%	0%
0%	0%	0%	0%	-	0%	0%	0%
0%	0%	0%	0%	-	0%	0%	0%
0%	0%	0%	0%	-	0%	0%	0%
0%	0%	0%	0%	-	0%	0%	0%
0%	0%	0%	0%	-	0%	0%	0%
0%	0%	0%	0%	-	0%	0%	0%
0%	0%	0%	0%	-	0%	0%	0%
0%	0%	0%	0%	-	0%	0%	0%
0%	0%	0%	0%	-	0%	0%	0%
0%	0%	0%	0%	-	0%	0%	0%
0%	0%	0%	0%	-	0%	0%	0%
0%	0%	0%	0%	-	0%	0%	0%
0%	0%	0%	0%	-	0%	0%	0%
0%	0%	0%	20%	-	0%	100%	0%
0%	0%	0%	0%	-	0%	0%	0%
0%	0%	0%	0%	-	0%	0%	0%
0%	0%	0%	0%	-	0%	0%	0%
0%	0%	0%	0%	-	0%	0%	0%
0%	0%	0%	0%	-	0%	0%	0%

0%	0%	0%	0%	-	0%	0%	0%
0%	0%	0%	0%	-	0%	0%	0%
0%	0%	0%	0%	-	0%	0%	0%
0%	0%	0%	0%	-	0%	0%	0%
0%	0%	0%	0%	-	0%	0%	0%
0%	0%	0%	0%	-	0%	0%	0%
0%	0%	0%	0%	-	0%	0%	0%
0%	0%	0%	0%	-	0%	0%	0%
0%	0%	0%	0%	-	0%	0%	0%
0%	0%	0%	0%	-	0%	0%	0%
0%	0%	0%	0%	-	0%	0%	0%
0%	0%	0%	0%	-	0%	0%	0%
0%	0%	0%	0%	-	0%	0%	0%
0%	0%	0%	0%	-	0%	0%	0%
0%	0%	0%	0%	-	0%	0%	0%
0%	0%	0%	0%	-	0%	0%	0%
0%	0%	0%	0%	-	0%	0%	0%
0%	0%	0%	0%	-	0%	0%	0%
0%	0%	0%	0%	-	0%	0%	0%
0%	0%	0%	0%	-	0%	0%	0%
0%	0%	0%	0%	-	0%	0%	0%
0%	0%	0%	0%	-	0%	0%	0%
0%	0%	0%	0%	-	0%	0%	0%
0%	0%	0%	0%	-	0%	0%	0%
0%	0%	0%	0%	-	0%	0%	0%
0%	0%	0%	0%	-	0%	0%	0%
0%	0%	0%	0%	-	0%	0%	0%
0%	0%	0%	0%	-	0%	0%	0%
0%	0%	0%	0%	-	0%	0%	0%
0%	0%	0%	0%	-	0%	0%	0%
0%	0%	0%	0%	-	0%	0%	0%
0%	0%	0%	0%	-	0%	0%	0%
0%	0%	0%	0%	-	0%	0%	0%
0%	0%	0%	0%	-	0%	0%	0%
0%	0%	0%	0%	-	0%	0%	0%
0%	0%	0%	0%	-	0%	0%	0%
0%	0%	0%	0%	-	0%	0%	0%
0%	0%	0%	0%	-	0%	0%	0%
0%	0%	0%	0%	-	0%	0%	0%
0%	0%	0%	0%	-	0%	0%	0%
0%	0%	0%	0%	-	0%	0%	0%
0%	0%	0%	0%	-	0%	0%	0%
0%	0%	0%	0%	-	0%	0%	0%
0%	0%	0%	0%	-	0%	0%	0%
0%	0%	0%	0%	-	0%	0%	0%
0%	0%	0%	0%	-	0%	0%	0%
0%	0%	0%	0%	-	0%	0%	0%
0%	0%	0%	0%	-	0%	0%	0%
0%	0%	0%	0%	-	0%	0%	0%
0%	0%	0%	0%	-	0%	0%	0%
0%	0%	0%	0%	-	0%	0%	0%
0%	0%	0%	0%	-	0%	0%	0%
0%	0%	0%	0%	-	0%	0%	0%
0%	0%	0%	0%	-	0%	0%	0%
0%	0%	0%	0%	-	0%	0%	0%
0%	0%	0%	0%	-	0%	0%	0%
0%	0%	0%	0%	-	0%	0%	0%
0%	0%	0%	0%	-	0%	0%	0%
0%	0%	0%	0%	-	0%	0%	0%
0%	0%	0%	0%	-	0%	0%	0%
0%	0%	0%	0%	-	0%	0%	0%
0%	0%	0%	0%	-	0%	0%	0%
0%	0%	0%	0%	-	0%	0%	0%
0%	0%	0%	0%	-	0%	0%	0%
0%	0%	0%	0%	-	0%	0%	0%
0%	0%	0%	0%	-	0%	0%	0%

0%	0%	0%	0%	-	0%	0%	0%
0%	0%	0%	0%	-	0%	0%	0%
0%	0%	0%	0%	-	0%	0%	0%
0%	0%	0%	0%	-	0%	0%	0%
0%	0%	0%	0%	-	0%	0%	0%
0%	0%	0%	0%	-	0%	0%	0%
0%	0%	0%	0%	-	0%	0%	0%
0%	0%	0%	0%	-	0%	0%	0%
0%	0%	0%	0%	-	0%	0%	0%
0%	0%	0%	0%	-	0%	0%	0%
0%	0%	0%	0%	-	0%	0%	0%
0%	0%	0%	0%	-	0%	0%	0%
0%	0%	0%	0%	-	0%	0%	0%
0%	0%	0%	0%	-	0%	0%	0%
0%	0%	0%	0%	-	0%	0%	0%
0%	0%	0%	0%	-	0%	0%	0%
0%	0%	0%	0%	-	0%	0%	0%
100%	0%	0%	0%	-	0%	0%	0%
0%	0%	0%	24%	5,04	100%	0%	0%
100%	0%	0%	0%	-	0%	0%	0%
0%	0%	0%	0%	-	0%	0%	0%
0%	0%	0%	0%	-	0%	0%	0%
0%	0%	0%	0%	-	0%	0%	0%
0%	0%	0%	0%	-	0%	0%	0%
0%	0%	0%	0%	-	0%	0%	0%
100%	0%	0%	0%	-	0%	0%	0%
0%	0%	0%	0%	-	0%	0%	0%
0%	0%	0%	0%	-	0%	0%	0%
0%	0%	0%	0%	-	0%	0%	0%
100%	0%	0%	0%	-	0%	0%	0%
0%	0%	0%	0%	-	0%	0%	0%
0%	0%	0%	0%	-	0%	0%	0%
0%	0%	0%	0%	-	0%	0%	0%
0%	0%	0%	0%	-	0%	0%	0%
0%	0%	0%	0%	-	0%	0%	0%
0%	0%	0%	0%	-	0%	0%	0%
0%	0%	0%	0%	-	0%	0%	0%
0%	0%	0%	0%	-	0%	0%	0%
0%	0%	0%	0%	-	0%	0%	0%
0%	0%	0%	0%	-	0%	0%	0%
0%	0%	0%	0%	-	0%	0%	0%
0%	0%	0%	0%	-	0%	0%	0%
0%	0%	0%	0%	-	0%	0%	0%
0%	0%	0%	0%	-	0%	0%	0%
0%	0%	0%	0%	-	0%	0%	0%
0%	0%	0%	0%	-	0%	0%	0%
0%	0%	0%	0%	-	0%	0%	0%
0%	0%	0%	0%	-	0%	0%	0%
0%	0%	0%	0%	-	0%	0%	0%
0%	0%	0%	0%	-	0%	0%	0%
0%	0%	0%	0%	-	0%	0%	0%
0%	0%	0%	0%	-	0%	0%	0%
0%	0%	0%	0%	-	0%	0%	0%
0%	0%	0%	0%	-	0%	0%	0%
0%	0%	0%	0%	-	0%	0%	0%
0%	0%	0%	0%	-	0%	0%	0%
0%	0%	0%	0%	-	0%	0%	0%
0%	0%	0%	0%	-	0%	0%	0%
0%	0%	0%	0%	-	0%	0%	0%
0%	0%	0%	0%	-	0%	0%	0%
0%	0%	0%	0%	-	0%	0%	0%
0%	0%	0%	0%	-	0%	0%	0%
0%	0%	0%	0%	-	0%	0%	0%
0%	0%	0%	0%	-	0%	0%	0%
0%	0%	0%	0%	-	0%	0%	0%
0%	0%	0%	0%	-	0%	0%	0%

0%	0%	0%	0%	-	0%	0%	0%
0%	0%	0%	0%	-	0%	0%	0%
0%	0%	0%	0%	-	0%	0%	0%
0%	0%	0%	0%	-	0%	0%	0%
0%	0%	0%	0%	-	0%	0%	0%
0%	0%	0%	0%	-	0%	0%	0%
0%	0%	0%	0%	-	0%	0%	0%
0%	0%	0%	0%	-	0%	0%	0%
0%	0%	0%	0%	-	0%	0%	0%
0%	0%	0%	0%	-	0%	0%	0%
0%	0%	0%	0%	-	0%	0%	0%
0%	0%	0%	0%	-	0%	0%	0%
0%	0%	0%	0%	-	0%	0%	0%
0%	0%	0%	0%	-	0%	0%	0%
0%	0%	0%	0%	-	0%	0%	0%
0%	0%	0%	0%	-	0%	0%	0%
0%	0%	0%	0%	-	0%	0%	0%
0%	0%	0%	0%	-	0%	0%	0%
0%	0%	0%	0%	-	0%	0%	0%
0%	0%	0%	0%	-	0%	0%	0%
0%	0%	0%	0%	-	0%	0%	0%
0%	0%	0%	0%	-	0%	0%	0%
0%	0%	0%	0%	-	0%	0%	0%
0%	0%	0%	0%	-	0%	0%	0%
0%	0%	0%	0%	-	0%	0%	0%
0%	0%	0%	0%	-	0%	0%	0%
0%	0%	0%	0%	-	0%	0%	0%
0%	0%	0%	0%	-	0%	0%	0%
0%	0%	0%	0%	-	0%	0%	0%
0%	0%	0%	0%	-	0%	0%	0%
0%	0%	0%	0%	-	0%	0%	0%
0%	0%	0%	0%	-	0%	0%	0%
0%	0%	0%	0%	-	0%	0%	0%
0%	0%	0%	0%	-	0%	0%	0%
0%	0%	0%	0%	-	0%	0%	0%
100%	0%	0%	10%	3,54	100%	0%	0%
100%	0%	0%	0%	-	0%	0%	0%
0%	0%	0%	0%	-	0%	0%	0%
0%	0%	0%	0%	-	0%	0%	0%
0%	0%	0%	0%	-	0%	0%	0%
0%	0%	0%	0%	-	0%	0%	0%
0%	0%	0%	0%	-	0%	0%	0%
0%	0%	0%	0%	-	0%	0%	0%
0%	0%	0%	0%	-	0%	0%	0%
0%	0%	0%	0%	-	0%	0%	0%
0%	0%	0%	0%	-	0%	0%	0%
0%	0%	0%	0%	-	0%	0%	0%
0%	0%	0%	0%	-	0%	0%	0%
0%	0%	0%	0%	-	0%	0%	0%
0%	0%	0%	0%	-	0%	0%	0%
0%	0%	0%	0%	-	0%	0%	0%
0%	0%	0%	0%	-	0%	0%	0%
0%	0%	0%	0%	-	0%	0%	0%
0%	0%	0%	0%	-	0%	0%	0%
0%	0%	0%	0%	-	0%	0%	0%
0%	0%	0%	0%	-	0%	0%	0%
0%	0%	0%	0%	-	0%	0%	0%
0%	0%	0%	0%	-	0%	0%	0%
0%	0%	0%	0%	-	0%	0%	0%
0%	0%	0%	0%	-	0%	0%	0%
0%	0%	0%	0%	-	0%	0%	0%
0%	0%	0%	0%	-	0%	0%	0%
0%	0%	0%	0%	-	0%	0%	0%
0%	0%	0%	0%	-	0%	0%	0%
0%	0%	0%	0%	-	0%	0%	0%
0%	0%	0%	0%	-	0%	0%	0%

0%	0%	0%	0%	-	0%	0%	0%
0%	0%	0%	0%	-	0%	0%	0%
0%	0%	0%	0%	-	0%	0%	0%
0%	0%	0%	0%	-	0%	0%	0%
0%	0%	0%	0%	-	0%	0%	0%
0%	0%	0%	0%	-	0%	0%	0%
0%	0%	0%	0%	-	0%	0%	0%
0%	0%	0%	0%	-	0%	0%	0%
0%	0%	0%	0%	-	0%	0%	0%
0%	0%	0%	0%	-	0%	0%	0%
0%	0%	0%	0%	-	0%	0%	0%
0%	0%	0%	0%	-	0%	0%	0%
0%	0%	0%	0%	-	0%	0%	0%
0%	0%	0%	0%	-	0%	0%	0%
0%	0%	0%	0%	-	0%	0%	0%
0%	0%	0%	0%	-	0%	0%	0%
0%	0%	0%	0%	-	0%	0%	0%
0%	0%	0%	0%	-	0%	0%	0%
0%	0%	0%	0%	-	0%	0%	0%
0%	0%	0%	0%	-	0%	0%	0%
0%	0%	0%	0%	-	0%	0%	0%
0%	0%	0%	0%	-	0%	0%	0%
0%	0%	0%	0%	-	0%	0%	0%
0%	0%	0%	0%	-	0%	0%	0%
0%	0%	0%	0%	-	0%	0%	0%
0%	0%	0%	0%	-	0%	0%	0%
0%	0%	0%	0%	-	0%	0%	0%
0%	0%	0%	0%	-	0%	0%	0%
0%	0%	0%	0%	-	0%	0%	0%
0%	0%	0%	0%	-	0%	0%	0%
0%	0%	0%	0%	-	0%	0%	0%
0%	0%	0%	0%	-	0%	0%	0%
0%	0%	0%	0%	-	0%	0%	0%
0%	0%	0%	0%	-	0%	0%	0%
0%	0%	0%	0%	-	0%	0%	0%
0%	0%	0%	0%	-	0%	0%	0%
0%	0%	0%	0%	-	0%	0%	0%
0%	0%	0%	0%	-	0%	0%	0%
0%	0%	0%	0%	-	0%	0%	0%
0%	0%	0%	0%	-	0%	0%	0%
0%	0%	0%	0%	-	0%	0%	0%
0%	0%	0%	0%	-	0%	0%	0%
0%	0%	0%	0%	-	0%	0%	0%
0%	0%	0%	0%	-	0%	0%	0%
0%	0%	0%	0%	-	0%	0%	0%
0%	0%	0%	0%	-	0%	0%	0%
0%	0%	0%	0%	-	0%	0%	0%
0%	0%	0%	0%	-	0%	0%	0%
0%	0%	0%	0%	-	0%	0%	0%
0%	0%	0%	0%	-	0%	0%	0%
0%	0%	0%	0%	-	0%	0%	0%
0%	0%	0%	0%	-	0%	0%	0%
0%	0%	0%	0%	-	0%	0%	0%
0%	0%	0%	0%	-	0%	0%	0%
0%	0%	0%	0%	-	0%	0%	0%
0%	0%	0%	15%	13,20	100%	0%	0%
0%	0%	0%	0%	-	0%	0%	0%
0%	0%	0%	0%	-	0%	0%	0%
0%	0%	0%	0%	-	0%	0%	0%
0%	0%	0%	0%	-	0%	0%	0%
0%	0%	0%	0%	-	0%	0%	0%
0%	0%	0%	0%	-	0%	0%	0%
0%	0%	0%	0%	-	0%	0%	0%
0%	0%	0%	0%	-	0%	0%	0%
0%	0%	0%	0%	-	0%	0%	0%
0%	0%	0%	0%	-	0%	0%	0%

0%	0%	0%	0%	-	0%	0%	0%
0%	0%	0%	0%	-	0%	0%	0%
0%	0%	0%	0%	-	0%	0%	0%
0%	0%	0%	0%	-	0%	0%	0%
0%	0%	0%	0%	-	0%	0%	0%
0%	0%	0%	0%	-	0%	0%	0%
0%	0%	0%	5%	2,25	0%	100%	0%
0%	0%	0%	0%	-	0%	0%	0%
0%	0%	0%	0%	-	0%	0%	0%
0%	0%	0%	0%	-	0%	0%	0%
0%	0%	0%	0%	-	0%	0%	0%
0%	0%	0%	0%	-	0%	0%	0%
0%	0%	0%	0%	-	0%	0%	0%
0%	0%	0%	0%	-	0%	0%	0%
0%	0%	0%	0%	-	0%	0%	0%
0%	0%	0%	0%	-	0%	0%	0%
0%	0%	0%	0%	-	0%	0%	0%
0%	0%	0%	0%	-	0%	0%	0%
0%	0%	0%	0%	-	0%	0%	0%
0%	0%	0%	0%	-	0%	0%	0%
0%	0%	0%	0%	-	0%	0%	0%
0%	0%	0%	0%	-	0%	0%	0%
0%	0%	0%	0%	-	0%	0%	0%
0%	0%	0%	0%	-	0%	0%	0%
0%	0%	0%	0%	-	0%	0%	0%
0%	0%	0%	0%	-	0%	0%	0%
0%	0%	0%	0%	-	0%	0%	0%
0%	0%	0%	0%	-	0%	0%	0%
0%	0%	0%	0%	-	0%	0%	0%
0%	0%	0%	0%	-	0%	0%	0%
0%	0%	0%	0%	-	0%	0%	0%
0%	0%	0%	0%	-	0%	0%	0%
0%	0%	0%	0%	-	0%	0%	0%
0%	0%	0%	0%	-	0%	0%	0%
0%	0%	0%	0%	-	0%	0%	0%
0%	0%	0%	0%	-	0%	0%	0%
0%	0%	0%	0%	-	0%	0%	0%
0%	0%	0%	0%	-	0%	0%	0%
0%	0%	0%	0%	-	0%	0%	0%
0%	0%	0%	0%	-	0%	0%	0%
0%	0%	0%	0%	-	0%	0%	0%
0%	0%	0%	0%	-	0%	0%	0%
0%	0%	0%	0%	-	0%	0%	0%
0%	0%	0%	0%	-	0%	0%	0%
0%	0%	0%	0%	-	0%	0%	0%
0%	0%	0%	0%	-	0%	0%	0%
0%	0%	0%	0%	-	0%	0%	0%
0%	0%	0%	0%	-	0%	0%	0%
0%	0%	0%	0%	-	0%	0%	0%
0%	0%	0%	65%	-	100%	0%	0%
0%	0%	0%	0%	-	0%	0%	0%
0%	0%	0%	0%	-	0%	0%	0%
0%	0%	0%	0%	-	0%	0%	0%
0%	0%	0%	0%	-	0%	0%	0%
0%	0%	0%	0%	-	0%	0%	0%
0%	0%	0%	0%	-	0%	0%	0%
0%	0%	0%	0%	-	0%	0%	0%
0%	0%	0%	0%	-	0%	0%	0%
0%	0%	0%	0%	-	0%	0%	0%
0%	0%	0%	0%	-	0%	0%	0%
0%	0%	0%	0%	-	0%	0%	0%
0%	0%	0%	0%	-	0%	0%	0%
0%	0%	0%	0%	-	0%	0%	0%
0%	0%	0%	0%	-	0%	0%	0%
0%	0%	0%	0%	-	0%	0%	0%
0%	0%	0%	0%	-	0%	0%	0%

0%	0%	0%	0%	-	0%	0%	0%
0%	0%	0%	0%	-	0%	0%	0%
0%	0%	0%	0%	-	0%	0%	0%
0%	0%	0%	0%	-	0%	0%	0%
0%	0%	0%	0%	-	0%	0%	0%
0%	0%	0%	0%	-	0%	0%	0%
0%	0%	0%	0%	-	0%	0%	0%
0%	0%	0%	0%	-	0%	0%	0%
0%	0%	0%	0%	-	0%	0%	0%
0%	0%	0%	0%	-	0%	0%	0%
0%	0%	0%	0%	-	0%	0%	0%
0%	0%	0%	0%	-	0%	0%	0%
0%	0%	0%	0%	-	0%	0%	0%
0%	0%	0%	0%	-	0%	0%	0%
0%	0%	0%	0%	-	0%	0%	0%
0%	0%	0%	0%	-	0%	0%	0%
0%	0%	0%	0%	-	0%	0%	0%
0%	0%	0%	0%	-	0%	0%	0%
0%	0%	0%	0%	-	0%	0%	0%
0%	0%	0%	0%	-	0%	0%	0%
0%	0%	0%	0%	-	0%	0%	0%
0%	0%	0%	0%	-	0%	0%	0%
0%	0%	0%	0%	-	0%	0%	0%
0%	0%	0%	0%	-	0%	0%	0%
0%	0%	0%	0%	-	0%	0%	0%
0%	0%	0%	0%	-	0%	0%	0%
0%	0%	0%	0%	-	0%	0%	0%
0%	0%	0%	0%	-	0%	0%	0%
0%	0%	0%	0%	-	0%	0%	0%
0%	0%	0%	0%	-	0%	0%	0%
0%	0%	0%	0%	-	0%	0%	0%
0%	0%	0%	0%	-	0%	0%	0%
0%	0%	0%	0%	-	0%	0%	0%
0%	0%	0%	0%	-	0%	0%	0%
0%	0%	0%	0%	-	0%	0%	0%
0%	0%	0%	0%	-	0%	0%	0%
0%	0%	0%	0%	-	0%	0%	0%
0%	0%	0%	0%	-	0%	0%	0%
0%	0%	0%	0%	-	0%	0%	0%
0%	0%	0%	0%	-	0%	0%	0%
0%	0%	0%	0%	-	0%	0%	0%
100%	0%	0%	0%	-	0%	0%	0%
0%	0%	0%	0%	-	0%	0%	0%
0%	0%	0%	0%	-	0%	0%	0%
0%	0%	0%	0%	-	0%	0%	0%
0%	0%	0%	0%	-	0%	0%	0%
0%	0%	0%	0%	-	0%	0%	0%
0%	0%	0%	0%	-	0%	0%	0%
0%	0%	0%	0%	-	0%	0%	0%
0%	0%	0%	0%	-	0%	0%	0%
0%	0%	0%	0%	-	0%	0%	0%
0%	0%	0%	0%	-	0%	0%	0%
0%	0%	0%	0%	-	0%	0%	0%
0%	0%	0%	0%	-	0%	0%	0%
0%	0%	0%	0%	-	0%	0%	0%
0%	0%	0%	0%	-	0%	0%	0%
0%	0%	0%	0%	-	0%	0%	0%
0%	0%	0%	0%	-	0%	0%	0%
0%	0%	0%	0%	-	0%	0%	0%
0%	0%	0%	0%	-	0%	0%	0%
0%	0%	0%	0%	-	0%	0%	0%
0%	0%	0%	0%	-	0%	0%	0%
0%	0%	0%	0%	-	0%	0%	0%
0%	0%	0%	0%	-	0%	0%	0%
0%	0%	0%	0%	-	0%	0%	0%
0%	0%	0%	0%	-	0%	0%	0%

0%	0%	0%	0%	-	0%	0%	0%
0%	0%	0%	0%	-	0%	0%	0%
0%	0%	0%	0%	-	0%	0%	0%
0%	0%	0%	0%	-	0%	0%	0%
0%	0%	0%	0%	-	0%	0%	0%
0%	0%	0%	0%	-	0%	0%	0%
0%	0%	0%	0%	-	0%	0%	0%
0%	0%	0%	0%	-	0%	0%	0%
0%	0%	0%	0%	-	0%	0%	0%
0%	0%	0%	0%	-	0%	0%	0%
0%	0%	0%	0%	-	0%	0%	0%
0%	0%	0%	0%	-	0%	0%	0%
0%	0%	0%	0%	-	0%	0%	0%
0%	0%	0%	0%	-	0%	0%	0%
0%	0%	0%	0%	-	0%	0%	0%
0%	0%	0%	0%	-	0%	0%	0%
0%	0%	0%	0%	-	0%	0%	0%
0%	0%	0%	0%	-	0%	0%	0%
0%	0%	0%	0%	-	0%	0%	0%
0%	0%	0%	0%	-	0%	0%	0%
0%	0%	0%	0%	-	0%	0%	0%
0%	0%	0%	0%	-	0%	0%	0%
0%	0%	0%	0%	-	0%	0%	0%
0%	0%	0%	0%	-	0%	0%	0%
0%	0%	0%	0%	-	0%	0%	0%
0%	0%	0%	0%	-	0%	0%	0%
0%	0%	0%	0%	-	0%	0%	0%
0%	0%	0%	0%	-	0%	0%	0%
0%	0%	0%	0%	-	0%	0%	0%
0%	0%	0%	0%	-	0%	0%	0%
0%	0%	0%	0%	-	0%	0%	0%
0%	0%	0%	0%	-	0%	0%	0%
0%	0%	0%	0%	-	0%	0%	0%
0%	0%	0%	0%	-	0%	0%	0%
0%	0%	0%	0%	-	0%	0%	0%
0%	0%	0%	0%	-	0%	0%	0%
0%	0%	0%	0%	-	0%	0%	0%
0%	0%	0%	0%	-	0%	0%	0%
0%	0%	0%	0%	-	0%	0%	0%
0%	0%	0%	0%	-	0%	0%	0%
0%	0%	0%	0%	-	0%	0%	0%
0%	0%	0%	0%	-	0%	0%	0%
0%	0%	0%	0%	-	0%	0%	0%
0%	0%	0%	0%	-	0%	0%	0%
0%	0%	0%	0%	-	0%	0%	0%
0%	0%	0%	0%	-	0%	0%	0%
0%	0%	0%	0%	-	0%	0%	0%
0%	0%	0%	0%	-	0%	0%	0%
0%	0%	0%	0%	-	0%	0%	0%
0%	0%	0%	0%	-	0%	0%	0%
0%	0%	0%	0%	-	0%	0%	0%
0%	0%	0%	0%	-	0%	0%	0%
0%	0%	0%	0%	-	0%	0%	0%
0%	0%	0%	0%	-	0%	0%	0%
0%	0%	0%	0%	-	0%	0%	0%
0%	0%	0%	0%	-	0%	0%	0%
0%	0%	0%	0%	-	0%	0%	0%
0%	0%	0%	0%	-	0%	0%	0%
0%	0%	0%	0%	-	0%	0%	0%
0%	0%	0%	0%	-	0%	0%	0%
0%	0%	0%	0%	-	0%	0%	0%
0%	0%	0%	0%	-	0%	0%	0%
0%	0%	0%	0%	-	0%	0%	0%
0%	0%	0%	0%	-	0%	0%	0%
0%	0%	0%	0%	-	0%	0%	0%
0%	0%	0%	0%	-	0%	0%	0%

0%	0%	0%	0%	-	0%	0%	0%
0%	0%	0%	0%	-	0%	0%	0%
0%	0%	0%	0%	-	0%	0%	0%
0%	0%	0%	0%	-	0%	0%	0%
0%	0%	0%	0%	-	0%	0%	0%
0%	0%	0%	0%	-	0%	0%	0%
0%	0%	0%	0%	-	0%	0%	0%
0%	0%	0%	0%	-	0%	0%	0%
0%	0%	0%	0%	-	0%	0%	0%
0%	0%	0%	0%	-	0%	0%	0%
0%	0%	0%	0%	-	0%	0%	0%
0%	0%	0%	0%	-	0%	0%	0%
0%	0%	0%	0%	-	0%	0%	0%
0%	0%	0%	0%	-	0%	0%	0%
0%	0%	0%	0%	-	0%	0%	0%
0%	0%	0%	0%	-	0%	0%	0%
0%	0%	0%	0%	-	0%	0%	0%
0%	0%	0%	0%	-	0%	0%	0%
0%	0%	0%	0%	-	0%	0%	0%
0%	0%	0%	0%	-	0%	0%	0%
0%	0%	0%	0%	-	0%	0%	0%
0%	0%	0%	0%	-	0%	0%	0%
0%	0%	0%	0%	-	0%	0%	0%
0%	0%	0%	0%	-	0%	0%	0%
0%	0%	0%	0%	-	0%	0%	0%
0%	0%	0%	0%	-	0%	0%	0%
0%	0%	0%	0%	-	0%	0%	0%
0%	0%	0%	0%	-	0%	0%	0%
0%	0%	0%	0%	-	0%	0%	0%
0%	0%	0%	0%	-	0%	0%	0%
0%	0%	0%	0%	-	0%	0%	0%
0%	0%	0%	0%	-	0%	0%	0%
0%	0%	0%	0%	-	0%	0%	0%
0%	0%	0%	0%	-	0%	0%	0%
0%	0%	0%	0%	-	0%	0%	0%
0%	0%	0%	0%	-	0%	0%	0%
0%	0%	0%	0%	-	0%	0%	0%
0%	0%	0%	0%	-	0%	0%	0%
0%	0%	0%	0%	-	0%	0%	0%
0%	0%	0%	0%	-	0%	0%	0%
0%	0%	0%	0%	-	0%	0%	0%
0%	0%	0%	0%	-	0%	0%	0%
0%	0%	0%	0%	-	0%	0%	0%
0%	0%	0%	0%	-	0%	0%	0%
0%	0%	0%	0%	-	0%	0%	0%
0%	0%	0%	0%	-	0%	0%	0%
0%	0%	0%	0%	-	0%	0%	0%
0%	0%	0%	0%	-	0%	0%	0%
0%	0%	0%	0%	-	0%	0%	0%
0%	0%	0%	0%	-	0%	0%	0%
0%	0%	0%	0%	-	0%	0%	0%
0%	0%	0%	0%	-	0%	0%	0%
0%	0%	0%	0%	-	0%	0%	0%
0%	0%	0%	0%	-	0%	0%	0%
0%	0%	0%	0%	-	0%	0%	0%
0%	0%	0%	0%	-	0%	0%	0%
0%	0%	0%	0%	-	0%	0%	0%
0%	0%	0%	0%	-	0%	0%	0%
0%	0%	0%	0%	-	0%	0%	0%
0%	0%	0%	0%	-	0%	0%	0%
0%	0%	0%	0%	-	0%	0%	0%
0%	0%	0%	0%	-	0%	0%	0%
0%	0%	0%	0%	-	0%	0%	0%
0%	0%	0%	0%	-	0%	0%	0%
0%	0%	0%	0%	-	0%	0%	0%
0%	0%	0%	0%	-	0%	0%	0%

0%	*0%*	*0%*	0%	-	*0%*	*0%*	*0%*
0%	*0%*	*0%*	0%	-	*0%*	*0%*	*0%*
0%	*0%*	*0%*	0%	-	*0%*	*0%*	*0%*
0%	*0%*	*0%*	0%	-	*0%*	*0%*	*0%*
0%	*0%*	*0%*	0%	-	*0%*	*0%*	*0%*
0%	*0%*	*0%*	0%	-	*0%*	*0%*	*0%*
0%	*0%*	*0%*	0%	-	*0%*	*0%*	*0%*
0%	*0%*	*0%*	0%	-	*0%*	*0%*	*0%*
0%	*0%*	*0%*	0%	-	*0%*	*0%*	*0%*
0%	*0%*	*0%*	0%	-	*0%*	*0%*	*0%*
0%	*0%*	*0%*	0%	-	*0%*	*0%*	*0%*
0%	*0%*	*0%*	0%	-	*0%*	*0%*	*0%*
0%	*0%*	*0%*	0%	-	*0%*	*0%*	*0%*
0%	*0%*	*0%*	0%	-	*0%*	*0%*	*0%*
0%	*0%*	*0%*	0%	-	*0%*	*0%*	*0%*
0%	*0%*	*0%*	0%	-	*0%*	*0%*	*0%*
0%	*0%*	*0%*	0%	-	*0%*	*0%*	*0%*
0%	*0%*	*0%*	0%	-	*0%*	*0%*	*0%*
0%	*0%*	*0%*	0%	-	*0%*	*0%*	*0%*
0%	*0%*	*0%*	0%	-	*0%*	*0%*	*0%*
0%	*0%*	*0%*	0%	-	*0%*	*0%*	*0%*
0%	*0%*	*0%*	0%	-	*0%*	*0%*	*0%*
0%	*0%*	*0%*	0%	-	*0%*	*0%*	*0%*
0%	*0%*	*0%*	0%	-	*0%*	*0%*	*0%*
0%	*0%*	*0%*	0%	-	*0%*	*0%*	*0%*
0%	*0%*	*0%*	0%	-	*0%*	*0%*	*0%*
0%	*0%*	*0%*	0%	-	*0%*	*0%*	*0%*
0%	*0%*	*0%*	0%	-	*0%*	*0%*	*0%*
0%	*0%*	*0%*	0%	-	*0%*	*0%*	*0%*
0%	*0%*	*0%*	0%	-	*0%*	*0%*	*0%*
0%	*0%*	*0%*	0%	-	*0%*	*0%*	*0%*
0%	*0%*	*0%*	0%	-	*0%*	*0%*	*0%*
0%	*0%*	*0%*	0%	-	*0%*	*0%*	*0%*
0%	*0%*	*0%*	0%	-	*0%*	*0%*	*0%*
0%	*0%*	*0%*	0%	-	*0%*	*0%*	*0%*
0%	*0%*	*0%*	0%	-	*0%*	*0%*	*0%*
0%	*0%*	*0%*	0%	-	*0%*	*0%*	*0%*
0%	*0%*	*0%*	0%	-	*0%*	*0%*	*0%*
0%	*0%*	*0%*	0%	-	*0%*	*0%*	*0%*
0%	*0%*	*0%*	0%	-	*0%*	*0%*	*0%*
0%	*0%*	*0%*	0%	-	*0%*	*0%*	*0%*
0%	*0%*	*0%*	0%	-	*0%*	*0%*	*0%*
0%	*0%*	*0%*	0%	-	*0%*	*0%*	*0%*
0%	*0%*	*0%*	0%	-	*0%*	*0%*	*0%*
0%	*0%*	*0%*	0%	-	*0%*	*0%*	*0%*
0%	*0%*	*0%*	0%	-	*0%*	*0%*	*0%*
0%	*0%*	*0%*	0%	-	*0%*	*0%*	*0%*
0%	*0%*	*0%*	0%	-	*0%*	*0%*	*0%*
0%	*0%*	*0%*	0%	-	*0%*	*0%*	*0%*
0%	*0%*	*0%*	0%	-	*0%*	*0%*	*0%*
0%	*0%*	*0%*	0%	-	*0%*	*0%*	*0%*
0%	*0%*	*0%*	0%	-	*0%*	*0%*	*0%*
0%	*0%*	*0%*	0%	-	*0%*	*0%*	*0%*
0%	*0%*	*0%*	0%	-	*0%*	*0%*	*0%*
0%	*0%*	*0%*	8%	0,88	*0%*	*100%*	*0%*
0%	*0%*	*0%*	0%	-	*0%*	*0%*	*0%*
0%	*0%*	*0%*	0%	-	*0%*	*0%*	*0%*
0%	*0%*	*0%*	0%	-	*0%*	*0%*	*0%*
0%	*0%*	*0%*	0%	-	*0%*	*0%*	*0%*
0%	*0%*	*0%*	0%	-	*0%*	*0%*	*0%*
0%	*0%*	*0%*	0%	-	*0%*	*0%*	*0%*
0%	*0%*	*0%*	0%	-	*0%*	*0%*	*0%*
0%	*0%*	*0%*	0%	-	*0%*	*0%*	*0%*
0%	*0%*	*0%*	0%	-	*0%*	*0%*	*0%*
0%	*0%*	*0%*	0%	-	*0%*	*0%*	*0%*
0%	*0%*	*0%*	0%	-	*0%*	*0%*	*0%*

0%	0%	0%	0%	-	0%	0%	0%
0%	0%	0%	0%	-	0%	0%	0%
0%	0%	0%	0%	-	0%	0%	0%
0%	0%	0%	0%	-	0%	0%	0%
0%	0%	0%	0%	-	0%	0%	0%
0%	0%	0%	0%	-	0%	0%	0%
0%	0%	0%	0%	-	0%	0%	0%
0%	0%	0%	0%	-	0%	0%	0%
0%	0%	0%	0%	-	0%	0%	0%
0%	0%	0%	0%	-	0%	0%	0%
70%	30%	0%	0%	-	0%	0%	0%
0%	0%	0%	0%	-	0%	0%	0%
0%	0%	0%	0%	-	0%	0%	0%
0%	0%	0%	0%	-	0%	0%	0%
0%	0%	0%	0%	-	0%	0%	0%
0%	0%	0%	0%	-	0%	0%	0%
0%	0%	0%	0%	-	0%	0%	0%
0%	0%	0%	0%	-	0%	0%	0%
100%	0%	0%	0%	-	0%	0%	0%
0%	0%	0%	0%	-	0%	0%	0%
100%	0%	0%	0%	-	0%	0%	0%
0%	0%	0%	0%	-	0%	0%	0%
0%	0%	0%	0%	-	0%	0%	0%
0%	0%	0%	0%	-	0%	0%	0%
0%	0%	0%	0%	-	0%	0%	0%
0%	0%	0%	0%	-	0%	0%	0%

CCA % of interaction	Total length in interaction with CCA	% Algae win	% Neutral	% Coral wins	Valonia % of interaction	Total length in interaction with Valonia	% Algae win
0%	-	0%	0%	0%	0%	-	0%
0%	-	0%	0%	0%	0%	-	0%
0%	-	0%	0%	0%	0%	-	0%
0%	-	0%	0%	0%	0%	-	0%
0%	-	0%	0%	0%	0%	-	0%
0%	-	0%	0%	0%	0%	-	0%
0%	-	0%	0%	0%	0%	-	0%
0%	-	0%	0%	0%	0%	-	0%
0%	-	0%	0%	0%	0%	-	0%
0%	-	0%	0%	0%	0%	-	0%
0%	-	0%	0%	0%	0%	-	0%
0%	-	0%	0%	0%	0%	-	0%
0%	-	0%	0%	0%	0%	-	0%
0%	-	0%	0%	0%	0%	-	0%
0%	-	0%	0%	0%	0%	-	0%
0%	-	0%	0%	0%	0%	-	0%
0%	-	0%	0%	0%	0%	-	0%
0%	-	0%	0%	0%	0%	-	0%
40%	8,28	25%	75%	0%	0%	-	0%
0%	-	0%	0%	0%	0%	-	0%
0%	-	0%	0%	0%	0%	-	0%
0%	-	0%	0%	0%	0%	-	0%
0%	-	0%	0%	0%	0%	-	0%
0%	-	0%	0%	0%	0%	-	0%
0%	-	0%	0%	0%	0%	-	0%
30%	2,40	0%	0%	100%	0%	-	0%
0%	-	0%	0%	0%	0%	-	0%
0%	-	0%	0%	0%	0%	-	0%
0%	-	0%	0%	0%	0%	-	0%
0%	-	0%	0%	0%	0%	-	0%
0%	-	0%	0%	0%	0%	-	0%
20%	9,00	0%	0%	100%	0%	-	0%
0%	-	0%	0%	0%	0%	-	0%
0%	-	0%	0%	0%	0%	-	0%
15%	1,20	0%	30%	70%	0%	-	0%
0%	-	0%	0%	0%	0%	-	0%
50%	15,00	100%	0%	0%	0%	-	0%
0%	-	0%	0%	0%	0%	-	0%
0%	-	0%	0%	0%	0%	-	0%
0%	-	0%	0%	0%	0%	-	0%
0%	-	0%	0%	0%	0%	-	0%
0%	-	0%	0%	0%	0%	-	0%
0%	-	0%	0%	0%	0%	-	0%
0%	-	0%	0%	0%	0%	-	0%
10%	2,04	0%	40%	60%	0%	-	0%
0%	-	0%	0%	0%	0%	-	0%
0%	-	0%	0%	0%	0%	-	0%
0%	-	0%	0%	0%	0%	-	0%
0%	-	0%	0%	0%	0%	-	0%
0%	-	0%	0%	0%	0%	-	0%
0%	-	0%	0%	0%	0%	-	0%
0%	-	0%	0%	0%	0%	-	0%
0%	-	0%	0%	0%	0%	-	0%
0%	-	0%	0%	0%	0%	-	0%
0%	-	0%	0%	0%	0%	-	0%
0%	-	0%	0%	0%	0%	-	0%
0%	-	0%	0%	0%	0%	-	0%
0%	-	0%	0%	0%	0%	-	0%
0%	-	0%	0%	0%	0%	-	0%
0%	-	0%	0%	0%	0%	-	0%
0%	-	0%	0%	0%	0%	-	0%
5%	4,04	50%	50%	0%	0%	-	0%

0%	-	0%	0%	0%	0%	-	0%
0%	-	0%	0%	0%	0%	-	0%
0%	-	0%	0%	0%	0%	-	0%
0%	-	0%	0%	0%	0%	-	0%
0%	-	0%	0%	0%	0%	-	0%
0%	-	0%	0%	0%	0%	-	0%
10%	3,66	0%	100%	0%	0%	-	0%
0%	-	0%	0%	0%	0%	-	0%
0%	-	0%	0%	0%	0%	-	0%
0%	-	0%	0%	0%	0%	-	0%
15%	2,10	0%	100%	0%	0%	-	0%
0%	-	0%	0%	0%	0%	-	0%
0%	-	0%	0%	0%	0%	-	0%
0%	-	0%	0%	0%	0%	-	0%
0%	-	0%	0%	0%	0%	-	0%
0%	-	0%	0%	0%	0%	-	0%
0%	-	0%	0%	0%	0%	-	0%
0%	-	0%	0%	0%	0%	-	0%
0%	-	0%	0%	0%	0%	-	0%
0%	-	0%	0%	0%	0%	-	0%
10%	1,90	0%	100%	0%	0%	-	0%
0%	-	0%	0%	0%	0%	-	0%
0%	-	0%	0%	0%	0%	-	0%
0%	-	0%	0%	0%	0%	-	0%
10%	0,38	0%	100%	0%	0%	-	0%
40%	2,74	0%	100%	0%	0%	-	0%
0%	-	0%	0%	0%	0%	-	0%
100%	4,96	0%	100%	0%	0%	-	0%
15%	1,23	0%	100%	0%	0%	-	0%
0%	-	0%	0%	0%	0%	-	0%
0%	-	0%	0%	0%	100%	1,44	0%
0%	-	0%	0%	0%	0%	-	0%
0%	-	0%	0%	0%	0%	-	0%
15%	2,43	0%	100%	0%	0%	-	0%
0%	-	0%	0%	0%	0%	-	0%
0%	-	0%	0%	0%	0%	-	0%
0%	-	0%	0%	0%	0%	-	0%
0%	-	0%	0%	0%	0%	-	0%
0%	-	0%	0%	0%	0%	-	0%
0%	-	0%	0%	0%	0%	-	0%
0%	-	0%	0%	0%	0%	-	0%
0%	-	0%	0%	0%	0%	-	0%
0%	-	0%	0%	0%	0%	-	0%
0%	-	0%	0%	0%	0%	-	0%
0%	-	0%	0%	0%	0%	-	0%
0%	-	0%	0%	0%	0%	-	0%
0%	-	0%	0%	0%	0%	-	0%
0%	-	0%	0%	0%	0%	-	0%
0%	-	0%	0%	0%	0%	-	0%
0%	-	0%	0%	0%	0%	-	0%
0%	-	0%	0%	0%	0%	-	0%
0%	-	0%	0%	0%	0%	-	0%
0%	-	0%	0%	0%	0%	-	0%
0%	-	0%	0%	0%	0%	-	0%
0%	-	0%	0%	0%	0%	-	0%
0%	-	0%	0%	0%	0%	-	0%
0%	-	0%	0%	0%	0%	-	0%
0%	-	0%	0%	0%	0%	-	0%
0%	-	0%	0%	0%	0%	-	0%
0%	-	0%	0%	0%	0%	-	0%
0%	-	0%	0%	0%	0%	-	0%
0%	-	0%	0%	0%	0%	-	0%
0%	-	0%	0%	0%	0%	-	0%
40%	1,20	0%	100%	0%	0%	-	0%
100%	4,20	20%	80%	0%	0%	-	0%
0%	-	0%	0%	0%	0%	-	0%

0%	-	0%	0%	0%	0%	-	0%
0%	-	0%	0%	0%	0%	-	0%
100%	5,25	0%	100%	0%	0%	-	0%
100%	5,85	0%	100%	0%	0%	-	0%
0%	-	0%	0%	0%	0%	-	0%
0%	-	0%	0%	0%	0%	-	0%
0%	-	0%	0%	0%	0%	-	0%
0%	-	0%	0%	0%	0%	-	0%
0%	-	0%	0%	0%	0%	-	0%
0%	-	0%	0%	0%	0%	-	0%
0%	-	0%	0%	0%	0%	-	0%
0%	-	0%	0%	0%	0%	-	0%
50%	3,92	0%	100%	0%	0%	-	0%
0%	-	0%	0%	0%	0%	-	0%
0%	-	0%	0%	0%	0%	-	0%
0%	-	0%	0%	0%	0%	-	0%
10%	3,10	0%	50%	50%	0%	-	0%
0%	-	0%	0%	0%	0%	-	0%
0%	-	0%	0%	0%	0%	-	0%
0%	-	0%	0%	0%	0%	-	0%
0%	-	0%	0%	0%	0%	-	0%
0%	-	0%	0%	0%	0%	-	0%
25%	4,75	0%	0%	100%	0%	-	0%
0%	-	0%	0%	0%	0%	-	0%
0%	-	0%	0%	0%	0%	-	0%
0%	-	0%	0%	0%	0%	-	0%
0%	-	0%	0%	0%	0%	-	0%
0%	-	0%	0%	0%	0%	-	0%
0%	-	0%	0%	0%	0%	-	0%
0%	-	0%	0%	0%	0%	-	0%
0%	-	0%	0%	0%	0%	-	0%
0%	-	0%	0%	0%	0%	-	0%
0%	-	0%	0%	0%	0%	-	0%
0%	-	0%	0%	0%	0%	-	0%
0%	-	0%	0%	0%	0%	-	0%
0%	-	0%	0%	0%	0%	-	0%
0%	-	0%	0%	0%	0%	-	0%
0%	-	0%	0%	0%	0%	-	0%
0%	-	0%	0%	0%	0%	-	0%
0%	-	0%	0%	0%	0%	-	0%
5%	-	0%	100%	0%	0%	-	0%
15%	-	50%	50%	0%	0%	-	0%
0%	-	0%	0%	0%	0%	-	0%
0%	-	0%	0%	0%	0%	-	0%
0%	-	0%	0%	0%	0%	-	0%
0%	-	0%	0%	0%	0%	-	0%
0%	-	0%	0%	0%	0%	-	0%
0%	-	0%	0%	0%	0%	-	0%
0%	-	0%	0%	0%	0%	-	0%
0%	-	0%	0%	0%	0%	-	0%
10%	3,10	40%	60%	0%	0%	-	0%
0%	-	0%	0%	0%	0%	-	0%
0%	-	0%	0%	0%	0%	-	0%
0%	-	0%	0%	0%	0%	-	0%
0%	-	0%	0%	0%	0%	-	0%
0%	-	0%	0%	0%	0%	-	0%
0%	-	0%	0%	0%	0%	-	0%
0%	-	0%	0%	0%	0%	-	0%
0%	-	0%	0%	0%	0%	-	0%
0%	-	0%	0%	0%	0%	-	0%
0%	-	0%	0%	0%	0%	-	0%
0%	-	0%	0%	0%	0%	-	0%
0%	-	0%	0%	0%	0%	-	0%
0%	-	0%	0%	0%	0%	-	0%
0%	-	0%	0%	0%	0%	-	0%
0%	-	0%	0%	0%	0%	-	0%

0%	-	0%	0%	0%	0%	-	0%
0%	-	0%	0%	0%	0%	-	0%
0%	-	0%	0%	0%	0%	-	0%
0%	-	0%	0%	0%	0%	-	0%
0%	-	0%	0%	0%	0%	-	0%
0%	-	0%	0%	0%	0%	-	0%
0%	-	0%	0%	0%	0%	-	0%
0%	-	0%	0%	0%	0%	-	0%
0%	-	0%	0%	0%	0%	-	0%
0%	-	0%	0%	0%	0%	-	0%
5%	5,06	15%	70%	15%	0%	-	0%
0%	-	0%	0%	0%	0%	-	0%
0%	-	0%	0%	0%	0%	-	0%
0%	-	0%	0%	0%	0%	-	0%
0%	-	0%	0%	0%	0%	-	0%
0%	-	0%	0%	0%	0%	-	0%
0%	-	0%	0%	0%	0%	-	0%
0%	-	0%	0%	0%	0%	-	0%
0%	-	0%	0%	0%	0%	-	0%
0%	-	0%	0%	0%	0%	-	0%
0%	-	0%	0%	0%	0%	-	0%
0%	-	0%	0%	0%	0%	-	0%
0%	-	0%	0%	0%	0%	-	0%
0%	-	0%	0%	0%	0%	-	0%
0%	-	0%	0%	0%	0%	-	0%
0%	-	0%	0%	0%	0%	-	0%
0%	-	0%	0%	0%	0%	-	0%
0%	-	0%	0%	0%	0%	-	0%
0%	-	0%	0%	0%	0%	-	0%
0%	-	0%	0%	0%	0%	-	0%
0%	-	0%	0%	0%	0%	-	0%
0%	-	0%	0%	0%	0%	-	0%
0%	-	0%	0%	0%	0%	-	0%
0%	-	0%	0%	0%	0%	-	0%
0%	-	0%	0%	0%	0%	-	0%
0%	-	0%	0%	0%	0%	-	0%
0%	-	0%	0%	0%	0%	-	0%
0%	-	0%	0%	0%	0%	-	0%
10%	4,77	0%	100%	0%	0%	-	0%
0%	-	0%	0%	0%	0%	-	0%
75%	1,80	0%	100%	0%	0%	-	0%
0%	-	0%	0%	0%	0%	-	0%
0%	-	0%	0%	0%	0%	-	0%
0%	-	0%	0%	0%	0%	-	0%
0%	-	0%	0%	0%	0%	-	0%
0%	-	0%	0%	0%	0%	-	0%
10%	10,60	60%	40%	0%	0%	-	0%
0%	-	0%	0%	0%	0%	-	0%
0%	-	0%	0%	0%	0%	-	0%
2%	0,27	0%	100%	0%	0%	-	0%
0%	-	0%	0%	0%	0%	-	0%
0%	-	0%	0%	0%	0%	-	0%
0%	-	0%	0%	0%	0%	-	0%
0%	-	0%	0%	0%	0%	-	0%
0%	-	0%	0%	0%	0%	-	0%
0%	-	0%	0%	0%	0%	-	0%
0%	-	0%	0%	0%	0%	-	0%
0%	-	0%	0%	0%	0%	-	0%
0%	-	0%	0%	0%	0%	-	0%
0%	-	0%	0%	0%	0%	-	0%
0%	-	0%	0%	0%	0%	-	0%
0%	-	0%	0%	0%	0%	-	0%
50%	8,10	0%	100%	0%	0%	-	0%
0%	-	0%	0%	0%	0%	-	0%
45%	6,84	0%	100%	0%	0%	-	0%
0%	-	0%	0%	0%	0%	-	0%

0%	-	0%	0%	0%	0%	-	0%
0%	-	0%	0%	0%	0%	-	0%
0%	-	0%	0%	0%	0%	-	0%
0%	-	0%	0%	0%	0%	-	0%
0%	-	0%	0%	0%	0%	-	0%
0%	-	0%	0%	0%	0%	-	0%
25%	4,05	25%	75%	0%	0%	-	0%
0%	-	0%	0%	0%	0%	-	0%
0%	-	0%	0%	0%	0%	-	0%
0%	-	0%	0%	0%	0%	-	0%
0%	-	0%	0%	0%	0%	-	0%
0%	-	0%	0%	0%	0%	-	0%
0%	-	0%	0%	0%	0%	-	0%
0%	-	0%	0%	0%	0%	-	0%
0%	-	0%	0%	0%	0%	-	0%
0%	-	0%	0%	0%	0%	-	0%
0%	-	0%	0%	0%	0%	-	0%
0%	-	0%	0%	0%	0%	-	0%
0%	-	0%	0%	0%	0%	-	0%
0%	-	0%	0%	0%	0%	-	0%
0%	-	0%	0%	0%	0%	-	0%
0%	-	0%	0%	0%	0%	-	0%
0%	-	0%	0%	0%	0%	-	0%
0%	-	0%	0%	0%	0%	-	0%
0%	-	0%	0%	0%	0%	-	0%
0%	-	0%	0%	0%	0%	-	0%
0%	-	0%	0%	0%	0%	-	0%
0%	-	0%	0%	0%	0%	-	0%
0%	-	0%	0%	0%	0%	-	0%
0%	-	0%	0%	0%	0%	-	0%
0%	-	0%	0%	0%	0%	-	0%
0%	-	0%	0%	0%	0%	-	0%
0%	-	0%	0%	0%	0%	-	0%
0%	-	0%	0%	0%	0%	-	0%
0%	-	0%	0%	0%	0%	-	0%
0%	-	0%	0%	0%	0%	-	0%
0%	-	0%	0%	0%	0%	-	0%
0%	-	0%	0%	0%	0%	-	0%
0%	-	0%	0%	0%	0%	-	0%
0%	-	0%	0%	0%	0%	-	0%
0%	-	0%	0%	0%	0%	-	0%
0%	-	0%	0%	0%	0%	-	0%
0%	-	0%	0%	0%	0%	-	0%
0%	-	0%	0%	0%	0%	-	0%
0%	-	0%	0%	0%	0%	-	0%
0%	-	0%	0%	0%	0%	-	0%
0%	-	0%	0%	0%	0%	-	0%
0%	-	0%	0%	0%	0%	-	0%
0%	-	0%	0%	0%	0%	-	0%
0%	-	0%	0%	0%	0%	-	0%
0%	-	0%	0%	0%	0%	-	0%
0%	-	0%	0%	0%	0%	-	0%
0%	-	0%	0%	0%	0%	-	0%
0%	-	0%	0%	0%	0%	-	0%
0%	-	0%	0%	0%	0%	-	0%
0%	-	0%	0%	0%	0%	-	0%
0%	-	0%	0%	0%	0%	-	0%
0%	-	0%	0%	0%	0%	-	0%
0%	-	0%	0%	0%	0%	-	0%
0%	-	0%	0%	0%	0%	-	0%
0%	-	0%	0%	0%	0%	-	0%
0%	-	0%	0%	0%	0%	-	0%
22%	4,40	30%	70%	0%	0%	-	0%
0%	-	0%	0%	0%	0%	-	0%
0%	-	0%	0%	0%	0%	-	0%
0%	-	0%	0%	0%	0%	-	0%

0%	-	0%	0%	0%	0%	-	0%
0%	-	0%	0%	0%	0%	-	0%
0%	-	0%	0%	0%	0%	-	0%
25%	3,30	40%	60%	0%	0%	-	0%
0%	-	0%	0%	0%	0%	-	0%
0%	-	0%	0%	0%	0%	-	0%
0%	-	0%	0%	0%	0%	-	0%
0%	-	0%	0%	0%	0%	-	0%
0%	-	0%	0%	0%	0%	-	0%
0%	-	0%	0%	0%	0%	-	0%
0%	-	0%	0%	0%	0%	-	0%
0%	-	0%	0%	0%	0%	-	0%
0%	-	0%	0%	0%	0%	-	0%
0%	-	0%	0%	0%	0%	-	0%
0%	-	0%	0%	0%	0%	-	0%
0%	-	0%	0%	0%	0%	-	0%
0%	-	0%	0%	0%	0%	-	0%
0%	-	0%	0%	0%	0%	-	0%
0%	-	0%	0%	0%	0%	-	0%
0%	-	0%	0%	0%	0%	-	0%
15%	3,06	0%	100%	0%	0%	-	0%
0%	-	0%	0%	0%	0%	-	0%
0%	-	0%	0%	0%	0%	-	0%
0%	-	0%	0%	0%	0%	-	0%
0%	-	0%	0%	0%	0%	-	0%
0%	-	0%	0%	0%	0%	-	0%
0%	-	0%	0%	0%	0%	-	0%
0%	-	0%	0%	0%	0%	-	0%
0%	-	0%	0%	0%	0%	-	0%
0%	-	0%	0%	0%	0%	-	0%
0%	-	0%	0%	0%	0%	-	0%
0%	-	0%	0%	0%	0%	-	0%
0%	-	0%	0%	0%	0%	-	0%
75%	19,69	100%	0%	0%	0%	-	0%
0%	-	0%	0%	0%	0%	-	0%
0%	-	0%	0%	0%	0%	-	0%
0%	-	0%	0%	0%	0%	-	0%
0%	-	0%	0%	0%	0%	-	0%
25%	15,09	0%	30%	70%	0%	-	0%
0%	-	0%	0%	0%	0%	-	0%
0%	-	0%	0%	0%	0%	-	0%
0%	-	0%	0%	0%	0%	-	0%
0%	-	0%	0%	0%	0%	-	0%
0%	-	0%	0%	0%	0%	-	0%
0%	-	0%	0%	0%	0%	-	0%
0%	-	0%	0%	0%	0%	-	0%
0%	-	0%	0%	0%	0%	-	0%
0%	-	0%	0%	0%	0%	-	0%
0%	-	0%	0%	0%	0%	-	0%
0%	-	0%	0%	0%	0%	-	0%
0%	-	0%	0%	0%	0%	-	0%
0%	-	0%	0%	0%	0%	-	0%
0%	-	0%	0%	0%	0%	-	0%
0%	-	0%	0%	0%	0%	-	0%
0%	-	0%	0%	0%	0%	-	0%
0%	-	0%	0%	0%	0%	-	0%
0%	-	0%	0%	0%	0%	-	0%
0%	-	0%	0%	0%	0%	-	0%
0%	-	0%	0%	0%	0%	-	0%
0%	-	0%	0%	0%	0%	-	0%
0%	-	0%	0%	0%	0%	-	0%
0%	-	0%	0%	0%	0%	-	0%
0%	-	0%	0%	0%	0%	-	0%
0%	-	0%	0%	0%	0%	-	0%
0%	-	0%	0%	0%	0%	-	0%
0%	-	0%	0%	0%	0%	-	0%

0%	-	0%	0%	0%	0%	-	0%
0%	-	0%	0%	0%	0%	-	0%
0%	-	0%	0%	0%	0%	-	0%
0%	-	0%	0%	0%	0%	-	0%
0%	-	0%	0%	0%	0%	-	0%
0%	-	0%	0%	0%	0%	-	0%
0%	-	0%	0%	0%	0%	-	0%
0%	-	0%	0%	0%	0%	-	0%
0%	-	0%	0%	0%	0%	-	0%
0%	-	0%	0%	0%	0%	-	0%
0%	-	0%	0%	0%	0%	-	0%
0%	-	0%	0%	0%	0%	-	0%
0%	-	0%	0%	0%	0%	-	0%
0%	-	0%	0%	0%	0%	-	0%
0%	-	0%	0%	0%	0%	-	0%
0%	-	0%	0%	0%	0%	-	0%
40%	3,20	0%	50%	50%	0%	-	0%
0%	-	0%	0%	0%	0%	-	0%
0%	-	0%	0%	0%	0%	-	0%
0%	-	0%	0%	0%	0%	-	0%
0%	-	0%	0%	0%	0%	-	0%
0%	-	0%	0%	0%	0%	-	0%
0%	-	0%	0%	0%	0%	-	0%
0%	-	0%	0%	0%	0%	-	0%
0%	-	0%	0%	0%	0%	-	0%
0%	-	0%	0%	0%	0%	-	0%
0%	-	0%	0%	0%	0%	-	0%
0%	-	0%	0%	0%	0%	-	0%
0%	-	0%	0%	0%	0%	-	0%
0%	-	0%	0%	0%	0%	-	0%
0%	-	0%	0%	0%	0%	-	0%
0%	-	0%	0%	0%	0%	-	0%
0%	-	0%	0%	0%	0%	-	0%
0%	-	0%	0%	0%	0%	-	0%
0%	-	0%	0%	0%	0%	-	0%
0%	-	0%	0%	0%	0%	-	0%
0%	-	0%	0%	0%	0%	-	0%
0%	-	0%	0%	0%	0%	-	0%
0%	-	0%	0%	0%	0%	-	0%
0%	-	0%	0%	0%	0%	-	0%
0%	-	0%	0%	0%	0%	-	0%
0%	-	0%	0%	0%	0%	-	0%
0%	-	0%	0%	0%	0%	-	0%
0%	-	0%	0%	0%	0%	-	0%
0%	-	0%	0%	0%	0%	-	0%
0%	-	0%	0%	0%	0%	-	0%
0%	-	0%	0%	0%	0%	-	0%
0%	-	0%	0%	0%	0%	-	0%
0%	-	0%	0%	0%	0%	-	0%
0%	-	0%	0%	0%	0%	-	0%
0%	-	0%	0%	0%	0%	-	0%
0%	-	0%	0%	0%	0%	-	0%
20%	7,40	50%	50%	0%	0%	-	0%
0%	-	0%	0%	0%	0%	-	0%
0%	-	0%	0%	0%	0%	-	0%
65%	19,50	100%	0%	0%	0%	-	0%
0%	-	0%	0%	0%	0%	-	0%
0%	-	0%	0%	0%	0%	-	0%
0%	-	0%	0%	0%	0%	-	0%
0%	-	0%	0%	0%	0%	-	0%
0%	-	0%	0%	0%	0%	-	0%
0%	-	0%	0%	0%	0%	-	0%
0%	-	0%	0%	0%	0%	-	0%
0%	-	0%	0%	0%	0%	-	0%
0%	-	0%	0%	0%	0%	-	0%
0%	-	0%	0%	0%	0%	-	0%

0%	-	0%	0%	0%	0%	-	0%
0%	-	0%	0%	0%	0%	-	0%
0%	-	0%	0%	0%	0%	-	0%
0%	-	0%	0%	0%	0%	-	0%
0%	-	0%	0%	0%	0%	-	0%
0%	-	0%	0%	0%	0%	-	0%
59%	5,88	0%	20%	80%	0%	-	0%
0%	-	0%	0%	0%	0%	-	0%
0%	-	0%	0%	0%	0%	-	0%
15%	2,83	0%	0%	100%	0%	-	0%
0%	-	0%	0%	0%	0%	-	0%
0%	-	0%	0%	0%	0%	-	0%
0%	-	0%	0%	0%	0%	-	0%
0%	-	0%	0%	0%	0%	-	0%
0%	-	0%	0%	0%	0%	-	0%
0%	-	0%	0%	0%	0%	-	0%
0%	-	0%	0%	0%	0%	-	0%
0%	-	0%	0%	0%	0%	-	0%
0%	-	0%	0%	0%	0%	-	0%
0%	-	0%	0%	0%	0%	-	0%
0%	-	0%	0%	0%	0%	-	0%
0%	-	0%	0%	0%	0%	-	0%
0%	-	0%	0%	0%	0%	-	0%
0%	-	0%	0%	0%	0%	-	0%
0%	-	0%	0%	0%	0%	-	0%
0%	-	0%	0%	0%	0%	-	0%
20%	16,00	20%	80%	0%	0%	-	0%
25%	9,38	0%	60%	40%	0%	-	0%
0%	-	0%	0%	0%	0%	-	0%
0%	-	0%	0%	0%	0%	-	0%
19%	11,21	100%	0%	0%	0%	-	0%
15%	7,95	0%	100%	0%	0%	-	0%
0%	-	0%	0%	0%	0%	-	0%
0%	-	0%	0%	0%	0%	-	0%
10%	2,60	0%	100%	0%	0%	-	0%
0%	-	0%	0%	0%	0%	-	0%
30%	8,70	0%	0%	100%	0%	-	0%
0%	-	0%	0%	0%	0%	-	0%
0%	-	0%	0%	0%	0%	-	0%
0%	-	0%	0%	0%	0%	-	0%
0%	-	0%	0%	0%	0%	-	0%
0%	-	0%	0%	0%	0%	-	0%
0%	-	0%	0%	0%	0%	-	0%
0%	-	0%	0%	0%	0%	-	0%
0%	-	0%	0%	0%	0%	-	0%
0%	-	0%	0%	0%	0%	-	0%
0%	-	0%	0%	0%	0%	-	0%
0%	-	0%	0%	0%	0%	-	0%
0%	-	0%	0%	0%	0%	-	0%
0%	-	0%	0%	0%	0%	-	0%
25%	2,00	100%	0%	0%	0%	-	0%
0%	-	0%	0%	0%	0%	-	0%
0%	-	0%	0%	0%	0%	-	0%
0%	-	0%	0%	0%	0%	-	0%
0%	-	0%	0%	0%	0%	-	0%
0%	-	0%	0%	0%	0%	-	0%
0%	-	0%	0%	0%	0%	-	0%
0%	-	0%	0%	0%	0%	-	0%
0%	-	0%	0%	0%	0%	-	0%
0%	-	0%	0%	0%	0%	-	0%
0%	-	0%	0%	0%	0%	-	0%
0%	-	0%	0%	0%	0%	-	0%
0%	-	0%	0%	0%	0%	-	0%
0%	-	0%	0%	0%	0%	-	0%
0%	-	0%	0%	0%	0%	-	0%
0%	-	0%	0%	0%	0%	-	0%

0%	-	0%	0%	0%	0%	-	0%
0%	-	0%	0%	0%	0%	-	0%
0%	-	0%	0%	0%	0%	-	0%
0%	-	0%	0%	0%	0%	-	0%
0%	-	0%	0%	0%	0%	-	0%
0%	-	0%	0%	0%	0%	-	0%
0%	-	0%	0%	0%	0%	-	0%
0%	-	0%	0%	0%	0%	-	0%
0%	-	0%	0%	0%	0%	-	0%
0%	-	0%	0%	0%	0%	-	0%
0%	-	0%	0%	0%	0%	-	0%
0%	-	0%	0%	0%	0%	-	0%
0%	-	0%	0%	0%	0%	-	0%
0%	-	0%	0%	0%	0%	-	0%
0%	-	0%	0%	0%	0%	-	0%
0%	-	0%	0%	0%	0%	-	0%
0%	-	0%	0%	0%	0%	-	0%
0%	-	0%	0%	0%	0%	-	0%
0%	-	0%	0%	0%	0%	-	0%
0%	-	0%	0%	0%	0%	-	0%
0%	-	0%	0%	0%	0%	-	0%
0%	-	0%	0%	0%	0%	-	0%
0%	-	0%	0%	0%	0%	-	0%
0%	-	0%	0%	0%	0%	-	0%
14%	2,94	0%	100%	0%	0%	-	0%
0%	-	0%	0%	0%	0%	-	0%
0%	-	0%	0%	0%	0%	-	0%
0%	-	0%	0%	0%	0%	-	0%
0%	-	0%	0%	0%	0%	-	0%
0%	-	0%	0%	0%	0%	-	0%
0%	-	0%	0%	0%	0%	-	0%
0%	-	0%	0%	0%	0%	-	0%
0%	-	0%	0%	0%	0%	-	0%
0%	-	0%	0%	0%	0%	-	0%
0%	-	0%	0%	0%	0%	-	0%
0%	-	0%	0%	0%	0%	-	0%
0%	-	0%	0%	0%	0%	-	0%
0%	-	0%	0%	0%	0%	-	0%
0%	-	0%	0%	0%	0%	-	0%
0%	-	0%	0%	0%	0%	-	0%
0%	-	0%	0%	0%	0%	-	0%
0%	-	0%	0%	0%	0%	-	0%
0%	-	0%	0%	0%	0%	-	0%
0%	-	0%	0%	0%	0%	-	0%
0%	-	0%	0%	0%	0%	-	0%
0%	-	0%	0%	0%	0%	-	0%
0%	-	0%	0%	0%	0%	-	0%
0%	-	0%	0%	0%	0%	-	0%
0%	-	0%	0%	0%	0%	-	0%
0%	-	0%	0%	0%	0%	-	0%
0%	-	0%	0%	0%	0%	-	0%
0%	-	0%	0%	0%	0%	-	0%
0%	-	0%	0%	0%	0%	-	0%
0%	-	0%	0%	0%	0%	-	0%
0%	-	0%	0%	0%	0%	-	0%
0%	-	0%	0%	0%	0%	-	0%
0%	-	0%	0%	0%	0%	-	0%
0%	-	0%	0%	0%	0%	-	0%
0%	-	0%	0%	0%	0%	-	0%
0%	-	0%	0%	0%	0%	-	0%
0%	-	0%	0%	0%	0%	-	0%
0%	-	0%	0%	0%	0%	-	0%
0%	-	0%	0%	0%	0%	-	0%
0%	-	0%	0%	0%	0%	-	0%
0%	-	0%	0%	0%	0%	-	0%
0%	-	0%	0%	0%	0%	-	0%

0%	-	0%	0%	0%	0%	-	0%
0%	-	0%	0%	0%	0%	-	0%
0%	-	0%	0%	0%	0%	-	0%
0%	-	0%	0%	0%	0%	-	0%
0%	-	0%	0%	0%	0%	-	0%
0%	-	0%	0%	0%	0%	-	0%
0%	-	0%	0%	0%	0%	-	0%
0%	-	0%	0%	0%	0%	-	0%
0%	-	0%	0%	0%	0%	-	0%
0%	-	0%	0%	0%	0%	-	0%
0%	-	0%	0%	0%	0%	-	0%
0%	-	0%	0%	0%	0%	-	0%
0%	-	0%	0%	0%	0%	-	0%
0%	-	0%	0%	0%	0%	-	0%
0%	-	0%	0%	0%	0%	-	0%
0%	-	0%	0%	0%	0%	-	0%
0%	-	0%	0%	0%	0%	-	0%
0%	-	0%	0%	0%	0%	-	0%
0%	-	0%	0%	0%	0%	-	0%
0%	-	0%	0%	0%	0%	-	0%
0%	-	0%	0%	0%	0%	-	0%
0%	-	0%	0%	0%	0%	-	0%
0%	-	0%	0%	0%	0%	-	0%
0%	-	0%	0%	0%	0%	-	0%
0%	-	0%	0%	0%	0%	-	0%
0%	-	0%	0%	0%	0%	-	0%
0%	-	0%	0%	0%	0%	-	0%
0%	-	0%	0%	0%	0%	-	0%
0%	-	0%	0%	0%	0%	-	0%
0%	-	0%	0%	0%	0%	-	0%
60%	-	60%	40%	0%	0%	-	0%
0%	-	0%	0%	0%	0%	-	0%
0%	-	0%	0%	0%	0%	-	0%
0%	-	0%	0%	0%	0%	-	0%
0%	-	0%	0%	0%	0%	-	0%
0%	-	0%	0%	0%	0%	-	0%
0%	-	0%	0%	0%	0%	-	0%
0%	-	0%	0%	0%	0%	-	0%
0%	-	0%	0%	0%	0%	-	0%
0%	-	0%	0%	0%	0%	-	0%
0%	-	0%	0%	0%	0%	-	0%
0%	-	0%	0%	0%	0%	-	0%
0%	-	0%	0%	0%	0%	-	0%
0%	-	0%	0%	0%	0%	-	0%
0%	-	0%	0%	0%	0%	-	0%
0%	-	0%	0%	0%	0%	-	0%
0%	-	0%	0%	0%	0%	-	0%
0%	-	0%	0%	0%	0%	-	0%
0%	-	0%	0%	0%	0%	-	0%
0%	-	0%	0%	0%	0%	-	0%
0%	-	0%	0%	0%	0%	-	0%
0%	-	0%	0%	0%	0%	-	0%
0%	-	0%	0%	0%	0%	-	0%
0%	-	0%	0%	0%	0%	-	0%
0%	-	0%	0%	0%	0%	-	0%
0%	-	0%	0%	0%	0%	-	0%
0%	-	0%	0%	0%	0%	-	0%
0%	-	0%	0%	0%	0%	-	0%
0%	-	0%	0%	0%	0%	-	0%
0%	-	0%	0%	0%	0%	-	0%
0%	-	0%	0%	0%	0%	-	0%
0%	-	0%	0%	0%	0%	-	0%
0%	-	0%	0%	0%	0%	-	0%
0%	-	0%	0%	0%	0%	-	0%
0%	-	0%	0%	0%	0%	-	0%
0%	-	0%	0%	0%	0%	-	0%

0%	-	0%	0%	0%	0%	-	0%
0%	-	0%	0%	0%	0%	-	0%
0%	-	0%	0%	0%	0%	-	0%
0%	-	0%	0%	0%	0%	-	0%
0%	-	0%	0%	0%	0%	-	0%
0%	-	0%	0%	0%	0%	-	0%
0%	-	0%	0%	0%	0%	-	0%
0%	-	0%	0%	0%	0%	-	0%
0%	-	0%	0%	0%	0%	-	0%
0%	-	0%	0%	0%	0%	-	0%
0%	-	0%	0%	0%	0%	-	0%
0%	-	0%	0%	0%	0%	-	0%
0%	-	0%	0%	0%	0%	-	0%
0%	-	0%	0%	0%	0%	-	0%
0%	-	0%	0%	0%	0%	-	0%
0%	-	0%	0%	0%	0%	-	0%
0%	-	0%	0%	0%	0%	-	0%
0%	-	0%	0%	0%	0%	-	0%
0%	-	0%	0%	0%	0%	-	0%
0%	-	0%	0%	0%	0%	-	0%
0%	-	0%	0%	0%	0%	-	0%
0%	-	0%	0%	0%	0%	-	0%
0%	-	0%	0%	0%	0%	-	0%
0%	-	0%	0%	0%	0%	-	0%
0%	-	0%	0%	0%	0%	-	0%
0%	-	0%	0%	0%	0%	-	0%
0%	-	0%	0%	0%	0%	-	0%
0%	-	0%	0%	0%	0%	-	0%
0%	-	0%	0%	0%	0%	-	0%
0%	-	0%	0%	0%	0%	-	0%
0%	-	0%	0%	0%	0%	-	0%
0%	-	0%	0%	0%	0%	-	0%
0%	-	0%	0%	0%	0%	-	0%
0%	-	0%	0%	0%	0%	-	0%
0%	-	0%	0%	0%	0%	-	0%
0%	-	0%	0%	0%	0%	-	0%
0%	-	0%	0%	0%	0%	-	0%
0%	-	0%	0%	0%	0%	-	0%
0%	-	0%	0%	0%	0%	-	0%
0%	-	0%	0%	0%	0%	-	0%
0%	-	0%	0%	0%	0%	-	0%
0%	-	0%	0%	0%	0%	-	0%
0%	-	0%	0%	0%	0%	-	0%
0%	-	0%	0%	0%	0%	-	0%
0%	-	0%	0%	0%	0%	-	0%
0%	-	0%	0%	0%	0%	-	0%
20%	-	100%	0%	0%	0%	-	0%
0%	-	0%	0%	0%	0%	-	0%
0%	-	0%	0%	0%	0%	-	0%
0%	-	0%	0%	0%	0%	-	0%
0%	-	0%	0%	0%	0%	-	0%
0%	-	0%	0%	0%	0%	-	0%
0%	-	0%	0%	0%	0%	-	0%
0%	-	0%	0%	0%	0%	-	0%
0%	-	0%	0%	0%	0%	-	0%
0%	-	0%	0%	0%	0%	-	0%
0%	-	0%	0%	0%	0%	-	0%
0%	-	0%	0%	0%	0%	-	0%
0%	-	0%	0%	0%	0%	-	0%
0%	-	0%	0%	0%	0%	-	0%
0%	-	0%	0%	0%	0%	-	0%
0%	-	0%	0%	0%	0%	-	0%
0%	-	0%	0%	0%	0%	-	0%
67%	-	100%	0%	0%	0%	-	0%
67%	-	100%	0%	0%	0%	-	0%
47%	14,10	0%	0%	100%	0%	-	0%

11%	5,06	0%	0%	100%	0%	-	0%
0%	-	0%	0%	0%	0%	-	0%
0%	-	0%	0%	0%	0%	-	0%
0%	-	0%	0%	0%	0%	-	0%
0%	-	0%	0%	0%	0%	-	0%
0%	-	0%	0%	0%	0%	-	0%
0%	-	0%	0%	0%	0%	-	0%
0%	-	0%	0%	0%	0%	-	0%
0%	-	0%	0%	0%	0%	-	0%
0%	-	0%	0%	0%	0%	-	0%
0%	-	0%	0%	0%	0%	-	0%
0%	-	0%	0%	0%	0%	-	0%
0%	-	0%	0%	0%	0%	-	0%
0%	-	0%	0%	0%	0%	-	0%
0%	-	0%	0%	0%	0%	-	0%
0%	-	0%	0%	0%	0%	-	0%
0%	-	0%	0%	0%	0%	-	0%
0%	-	0%	0%	0%	0%	-	0%
0%	-	0%	0%	0%	0%	-	0%
0%	-	0%	0%	0%	0%	-	0%
0%	-	0%	0%	0%	0%	-	0%
0%	-	0%	0%	0%	0%	-	0%
0%	-	0%	0%	0%	0%	-	0%
0%	-	0%	0%	0%	0%	-	0%
0%	-	0%	0%	0%	0%	-	0%
0%	-	0%	0%	0%	0%	-	0%
53%	9,00	100%	0%	0%	0%	-	0%
0%	-	0%	0%	0%	0%	-	0%
0%	-	0%	0%	0%	0%	-	0%
0%	-	0%	0%	0%	0%	-	0%
0%	-	0%	0%	0%	0%	-	0%
0%	-	0%	0%	0%	0%	-	0%
0%	-	0%	0%	0%	0%	-	0%
0%	-	0%	0%	0%	0%	-	0%
0%	-	0%	0%	0%	0%	-	0%
0%	-	0%	0%	0%	0%	-	0%
0%	-	0%	0%	0%	0%	-	0%
0%	-	0%	0%	0%	0%	-	0%
0%	-	0%	0%	0%	0%	-	0%
0%	-	0%	0%	0%	0%	-	0%
0%	-	0%	0%	0%	0%	-	0%
0%	-	0%	0%	0%	0%	-	0%
0%	-	0%	0%	0%	0%	-	0%
0%	-	0%	0%	0%	0%	-	0%
0%	-	0%	0%	0%	0%	-	0%
0%	-	0%	0%	0%	0%	-	0%
0%	-	0%	0%	0%	0%	-	0%
0%	-	0%	0%	0%	0%	-	0%
0%	-	0%	0%	0%	0%	-	0%
0%	-	0%	0%	0%	0%	-	0%
0%	-	0%	0%	0%	0%	-	0%
0%	-	0%	0%	0%	0%	-	0%
0%	-	0%	0%	0%	0%	-	0%
0%	-	0%	0%	0%	0%	-	0%
0%	-	0%	0%	0%	0%	-	0%
0%	-	0%	0%	0%	0%	-	0%
0%	-	0%	0%	0%	0%	-	0%
0%	-	0%	0%	0%	0%	-	0%
0%	-	0%	0%	0%	0%	-	0%
0%	-	0%	0%	0%	0%	-	0%
0%	-	0%	0%	0%	0%	-	0%
0%	-	0%	0%	0%	0%	-	0%
15%	3,15	100%	0%	0%	0%	-	0%
0%	-	0%	0%	0%	0%	-	0%
70%	46,48	60%	40%	0%	0%	-	0%
0%	-	0%	0%	0%	0%	-	0%

5%	2,13	0%	0%	100%	0%	-	0%
0%	-	0%	0%	0%	0%	-	0%
20%	5,10	100%	0%	0%	0%	-	0%
0%	-	0%	0%	0%	0%	-	0%
0%	-	0%	0%	0%	0%	-	0%
0%	-	0%	0%	0%	0%	-	0%
0%	-	0%	0%	0%	0%	-	0%
20%	5,00	100%	0%	0%	0%	-	0%
20%	4,60	0%	0%	100%	0%	-	0%
100%	4,00	30%	20%	50%	0%	-	0%
0%	-	0%	0%	0%	0%	-	0%
0%	-	0%	0%	0%	0%	-	0%
0%	-	0%	0%	0%	0%	-	0%
0%	-	0%	0%	0%	0%	-	0%
0%	-	0%	0%	0%	0%	-	0%
0%	-	0%	0%	0%	0%	-	0%
0%	-	0%	0%	0%	0%	-	0%
0%	-	0%	0%	0%	0%	-	0%
0%	-	0%	0%	0%	0%	-	0%
0%	-	0%	0%	0%	0%	-	0%
0%	-	0%	0%	0%	0%	-	0%
0%	-	0%	0%	0%	0%	-	0%
0%	-	0%	0%	0%	0%	-	0%
0%	-	0%	0%	0%	0%	-	0%
0%	-	0%	0%	0%	0%	-	0%
0%	-	0%	0%	0%	0%	-	0%
0%	-	0%	0%	0%	0%	-	0%
0%	-	0%	0%	0%	0%	-	0%
0%	-	0%	0%	0%	0%	-	0%
0%	-	0%	0%	0%	0%	-	0%
0%	-	0%	0%	0%	0%	-	0%
0%	-	0%	0%	0%	0%	-	0%
0%	-	0%	0%	0%	0%	-	0%
0%	-	0%	0%	0%	0%	-	0%
0%	-	0%	0%	0%	0%	-	0%
0%	-	0%	0%	0%	0%	-	0%
0%	-	0%	0%	0%	0%	-	0%
0%	-	0%	0%	0%	0%	-	0%
0%	-	0%	0%	0%	0%	-	0%
0%	-	0%	0%	0%	0%	-	0%
0%	-	0%	0%	0%	0%	-	0%
0%	-	0%	0%	0%	0%	-	0%
0%	-	0%	0%	0%	0%	-	0%
0%	-	0%	0%	0%	0%	-	0%
0%	-	0%	0%	0%	0%	-	0%
0%	-	0%	0%	0%	0%	-	0%
0%	-	0%	0%	0%	0%	-	0%
0%	-	0%	0%	0%	0%	-	0%
0%	-	0%	0%	0%	0%	-	0%
0%	-	0%	0%	0%	0%	-	0%
0%	-	0%	0%	0%	0%	-	0%
0%	-	0%	0%	0%	0%	-	0%
0%	-	0%	0%	0%	0%	-	0%
0%	-	0%	0%	0%	0%	-	0%
0%	-	0%	0%	0%	0%	-	0%
0%	-	0%	0%	0%	0%	-	0%
0%	-	0%	0%	0%	0%	-	0%
0%	-	0%	0%	0%	0%	-	0%
0%	-	0%	0%	0%	0%	-	0%
25%	5,55	0%	100%	0%	0%	-	0%
0%	-	0%	0%	0%	0%	-	0%
0%	-	0%	0%	0%	0%	-	0%
0%	-	0%	0%	0%	0%	-	0%
0%	-	0%	0%	0%	0%	-	0%
0%	-	0%	0%	0%	0%	-	0%
0%	-	0%	0%	0%	0%	-	0%

0%	-	0%	0%	0%	0%	-	0%
0%	-	0%	0%	0%	0%	-	0%
0%	-	0%	0%	0%	0%	-	0%
0%	-	0%	0%	0%	0%	-	0%
0%	-	0%	0%	0%	0%	-	0%
0%	-	0%	0%	0%	0%	-	0%
0%	-	0%	0%	0%	0%	-	0%
0%	-	0%	0%	0%	0%	-	0%
0%	-	0%	0%	0%	0%	-	0%
0%	-	0%	0%	0%	0%	-	0%
0%	-	0%	0%	0%	0%	-	0%
0%	-	0%	0%	0%	0%	-	0%
0%	-	0%	0%	0%	0%	-	0%
0%	-	0%	0%	0%	0%	-	0%
0%	-	0%	0%	0%	0%	-	0%
0%	-	0%	0%	0%	0%	-	0%
0%	-	0%	0%	0%	0%	-	0%
0%	-	0%	0%	0%	0%	-	0%
0%	-	0%	0%	0%	0%	-	0%
0%	-	0%	0%	0%	0%	-	0%
0%	-	0%	0%	0%	0%	-	0%
20%	7,71	0%	100%	0%	0%	-	0%
0%	-	0%	0%	0%	0%	-	0%
0%	-	0%	0%	0%	0%	-	0%
0%	-	0%	0%	0%	0%	-	0%
0%	-	0%	0%	0%	0%	-	0%
60%	11,88	0%	0%	100%	0%	-	0%
0%	-	0%	0%	0%	0%	-	0%
0%	-	0%	0%	0%	0%	-	0%
0%	-	0%	0%	0%	0%	-	0%
0%	-	0%	0%	0%	0%	-	0%
0%	-	0%	0%	0%	0%	-	0%
0%	-	0%	0%	0%	0%	-	0%
0%	-	0%	0%	0%	0%	-	0%
8%	1,52	100%	0%	0%	0%	-	0%
0%	-	0%	0%	0%	0%	-	0%
0%	-	0%	0%	0%	0%	-	0%
0%	-	0%	0%	0%	0%	-	0%
0%	-	0%	0%	0%	0%	-	0%
0%	-	0%	0%	0%	0%	-	0%
0%	-	0%	0%	0%	0%	-	0%
0%	-	0%	0%	0%	0%	-	0%
0%	-	0%	0%	0%	0%	-	0%
0%	-	0%	0%	0%	0%	-	0%
0%	-	0%	0%	0%	0%	-	0%
0%	-	0%	0%	0%	0%	-	0%
0%	-	0%	0%	0%	0%	-	0%
0%	-	0%	0%	0%	0%	-	0%
0%	-	0%	0%	0%	0%	-	0%
0%	-	0%	0%	0%	0%	-	0%
0%	-	0%	0%	0%	0%	-	0%
0%	-	0%	0%	0%	0%	-	0%
0%	-	0%	0%	0%	0%	-	0%
0%	-	0%	0%	0%	0%	-	0%
0%	-	0%	0%	0%	0%	-	0%
0%	-	0%	0%	0%	0%	-	0%
0%	-	0%	0%	0%	0%	-	0%
20%	7,60	30%	70%	0%	0%	-	0%
0%	-	0%	0%	0%	0%	-	0%
0%	-	0%	0%	0%	0%	-	0%
0%	-	0%	0%	0%	0%	-	0%
0%	-	0%	0%	0%	0%	-	0%
0%	-	0%	0%	0%	0%	-	0%
25%	3,75	0%	65%	35%	0%	-	0%
0%	-	0%	0%	0%	0%	-	0%
64%	10,24	100%	0%	0%	0%	-	0%

0%	-	0%	0%	0%	0%	-	0%
0%	-	0%	0%	0%	0%	-	0%
0%	-	0%	0%	0%	0%	-	0%
0%	-	0%	0%	0%	0%	-	0%
0%	-	0%	0%	0%	0%	-	0%
0%	-	0%	0%	0%	0%	-	0%
25%	11,25	100%	0%	0%	0%	-	0%
0%	-	0%	0%	0%	0%	-	0%
0%	-	0%	0%	0%	0%	-	0%
0%	-	0%	0%	0%	0%	-	0%
0%	-	0%	0%	0%	0%	-	0%
0%	-	0%	0%	0%	0%	-	0%
0%	-	0%	0%	0%	0%	-	0%
0%	-	0%	0%	0%	0%	-	0%
0%	-	0%	0%	0%	0%	-	0%
0%	-	0%	0%	0%	0%	-	0%
0%	-	0%	0%	0%	0%	-	0%
0%	-	0%	0%	0%	0%	-	0%
0%	-	0%	0%	0%	0%	-	0%
0%	-	0%	0%	0%	0%	-	0%
0%	-	0%	0%	0%	0%	-	0%
0%	-	0%	0%	0%	0%	-	0%
20%	-	100%	0%	0%	0%	-	0%
0%	-	0%	0%	0%	0%	-	0%
22%	5,00	60%	40%	0%	0%	-	0%
0%	-	0%	0%	0%	0%	-	0%
0%	-	0%	0%	0%	0%	-	0%
0%	-	0%	0%	0%	0%	-	0%
0%	-	0%	0%	0%	0%	-	0%
0%	-	0%	0%	0%	0%	-	0%
0%	-	0%	0%	0%	0%	-	0%
23%	11,50	0%	0%	100%	0%	-	0%
0%	-	0%	0%	0%	0%	-	0%
0%	-	0%	0%	0%	0%	-	0%
0%	-	0%	0%	0%	0%	-	0%
0%	-	0%	0%	0%	0%	-	0%
0%	-	0%	0%	0%	0%	-	0%
0%	-	0%	0%	0%	0%	-	0%
0%	-	0%	0%	0%	0%	-	0%
0%	-	0%	0%	0%	0%	-	0%
0%	-	0%	0%	0%	0%	-	0%
0%	-	0%	0%	0%	0%	-	0%
0%	-	0%	0%	0%	0%	-	0%
0%	-	0%	0%	0%	0%	-	0%
0%	-	0%	0%	0%	0%	-	0%
0%	-	0%	0%	0%	0%	-	0%
0%	-	0%	0%	0%	0%	-	0%
0%	-	0%	0%	0%	0%	-	0%
0%	-	0%	0%	0%	0%	-	0%
0%	-	0%	0%	0%	0%	-	0%
0%	-	0%	0%	0%	0%	-	0%
0%	-	0%	0%	0%	0%	-	0%
0%	-	0%	0%	0%	0%	-	0%
0%	-	0%	0%	0%	0%	-	0%
16%	4,00	100%	0%	0%	0%	-	0%
0%	-	0%	0%	0%	0%	-	0%
0%	-	0%	0%	0%	0%	-	0%
0%	-	0%	0%	0%	0%	-	0%
0%	-	0%	0%	0%	0%	-	0%
0%	-	0%	0%	0%	0%	-	0%
0%	-	0%	0%	0%	0%	-	0%
0%	-	0%	0%	0%	0%	-	0%
0%	-	0%	0%	0%	0%	-	0%
0%	-	0%	0%	0%	0%	-	0%
0%	-	0%	0%	0%	0%	-	0%
0%	-	0%	0%	0%	0%	-	0%
0%	-	0%	0%	0%	0%	-	0%

0%	-	0%	0%	0%	0%	-	0%
0%	-	0%	0%	0%	0%	-	0%
0%	-	0%	0%	0%	0%	-	0%
0%	-	0%	0%	0%	0%	-	0%
0%	-	0%	0%	0%	0%	-	0%
0%	-	0%	0%	0%	0%	-	0%
0%	-	0%	0%	0%	0%	-	0%
0%	-	0%	0%	0%	0%	-	0%
0%	-	0%	0%	0%	0%	-	0%
0%	-	0%	0%	0%	0%	-	0%
0%	-	0%	0%	0%	0%	-	0%
0%	-	0%	0%	0%	0%	-	0%
0%	-	0%	0%	0%	0%	-	0%
0%	-	0%	0%	0%	0%	-	0%
0%	-	0%	0%	0%	0%	-	0%
0%	-	0%	0%	0%	0%	-	0%
0%	-	0%	0%	0%	0%	-	0%
0%	-	0%	0%	0%	0%	-	0%
0%	-	0%	0%	0%	0%	-	0%
0%	-	0%	0%	0%	0%	-	0%
0%	-	0%	0%	0%	0%	-	0%
0%	-	0%	0%	0%	0%	-	0%
0%	-	0%	0%	0%	0%	-	0%
0%	-	0%	0%	0%	0%	-	0%
0%	-	0%	0%	0%	0%	-	0%
0%	-	0%	0%	0%	0%	-	0%
0%	-	0%	0%	0%	0%	-	0%
0%	-	0%	0%	0%	0%	-	0%
0%	-	0%	0%	0%	0%	-	0%
0%	-	0%	0%	0%	0%	-	0%
0%	-	0%	0%	0%	0%	-	0%
0%	-	0%	0%	0%	0%	-	0%
0%	-	0%	0%	0%	0%	-	0%
0%	-	0%	0%	0%	0%	-	0%
0%	-	0%	0%	0%	0%	-	0%
12%	2,00	100%	0%	0%	0%	-	0%
0%	-	0%	0%	0%	0%	-	0%
0%	-	0%	0%	0%	0%	-	0%
0%	-	0%	0%	0%	0%	-	0%
30%	8,21	50%	50%	0%	0%	-	0%
0%	-	0%	0%	0%	0%	-	0%
0%	-	0%	0%	0%	0%	-	0%
7%	3,85	0%	100%	0%	0%	-	0%
0%	-	0%	0%	0%	0%	-	0%
0%	-	0%	0%	0%	0%	-	0%
0%	-	0%	0%	0%	0%	-	0%
0%	-	0%	0%	0%	0%	-	0%
0%	-	0%	0%	0%	0%	-	0%
20%	66,00	60%	40%	0%	0%	-	0%
0%	-	0%	0%	0%	0%	-	0%
0%	-	0%	0%	0%	0%	-	0%
0%	-	0%	0%	0%	0%	-	0%
0%	-	0%	0%	0%	0%	-	0%
0%	-	0%	0%	0%	0%	-	0%
0%	-	0%	0%	0%	0%	-	0%
0%	-	0%	0%	0%	0%	-	0%
0%	-	0%	0%	0%	0%	-	0%
0%	-	0%	0%	0%	0%	-	0%
0%	-	0%	0%	0%	0%	-	0%
0%	-	0%	0%	0%	0%	-	0%
60%	1,73	0%	100%	0%	0%	-	0%
0%	-	0%	0%	0%	0%	-	0%
30%	-	100%	0%	0%	0%	-	0%
25%	-	100%	0%	0%	0%	-	0%
50%	-	100%	0%	0%	0%	-	0%
10%	-	100%	0%	0%	0%	-	0%

0%	-	0%	0%	0%	0%	-	0%
0%	-	0%	0%	0%	0%	-	0%
0%	-	0%	0%	0%	0%	-	0%
0%	-	0%	0%	0%	0%	-	0%
60%	-	100%	0%	0%	0%	-	0%
0%	-	0%	0%	0%	0%	-	0%
0%	-	0%	0%	0%	0%	-	0%
15%	-	100%	0%	0%	0%	-	0%
0%	-	0%	0%	0%	0%	-	0%
0%	-	0%	0%	0%	0%	-	0%
15%	-	100%	0%	0%	0%	-	0%
8%	-	100%	0%	0%	0%	-	0%
30%	-	100%	0%	0%	0%	-	0%
0%	-	0%	0%	0%	0%	-	0%
10%	-	100%	0%	0%	0%	-	0%
0%	-	0%	0%	0%	0%	-	0%
0%	-	0%	0%	0%	0%	-	0%
0%	-	0%	0%	0%	0%	-	0%
10%	-	100%	0%	0%	0%	-	0%
0%	-	0%	0%	0%	0%	-	0%
0%	-	0%	0%	0%	0%	-	0%
0%	-	0%	0%	0%	0%	-	0%
0%	-	0%	0%	0%	0%	-	0%
0%	-	0%	0%	0%	0%	-	0%
0%	-	0%	0%	0%	0%	-	0%
0%	-	0%	0%	0%	0%	-	0%
0%	-	0%	0%	0%	0%	-	0%
30%	-	100%	0%	0%	0%	-	0%
0%	-	0%	0%	0%	0%	-	0%
0%	-	0%	0%	0%	0%	-	0%
0%	-	0%	0%	0%	0%	-	0%
0%	-	0%	0%	0%	0%	-	0%
0%	-	0%	0%	0%	0%	-	0%
0%	-	0%	0%	0%	0%	-	0%
0%	-	0%	0%	0%	0%	-	0%
0%	-	0%	0%	0%	0%	-	0%
0%	-	0%	0%	0%	0%	-	0%
0%	-	0%	0%	0%	0%	-	0%
0%	-	0%	0%	0%	0%	-	0%
0%	-	0%	0%	0%	0%	-	0%
0%	-	0%	0%	0%	0%	-	0%
11%	2,00	0%	100%	0%	0%	-	0%
0%	-	0%	0%	0%	0%	-	0%
0%	-	0%	0%	0%	0%	-	0%
0%	-	0%	0%	0%	0%	-	0%
0%	-	0%	0%	0%	0%	-	0%
0%	-	0%	0%	0%	0%	-	0%
0%	-	0%	0%	0%	0%	-	0%
0%	-	0%	0%	0%	0%	-	0%
0%	-	0%	0%	0%	0%	-	0%
0%	-	0%	0%	0%	0%	-	0%
0%	-	0%	0%	0%	0%	-	0%
0%	-	0%	0%	0%	0%	-	0%
0%	-	0%	0%	0%	0%	-	0%
0%	-	0%	0%	0%	0%	-	0%
0%	-	0%	0%	0%	0%	-	0%
0%	-	0%	0%	0%	0%	-	0%
0%	-	0%	0%	0%	0%	-	0%
0%	-	0%	0%	0%	0%	-	0%
0%	-	0%	0%	0%	0%	-	0%
0%	-	0%	0%	0%	0%	-	0%
0%	-	0%	0%	0%	0%	-	0%
0%	-	0%	0%	0%	0%	-	0%
0%	-	0%	0%	0%	0%	-	0%
0%	-	0%	0%	0%	0%	-	0%
0%	-	0%	0%	0%	0%	-	0%

9%	2,43	0%	100%	0%	0%	-	0%
0%	-	0%	0%	0%	0%	-	0%
0%	-	0%	0%	0%	0%	-	0%
0%	-	0%	0%	0%	0%	-	0%
12%	6,84	100%	0%	0%	0%	-	0%
40%	-	0%	100%	0%	0%	-	0%
0%	-	0%	0%	0%	0%	-	0%
0%	-	0%	0%	0%	0%	-	0%
0%	-	0%	0%	0%	0%	-	0%
0%	-	0%	0%	0%	0%	-	0%
0%	-	0%	0%	0%	0%	-	0%
0%	-	0%	0%	0%	0%	-	0%
0%	-	0%	0%	0%	0%	-	0%
0%	-	0%	0%	0%	0%	-	0%
0%	-	0%	0%	0%	0%	-	0%
0%	-	0%	0%	0%	0%	-	0%
0%	-	0%	0%	0%	0%	-	0%
0%	-	0%	0%	0%	0%	-	0%
0%	-	0%	0%	0%	0%	-	0%
0%	-	0%	0%	0%	0%	-	0%
0%	-	0%	0%	0%	0%	-	0%
0%	-	0%	0%	0%	0%	-	0%
0%	-	0%	0%	0%	0%	-	0%
0%	-	0%	0%	0%	0%	-	0%
0%	-	0%	0%	0%	0%	-	0%
0%	-	0%	0%	0%	0%	-	0%
0%	-	0%	0%	0%	0%	-	0%
0%	-	0%	0%	0%	0%	-	0%
0%	-	0%	0%	0%	0%	-	0%
0%	-	0%	0%	0%	0%	-	0%
0%	-	0%	0%	0%	0%	-	0%
0%	-	0%	0%	0%	0%	-	0%
0%	-	0%	0%	0%	0%	-	0%
0%	-	0%	0%	0%	0%	-	0%
0%	-	0%	0%	0%	0%	-	0%
0%	-	0%	0%	0%	0%	-	0%
0%	-	0%	0%	0%	0%	-	0%
0%	-	0%	0%	0%	0%	-	0%
0%	-	0%	0%	0%	0%	-	0%
0%	-	0%	0%	0%	0%	-	0%
0%	-	0%	0%	0%	0%	-	0%
0%	-	0%	0%	0%	0%	-	0%
0%	-	0%	0%	0%	0%	-	0%
0%	-	0%	0%	0%	0%	-	0%
0%	-	0%	0%	0%	0%	-	0%
0%	-	0%	0%	0%	0%	-	0%
0%	-	0%	0%	0%	0%	-	0%
0%	-	0%	0%	0%	0%	-	0%
0%	-	0%	0%	0%	0%	-	0%
0%	-	0%	0%	0%	0%	-	0%
0%	-	0%	0%	0%	0%	-	0%
0%	-	0%	0%	0%	0%	-	0%
0%	-	0%	0%	0%	0%	-	0%
0%	-	0%	0%	0%	0%	-	0%
0%	-	0%	0%	0%	0%	-	0%
0%	-	0%	0%	0%	0%	-	0%
0%	-	0%	0%	0%	0%	-	0%
0%	-	0%	0%	0%	0%	-	0%
0%	-	0%	0%	0%	0%	-	0%
0%	-	0%	0%	0%	0%	-	0%
0%	-	0%	0%	0%	0%	-	0%
0%	-	0%	0%	0%	0%	-	0%
0%	-	0%	0%	0%	0%	-	0%
0%	-	0%	0%	0%	0%	-	0%
0%	-	0%	0%	0%	0%	-	0%
0%	-	0%	0%	0%	0%	-	0%

0%	-	0%	0%	0%	0%	-	0%
0%	-	0%	0%	0%	0%	-	0%
0%	-	0%	0%	0%	0%	-	0%
0%	-	0%	0%	0%	0%	-	0%
0%	-	0%	0%	0%	0%	-	0%
0%	-	0%	0%	0%	0%	-	0%
0%	-	0%	0%	0%	0%	-	0%
0%	-	0%	0%	0%	0%	-	0%
0%	-	0%	0%	0%	0%	-	0%
0%	-	0%	0%	0%	0%	-	0%
0%	-	0%	0%	0%	0%	-	0%
0%	-	0%	0%	0%	0%	-	0%
0%	-	0%	0%	0%	0%	-	0%
0%	-	0%	0%	0%	0%	-	0%
0%	-	0%	0%	0%	0%	-	0%
0%	-	0%	0%	0%	0%	-	0%
0%	-	0%	0%	0%	0%	-	0%
0%	-	0%	0%	0%	0%	-	0%
0%	-	0%	0%	0%	0%	-	0%
0%	-	0%	0%	0%	0%	-	0%
0%	-	0%	0%	0%	0%	-	0%
0%	-	0%	0%	0%	0%	-	0%
0%	-	0%	0%	0%	0%	-	0%
0%	-	0%	0%	0%	0%	-	0%
0%	-	0%	0%	0%	0%	-	0%
0%	-	0%	0%	0%	0%	-	0%
0%	-	0%	0%	0%	0%	-	0%
0%	-	0%	0%	0%	0%	-	0%
15%	0,86	0%	100%	0%	0%	-	0%
0%	-	0%	0%	0%	0%	-	0%
8%	1,90	100%	0%	0%	0%	-	0%
0%	-	0%	0%	0%	0%	-	0%
0%	-	0%	0%	0%	0%	-	0%
0%	-	0%	0%	0%	0%	-	0%
0%	-	0%	0%	0%	0%	-	0%
0%	-	0%	0%	0%	0%	-	0%
0%	-	0%	0%	0%	0%	-	0%
0%	-	0%	0%	0%	0%	-	0%
0%	-	0%	0%	0%	0%	-	0%
0%	-	0%	0%	0%	0%	-	0%
0%	-	0%	0%	0%	0%	-	0%
0%	-	0%	0%	0%	0%	-	0%
0%	-	0%	0%	0%	0%	-	0%
0%	-	0%	0%	0%	0%	-	0%
0%	-	0%	0%	0%	0%	-	0%
0%	-	0%	0%	0%	0%	-	0%
0%	-	0%	0%	0%	0%	-	0%
0%	-	0%	0%	0%	0%	-	0%
0%	-	0%	0%	0%	0%	-	0%
0%	-	0%	0%	0%	0%	-	0%
0%	-	0%	0%	0%	0%	-	0%
0%	-	0%	0%	0%	0%	-	0%
0%	-	0%	0%	0%	0%	-	0%
0%	-	0%	0%	0%	0%	-	0%
0%	-	0%	0%	0%	0%	-	0%
0%	-	0%	0%	0%	0%	-	0%
0%	-	0%	0%	0%	0%	-	0%
0%	-	0%	0%	0%	0%	-	0%
0%	-	0%	0%	0%	0%	-	0%
0%	-	0%	0%	0%	0%	-	0%
0%	-	0%	0%	0%	0%	-	0%
0%	-	0%	0%	0%	0%	-	0%
0%	-	0%	0%	0%	0%	-	0%
0%	-	0%	0%	0%	0%	-	0%
0%	-	0%	0%	0%	0%	-	0%
0%	-	0%	0%	0%	0%	-	0%

0%	-	0%	0%	0%	0%	-	0%
0%	-	0%	0%	0%	0%	-	0%
0%	-	0%	0%	0%	0%	-	0%
0%	-	0%	0%	0%	0%	-	0%
0%	-	0%	0%	0%	0%	-	0%
0%	-	0%	0%	0%	0%	-	0%
0%	-	0%	0%	0%	0%	-	0%
0%	-	0%	0%	0%	0%	-	0%
0%	-	0%	0%	0%	0%	-	0%
0%	-	0%	0%	0%	0%	-	0%
0%	-	0%	0%	0%	0%	-	0%
0%	-	0%	0%	0%	0%	-	0%
0%	-	0%	0%	0%	0%	-	0%
0%	-	0%	0%	0%	0%	-	0%
0%	-	0%	0%	0%	0%	-	0%
0%	-	0%	0%	0%	0%	-	0%
0%	-	0%	0%	0%	0%	-	0%
0%	-	0%	0%	0%	0%	-	0%
0%	-	0%	0%	0%	0%	-	0%
0%	-	0%	0%	0%	0%	-	0%
0%	-	0%	0%	0%	0%	-	0%
0%	-	0%	0%	0%	0%	-	0%
0%	-	0%	0%	0%	0%	-	0%
0%	-	0%	0%	0%	0%	-	0%
0%	-	0%	0%	0%	0%	-	0%
0%	-	0%	0%	0%	0%	-	0%

% Neutral	% Coral wins	**Total %**
0%	0%	**100%**
0%	0%	**100%**
0%	0%	**100%**
0%	0%	**100%**
0%	0%	**100%**
0%	0%	**0%**
0%	0%	**100%**
0%	0%	**100%**
0%	0%	**100%**
0%	0%	**100%**
0%	0%	**100%**
0%	0%	**100%**
0%	0%	**100%**
0%	0%	**100%**
0%	0%	**100%**
0%	0%	**100%**
0%	0%	**100%**
0%	0%	**100%**
0%	0%	**100%**
0%	0%	**100%**
0%	0%	**100%**
0%	0%	**100%**
0%	0%	**100%**
0%	0%	**100%**
0%	0%	**100%**
0%	0%	**100%**
0%	0%	**100%**
0%	0%	**100%**
0%	0%	**100%**
0%	0%	**100%**
0%	0%	**100%**
0%	0%	**100%**
0%	0%	**100%**
0%	0%	**100%**
0%	0%	**100%**
0%	0%	**100%**
0%	0%	**100%**
0%	0%	**100%**
0%	0%	**100%**
0%	0%	**100%**
0%	0%	**100%**
0%	0%	**100%**
0%	0%	**100%**
0%	0%	**100%**
0%	0%	**100%**
0%	0%	**100%**
0%	0%	**100%**
0%	0%	**100%**
0%	0%	**100%**
0%	0%	**100%**
0%	0%	**100%**
0%	0%	**100%**
0%	0%	**100%**
0%	0%	**100%**
0%	0%	**100%**
0%	0%	**100%**
0%	0%	**100%**
0%	0%	**100%**
0%	0%	**100%**
0%	0%	**100%**
0%	0%	**100%**
0%	0%	**100%**

0%	0%	100%
0%	0%	100%
0%	0%	100%
0%	0%	100%
0%	0%	100%
0%	0%	100%
0%	0%	100%
0%	0%	100%
0%	0%	100%
0%	0%	100%
0%	0%	100%
0%	0%	100%
0%	0%	100%
0%	0%	100%
0%	0%	100%
0%	0%	100%
0%	0%	100%
0%	0%	100%
0%	0%	100%
0%	0%	100%
0%	0%	100%
0%	0%	100%
0%	0%	100%
0%	0%	100%
0%	0%	100%
0%	0%	100%
0%	0%	100%
0%	0%	100%
0%	0%	100%
0%	0%	100%
100%	0%	100%
0%	0%	0%
0%	0%	0%
0%	0%	100%
0%	0%	100%
0%	0%	100%
0%	0%	100%
0%	0%	100%
0%	0%	100%
0%	0%	100%
0%	0%	100%
0%	0%	100%
0%	0%	100%
0%	0%	100%
0%	0%	100%
0%	0%	100%
0%	0%	100%
0%	0%	100%
0%	0%	100%
0%	0%	100%
0%	0%	100%
0%	0%	100%
0%	0%	100%
0%	0%	100%
0%	0%	100%
0%	0%	100%
0%	0%	100%
0%	0%	100%
0%	0%	100%
0%	0%	100%
0%	0%	100%
0%	0%	100%
0%	0%	0%
0%	0%	100%
0%	0%	100%
0%	0%	0%

0%	0%	100%
0%	0%	100%
0%	0%	100%
0%	0%	100%
0%	0%	100%
0%	0%	100%
0%	0%	100%
0%	0%	0%
0%	0%	0%
0%	0%	0%
0%	0%	100%
0%	0%	0%
0%	0%	100%
0%	0%	100%
0%	0%	100%
0%	0%	100%
0%	0%	100%
0%	0%	0%
0%	0%	0%
0%	0%	100%
0%	0%	0%
0%	0%	100%
0%	0%	100%
0%	0%	100%
0%	0%	0%
0%	0%	0%
0%	0%	0%
0%	0%	100%
0%	0%	100%
0%	0%	100%
0%	0%	100%
0%	0%	100%
0%	0%	100%
0%	0%	100%
0%	0%	0%
0%	0%	0%
0%	0%	100%
0%	0%	100%
0%	0%	100%
0%	0%	100%
0%	0%	100%
0%	0%	100%
0%	0%	100%
0%	0%	100%
0%	0%	100%
0%	0%	100%
0%	0%	100%
0%	0%	100%
0%	0%	100%
0%	0%	100%
0%	0%	100%
0%	0%	100%
0%	0%	100%
0%	0%	100%
0%	0%	100%
0%	0%	100%
0%	0%	100%
0%	0%	100%
0%	0%	100%
0%	0%	100%
0%	0%	100%
0%	0%	100%
0%	0%	100%
0%	0%	100%
0%	0%	100%
0%	0%	100%

0%	0%	0%
0%	0%	100%
0%	0%	100%
0%	0%	100%
0%	0%	100%
0%	0%	100%
0%	0%	100%
0%	0%	100%
0%	0%	100%
0%	0%	100%
0%	0%	100%
0%	0%	100%
0%	0%	100%
0%	0%	0%
0%	0%	100%
0%	0%	100%
0%	0%	100%
0%	0%	100%
0%	0%	0%
0%	0%	0%
0%	0%	100%
0%	0%	100%
0%	0%	100%
0%	0%	100%
0%	0%	100%
0%	0%	100%
0%	0%	100%
0%	0%	100%
0%	0%	100%
0%	0%	100%
0%	0%	100%
0%	0%	100%
0%	0%	0%
0%	0%	100%
0%	0%	100%
0%	0%	100%
0%	0%	100%
0%	0%	100%
0%	0%	100%
0%	0%	100%
0%	0%	100%
0%	0%	100%
0%	0%	100%
0%	0%	100%
0%	0%	100%
0%	0%	100%
0%	0%	100%
0%	0%	100%
0%	0%	100%
0%	0%	100%
0%	0%	100%
0%	0%	100%
0%	0%	100%
0%	0%	100%
0%	0%	100%
0%	0%	100%
0%	0%	100%
0%	0%	100%
0%	0%	100%
0%	0%	100%
0%	0%	100%
0%	0%	100%
0%	0%	100%
0%	0%	100%
0%	0%	100%
0%	0%	100%

0%	0%	100%
0%	0%	100%
0%	0%	100%
0%	0%	100%
0%	0%	100%
0%	0%	100%
0%	0%	100%
0%	0%	100%
0%	0%	100%
0%	0%	100%
0%	0%	100%
0%	0%	100%
0%	0%	100%
0%	0%	100%
0%	0%	100%
0%	0%	100%
0%	0%	100%
0%	0%	100%
0%	0%	100%
0%	0%	100%
0%	0%	100%
0%	0%	100%
0%	0%	100%
0%	0%	100%
0%	0%	100%
0%	0%	100%
0%	0%	100%
0%	0%	100%
0%	0%	100%
0%	0%	100%
0%	0%	100%
0%	0%	100%
0%	0%	0%
0%	0%	100%
0%	0%	100%
0%	0%	100%
0%	0%	100%
0%	0%	100%
0%	0%	100%
0%	0%	100%
0%	0%	100%
0%	0%	100%
0%	0%	100%
0%	0%	100%
0%	0%	100%
0%	0%	100%
0%	0%	100%
0%	0%	100%
0%	0%	100%
0%	0%	100%
0%	0%	100%
0%	0%	100%
0%	0%	100%
0%	0%	100%
0%	0%	100%
0%	0%	100%
0%	0%	100%
0%	0%	100%
0%	0%	100%
0%	0%	100%
0%	0%	100%
0%	0%	100%
0%	0%	100%
0%	0%	100%
0%	0%	100%
0%	0%	0%

0%	*0%*	***100%***
0%	*0%*	***100%***
0%	*0%*	***100%***
0%	*0%*	***100%***
0%	*0%*	***100%***
0%	*0%*	***100%***
0%	*0%*	***100%***
0%	*0%*	***100%***
0%	*0%*	***100%***
0%	*0%*	***100%***
0%	*0%*	***100%***
0%	*0%*	***100%***
0%	*0%*	***100%***
0%	*0%*	***100%***
0%	*0%*	***100%***
0%	*0%*	***100%***
0%	*0%*	***100%***
0%	*0%*	***100%***
0%	*0%*	***100%***
0%	*0%*	***100%***
0%	*0%*	***100%***
0%	*0%*	***100%***
0%	*0%*	***100%***
0%	*0%*	***100%***
0%	*0%*	***100%***
0%	*0%*	***100%***
0%	*0%*	***100%***
0%	*0%*	***100%***
0%	*0%*	***100%***
0%	*0%*	***100%***
0%	*0%*	***100%***
0%	*0%*	***100%***
0%	*0%*	***100%***
0%	*0%*	***100%***
0%	*0%*	***100%***
0%	*0%*	***100%***
0%	*0%*	***100%***
0%	*0%*	***100%***
0%	*0%*	***100%***
0%	*0%*	***100%***
0%	*0%*	***100%***
0%	*0%*	***100%***
0%	*0%*	***100%***
0%	*0%*	***100%***
0%	*0%*	***100%***
0%	*0%*	***100%***
0%	*0%*	***100%***
0%	*0%*	***100%***
0%	*0%*	***100%***
0%	*0%*	***100%***
0%	*0%*	***100%***
0%	*0%*	***100%***
0%	*0%*	***100%***
0%	*0%*	***100%***
0%	*0%*	***100%***
0%	*0%*	***100%***
0%	*0%*	***100%***
0%	*0%*	***100%***
0%	*0%*	***100%***
0%	*0%*	***100%***
0%	*0%*	***100%***
0%	*0%*	***100%***
0%	*0%*	***100%***
0%	*0%*	***100%***
0%	*0%*	***100%***
0%	*0%*	***100%***

0%	0%	100%
0%	0%	100%
0%	0%	100%
0%	0%	100%
0%	0%	100%
0%	0%	100%
0%	0%	100%
0%	0%	100%
0%	0%	100%
0%	0%	0%
0%	0%	0%
0%	0%	100%
0%	0%	100%
0%	0%	100%
0%	0%	0%
0%	0%	100%
0%	0%	100%
0%	0%	100%
0%	0%	100%
0%	0%	100%
0%	0%	100%
0%	0%	100%
0%	0%	100%
0%	0%	100%
0%	0%	100%
0%	0%	100%
0%	0%	100%
0%	0%	100%
0%	0%	100%
0%	0%	100%
0%	0%	100%
0%	0%	100%
0%	0%	100%
0%	0%	100%
0%	0%	100%
0%	0%	100%
0%	0%	0%
0%	0%	0%
0%	0%	100%
0%	0%	100%
0%	0%	100%
0%	0%	100%
0%	0%	0%
0%	0%	0%
0%	0%	100%
0%	0%	0%
0%	0%	100%
0%	0%	100%
0%	0%	0%
0%	0%	0%
0%	0%	0%
0%	0%	0%
0%	0%	100%
0%	0%	100%
0%	0%	100%
0%	0%	100%
0%	0%	100%
0%	0%	100%
0%	0%	100%
0%	0%	100%
0%	0%	100%
0%	0%	100%
0%	0%	100%
0%	0%	100%
0%	0%	100%
0%	0%	100%

0%	*0%*	***100%***
0%	*0%*	***100%***
0%	*0%*	***100%***
0%	*0%*	***0%***
0%	*0%*	***100%***
0%	*0%*	***100%***
0%	*0%*	***100%***
0%	*0%*	***100%***
0%	*0%*	***100%***
0%	*0%*	***100%***
0%	*0%*	***100%***
0%	*0%*	***100%***
0%	*0%*	***100%***
0%	*0%*	***0%***
0%	*0%*	***100%***
0%	*0%*	***100%***
0%	*0%*	***100%***
0%	*0%*	***100%***
0%	*0%*	***100%***
0%	*0%*	***100%***
0%	*0%*	***100%***
0%	*0%*	***100%***
0%	*0%*	***100%***
0%	*0%*	***100%***
0%	*0%*	***0%***
0%	*0%*	***100%***
0%	*0%*	***100%***
0%	*0%*	***100%***
0%	*0%*	***100%***
0%	*0%*	***100%***
0%	*0%*	***100%***
0%	*0%*	***100%***
0%	*0%*	***100%***
0%	*0%*	***100%***
0%	*0%*	***100%***
0%	*0%*	***100%***
0%	*0%*	***100%***
0%	*0%*	***100%***
0%	*0%*	***100%***
0%	*0%*	***100%***
0%	*0%*	***100%***
0%	*0%*	***100%***
0%	*0%*	***0%***
0%	*0%*	***100%***
0%	*0%*	***0%***
0%	*0%*	***100%***
0%	*0%*	***100%***
0%	*0%*	***100%***
0%	*0%*	***100%***
0%	*0%*	***100%***
0%	*0%*	***100%***
0%	*0%*	***0%***
0%	*0%*	***100%***
0%	*0%*	***100%***
0%	*0%*	***100%***
0%	*0%*	***100%***
0%	*0%*	***100%***
0%	*0%*	***100%***
0%	*0%*	***100%***
0%	*0%*	***100%***
0%	*0%*	***100%***
0%	*0%*	***0%***
0%	*0%*	***100%***
0%	*0%*	***100%***
0%	*0%*	***100%***
0%	*0%*	***0%***

0%	*0%*	***100%***
0%	*0%*	***0%***
0%	*0%*	***0%***
0%	*0%*	***100%***
0%	*0%*	***100%***
0%	*0%*	***100%***
0%	*0%*	***100%***
0%	*0%*	***100%***
0%	*0%*	***100%***
0%	*0%*	***100%***
0%	*0%*	***0%***
0%	*0%*	***100%***
0%	*0%*	***100%***
0%	*0%*	***100%***
0%	*0%*	***100%***
0%	*0%*	***100%***
0%	*0%*	***100%***
0%	*0%*	***0%***
0%	*0%*	***100%***
0%	*0%*	***100%***
0%	*0%*	***100%***
0%	*0%*	***100%***
0%	*0%*	***0%***
0%	*0%*	***100%***
0%	*0%*	***100%***
0%	*0%*	***100%***
0%	*0%*	***100%***
0%	*0%*	***100%***
0%	*0%*	***100%***
0%	*0%*	***100%***
0%	*0%*	***100%***
0%	*0%*	***100%***
0%	*0%*	***100%***
0%	*0%*	***100%***
0%	*0%*	***100%***
0%	*0%*	***0%***
0%	*0%*	***100%***
0%	*0%*	***100%***
0%	*0%*	***100%***
0%	*0%*	***100%***
0%	*0%*	***100%***
0%	*0%*	***100%***
0%	*0%*	***100%***
0%	*0%*	***0%***
0%	*0%*	***100%***
0%	*0%*	***0%***
0%	*0%*	***0%***
0%	*0%*	***100%***
0%	*0%*	***100%***
0%	*0%*	***100%***
0%	*0%*	***100%***
0%	*0%*	***100%***
0%	*0%*	***100%***
0%	*0%*	***100%***
0%	*0%*	***100%***
0%	*0%*	***100%***
0%	*0%*	***100%***
0%	*0%*	***100%***
0%	*0%*	***100%***
0%	*0%*	***100%***
0%	*0%*	***100%***
0%	*0%*	***100%***
0%	*0%*	***100%***
0%	*0%*	***100%***
0%	*0%*	***100%***
0%	*0%*	***100%***

0%	*0%*	***100%***
0%	*0%*	***100%***
0%	*0%*	***0%***
0%	*0%*	***100%***
0%	*0%*	***100%***
0%	*0%*	***0%***
0%	*0%*	***100%***
0%	*0%*	***100%***
0%	*0%*	***100%***
0%	*0%*	***100%***
0%	*0%*	***100%***
0%	*0%*	***100%***
0%	*0%*	***100%***
0%	*0%*	***100%***
0%	*0%*	***100%***
0%	*0%*	***100%***
0%	*0%*	***100%***
0%	*0%*	***100%***
0%	*0%*	***100%***
0%	*0%*	***100%***
0%	*0%*	***0%***
0%	*0%*	***100%***
0%	*0%*	***100%***
0%	*0%*	***100%***
0%	*0%*	***100%***
0%	*0%*	***0%***
0%	*0%*	***0%***
0%	*0%*	***100%***
0%	*0%*	***100%***
0%	*0%*	***100%***
0%	*0%*	***100%***
0%	*0%*	***100%***
0%	*0%*	***100%***
0%	*0%*	***100%***
0%	*0%*	***0%***
0%	*0%*	***0%***
0%	*0%*	***100%***
0%	*0%*	***100%***
0%	*0%*	***100%***
0%	*0%*	***100%***
0%	*0%*	***100%***
0%	*0%*	***100%***
0%	*0%*	***100%***
0%	*0%*	***100%***
0%	*0%*	***0%***
0%	*0%*	***100%***
0%	*0%*	***0%***
0%	*0%*	***100%***
0%	*0%*	***100%***
0%	*0%*	***100%***
0%	*0%*	***100%***
0%	*0%*	***0%***
0%	*0%*	***0%***
0%	*0%*	***100%***
0%	*0%*	***0%***
0%	*0%*	***100%***
0%	*0%*	***100%***
0%	*0%*	***0%***
0%	*0%*	***100%***
0%	*0%*	***100%***
0%	*0%*	***100%***
0%	*0%*	***100%***
0%	*0%*	***100%***
0%	*0%*	***100%***
0%	*0%*	***100%***
0%	*0%*	***0%***

0%	0%	0%
0%	0%	100%
0%	0%	100%
0%	0%	100%
0%	0%	100%
0%	0%	100%
0%	0%	100%
0%	0%	100%
0%	0%	100%
0%	0%	100%
0%	0%	100%
0%	0%	100%
0%	0%	0%
0%	0%	100%
0%	0%	100%
0%	0%	100%
0%	0%	100%
0%	0%	100%
0%	0%	100%
0%	0%	100%
0%	0%	100%
0%	0%	100%
0%	0%	100%
0%	0%	0%
0%	0%	100%
0%	0%	0%
0%	0%	0%
0%	0%	0%
0%	0%	100%
0%	0%	100%
0%	0%	100%
0%	0%	100%
0%	0%	100%
0%	0%	100%
0%	0%	100%
0%	0%	100%
0%	0%	100%
0%	0%	100%
0%	0%	100%
0%	0%	0%
0%	0%	0%
0%	0%	100%
0%	0%	0%
0%	0%	100%
0%	0%	100%
0%	0%	0%
0%	0%	100%
0%	0%	100%
0%	0%	100%
0%	0%	100%
0%	0%	0%
0%	0%	100%
0%	0%	100%
0%	0%	100%
0%	0%	100%
0%	0%	100%
0%	0%	100%
0%	0%	100%
0%	0%	100%
0%	0%	100%
0%	0%	100%
0%	0%	100%
0%	0%	100%
0%	0%	100%
0%	0%	100%
0%	0%	100%

0%	0%	100%
0%	0%	100%
0%	0%	100%
0%	0%	100%
0%	0%	100%
0%	0%	0%
0%	0%	100%
0%	0%	100%
0%	0%	100%
0%	0%	100%
0%	0%	100%
0%	0%	100%
0%	0%	100%
0%	0%	100%
0%	0%	100%
0%	0%	100%
0%	0%	100%
0%	0%	100%
0%	0%	100%
0%	0%	100%
0%	0%	100%
0%	0%	100%
0%	0%	100%
0%	0%	100%
0%	0%	100%
0%	0%	100%
0%	0%	100%
0%	0%	100%
0%	0%	100%
0%	0%	100%
0%	0%	100%
0%	0%	100%
0%	0%	100%
0%	0%	100%
0%	0%	100%
0%	0%	100%
0%	0%	100%
0%	0%	100%
0%	0%	100%
0%	0%	100%
0%	0%	100%
0%	0%	100%
0%	0%	100%
0%	0%	100%
0%	0%	100%
0%	0%	0%
0%	0%	100%
0%	0%	100%
0%	0%	100%
0%	0%	100%
0%	0%	0%
0%	0%	100%
0%	0%	100%
0%	0%	100%
0%	0%	100%
0%	0%	100%
0%	0%	100%
0%	0%	100%
0%	0%	100%
0%	0%	100%
0%	0%	100%
0%	0%	100%
0%	0%	100%
0%	0%	100%
0%	0%	100%
0%	0%	100%

0%	0%	100%
0%	0%	100%
0%	0%	100%
0%	0%	100%
0%	0%	0%
0%	0%	100%
0%	0%	100%
0%	0%	100%
0%	0%	100%
0%	0%	100%
0%	0%	100%
0%	0%	100%
0%	0%	0%
0%	0%	0%
0%	0%	100%
0%	0%	100%
0%	0%	100%
0%	0%	100%
0%	0%	100%
0%	0%	100%
0%	0%	100%
0%	0%	100%
0%	0%	100%
0%	0%	100%
0%	0%	100%
0%	0%	0%
0%	0%	100%
0%	0%	100%
0%	0%	100%
0%	0%	100%
0%	0%	100%
0%	0%	100%
0%	0%	100%
0%	0%	100%
0%	0%	100%
0%	0%	100%
0%	0%	100%
0%	0%	100%
0%	0%	100%
0%	0%	0%
0%	0%	100%
0%	0%	100%
0%	0%	100%
0%	0%	100%
0%	0%	100%
0%	0%	100%
0%	0%	100%
0%	0%	100%
0%	0%	100%
0%	0%	100%
0%	0%	100%
0%	0%	100%
0%	0%	100%
0%	0%	100%
0%	0%	100%
0%	0%	100%
0%	0%	100%
0%	0%	100%
0%	0%	100%
0%	0%	100%
0%	0%	100%
0%	0%	100%
0%	0%	100%
0%	0%	100%
0%	0%	100%
0%	0%	100%

0%	0%	100%
0%	0%	100%
0%	0%	100%
0%	0%	100%
0%	0%	100%
0%	0%	100%
0%	0%	100%
0%	0%	100%
0%	0%	100%
0%	0%	100%
0%	0%	100%
0%	0%	0%
0%	0%	100%
0%	0%	100%
0%	0%	100%
0%	0%	100%
0%	0%	100%
0%	0%	0%
0%	0%	100%
0%	0%	100%
0%	0%	100%
0%	0%	100%
0%	0%	100%
0%	0%	100%
0%	0%	100%
0%	0%	100%
0%	0%	100%
0%	0%	100%
0%	0%	100%
0%	0%	100%
0%	0%	100%
0%	0%	100%
0%	0%	0%
0%	0%	100%
0%	0%	100%
0%	0%	100%
0%	0%	0%
0%	0%	100%
0%	0%	100%
0%	0%	100%
0%	0%	0%
0%	0%	100%
0%	0%	100%
0%	0%	100%
0%	0%	100%
0%	0%	100%
0%	0%	100%
0%	0%	100%
0%	0%	100%
0%	0%	100%
0%	0%	100%
0%	0%	100%
0%	0%	100%
0%	0%	100%
0%	0%	100%
0%	0%	100%
0%	0%	100%
0%	0%	100%
0%	0%	100%
0%	0%	100%
0%	0%	100%
0%	0%	100%
0%	0%	100%
0%	0%	100%
0%	0%	100%
0%	0%	100%

0%	0%	100%
0%	0%	100%
0%	0%	100%
0%	0%	100%
0%	0%	100%
0%	0%	100%
0%	0%	100%
0%	0%	100%
0%	0%	100%
0%	0%	100%
0%	0%	100%
0%	0%	100%
0%	0%	100%
0%	0%	100%
0%	0%	100%
0%	0%	100%
0%	0%	100%
0%	0%	100%
0%	0%	100%
0%	0%	100%
0%	0%	100%
0%	0%	100%
0%	0%	100%
0%	0%	100%
0%	0%	100%
0%	0%	100%
0%	0%	0%
0%	0%	100%
0%	0%	100%
0%	0%	100%
0%	0%	100%
0%	0%	100%
0%	0%	100%
0%	0%	100%
0%	0%	100%
0%	0%	100%
0%	0%	100%
0%	0%	100%
0%	0%	100%
0%	0%	100%
0%	0%	100%
0%	0%	100%
0%	0%	0%
0%	0%	100%
0%	0%	100%
0%	0%	100%
0%	0%	100%
0%	0%	100%
0%	0%	0%
0%	0%	100%
0%	0%	100%
0%	0%	0%
0%	0%	0%
0%	0%	100%
0%	0%	100%
0%	0%	100%
0%	0%	100%
0%	0%	100%
0%	0%	0%
0%	0%	100%
0%	0%	100%
0%	0%	100%
0%	0%	100%
0%	0%	100%
0%	0%	100%
0%	0%	100%

0%	0%	100%
0%	0%	0%
0%	0%	100%
0%	0%	100%
0%	0%	100%
0%	0%	100%
0%	0%	100%
0%	0%	100%
0%	0%	100%
0%	0%	100%
0%	0%	100%
0%	0%	100%
0%	0%	100%
0%	0%	100%
0%	0%	100%
0%	0%	100%
0%	0%	100%
0%	0%	100%
0%	0%	100%
0%	0%	100%
0%	0%	100%
0%	0%	100%
0%	0%	100%
0%	0%	100%
0%	0%	100%
0%	0%	100%
0%	0%	100%
0%	0%	100%
0%	0%	100%
0%	0%	100%
0%	0%	100%
0%	0%	0%
0%	0%	0%
0%	0%	100%
0%	0%	100%
0%	0%	100%
0%	0%	100%
0%	0%	100%
0%	0%	100%
0%	0%	100%
0%	0%	100%
0%	0%	100%
0%	0%	100%
0%	0%	100%
0%	0%	100%
0%	0%	100%
0%	0%	100%
0%	0%	100%
0%	0%	100%
0%	0%	100%
0%	0%	100%
0%	0%	100%
0%	0%	100%
0%	0%	100%
0%	0%	100%
0%	0%	100%
0%	0%	100%
0%	0%	100%
0%	0%	100%
0%	0%	100%
0%	0%	100%
0%	0%	100%
0%	0%	100%
0%	0%	100%
0%	0%	100%
0%	0%	100%

0%	*0%*	***100%***
0%	*0%*	***100%***
0%	*0%*	***100%***
0%	*0%*	***100%***
0%	*0%*	***100%***
0%	*0%*	***100%***
0%	*0%*	***100%***
0%	*0%*	***100%***
0%	*0%*	***100%***
0%	*0%*	***100%***
0%	*0%*	***100%***
0%	*0%*	***100%***
0%	*0%*	***100%***
0%	*0%*	***100%***
0%	*0%*	***100%***
0%	*0%*	***100%***
0%	*0%*	***100%***
0%	*0%*	***100%***
0%	*0%*	***100%***
0%	*0%*	***100%***
0%	*0%*	***0%***
0%	*0%*	***100%***
0%	*0%*	***100%***
0%	*0%*	***100%***
0%	*0%*	***100%***
0%	*0%*	***100%***
0%	*0%*	***100%***
0%	*0%*	***100%***
0%	*0%*	***100%***
0%	*0%*	***100%***
0%	*0%*	***100%***
0%	*0%*	***100%***
0%	*0%*	***100%***
0%	*0%*	***100%***
0%	*0%*	***100%***
0%	*0%*	***100%***
0%	*0%*	***100%***
0%	*0%*	***0%***
0%	*0%*	***100%***
0%	*0%*	***100%***
0%	*0%*	***100%***
0%	*0%*	***100%***
0%	*0%*	***0%***
0%	*0%*	***100%***
0%	*0%*	***0%***
0%	*0%*	***0%***
0%	*0%*	***0%***
0%	*0%*	***100%***
0%	*0%*	***100%***
0%	*0%*	***100%***
0%	*0%*	***100%***
0%	*0%*	***100%***
0%	*0%*	***100%***
0%	*0%*	***100%***
0%	*0%*	***100%***
0%	*0%*	***100%***
0%	*0%*	***100%***
0%	*0%*	***100%***
0%	*0%*	***0%***
0%	*0%*	***0%***
0%	*0%*	***100%***
0%	*0%*	***100%***
0%	*0%*	***100%***
0%	*0%*	***100%***
0%	*0%*	***100%***
0%	*0%*	***100%***

0%	0%	100%
0%	0%	100%
0%	0%	100%
0%	0%	100%
0%	0%	100%
0%	0%	100%
0%	0%	100%
0%	0%	100%
0%	0%	100%
0%	0%	100%
0%	0%	100%
0%	0%	100%
0%	0%	100%
0%	0%	100%
0%	0%	100%
0%	0%	100%
0%	0%	100%
0%	0%	0%
0%	0%	0%
0%	0%	100%
0%	0%	0%
0%	0%	0%
0%	0%	0%
0%	0%	100%
0%	0%	0%
0%	0%	0%
0%	0%	100%
0%	0%	100%
0%	0%	100%
0%	0%	100%
0%	0%	100%
0%	0%	100%
0%	0%	100%
0%	0%	100%
0%	0%	100%
0%	0%	100%
0%	0%	100%
0%	0%	0%
0%	0%	100%
0%	0%	100%
0%	0%	100%
0%	0%	100%
0%	0%	100%
0%	0%	100%
0%	0%	100%
0%	0%	100%
0%	0%	100%
0%	0%	100%
0%	0%	100%
0%	0%	100%
0%	0%	100%
0%	0%	100%
0%	0%	100%
0%	0%	100%
0%	0%	0%
0%	0%	100%
0%	0%	100%
0%	0%	100%
0%	0%	100%
0%	0%	0%
0%	0%	100%
0%	0%	100%
0%	0%	0%
0%	0%	100%
0%	0%	100%
0%	0%	100%

0%	0%	100%
0%	0%	100%
0%	0%	0%
0%	0%	100%
0%	0%	100%
0%	0%	0%
0%	0%	100%
0%	0%	100%
0%	0%	100%
0%	0%	100%
0%	0%	0%
0%	0%	100%
0%	0%	100%
0%	0%	100%
0%	0%	100%
0%	0%	100%
0%	0%	100%
0%	0%	100%
0%	0%	100%
0%	0%	100%
0%	0%	100%
0%	0%	100%
0%	0%	0%
0%	0%	100%
0%	0%	100%
0%	0%	100%
0%	0%	0%
0%	0%	100%
0%	0%	100%
0%	0%	100%
0%	0%	100%
0%	0%	100%
0%	0%	0%
0%	0%	0%
0%	0%	0%
0%	0%	100%
0%	0%	0%
0%	0%	0%
0%	0%	0%
0%	0%	0%
0%	0%	100%
0%	0%	0%
0%	0%	0%
0%	0%	0%
0%	0%	100%
0%	0%	100%
0%	0%	0%
0%	0%	100%
0%	0%	0%
0%	0%	100%
0%	0%	100%
0%	0%	100%
0%	0%	100%
0%	0%	100%
0%	0%	100%
0%	0%	100%
0%	0%	100%
0%	0%	100%
0%	0%	100%
0%	0%	100%
0%	0%	100%
0%	0%	0%
0%	0%	100%
0%	0%	100%
0%	0%	100%
0%	0%	100%

0%	*0%*	***100%***
0%	*0%*	***100%***
0%	*0%*	***100%***
0%	*0%*	***100%***
0%	*0%*	***100%***
0%	*0%*	***100%***
0%	*0%*	***100%***
0%	*0%*	***100%***
0%	*0%*	***100%***
0%	*0%*	***0%***
0%	*0%*	***100%***
0%	*0%*	***100%***
0%	*0%*	***100%***
0%	*0%*	***100%***
0%	*0%*	***100%***
0%	*0%*	***0%***
0%	*0%*	***100%***
0%	*0%*	***100%***
0%	*0%*	***100%***
0%	*0%*	***100%***
0%	*0%*	***100%***
0%	*0%*	***100%***
0%	*0%*	***100%***
0%	*0%*	***0%***
0%	*0%*	***100%***
0%	*0%*	***100%***
